# Microarray profiling and functional analysis of differentially expressed plasma exosomal circular RNAs in Graves’ disease

**DOI:** 10.1186/s40659-020-00299-y

**Published:** 2020-07-29

**Authors:** Ying Sun, Wei Wang, Yuxiao Tang, Daping Wang, Liang Li, Min Na, Guantong Jiang, Qian Li, Shulin Chen, Jin Zhou

**Affiliations:** 1 Department of Endocrinology, Affiliated Yantai Yuhuangding Hospital of Qingdao University Medical College, Yantai, Shandong China; 2 Department of Neurosurgery, Affiliated Zhongshan Hospital of Dalian University, Dalian, Liaoning China; 3 Department of Radiology, Dalian Sixth People’s Hospital, Dalian, Liaoning China; 4 Department of Endocrinology and Metabolism, Binzhou Medical University Hospital, Binzhou, Shandong China; 5 Department of Scientific Research, Affiliated Yantai Yuhuangding Hospital of Qingdao University Medical College, Yantai, Shandong China

**Keywords:** Circular RNA, Graves’ disease, Exosomes, hsa_circRNA_000102, Immune activation

## Abstract

**Background:**

Circulating RNA (circRNA) regulates various bioactivities in cells. A better understanding of the exosomal circRNA can provide novel insights into the pathogenesis and treatment of Graves’ disease (GD). We aimed to profile the differentially expressed circRNAs (DEcRs) in plasma exosomes of patients with GD and speculate and probe the functions of the DEcR by comprehensive bioinformatics analyses.

**Methods:**

Serum exosomes were isolated from five primary GD patients and five healthy controls via ultracentrifugation. After verification with transmission electron microscopy, exosome samples were subjected to microarray profiling using human circRNA microarrays. Two up-regulated and two down-regulated DEcRs were selected for validation in plasma exosomes from 20 GD and 20 healthy control participants using reverse transcriptase-quantitative polymerase chain reaction (RT-qPCR). The circRNA/microRNA/mRNA interaction network was then assembled and the analysis of the Gene Ontology and KEGG (Kyoto Encyclopedia of Genes and Genomes) pathways was utilized to predict the potential functions of the DEcR associated genes.

**Results:**

There were 15 DEcRs revealed in primary GD cases. The intronic circRNA hsa_circRNA_000102 was confirmed as an up-regulated component in plasma exosomes from patients with GD. The circRNA/microRNA/mRNA interaction network unveiled the most potential targeting microRNAs of hsa_circRNA_000102 and its associated genes. The functional analyses predicted involvement of hsa_circRNA_000102 associated genes in pathways of immune system activation, such as viral infection and interferon-beta signaling.

**Conclusions:**

hsa_circRNA_000102 is a differentially up-regulated plasma exosomal circRNA in patients with GD. Our study highlights multiple pathways, particularly virus infection and interferon-beta signaling, for mediating immune activation in Graves’ disease.

## Background

Graves’ disease (GD) is an autoimmune disorder characterized by typical clinical manifestations with unique association of diffuse goiter, ophthalmopathy, and pretibial myxedema [1]. GD is the most common cause of hyperthyroidism, identified in patients spanning the globe [2]. Timely diagnosis and appropriate treatments are critical for the prognosis of patients with GD. A recent observational study in Italy reported that the clinical phenotype of GD is becoming milder, likely due to increased awareness on thyroid disease and the availability of sensitive diagnostic tests, in conjunction with timely and effective treatments [3]. However, cases of extreme hyperthyroidism surface occasionally. Additionally, current treatments for GD, including antithyroid medications, radioactive iodine, and surgery (thyroidectomy), often result in adverse effects. Therefore, the importance of improving the understanding of the molecular and intermolecular interactions and the key signaling pathways affected by Graves’ disease persists.

The pathogenesis of Graves’ disease remains insufficiently elucidated. However, studies revealed GD as a B cell-mediated, T cell-dependent autoimmune disease with multiple types of immune cell involvement [4]. In patients with GD, overproduction of thyroid hormones and thyroid hyperplasia are often caused by stimulatory autoantibodies against the thyroid-stimulating hormone receptor (TSHR), also known as the thyroid-stimulating antibody (TSAb) [5]. Over the course of GD, communication between different immune cells is of great importance [6]. Aside from direct cell-to-cell contact, a novel and perhaps even more complex mode of immune regulation is provided by exosomes that are released by dendritic cells, B cells, T cells, and other cell types [7]. With the diameters ranging from 30 to 150 nm, exosomes are small membrane vesicles of endocytic origin, which are secreted by almost all cell types, displaying diverse functions dependent upon their origin [8]. Exosomes are rich in content and contain protein, lipid, and nucleic acid, which are involved in immune responses, antigen presentation, intercellular communication, protein and RNA transport and other physiological processes [9]. Current research into the genetic etiology of GD suggests that the “hidden” heredity of GD is likely associated with rare genomic variants including noncoding RNAs (ncRNAs), microRNAs (miRNAs), and/or epigenetic factors [10]. The exosomal circular RNAs (circRNAs), rather than those unprotected ncRNAs in blood, potentially represent novel bridges for communication of various immune cells in GD.

The first circular RNA (circRNA) was identified as ‘scrambled exons’ more than 40 years ago [11], but it was only recently more and more evidence of their expression in various diseases have been emerging. Unlike traditional linear RNA, which contains 5’ and 3’ ends, circRNAs have unique structures resulting from a 3′ to 5′ end-joining event (backsplicing). Meanwhile, two different models of circRNA biogenesis have been described, the lariat or exon skipping model and the direct backsplicing model Meanwhile, two different models of circRNA biogenesis have been described, the lariat or exon skipping model and the direct backsplicing model. [12]. CircRNAs can be derived from exon, intron, untranslated or intergenic regions of the genome, and a large amount of them have been identified in mouse and human by high-throughput sequencing and bioinformatics analyses [13]. CircRNA is produced by special selective shear and is abundant in the cytoplasm of eukaryotic cells. It expresses with tissue, timing and disease specificity [14]. Though to the multiple functions of circRNAs recently inferred, the most important is the involvement of circRNAs in post-transcriptional regulation, similar to the roles of an endogenous RNA or miRNA sponge in competitively inhibiting RNA/miRNA transcriptional regulation [15, 16]. In addition, circRNA can also regulate variable shear or transcription and modify the expression of its related genes [17]. Therefore, circRNAs are expected to provide a new molecular basis of prediction, diagnosis and treatment of complex diseases. However, the means and extent to which exosomal circRNAs play a role in the pathogenesis of GD remains completely unknown.

In this study, the implications of circRNA in GD and exosomal circular RNAs were profiled in the serum samples from patients with and without GD through human circRNA microarray. Comprehensive bioinformatics analyses were conducted to explore and probe the functions of the differentially expressed circRNAs.

## Results

### Validation of study cohorts and isolated exosomes

This study involved two cohorts of subjects: the discovery cohort and the validation cohort. The clinical characteristics of the participants in the discovery cohort are summarized in Additional file 1: Table S2. The mean age of patients with Graves’ disease was 36.6 ± 11.5 years as compared to mean age of 37.8 ± 9.6 years among controls (P = 0.86). As expected, Graves’ disease patients had significantly elevated FT3, elevated FT4, and suppressed TSH levels in comparison to controls (Additional file 1: Table S2). Notably, the concentration of plasma TRAb (TSH receptor antibodies, also called TSAb), the specific thyroid autoantibody produced in GD (29.49 ± 14.77 IU/L), was remarkably higher in GD patients than in healthy control subjects (0.55 ± 0.32 IU/L). In the validation cohort (Additional file 1: Table S3), there is no difference in the age of patients with Graves’ disease (37.55 ± 12.18 years) and control groups (37.15 ± 10.89 years) (P = 0.91). TSH(GD group: 0.0145 ± 0.0294 IU/ml vs. control group: 1.851 ± 0.8749 IU/ml), FT4(GD group: 90.66 ± 10.71 pmol/L vs. control group: 16.32 ± 2.06 pmol/L), FT3(GD group: 38.2 ± 10.83 pmol/L vs. control group: 4.85 ± 0.93 pmol/L), had a significant differences between two groups(P < 0.0001). There are significant differences in TRAb between GD (26.13 ± 12.9 IU/L) and healthy group (0.71 ± 0.46 IU/L) (P < 0.0001). The median TgAb level of GD and healthy control groups were 1887(127.6–4000) IU/ml and 20.82(10–114.1) IU/ml, respectively (P < 0.0001). Next, we verified isolated exosomes through observation of their morphology by transmission electron microscopy. As shown in Additional file 1: Figure S1, these exosomes displayed irregular spheres with a clearly defined and relatively intact membrane. They have diameters ranging from 30 nm to 100 nm, distinguishable from other much larger extracellular vesicles like microvesicles and apoptotic bodies.

### Identification of differentially expressed plasma exosomal circRNAs from patients with GD and healthy control subjects

**Fig. 1:**
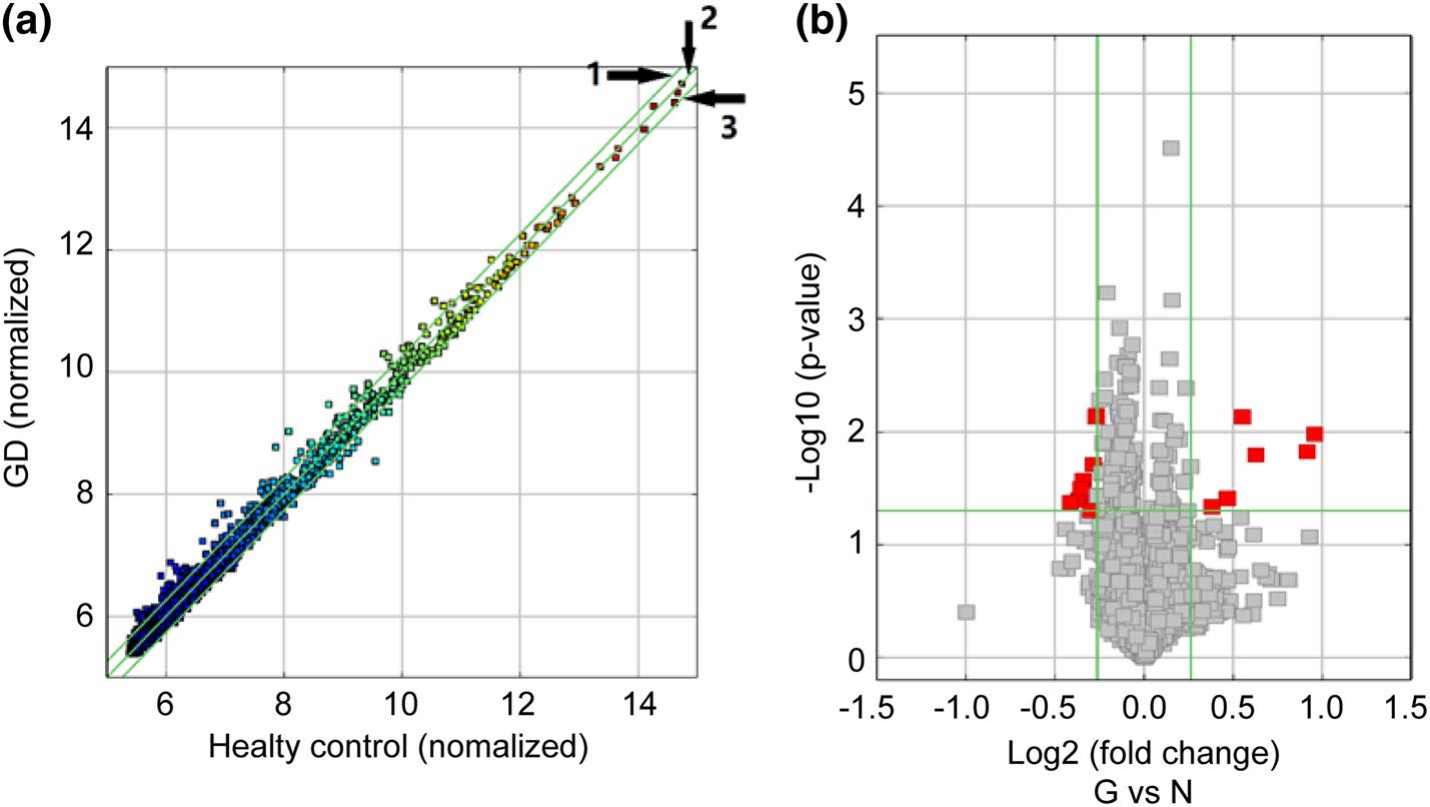
Differential expression of circRNAs in plasma exosomes from patients with GD and healthy control subjects. **a** Scatter plots with the mean expression values of samples in each group. Raw junction reads for all samples were normalized by total mapped read numbers and were transformed by log2. Each dot represents a single circRNA. The exhibiting fold change is 2.0 of Line 1 and Line 3, whereas the dots on Line 2 indicate circRNAs with equal expression between GD group and healthy control group. **b** Volcano plot representation of differentially expressed transcripts and their statistical significance. The fold changes are represented in log2 scale as depicted on the x-axis, whereas the − log10 *P*-value is depicted on the y-axis. Transcripts with greater statistical significance are higher in the plot. The red circles represent circRNA genes that show differential expression with fold changes > 1.2 and *P*-values < 0.05 between the GD and normal control exosomes samples

The plasma exosomes isolated from the discovery cohort were subjected to the microarray profiling of the differentially expressed circRNAs. A number of the 13,000 total circRNAs detected, developed an altered expression. As shown in the scatter plot, denoting the means of the expression values of the two groups (Fig. 1a), many circRNAs, from patients with GD, exhibit more than a twofold up-regulation or down-regulation of plasma exosomes compared to healthy control subjects. There are 15 significantly differentially expressed circRNAs that met the criteria of exhibiting fold changes > 1.2 and *P*-values < 0.05, as shown in the volcano plot representation of differentially expressed transcripts and their statistical significance (Fig. 1b). Among them, six circRNAs were up-regulated and nine circRNAs were down-regulated (Table 1).

**Table 1:** The list of differential expressed circRNAs in plasma exosome samples from patients with GD and healthy control subjects

circRNA ID	P-value	FC (abs)	Regulation	circRNA_type	GeneSymbol
hsa_circRNA_102059	0.038876	1.38207	Up	Exonic	MED1
hsa_circRNA_405965	0.01488	1.884644	Up	Exonic	CCNT2-AS1
hsa_circRNA_008389	0.010443	1.941364	Up	Exonic	DNAJC11
hsa_circRNA_000102	0.015919	1.543085	Up	Intronic	AKNAD1
hsa_circRNA_402094	0.007404	1.466367	Up	Exonic	HKR1
hsa_circRNA_005133	0.04595	1.301035	Up	Exonic	DDR1
hsa_circRNA_405709	0.00715	1.203396	Down	Exonic	MIER2
hsa_circRNA_072697	0.026879	1.267682	Down	Exonic	PPWD1
hsa_circRNA_025500	0.03979	1.288536	Down	Exonic	DUSP16
hsa_circRNA_001067	0.038894	1.214824	Down	Intronic	RP11-351M8.1
hsa_circRNA_043898	0.019398	1.219508	Down	Exonic	EZH1
hsa_circRNA_004939	0.049734	1.231611	Down	Sense overlapping	IL4R
hsa_circRNA_104619	0.007381	1.20487	Down	Exonic	PRKDC
hsa_circRNA_407279	0.031666	1.277486	Down	Exonic	SYTL5
hsa_circRNA_001454	0.027548	1.267223	Down	Exonic	KIAA0922

*FC* fold change (absolute value)

### *hsa_circRNA_000102* is the validated differentially expressed plasma exosomal circRNA in GD

**Fig. 2:**
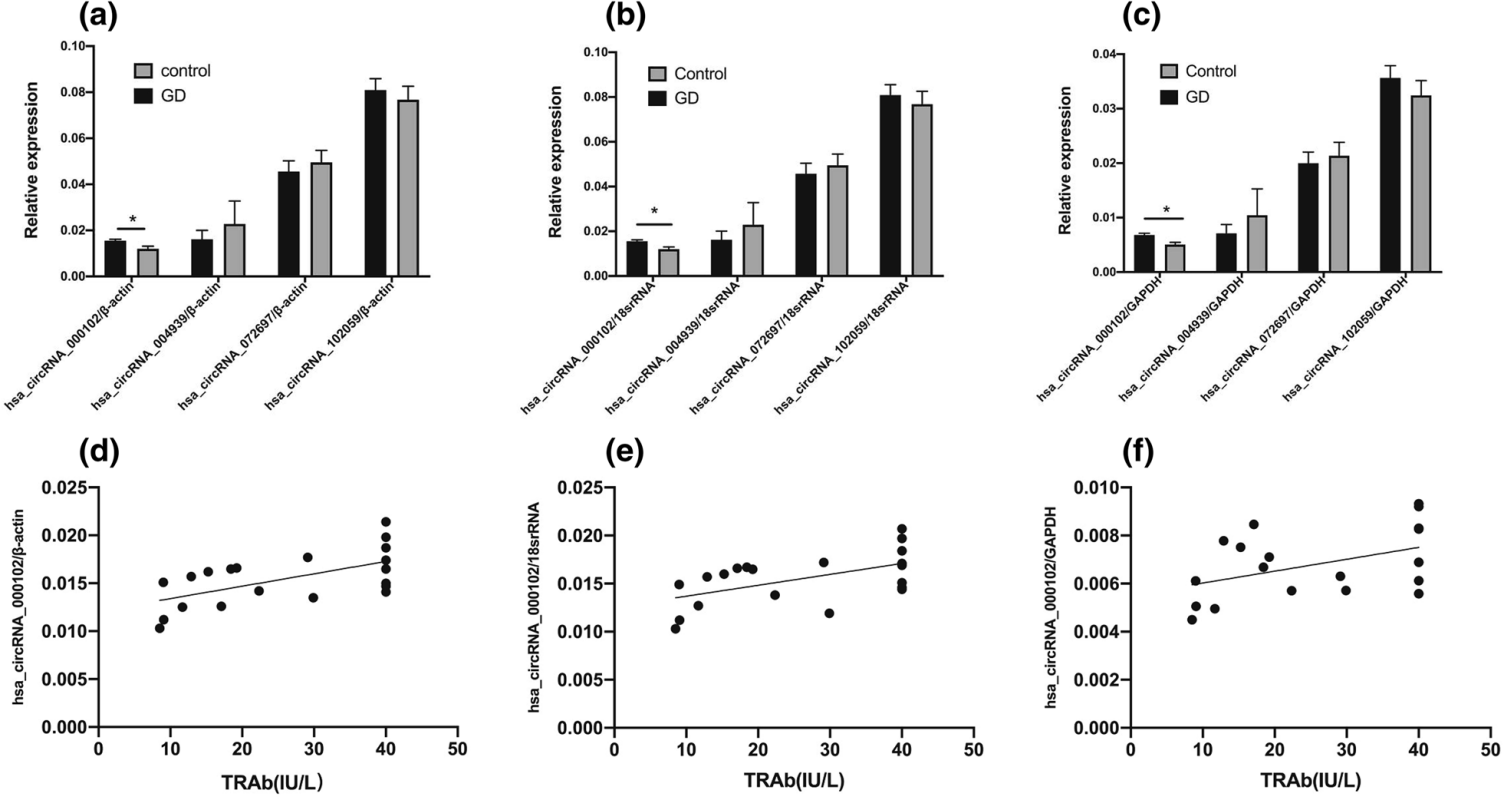
Validation of selected candidate differentially expressed circRNAs by Real-time qPCR. Data represents the mean ± SD of three independent experiments. n = 10 for each group. *P < 0.05

Among 15 significantly differentially expressed circRNAs, 12 are exonic, two are intronic, and one is a combination of both (Table 1). We examined the genes associated with the back splicing-mediated generation of these circRNAs, and found MED1, AKNAD1, PPWD1 and IL4R were related to nuclear co-receptor, transcription factor, mRNA splicing and cytokine receptor pathways, respectively. These molecules and pathways possess the ability for an immune activation, as observed in patients with GD. Therefore, the corresponding four circRNAs, including two up-regulated circRNAs (hsa_circRNA_000102, hsa_circRNA_102059) and two down-regulated circRNAs (hsa_circRNA_004939, hsa_circRNA_072697), were initially selected for further verification by RT-qPCR. As shown in Fig. 2, the consistency between RT-qPCR results and microarray analysis data had been demonstrated only for hsa_circRNA_000102. However, the expression levels of hsa_circRNA_102059, hsa_circRNA_004939, and hsa_circRNA_072697 did not show significant differences between plasma exosomal samples from patients with GD and healthy control subjects (Fig. 2a–c). Furthermore, it was observed that expression levels of hsa_circRNA_000102/β-actin(r = 0.5983, P = 0.0053), hsa_circRNA_000102/18srRNA(r = 0.5462, P = 0.0127), hsa_circRNA_000102/ GAPDH(r = 0.4526, P = 0.0451) correlate significantly with TRAb levels (Fig. 2d–f). Therefore, our next stage of bioinformatics analyses focused on delineating the intermolecular interactions and the key signaling pathways associated with hsa_circRNA_000102.

### Prediction of the circRNA/microRNA/mRNA interaction network for hsa_circRNA_000102

**Fig. 3:**
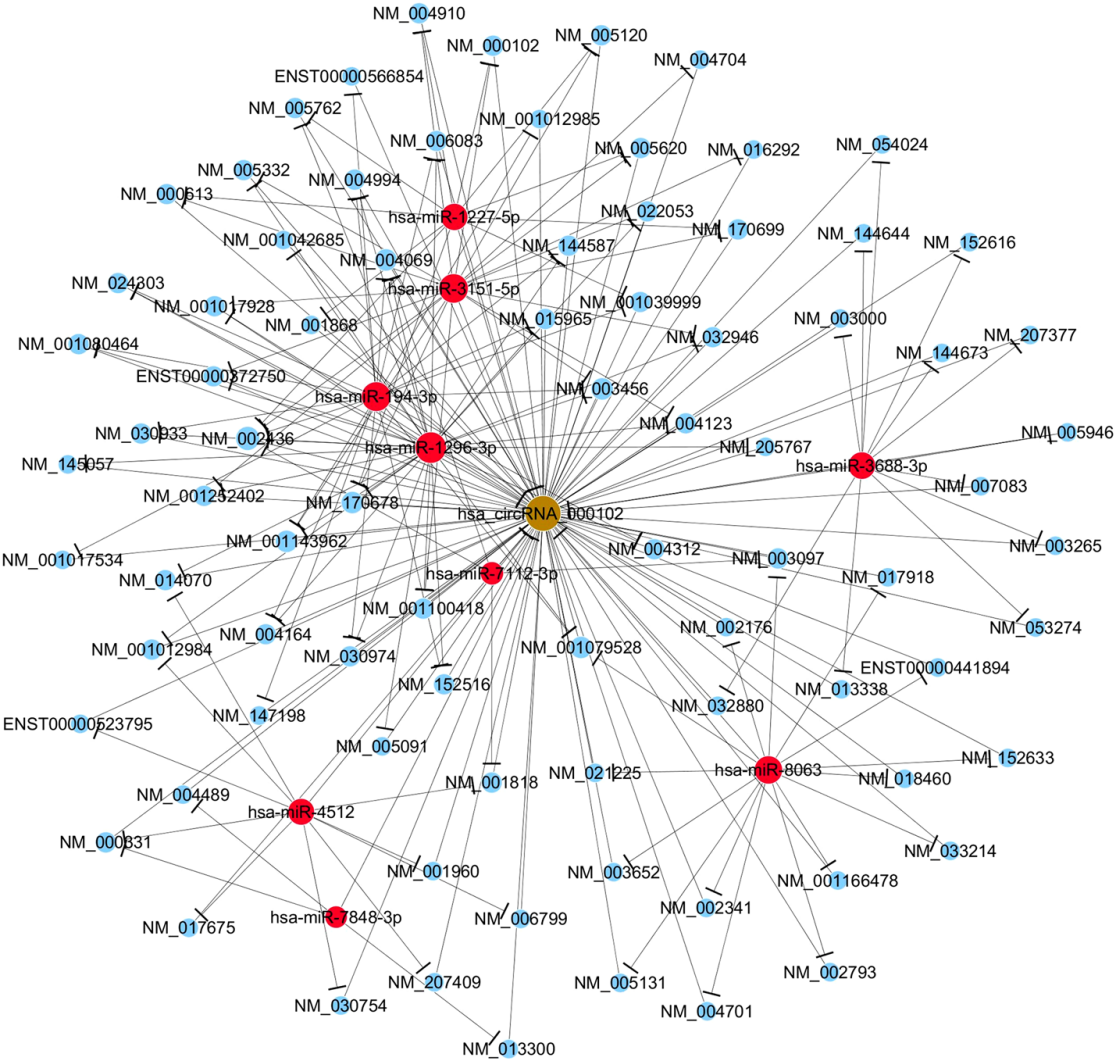
The circRNA-miRNA-mRNA regulatory network of hsa_circRNA_000102. CircRNA, miRNA, and mRNA are indicated as spheres in brown, red, blue color, respectively. *CircRNA* circular RNA, *miRNA* microRNA, *mRNA* messenger RNA

Recent studies on circRNA/microRNA interactions have indicated that circRNAs play a key role in the regulation of gene expression by interacting with their target microRNAs (miRNAs) [18]. To find the potential target miRNAs and mRNA associated with GD, a circRNA/microRNA/mRNA interaction network was constructed to predict the intermolecular interaction for the experimentally confirmed circRNA (hsa_circRNA_000102). An Arraystars homemade miRNA target prediction software based on TargetScan and miRanda was utilized (Fig. 3). There were nine miRNAs (hsa-miR-3151-5p, hsa-miR-1227-5p, hsa-miR-194-3p, hsa-miR-1296-3p, hsa-miR-3688-3p, hsa-miR-7112-3p, hsa-miR-8063, hsa-miR-4512 and hsa-miR-7848-3p) and numerous mRNAs predicted to be interacting with hsa_circRNA_000102 directly or indirectly.

### GO analysis and KEGG pathway analysis of hsa_circRNA_000102 associated genes

**Fig. 4:**
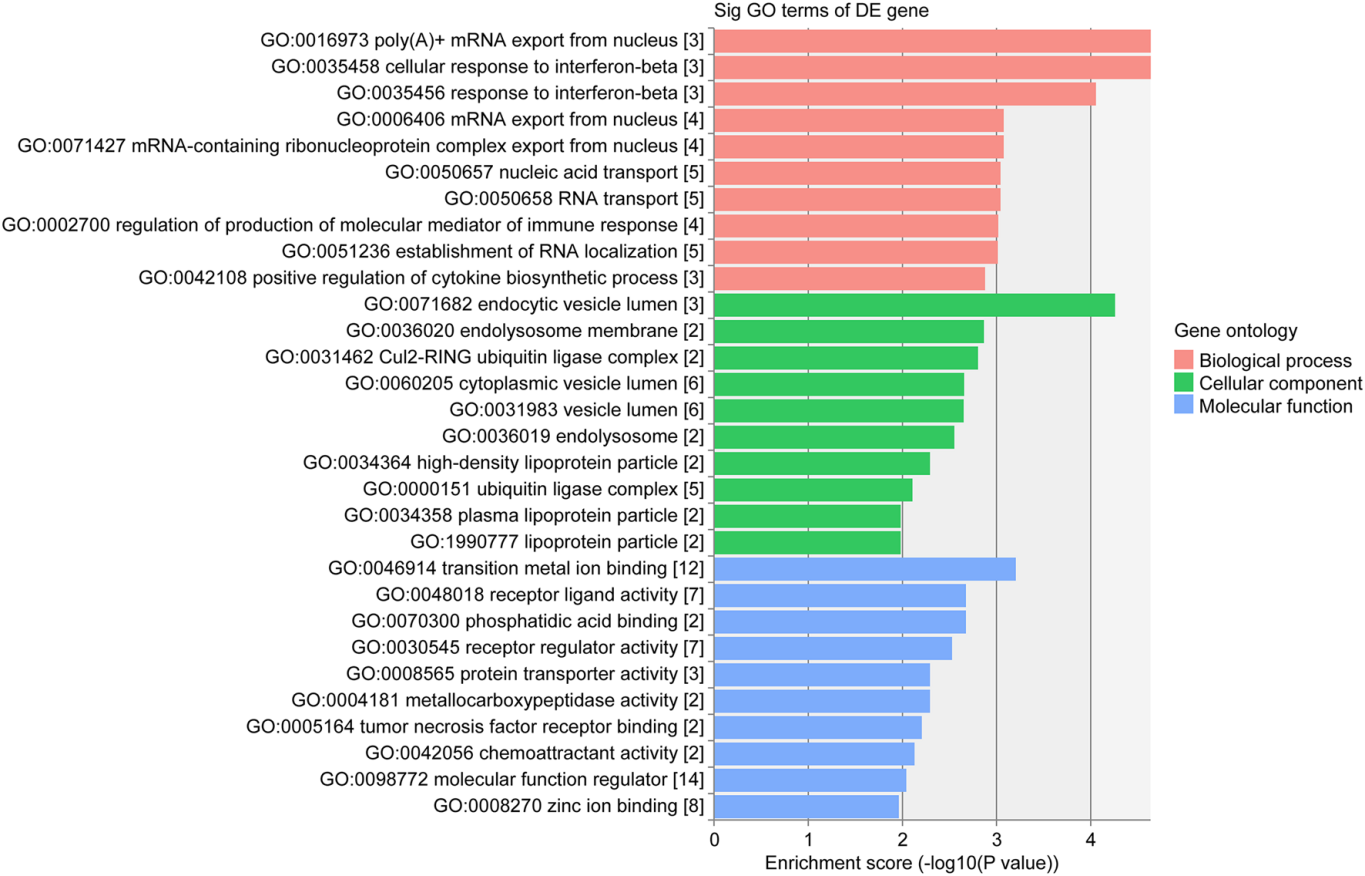
The top 10 significantly enriched terms of biological process by GO analysis of hsa_circRNA_000102 associated genes. GO analysis was divided into three parts: biological process, cell component and molecular function

**Fig. 5:**
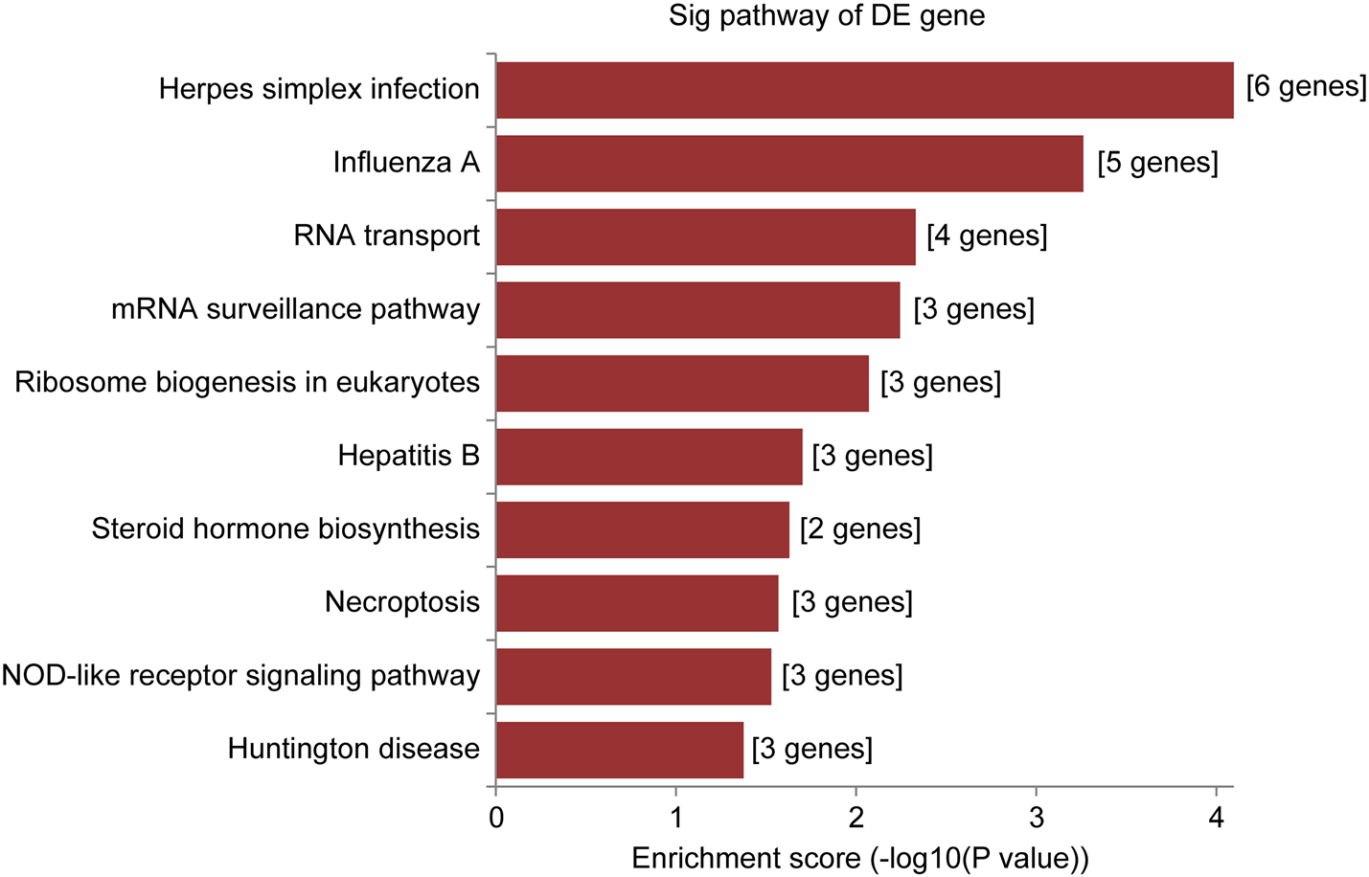
The top 10 significant enriched pathways by KEGG pathway analysis of hsa_circRNA_000102 associated genes

Predicted genes in the circRNA/microRNA/mRNA interaction network of hsa_circRNA_000102 were used for GO function analysis to annotate and speculate the potential functions of this circRNA. GO analysis was divided into three parts: biological process (BP), cell component (CC) and molecular function (MF) (Fig. 4). GO analysis of BP showed that hsa_circRNA_000102 was significantly linked to Poly(A)+ mRNA export from nucleus, cellular response to interferon-beta, mRNA-containing ribonucleoprotein complex export from nucleus, mRNA export from nucleus, RNA transport, nucleic acid transport, regulation of production of molecular mediator of immune response, establishment of RNA localization, and positive regulation of cytokine biosynthetic process. Significant GO cell component terms of hsa_circRNA_000102 showed that it was associated with endocytic vesicle lumen, endolysosome membrane, Cul2-RING ubiquitin ligase complex, endolysosome, high-density lipoprotein particle, lipoprotein particle, plasma lipoprotein particle, cytoplasmic vesicle lumen, vesicle lumen, and ubiquitin ligase complex. For molecular function, hsa_circRNA_000102 was associated with transition metal ion binding, receptor ligand activity, phosphatidic acid binding, receptor regulator activity, protein transporter activity, metallocarboxypeptidase activity, tumor necrosis factor receptor binding, chemoattractant activity, molecular function regulator, and Zinc ion binding. The top 10 KEGG pathways showed that hsa_circRNA_000102 associated genes might be involved in Herpes simplex infection, Influenza A, RNA transport, mRNA surveillance pathway, Ribosome biogenesis in eukaryotes, Hepatitis B, steroid hormone biosynthesis, necroptosis, NOD-like receptor signaling pathway and Huntington disease (Fig. 5).

## Discussion

In this study, we profiled the plasma exosomal circRNAs from patients with primary GD and healthy control subjects by human circRNA microarray. Before circRNA labeling and hybridization, RNAse R is used to efficiently digesting nearly all linear RNA species. Although RNAse R treatment may not exclude all entities, it is one of few ways at current available for enriching circRNAs. Our initial screening resulted in 15 differentially expressed circRNAs. One of the four selected candidate circRNAs, hsa_circRNA_000102, was verified to be significantly enriched in the plasma exosomes of patients with GD. Our comprehensive bioinformatics analyses, including prediction of a circRNA/microRNA/mRNA interaction network for hsa_circRNA_000102 and GO and KEGG pathway annotations of hsa_circRNA_000102 associated genes, revealed potential pathways correlated to the pathogenesis of GD.

hsa_circRNA_000102 is a circular intronic RNA (ciRNA), a new type of circRNA in human cells that is derived from introns discovered by Zhang *et al.* and others [19]. These RNAs escape debranching and depend on consensus RNA elements near the 5’ splice site and the branchpoint for proper processing [19]. Interestingly, ciRNAs partially accumulate to their sites of synthesis and colocalize with the elongation RNA polymerase II (Pol II). Moreover, the existence of ciRNAs might influence the transcription of their parent genes. For example, they are specifically associated with phosphorylated Pol II whereby a depletion of ciRNAs may lead to a significant reduction in the transcription of parent genes. Therefore, the higher level of hsa_circRNA_000102 in plasma exosomes of patients with GD, indicates the potentially elevated expression of its parent gene, *AKNAD1*, which encodes a protein with a domain found in an AT-hook-containing transcription factor. Though being not yet well-characterized, *AKNAD1* is considered to involve in biological processes of “microtubule-based movement” and “microtubule-based process”. Till now, the relation between gene *AKNAD1* and circRNA has not been discussed. Only one research has identified a significant copy number variation in gene *AKNAD1* which was validated to be associated with type-2 diabetes through stress of endoplasmic reticulum [20], suggesting a potential link of AKNAD1 and autoimmune diseases. However, more work on AKNAD1 in immune regulation and the underlying mechanisms on potential immune activation in GD is required to delineate the relationship between *AKNAD1* expression and the pathogenesis of GD.

The roles of circRNAs have been identified in tumor signaling pathways, invasion and migration, as well as tumor immunity [20]. Previous research proposed that mRNAs, transcribed pseudogenes, and circRNAs communicate with and co-regulate each other through competition for binding to the shared miRNAs [21]. Studies have also revealed that the activity of circRNA is decided by a series of factors, including the abundance of miRNAs, RNA editing, changes in the RNA 3′ untranslated region (UTR) and RNA binding proteins (RBPs) [22]. Therefore, interpreting the cross talks among circRNAs, miRNAs and mRNAs would provide novel insights into the mechanisms of gene regulation and immune cell interaction in Graves’ disease. We predicted the circRNAs/miRNAs/mRNAs interaction network of hsa_circRNA_000102, and then performed GO enrichment analysis and KEGG pathway analysis of the particular circRNA associated genes. Evidence demonstrated that hsa_circRNA_000102 is related to Herpes simplex infection, Influenza A signaling and interferon-beta signaling.

Both genetic and environmental factors have been suggested to contribute to the epidemiology of Graves’ disease [23]. It is not known whether the infection of a Herpes virus or Influenza A and Graves’ disease could co-exist simply by chance or the former could trigger the latter. A previous study has detected Herpes virus DNA in post-operative thyroid tissue specimens of patients with autoimmune thyroid disease, including Graves’ disease and Hashimoto thyroiditis [24]. The possible role of human Herpes viruses in the pathogenesis of autoimmune thyroid diseases is largely unknown and data from one study did not support their association [25]. However, another study indicated that Graves’ disease is linked to infectious mononucleosis due to primary Epstein-Barr virus infection in patients [26]. This suggests that both Herpes virus and Influenza A virus can employ similar mechanisms as Epstein–Barr virus to trigger immune activation.

We speculate that the association between viral infection and GD is due to the efficient T cell activation. Viral infection potentially increases the efficiency of T cell activation through fast attraction of dendritic cells from peripheral to secondary lymphoid tissues. Furthermore, continuous activation of innate immunity against viral infection might also profoundly strengthen autoimmune disorders by promoting autoantigen release without the need for specific activation of autoreactive T cells. As previously reported [27, 28], thyroid autoimmunity and dysfunctions have been observed over the course of IFN-beta therapy. A patient with multiple sclerosis received IFN-beta-1 therapy for 22 months and ultimately developed a severe Graves’ disease with persistently positive TRAb [27]. Since thyroidal side effects can be late to diagnose, patients undergoing long-term IFN-beta therapy should be closely monitored for thyroid hormones and antibodies throughout the treatment to prevent the possible occurrence of GD [27]. Taken together, although further studies are needed to clarify the relationship between infection of Herpes virus/Influenza A or IFN-beta signaling and GD, our study indicates that the impact of virus infection and IFN signaling should be taken into consideration during the prevention and/or treatment of GD.

The current investigation has several strengths and limitations. Its major strength is its novelty; to the best of our knowledge, the current study is the first one on profiling of plasma exosomal circRNAs in patients with GD. Our work also provides a feasible framework for searching and validating circular RNAs to better facilitate target identification and development of target-oriented therapeutics. The major limitation of the current study is its sample size of patients used for discovery and validation. Moreover, the patients recruited in this study were from a single institution and thus are subject to referral bias. Thirdly, there are some other limitations in this study. For examples, ribosomal RNAs were not removed, and the expression of AKNAD1 was not measured. In addition, no mock-treated samples were used during samples prep stage for generation of circRNA profile. Also, circRNA arrays we used is based on backsplice junctions pre-designed for those already existing in database. Therefore, this profile is very likely to lack novel circRNAs that could have been discovered using alternative methods to generate circRNA profiles.

## Conclusions

In this study, we performed human circRNA microarray in the plasma exosomes from patients with GD and healthy control subjects and verified hsa_circRNA_000102 as a differentially up-regulated plasma exosomal circRNA in GD. Our comprehensive bioinformatics analyses including construction of circRNA/microRNA/mRNA interaction network, GO annotation and KEGG pathway analysis, highlighted multiple pathways, particularly the virus infection and interferon-beta signaling, in mediating immune activation in Graves’ disease.

## Materials and methods

### Participants

Between August 2017 and October 2017, five newly diagnosed patients with GD (two males and three females) and five healthy control subjects were recruited by the Affiliated Yantai Yu-huangding Hospital of Qingdao University Medical, Shandong Province, China. The newly diagnosed GD patients had typical characteristics of hyperthyroidism: a palpable, soft, and diffuse goitre confirmed by ultrasound examination, increased thyroid radioiodine uptake, elevated serum free thyroxine (FT4), very low thyroid-stimulating hormone (TSH), and high levels of thyroid-stimulating antibody (TSAb). Age- and gender-matched healthy control subjects with normal thyroid function (TSH: 0.27–4.2 mIU/L), free of goitre and any family history of GD who meeting the exclusion criteria selected from healthy hospital staff were admitted into the study. Exclusion criteria included patients with liver disorder, renal disorder, clinical evidence of tumor, infection, any autoimmune diseases, history of intake of immunosuppressive drugs, such as mannan peptide, lentinan, interferon, corticosteroids, and cyclophosphamide, within 1 year of the study, pregnant, and lactating women. In the validation cohort, 20 patients with GD and 20 healthy control subjects were recruited adhering to the same inclusion and exclusion criteria. The research protocol was approved by Medical Ethics Committee of Affiliated Yantai Yu-huangding Hospital of Qingdao University Medical and the written informed consent was obtained from all participants.

### Laboratory test

The levels of serum TSH (11731459, Roche), FT4 (07976836, Roche), FT3 (free triiodothyronine) (06437206, Roche), TSAb(04388780190, Roche), thyroid peroxidase antibody (TPOAb) (06368590190, Roche), and thyroglobulin antibody (TgAb) (06368697, Roche) were measured via chemiluminescent enzyme immunoassay specific kits (Cobas 6000, Roche).

### Ultracentrifugation exosomes isolation and RNA extraction

Venous blood samples from subjects were collected in evacuated tubes with the anticoagulant Ethylenediamine tetraacetic acid dipotassium (K2EDTA) and stored at − 80 °C for exosome isolation. All blood samples were processed by centrifugation at 1000×g for 10 min. The plasma was immediately separated and transferred to a new RNase free tube. The liquid samples were centrifuged at 500×*g* for 5 min to eliminate cells. The supernatant was then transferred to a new polycarbonate tube and centrifuged at 2000×*g* for 10 min. Supernatants from the previously centrifuged samples were collected and centrifuged again at 10,000×*g* for 30 min to eliminate shed microvesicles (sMV, 200–1000 nm). The supernatants were then collected, filtered with 0.22 μm membrane filter (Merck Millipore), and centrifuged at 100,000×*g* for 2 h to obtain exosomes. For RNA isolation, an exosome pellet was washed once with 1×PBS and centrifuged at 100,000×*g* for 2 h. Exosomes were observed under a transmission electron microscope (TEM, TecnaiG2 Spirit 120 kV). Total RNA was extracted from exosomes using the TRIzol LS Reagent (10296028, Invitrogen Life Technologies, Carlsbad, CA, USA) according to the manufacturer’s protocol.

### Linear RNA digestion, circRNA labeling and hybridization

The exosomal RNAs extracted from the discovery cohort (five GD patients and five healthy control subjects) were used for microarray analysis, as previously described [29]. RNase R (RNR07250, Epicentre, Inc.) is used to digest nearly all linear RNA species and enrich circRNAs. The circRNA expression microarray slide (AS-S-CR-H-V2.0, Arraystar Human circRNA Arrays V2, 8 × 15 K; Arraystar Inc.) was used. Sample labeling and array hybridization were performed according to the manufacturer’s protocol. The hybridized arrays were scanned using the Agilent Scanner G2505C (Agilent Technologies, USA).

### CircRNA microarray data analysis

Raw data extraction of the scanned images was conducted using the Agilent Feature Extraction software (version 11.0.1.1). A series of data processing, including quantile normalization using a log2 ratio, was performed using the R software limma package. The circRNAs containing flags in “P” or “M” in at least five out of 10 samples (defined by GeneSpring software) were retained for further differential analyses. Differentially expressed circRNAs displaying statistically significant differences between the GD and healthy control groups were identified using fold change cutoff and through Volcano Plot filtering. The “fold change” (i.e. the ratio of the group averages) between the groups for each circRNA was computed. A *t*-test was employed to determine the statistical significance of the differences measured. CircRNAs denoted as significantly differentially expressed, were those having fold changes > 1.2 and *P*-values < 0.05 between the GD and healthy control exosome samples. The package gplots and function heatmap2 in R software were used for mapping. The distance metric was set as Euclidean distance.

### Real-time quantitative PCR (RT-qPCR) validation of microarray data

Total RNA from each exosome sample was quantified using the NanoDrop ND-1000 spectrophotometer. The reverse-transcription (RT) primers for circRNAs and β-actin were designed using Primer 5.0 and listed in Additional file 1: Table S1. Complementary DNA (cDNA) was synthesized using SuperScript™ III Reverse Transcriptase (18080-044, Invitrogen). CircRNAs transcripts were quantified using a SYBR Green Real-time PCR Master Mix (AS-MR-006-25, Arraystar, US). PCR was performed with the ViiA 7 Real-Time PCR System (Applied Biosystems). The thermal cycler conditions consisted of one cycle of denaturation at 95 °C for 10 min followed by 40 cycles of 95 °C for 10 s and 60  °C for 60 s. Melting curves were generated to ensure the specificity of the RT-qPCR. The expression of circRNAs was calculated by standard curve method using β-actin, 18srRNA and GAPDH as housekeeping genes. Then we compare the relationship among circRNAs and TRAb. All reactions were performed in triplicate.

### Annotation for circRNA/miRNA Interaction

RNA transcripts crosstalk by competing for common microRNAs and have been termed as competing endogenous RNAs (ceRNAs) [30]. Studies identified microRNA response elements (MREs) as the foundation of this interaction [31]. CeRNAs include pseudogene transcripts, long non-coding RNAs (lncRNAs), circRNAs and mRNAs, all of whose transcripts can compete for the same MRE to regulate mutually. Potential microRNA targets were identified with home-made miRNA target prediction software based on TargetScan and miRanda [32, 33]. The ceRNA network was constructed through merging the common targeted miRNAs. Three conditions must exist for the ceRNA network to occur, as previously described, which include: well defined relative concentrations of ceRNAs and their microRNAs; an appropriate number of microRNAs that ceRNA can “sponge”; and knowledge that not all of the MREs on ceRNAs are equal [30]. Therefore, only the ceRNA-pair relations passing these measures were accepted as filtered. The co-expression network was created according to the correlation analysis between normalized intensity of the differential circRNAs, miRNAs and mRNAs. Pearson correlation coefficients over 0.95 were retained for the construction using CYTOSCAPE software (The Cytoscape Consortium, San Diego, CA).

### Bioinformatics analysis

Gene ontology (GO) analysis was performed to explore the functional roles of circRNA-targeting genes in terms of biological processes, cellular components and molecular functions, as previously described [34]. Biological systems defined by the Kyoto Encyclopedia of Genes and Genomes (KEGG) (http://www.genome.jp/kegg/), was used to explore those pathways related to circRNA-targeting genes, as previously described [35].

### Statistics

SPSS software, version 17.0 (SPSS Inc.) was used for statistical analyses. Data are expressed as the mean ± SD (standard deviation) or median values with ranges, as indicated. An unpaired two-tailed Student’s *t*-test, or a nonparametric Mann–Whitney test was used for the comparisons between groups. Welch’s correction was added when the variances were significantly different between the two groups. Pearson’s correlation coefficient was used to analyze correlation between hsa_circRNA_000102 and TRAb. A two-sided *P* value < 0.05 was considered to be statistically significant.

## Supplementary information

**Additional file 1.** Additional figure and tables.

## Data Availability

All data generated or analysed during this study are included in this published article.

## Abbreviations

circRNA: Circulating RNA
GD: Graves’ disease
DEcRs: Differentially expressed circRNAs
KEGG: Kyoto Encyclopedia of Genes and Genomes
TSHR: Thyroid-stimulating hormone receptor
TSAb: Thyroid-stimulating antibody
ncRNAs: Noncoding RNAs
miRNAs: microRNAs
FT4: Free thyroxine
FT3: Free triiodothyronine
TRAb: TSH receptor antibodies
ciRNA: Circular intronic RNA
RBPs: RNA binding proteins
TPOAb: Thyroid peroxidase antibody
TgAb: Thyroglobulin antibody
ceRNAs: Endogenous RNAs
MREs: microRNA response elements
lncRNAs: Long non-coding RNAs
