# Supplementary material for: Microarray profiling and functional analysis of differentially expressed plasma exosomal circular RNAs in Graves’ disease
Source: Biol Res. 2020 Jul 29;53:32. doi: 10.1186/s40659-020-00299-y (PMC7388456; doi:10.1186/s40659-020-00299-y)
Supplement: Supplementary file 1 — Additional file 1. Additional figure and tables. [file 40659_2020_299_MOESM1_ESM.doc]

Microarray Profiling and Functional Analysis of Differentially Expressed Plasma Exosomal Circular RNAs in Graves' Disease

**Additional information**


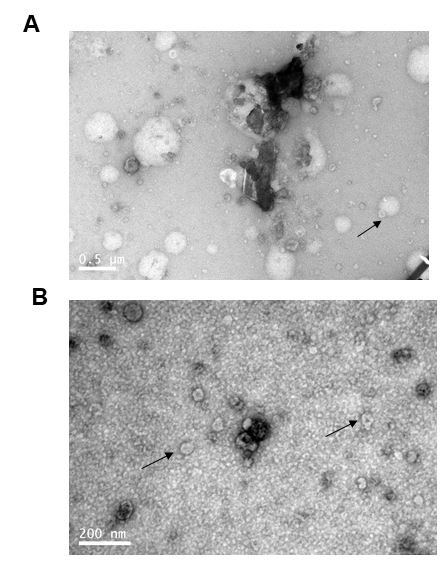


**Figure S1. Detection of plasma exosomes by transmission electron microscopy (TEM). (A-B)** Representative TEM images of plasma exosomes. Exosomes were processed with negative staining to enhance the view of membrane structures. The exosomes as indicated by arrows have diameters ranging from 30nm to 100nm. Scale bar, 500nm for A and 200nm for B.

Table S1. The DNA sequences of primers used in this study.

| **Gene name** | **Host genes** | **Primers (**5' - 3'**)** | **Annealing temperature (°C)** | **Product length (bp)** |
| --- | --- | --- | --- | --- |
| β-actin |  | F: GTGGCCGAGGACTTTGATTG  R: CCTGTAACAACGCATCTCATATT | 60 | 73 |
| GAPDH |  | F: CGGAGTCAACGGATTTGGTCGTAT  R: AGCCTTCTCCATGGTGGTGAAGAC | 60 | 307 |
| 18srRNA |  | F: GTAACCCGTTGAACCCCATT  R: CCATCCAATCGGTAGTAGCG | 60 | 151 |
| hsa_circRNA_000102 | AKNAD1 | F: AACGTATGAGGGTAGAAGAGAGA  R: TCAGGTCTATAATCAATTTCATCTC | 60 | 60 |
| hsa_circRNA_004939 | IL4R | F: CCTTGGCATCTCCCAATG  R: GTGTGGGTGCTACATTTCAGG | 60 | 140 |
| hsa_circRNA_072697 | PPWD1 | F: CAGATTTGTAACTGGAAAACTCAT  R: AGACTCTTTCAAACTCTAAGAGCTT | 60 | 70 |
| hsa_circRNA_102059 | MED1 | F: AACTTATGCACCCCTGTATGAACT  R: TTGTCATCCAGTAGGTCAGAAGG | 60 | 149 |

F, forward; R, reverse.

Table S2. The clinical characteristics of the participants in the discovery cohort.

| **Sample Number** | **Age**  **(years)** | **Gender** | **TSH (0.27-4.2) IU/ml** | **FT4**  **(12-22) pmol/L** | **FT3 (2.8-7.1) pmol/L** | **TRAb**  **(0-1.75) IU/L** | **TPOAb(0-34) IU/L** | **TgAb**  **(0-115) IU/ml** |
| --- | --- | --- | --- | --- | --- | --- | --- | --- |
| G1 | 45 | female | <0.005 | 73.03 | 39.92 | >40 | 262.5 | 982.1 |
| G2 | 52 | female | <0.005 | >100 | >50 | 18.44 | <5 | 374.2 |
| G3 | 31 | male | <0.005 | >100 | >50 | >40 | 277.8 | 677.1 |
| G4 | 31 | male | <0.005 | >100 | 49.69 | >40 | 57.79 | >4000 |
| G5 | 24 | male | <0.005 | >100 | 42.58 | 9.02 | 576.9 | 127.6 |
| N1 | 25 | female | 1.70 | 15.9 | 4.83 | 1.09 | <5 | 25.57 |
| N2 | 34 | male | 1.00 | 18.47 | 5.17 | <0.3 | 16.21 | 16.87 |
| N3 | 36 | male | 1.83 | 14.82 | 4.60 | <0.3 | 11.1 | 23.19 |
| N4 | 44 | female | 1.56 | 17.31 | 3.99 | 0.57 | <5 | <10 |
| N5 | 50 | male | 1.95 | 16.50 | 4.28 | 0.48 | 7 | 17.4 |

GD, Graves’ disease; TSH, thyrotropin (the normal range was 0.27-4.2mU/L）; FT4, free thyroxine (the detection range was 0.3-100 pmol/L, and the serum level of FT4 > 22 pmol/L was defined as elevated, while <12 pmol/L was defined as decreased); FT3, free triiodothyronine (the detection range was 0.4-50 pmol/L, and the serum level of FT3 > 7.1 pmol/L was defined as elevated, while <2.8 pmol/L was defined as decreased); TRAb, TSH receptor autoantibody (the detection range was 0.3-40 IU/ml, and TRAb > 1.75IU/L was defined as positive); TPOAb, thyroperoxidase antibody (the detection range was 5-600 IU/ml, and TPOAb > 34 IU/ml was defined as positive); TgAb, thyrogloblin antibody (the detection range was 10-4000 IU/ml, and TgAb >115 IU/ml was defined as positive).

Table S3. The clinical characteristics of patients with GD and healthy control subjects in the validation cohort.

|  | **Age**  **(years)** | **Male/**  **Female** | **TSH (0.27-4.2)**  **IU/ml^a^** | **FT4**  **(12-22)**  **pmol/L^a^** | **FT3**  **(2.8-7.1)**  **pmol/L^a^** | **TRAb**  **(0-1.75)**  **IU/L^a^** | **TPOAb**  **(0-34)**  **IU/L^a^** | **TgAb**  **(0-115)**  **IU/ml^b^** |
| --- | --- | --- | --- | --- | --- | --- | --- | --- |
| GD | 37.55±  12.18 | 10/10 | 0.0145±  0.0294 | 90.66±  10.71 | 38.2±  10.83 | 26.13±  12.9 | 302.8±  161.3 | 1887  (127.6-4000) |
| Healthy control | 37.15±  10.89 | 10/10 | 1.851±  0.8749* | 16.32±  2.06* | 4.85±  0.93* | 0.71±  0.46* | 12.18±  8.35* | 20.82  (10-114.1) * |

^a^Data are expressed as mean±standard deviation. ^b^Data presented as median (range). ^*^*P* < 0.05 vs. healthy controls.

GD, Graves’ disease; TSH, thyrotropin; FT4, free thyroxine; FT3, free triiodothyronine; TRAb, TSH receptor autoantibody; TPOAb, thyroperoxidase antibody; TgAb, thyrogloblin antibody.

Table S4. A list of upregulated and downregulated circRNAs in the plasma exosomes from the GD patients (data are in an independent Excel document).

| **G_vs_N 1 fold down regulated CircRNAs** | | | | | | | | | | | | | | | |
| --- | --- | --- | --- | --- | --- | --- | --- | --- | --- | --- | --- | --- | --- | --- | --- |
|  | **P-value and FDR** | | **Fold change and Regulation** | | **Annotations** | | | | | | | | | | |
| **probeID** | **P-value** | **FDR** | **FC (abs)** | **Regulation** | **circRNA** | **Alias** | **source** | **chrom** | **strand** | **txStart** | **txEnd** | **circRNA_type** | **best_transcript** | **GeneSymbol** | **Sequence** |
| ASCRP3000003 | 0.580949788 | 0.925542365 | 1.0505624 | down | hsa_circRNA_007418 | hsa_circ_0007418 | circBase | chr1 | + | 22816372 | 22818026 | exonic | NM_014870 | ZBTB40 | AAAAAACTAGAAATGTGTTCAGAAATTAAAGGTCCACAGAAGGAGGGCCTGTCCTCCCAA |
| ASCRP3000007 | 0.385809767 | 0.91119147 | 1.0299625 | down | hsa_circRNA_404446 | | 25070500 | chr1 | - | 19670851 | 19684057 | exonic | NM_004930 | CAPZB | AAAAACAAAGGATATCGTCAATGGGCTGAGGTATTTTGAAGGTGGCGTCTCATCTGTCTA |
| ASCRP3000015 | 0.805412918 | 0.974037816 | 1.0222463 | down | hsa_circRNA_103950 | hsa_circ_0001534 | circBase | chr5 | - | 137278563 | 137278905 | exonic | NM_016603 | FAM13B | AAAAACTGGCTCTGGATCTCCGATTGTCAAGTTCTCGAGCAGCTTCTATGGATCTCCATC |
| ASCRP3000016 | 0.654099408 | 0.933214341 | 1.0226509 | down | hsa_circRNA_007390 | hsa_circ_0007390 | circBase | chr15 | - | 73854236 | 73866136 | exonic | NM_012428 | NPTN | AAAAACTTGCGCCAGAGAAACACAAATTAAGTACTGCTTACAATCCGCTCCTGACATCAC |
| ASCRP3000023 | 0.5505425 | 0.919549314 | 1.0245409 | down | hsa_circRNA_404823 | | 25070500 | chr10 | - | 128973475 | 128975403 | exonic | NM_001039762 | FAM196A | AAAAAGGAGAGGCCCATCGGGAAGGAATTATTTTCCAGTACCACTCTCCATGGAATAAAA |
| ASCRP3000025 | 0.239742678 | 0.909237851 | 1.239421 | down | hsa_circRNA_102887 | hsa_circ_0057691 | circBase | chr2 | - | 200233327 | 200246543 | exonic | NM_015265 | SATB2 | AAAAAGTACAAGAAGATTAAAGGAATAATCAAGCTGGGAAGGTGGAACCCTCTCCCCCTC |
| ASCRP3000028 | 0.525929572 | 0.916971445 | 1.0227038 | down | hsa_circRNA_406825 | | 25070500 | chr6 | + | 109762831 | 109763131 | intronic | ENST00000258052 | SMPD2 | AAAAATAGCTGATCAGAGCTGGAAGACAAGGGAGGGGAAGAAGGCTGGGTGTGAGAAGCC |
| ASCRP3000035 | 0.265959451 | 0.909237851 | 1.0446032 | down | hsa_circRNA_061825 | hsa_circ_0061825 | circBase | chr21 | - | 43782390 | 43786644 | exonic | NM_003225 | TFF1 | AAAAATTAAAAGAGATCGATATTAAATCCCTGACTCGGGGTCGCCTTTGGAGCAGAGAGG |
| ASCRP3000048 | 0.255124582 | 0.909237851 | 1.0500357 | down | hsa_circRNA_101633 | hsa_circ_0036629 | circBase | chr15 | + | 85657103 | 85664245 | exonic | NM_002605 | PDE8A | AAAACATGGAGAGGTAATCAATATTATCAATGCTGCCCAGGAAAGTAGTCCCATGCCTGT |
| ASCRP3000064 | 0.851279607 | 0.978900086 | 1.0123916 | down | hsa_circRNA_087119 | hsa_circ_0087119 | circBase | chr9 | + | 68409997 | 68413434 | exonic | uc004aew.1 | AK308561 | AAAAGAGAGGCCGCCGCCTCTCTCTCCCTCTGGTTCTATTTCTCCATCCCTCTCTCCTTT |
| ASCRP3000069 | 0.288414078 | 0.909237851 | 1.2131102 | down | hsa_circRNA_000829 | hsa_circ_0000829 | circBase | chr18 | - | 12506475 | 12535600 | exonic | NM_020148 | SPIRE1 | AAAAGATACACCTTGCGAAAAGTGATGTTGTGTGCTGCTCATCTCCCTACTGAATCAGAT |
| ASCRP3000073 | 0.72754389 | 0.951172087 | 1.0357356 | down | hsa_circRNA_402810 | | 25242744 | chr3 | - | 31725125 | 31774903 | exonic | NM_017784 | OSBPL10 | AAAAGCAAGTTCATGGGCATGTCCGTGGGGGTCTCTATGATAGGGGAAGAAAACATCCTG |
| ASCRP3000079 | 0.854342772 | 0.978900086 | 1.0080667 | down | hsa_circRNA_406648 | | 25070500 | chr5 | + | 112870001 | 112874878 | exonic | NM_022828 | YTHDC2 | AAAAGGAAAAACAGCAAGGGTTTCTCCAAAGACACTTCTGACATTTTGTACTAATGGGGT |
| ASCRP3000081 | 0.12607244 | 0.909237851 | 1.0311466 | down | hsa_circRNA_103519 | hsa_circ_0068075 | circBase | chr3 | + | 179045348 | 179046139 | exonic | NM_016331 | ZNF639 | AAAAGGAAGACTCTACACCCTTCTCGTTATTCAGGCATTTTTTACTGTCTACAGAAACTT |
| ASCRP3000084 | 0.932239099 | 0.986846222 | 1.0028113 | down | hsa_circRNA_405561 | | 25070500 | chr17 | + | 29183972 | 29192818 | exonic | NM_024857 | ATAD5 | AAAAGTTACATAGGGTGTTGTTTGTGGCATTTGAAACCACCCTCTTGTCCTCTCTTAACT |
| ASCRP3000103 | 0.033123069 | 0.909237851 | 1.0586579 | down | hsa_circRNA_104496 | hsa_circ_0082440 | circBase | chr7 | + | 132990630 | 133002144 | exonic | NM_021807 | EXOC4 | AAAATTCCTTGATACCTCTCACTATTCTACTGCTGGAAGCTCAAGTGGTGTCAGCAGTTG |
| ASCRP3000106 | 0.325092563 | 0.909237851 | 1.0970159 | down | hsa_circRNA_026134 | hsa_circ_0026134 | circBase | chr12 | + | 49658864 | 49667113 | exonic | uc001rtt.1 | TUBA1C | AAAATTGAAGTTTCCATTTACTACTTCTCCCCCGGACTCCTTGGTAGTCTGTTAGTGGGA |
| ASCRP3000109 | 0.491723408 | 0.91119147 | 1.0615816 | down | hsa_circRNA_102771 | hsa_circ_0055377 | circBase | chr2 | + | 80772106 | 80783018 | exonic | NM_004389 | CTNNA2 | AAAATTGCTTTCTGAAACAGGTTGCCAATTTGGCCTGTTCCATCTCCAACAATGAAGAAG |
| ASCRP3000115 | 0.954413138 | 0.992077624 | 1.0021087 | down | hsa_circRNA_000980 | hsa_circ_0000980 | circBase | chr2 | + | 19538077 | 19538212 | antisense | ENST00000449124 | AC092594.1 | AAACAAGCATCATCCTCTTCTGAAGAAAGACAAGCATGAGGTGCTCCAACATTTTTCAGG |
| ASCRP3000116 | 0.329643428 | 0.909237851 | 1.0244564 | down | hsa_circRNA_102798 | hsa_circ_0009020 | circBase | chr2 | - | 110321942 | 110323436 | exonic | NM_144710 | SEPT10 | AAACAAGCCAGTCAGGGACAGTTGCCGTTTGCTGTTGTGGGAAGTATGGATGAGGTAAAA |
| ASCRP3000117 | 0.691298357 | 0.943480789 | 1.0320789 | down | hsa_circRNA_407330 | | 25070500 | chrX | - | 129799616 | 129813722 | exonic | NM_006375 | ENOX2 | AAACAAGCTGAGATCTCCCACCTCCTGCAACCCGAGAAAGACCACCAGGATGCAAAACAG |
| ASCRP3000120 | 0.047146676 | 0.909237851 | 1.0673282 | down | hsa_circRNA_101466 | hsa_circ_0005387 | circBase | chr15 | - | 28505922 | 28508315 | exonic | NM_004667 | HERC2 | AAACACATAGTGGGAATTGCCTGTGGGCCTGCCCAGGGCAAGTGTGGTCTTGGGGAGATG |
| ASCRP3000122 | 0.089028051 | 0.909237851 | 1.1279528 | down | hsa_circRNA_404317 | | 25242744 | chrX | - | 8553307 | 8565297 | exonic | NM_000216 | KAL1 | AAACACTTCCGTTCTTCCAAAGCCTCTCTTCCCCAAGAAGAGCTACGAATGCTTGACCAG |
| ASCRP3000124 | 0.067537581 | 0.909237851 | 1.0469404 | down | hsa_circRNA_007074 | hsa_circ_0007074 | circBase | chr1 | - | 54348615 | 54348949 | exonic | ENST00000465897 | YIPF1 | AAACAGACCATTACTTTCCAATTTGGCAATGCAGCCACTTCTCTGACAGCAAACCCAGAT |
| ASCRP3000129 | 0.599493821 | 0.929544944 | 1.0453582 | down | hsa_circRNA_407303 | | 25070500 | chrX | + | 77264598 | 77275895 | exonic | NM_000052 | ATP7A | AAACATCAAAGGTGAGGGGAATGACGTGTGCCTCCTGCGTACATAAAATAGAGTCTAGTC |
| ASCRP3000133 | 0.289320759 | 0.909237851 | 1.043185 | down | hsa_circRNA_003063 | hsa_circ_0003063 | circBase | chr18 | + | 8076452 | 8114825 | exonic | NM_002845 | PTPRM | AAACCAAAATAGACTGTGTCCAAGTGGCCACAAAAGTCCCAGGTGCTGTTCCCACTGAAT |
| ASCRP3000134 | 0.369842296 | 0.91119147 | 1.0462627 | down | hsa_circRNA_075881 | hsa_circ_0075881 | circBase | chr6 | + | 25605039 | 25606501 | exonic | NM_017640 | LRRC16A | AAACCAAGAACCGCCTCACGGCCTGAAGCTTGGGAATGATGCCGTATCCCAGGATTCTTC |
| ASCRP3000135 | 0.547046393 | 0.919549314 | 1.0299124 | down | hsa_circRNA_013959 | hsa_circ_0013959 | circBase | chr1 | - | 147131761 | 147131890 | exonic | NM_016361 | ACP6 | AAACCATATTCTCCTTACGACTCTCAATACCATGAGACCACCCTGAAGGTAGAGTGGAAC |
| ASCRP3000139 | 0.285412894 | 0.909237851 | 1.2180296 | down | hsa_circRNA_000968 | hsa_circ_0000968 | circBase | chr19 | + | 58774155 | 58774397 | intronic | ENST00000595981 | ZNF544 | AAACCCTATGAATGTGACCATTGCGAGAAAGCCTTTAGCCAACGGTCTCCCCTCATCATG |
| ASCRP3000153 | 0.054637356 | 0.909237851 | 1.0548013 | down | hsa_circRNA_100865 | hsa_circ_0002038 | circBase | chr11 | + | 68334481 | 68343511 | exonic | NM_001164160 | PPP6R3 | AAACTAACAAGAGGAACACGGTAGATCTAAAAAGTGTGATGAAGACCACATGGGGTGTGC |
| ASCRP3000162 | 0.834562892 | 0.978181218 | 1.0131069 | down | hsa_circRNA_400732 | | 25242744 | chr11 | + | 32987845 | 32995095 | exonic | NM_001076786 | QSER1 | AAACTCTAAGGACTTCTAAAACAACCACCAAATCTGCACAAAATTTGAACCTCCCGCTCC |
| ASCRP3000164 | 0.129517662 | 0.909237851 | 1.0832561 | down | hsa_circRNA_403821 | | 25242744 | chr7 | + | 65220766 | 65221561 | exonic | NR_003110 | CCT6P1 | AAACTGATACAAGGGCCTTCATCCCAGAATAATCACTGAAGGATTTGAAGCTGTGAAGGA |
| ASCRP3000173 | 0.000590952 | 0.870215632 | 1.1555018 | down | hsa_circRNA_046753 | hsa_circ_0046753 | circBase | chr18 | + | 3252272 | 3256234 | exonic | uc002kls.3 | MYL12A | AAACTTTATGATAAAATAGCTGCTGAGCGGTTTTGCAGACTACCATCTTCCCTGCAACTC |
| ASCRP3000175 | 0.252855904 | 0.909237851 | 1.0574213 | down | hsa_circRNA_045801 | hsa_circ_0045801 | circBase | chr17 | - | 74385612 | 74397235 | exonic | NM_022066 | UBE2O | AAACTTTTTTTTACTTGCAGGTCTCTTCTTCGATGATTCCTATGGCTTCTACCCAGGCCA |
| ASCRP3000190 | 0.269338308 | 0.909237851 | 1.0577133 | down | hsa_circRNA_102686 | hsa_circ_0053967 | circBase | chr2 | + | 36668400 | 36749456 | exonic | NM_016441 | CRIM1 | AAAGACCTGTCTTGAGAAAAGGCCAGTGTTGTCCCTACTGCATAGAAGAGAAGCCAGATT |
| ASCRP3000191 | 0.494249707 | 0.91167947 | 1.0193912 | down | hsa_circRNA_104090 | hsa_circ_0006109 | circBase | chr6 | + | 30618770 | 30619243 | exonic | NM_145029 | C6orf136 | AAAGACGAGCATTACCGCTTCCCAAGCTCTTCCTTCAGTCCCACGACTACAGTCTGTATT |
| ASCRP3000192 | 0.435836357 | 0.91119147 | 1.0358175 | down | hsa_circRNA_039783 | hsa_circ_0039783 | circBase | chr16 | + | 67070541 | 67100701 | exonic | NM_001755 | CBFB | AAAGACTGGATGGTATGGGCTGTCTGGAGTTTGATGAGGAGCGAGCCCAGGCTTTTGTGG |
| ASCRP3000195 | 0.551499703 | 0.919549314 | 1.031374 | down | hsa_circRNA_405972 | | 25070500 | chr2 | - | 150432224 | 150438785 | exonic | NM_015702 | MMADHC | AAAGAGAAGTGCTCTTAGAAAAGGTGCTTTGTAACAGAGCCAGACTGGTTTCCTATCTCC |
| ASCRP3000199 | 0.2774294 | 0.909237851 | 1.1171225 | down | hsa_circRNA_102890 | hsa_circ_0002867 | circBase | chr2 | - | 200245086 | 200298237 | exonic | NM_015265 | SATB2 | AAAGAGATGAACCAGAGCACATTAGCCAAAGAATGCCCTCTCTCCCAGGTTTGATGATTC |
| ASCRP3000205 | 0.331835346 | 0.909237851 | 1.046118 | down | hsa_circRNA_002876 | hsa_circ_0002876 | circBase | chr17 | + | 19213197 | 19232960 | exonic | NM_014964 | EPN2 | AAAGATGACTTTTCTGAATTTGACAACCTTCGGACTTCAAAAAAAACAGCCACCTCCCCG |
| ASCRP3000209 | 0.492776717 | 0.91119147 | 1.0281575 | down | hsa_circRNA_021832 | hsa_circ_0021832 | circBase | chr11 | + | 46342260 | 46342972 | exonic | uc001ncg.3 | CREB3L1 | AAAGCAAAACCAAAAAAAAAAAAAAAAAGATGCAGCATCAAGATCTGGGCCCCAACACCA |
| ASCRP3000217 | 0.987244956 | 0.998148217 | 1.0014324 | down | hsa_circRNA_100018 | hsa_circ_0009361 | circBase | chr1 | - | 1749275 | 1770677 | exonic | NM_002074 | GNB1 | AAAGCATGTGCAGATGCAACTCTCTCTCAGACAAATTTACATGTATTGGAGACCAGACCA |
| ASCRP3000230 | 0.443786317 | 0.91119147 | 1.018234 | down | hsa_circRNA_406107 | | 25070500 | chr20 | - | 35693826 | 35696589 | exonic | NM_002895 | RBL1 | AAAGGAGAATGCCTCCTGGACCTTTCAAGTTTTACTGATAATAGTTTAATACAATTTTTT |
| ASCRP3000236 | 0.751705429 | 0.957450341 | 1.0143315 | down | hsa_circRNA_102766 | hsa_circ_0055251 | circBase | chr2 | + | 74056531 | 74087278 | exonic | NM_006463 | STAMBP | AAAGGATTTCATCCACACAGCAAGGATCCACCTCTGTTCTGTATGCTGCATCCCCTTTTT |
| ASCRP3000271 | 0.216924926 | 0.909237851 | 1.1188031 | down | hsa_circRNA_020960 | hsa_circ_0020960 | circBase | chr11 | - | 5269501 | 5270820 | exonic | NM_000559 | HBG1 | AAATAATAAATCTATTCTGCTGAGAGATCACGCTCCTGGTTGTCTACCCATGGACCCAGA |
| ASCRP3000274 | 0.302941009 | 0.909237851 | 1.1010645 | down | hsa_circRNA_034125 | hsa_circ_0034125 | circBase | chr15 | + | 25312558 | 25351441 | exonic | uc001yxh.1 | SNURF-SNRPN | AAATAATTCTGCCCTCCCATTCTCAAGGGCCAGGGGGAATTCAAATATTGCAGTTGTCAT |
| ASCRP3000297 | 0.053658805 | 0.909237851 | 1.0617312 | down | hsa_circRNA_404782 | | 25070500 | chr10 | - | 79064144 | 79065150 | intronic | NM_001271518 | KCNMA1 | AAATCCTGGGGTTAGGGGCCCCTCTGCTATGGAGTTGAGACCCATGCCCTCAACCTCAAA |
| ASCRP3000298 | 0.642172439 | 0.929581513 | 1.0204143 | down | hsa_circRNA_405487 | | 25070500 | chr16 | - | 72318470 | 72342087 | sense overlapping | NR_126330 | LINC01572 | AAATCGACTCTGACTCAATCCTTGAGGTCTGGCCTCATCAGTGAAAAGAAGGACTGACTT |
| ASCRP3000301 | 0.945473581 | 0.990803144 | 1.0024049 | down | hsa_circRNA_003251 | hsa_circ_0003251 | circBase | chr12 | + | 1003727 | 1006847 | exonic | NM_014823 | WNK1 | AAATCTAGTCGAAGCAGTTCCTTGGGGAATAAAAGCCCCCAGCTTTCAGTTTCTCAAGTC |
| ASCRP3000305 | 0.54969981 | 0.919549314 | 1.0251858 | down | hsa_circRNA_406640 | | 25070500 | chr5 | + | 95966670 | 95970601 | intronic | ENST00000505143 | CAST | AAATCTTTGCATTCTGACTGTGCGATTTGGGAGATTCAGAAGAGCACTGGAGTCTCCACC |
| ASCRP3000308 | 0.990640451 | 0.998490743 | 1.0004041 | down | hsa_circRNA_002912 | hsa_circ_0002912 | circBase | chr3 | + | 44871462 | 44881948 | exonic | NM_020242 | KIF15 | AAATGAGGCAACTAGAACATGTGATGGATTCTGCTGCTGAGGATCCCCAGAAAGTTGTAG |
| ASCRP3000310 | 0.826609046 | 0.977459664 | 1.0168743 | down | hsa_circRNA_103730 | hsa_circ_0005654 | circBase | chr4 | - | 121675707 | 121732604 | exonic | NM_018699 | PRDM5 | AAATGATGGACTGAAGATGCACATTCGTACTCACACCAGGGAGATCCTAAGAAAAAGCTT |
| ASCRP3000316 | 0.572854077 | 0.922333971 | 1.0140494 | down | hsa_circRNA_016661 | hsa_circ_0016661 | circBase | chr1 | - | 225718255 | 225755116 | exonic | NM_018212 | ENAH | AAATGGCCCATCCCAAGAAGAATTGGAAATTCAAAGAAGTGAACAGAGTATCTGTCAGGC |
| ASCRP3000317 | 0.706884211 | 0.945024804 | 1.0238779 | down | hsa_circRNA_083127 | hsa_circ_0083127 | circBase | chr7 | - | 156619298 | 156626486 | exonic | NM_022458 | LMBR1 | AAATGGCTCCCTGATTCATGATGAACAAGAAGATGAAGATGCCATCGTCAACAGGATTTC |
| ASCRP3000327 | 0.781196764 | 0.966482321 | 1.0150506 | down | hsa_circRNA_103495 | hsa_circ_0001349 | circBase | chr3 | + | 152132729 | 152150709 | exonic | NM_021038 | MBNL1 | AAATTAATGCGAACAGACAGACTTGAGGGCCGTTGCTCCAGGGAGAACTGCAAATATCTT |
| ASCRP3000331 | 0.331374715 | 0.909237851 | 1.1122214 | down | hsa_circRNA_103492 | hsa_circ_0001346 | circBase | chr3 | + | 149563797 | 149639014 | exonic | NM_007282 | RNF13 | AAATTCAAGAAAGGTGATTTTACAACGAGATGCTGCTCTCCATAGGGATGCTCATGCTGT |
| ASCRP3000350 | 0.557982152 | 0.920978531 | 1.0331175 | down | hsa_circRNA_003657 | hsa_circ_0003657 | circBase | chr17 | + | 41576229 | 41577471 | exonic | NM_004941 | DHX8 | AACAAACACTGGGTTGACCCTCTGCCTGATGATGAGGACCTTGAGATTGAATTGGTTGAG |
| ASCRP3000358 | 0.27097764 | 0.909237851 | 1.0528143 | down | hsa_circRNA_028826 | hsa_circ_0028826 | circBase | chr12 | - | 120600873 | 120622046 | exonic | NM_006836 | GCN1L1 | AACAACTGGCCCAGGAAATGCTGATCATCTCCCACCACCCATCCTTAGAGATGCAGCCTC |
| ASCRP3000362 | 0.366102531 | 0.91119147 | 1.0530967 | down | hsa_circRNA_404177 | | 25242744 | chr9 | + | 71668055 | 71679951 | exonic | NM_000144 | FXN | AACAAGCAAATCTGGCTATCTTCTCCATCCAGCTCTCTAGATGAGACCACCTATGAAAGA |
| ASCRP3000374 | 0.167685505 | 0.909237851 | 1.1463896 | down | hsa_circRNA_040831 | hsa_circ_0040831 | circBase | chr16 | + | 88694029 | 88695262 | exonic | NM_144604 | ZC3H18 | AACACATCCCCAGACCGAGGTCCCTGAGCGTGAGCAGCGTCTCCTCAGTGTCCAGTGCTA |
| ASCRP3000388 | 0.063838639 | 0.909237851 | 1.0464944 | down | hsa_circRNA_104450 | hsa_circ_0002747 | circBase | chr7 | + | 102994367 | 102996240 | exonic | NM_002803 | PSMC2 | AACAGCCTTTACAGGTTGCCAGCTCTGGATGAGGGGGATATTGCCTTGTTGAAAACTTAT |
| ASCRP3000392 | 0.237031303 | 0.909237851 | 1.0399202 | down | hsa_circRNA_002084 | hsa_circ_0002084 | circBase | chr19 | + | 49416267 | 49416821 | exonic | NM_006184 | NUCB1 | AACAGGTTTAACCCCAAGACCTTCTTCATACTGCATGGCCACCCGGGACCTTGCCCAGTA |
| ASCRP3000406 | 0.05053895 | 0.909237851 | 1.0933312 | down | hsa_circRNA_092392 | hsa_circ_0000368 | circBase | chr11 | + | 128628009 | 128638167 | exonic | NM_002017 | FLI1 | AACATGACCACCAACGAGAGGAGAGTCATCGTCCCCGCAGGAGGCTCTGTCGGTGGTGAG |
| ASCRP3000433 | 0.537365891 | 0.919549314 | 1.1114825 | down | hsa_circRNA_401976 | | 25242744 | chr18 | + | 55398645 | 55399255 | exonic | uc002lgv.1 | LOC100505549 | AACCATTCAGTACTGATTAAATGCTTGGAACTTGTCAGATATGCTCTCCCAGCCCTTCTT |
| ASCRP3000438 | 0.252192688 | 0.909237851 | 1.0504179 | down | hsa_circRNA_043650 | hsa_circ_0043650 | circBase | chr17 | + | 39845126 | 39847898 | exonic | NM_005801 | EIF1 | AACCCTTATTTAAATAAAACTTGTTTTCAGAAATACCGCCCCTCTGCCCCAGTCACTGAG |
| ASCRP3000440 | 0.643671101 | 0.929872547 | 1.0590506 | down | hsa_circRNA_100808 | hsa_circ_0003110 | circBase | chr11 | - | 46529740 | 46534363 | exonic | NM_017749 | AMBRA1 | AACCGACTTGGAATTTGAGGACTTTGAGAGGCTGCTGGAATCTTCCCTCATTTCATTATC |
| ASCRP3000441 | 0.438991075 | 0.91119147 | 1.0265976 | down | hsa_circRNA_101904 | hsa_circ_0040827 | circBase | chr16 | + | 88066714 | 88071617 | exonic | NM_017869 | BANP | AACCTCCAGATCCATCACGTGGGGCAGGACGGTCAGAGCCGATGATGAGCACCCCACCTC |
| ASCRP3000450 | 0.153626193 | 0.909237851 | 1.0492721 | down | hsa_circRNA_100076 | hsa_circ_0010430 | circBase | chr1 | - | 21024871 | 21031392 | exonic | NM_020816 | KIF17 | AACCTGTGATCCAGCATGACGTGGAGGCCGAGAAGCAGCTGATCCGGGAGATGAGCGGGG |
| ASCRP3000464 | 0.549430598 | 0.919549314 | 1.0174365 | down | hsa_circRNA_004087 | hsa_circ_0004087 | circBase | chr16 | - | 80718434 | 80719026 | exonic | NM_152342 | CDYL2 | AACGGGCTCGGTTGAAAGGATTGTAGACAAGAGGAAGAACAAGAAAGGAAAATGGGAGTA |
| ASCRP3000469 | 0.86447454 | 0.979863126 | 1.0082431 | down | hsa_circRNA_035861 | hsa_circ_0035861 | circBase | chr15 | + | 65223019 | 65226434 | exonic | NM_182703 | ANKDD1A | AACGTGGTTGATCATGAGGGGAACACTGCCCTTCATCTGGCTGCTGGTCGGGGCCATATG |
| ASCRP3000470 | 0.291175523 | 0.909237851 | 1.0410773 | down | hsa_circRNA_103990 | hsa_circ_0003967 | circBase | chr5 | + | 151169883 | 151170623 | exonic | NM_005754 | G3BP1 | AACGTTTGTCCTTGCTCCTGAGATTTTATGGAAAGAACTCTTCTTATGTCCATGGGGGAT |
| ASCRP3000473 | 0.948234621 | 0.992077624 | 1.0026336 | down | hsa_circRNA_005593 | hsa_circ_0005593 | circBase | chr5 | + | 52899281 | 52900725 | exonic | ENST00000502423 | NDUFS4 | AACTAATCCTGAGGACTTCATCCGGTCACCATCTACCTATTCTTCAAATGGTCGTTGAGG |
| ASCRP3000474 | 0.231678487 | 0.909237851 | 1.0533298 | down | hsa_circRNA_061343 | hsa_circ_0061343 | circBase | chr21 | - | 27347382 | 27369731 | exonic | NM_000484 | APP | AACTACATCACCGCTCTGCAGGCTGTTCCTCCTCGGTGTCCCAAAGTTTACTCAAGACTA |
| ASCRP3000481 | 0.889642246 | 0.979863126 | 1.0037046 | down | hsa_circRNA_103563 | hsa_circ_0008583 | circBase | chr3 | - | 196817782 | 196846401 | exonic | NM_004087 | DLG1 | AACTAGCCAGAAGCGATCCCTCTATGTCAGCTTCTTCTCAGCCTGTTGATAACCATGTTA |
| ASCRP3000484 | 0.146265291 | 0.909237851 | 1.0598855 | down | hsa_circRNA_406886 | | 25070500 | chr6 | + | 157469757 | 157471987 | sense overlapping | NM_017519 | ARID1B | AACTATGGCAACTCTCAGGCTCCCATGGTGCACCAGCCTGACCAGTACGGGTAACTACTC |
| ASCRP3000485 | 0.8202942 | 0.976440085 | 1.0107409 | down | hsa_circRNA_002837 | hsa_circ_0002837 | circBase | chr19 | - | 41847787 | 41848152 | exonic | NM_000660 | TGFB1 | AACTATTGCTTCAGGGGAAATTGAGGGCTTTCGCCTTAGCGCCCACTGCTCCTGTGACAG |
| ASCRP3000507 | 0.711917741 | 0.946024376 | 1.0147417 | down | hsa_circRNA_000224 | hsa_circ_0000224 | circBase | chr10 | + | 20436712 | 20466338 | exonic | NM_032812 | PLXDC2 | AACTTCAAAGGCACAGCACTTGTGGTCCAGTGGGACCATGTACATCTCCAGGATAATTAT |
| ASCRP3000515 | 0.677775879 | 0.94025363 | 1.058409 | down | hsa_circRNA_102927 | hsa_circ_0058493 | circBase | chr2 | + | 227729319 | 227732034 | exonic | NM_032276 | RHBDD1 | AACTTGTGGCTATTCATTTATTCTCACCAGGGTTCAGCCGTCTGTATATCTCCCCAGATA |
| ASCRP3000517 | 0.716782692 | 0.948648358 | 1.0224075 | down | hsa_circRNA_001526 | hsa_circ_0001526 | circBase | chr5 | - | 131822730 | 131823714 | sense overlapping | NM_002198 | IRF1 | AACTTTCGCTGTGCCATGAACTCCCTGCCAGATATCGAGGAGGAGATGATCTTCCAGATC |
| ASCRP3000521 | 0.924355089 | 0.983668077 | 1.0036846 | down | hsa_circRNA_406730 | | 25070500 | chr6 | - | 1099947 | 1101761 | sense overlapping | NR_027115 | LOC285768 | AAGAAAAATGAAAGGGTCCTGCCTACCCATCTGAGGACATGTCTGCTCATCACCCATGTG |
| ASCRP3000522 | 0.465231717 | 0.91119147 | 1.0378037 | down | hsa_circRNA_405841 | | 25070500 | chr2 | + | 28210859 | 28218323 | sense overlapping | NM_004899 | BRE | AAGAAAAATGTTTACAGGGTCTGGCCCCCGCTTCATAAACAGGGGGATATCATTTTCAAT |
| ASCRP3000537 | 0.497348939 | 0.913224439 | 1.0183492 | down | hsa_circRNA_001937 | hsa_circ_0000700 | circBase | chr16 | + | 53155459 | 53155541 | intronic | ENST00000447540 | CHD9 | AAGAACAGCTCTCTGGCTGAACATATCCCTTGGATACTCTAAGACCTGACCCTGATTAAG |
| ASCRP3000538 | 0.218052506 | 0.909237851 | 1.0550217 | down | hsa_circRNA_030431 | hsa_circ_0030431 | circBase | chr13 | - | 75936161 | 75936743 | exonic | NM_014832 | TBC1D4 | AAGAACAGGACCATGCTCTTCCAGGTTCCTGATGTTATTAGCAGCATAAGGCAATTATCT |
| ASCRP3000544 | 0.446454787 | 0.91119147 | 1.0753561 | down | hsa_circRNA_101034 | hsa_circ_0007723 | circBase | chr12 | + | 28458581 | 28460682 | exonic | NM_018318 | CCDC91 | AAGAACTTCAGTATCTGGAGTCCATCTTTCACCATCTTCTCCTGAGATTGTACTGGACCG |
| ASCRP3000554 | 0.510830957 | 0.916805276 | 1.0317379 | down | hsa_circRNA_406091 | | 25070500 | chr20 | - | 25000645 | 25004277 | exonic | NM_032501 | ACSS1 | AAGAAGTATGATCGCTCCTCCCTGCGGACCCTGGGGTCAGCCAAGTGCAAGGTGGTTATC |
| ASCRP3000558 | 0.099468764 | 0.909237851 | 1.0696589 | down | hsa_circRNA_101630 | hsa_circ_0036627 | circBase | chr15 | + | 85656607 | 85669605 | exonic | NM_002605 | PDE8A | AAGAATATTTTTCTCAGTTTCCAGCCAGAGACGACACTCTTCCATGGCCCGGATACATTC |
| ASCRP3000559 | 0.490776914 | 0.91119147 | 1.0316946 | down | hsa_circRNA_007258 | hsa_circ_0007258 | circBase | chr16 | + | 29847024 | 29848279 | exonic | NM_005115 | MVP | AAGAATCAGCTGGGGCAGAAGCGCGTGGTCAAGGGGAAGAATGGCTGGTCACCACAGTAG |
| ASCRP3000572 | 0.082081564 | 0.909237851 | 1.0777186 | down | hsa_circRNA_103179 | hsa_circ_0062593 | circBase | chr22 | + | 24509532 | 24530382 | exonic | NM_012295 | CABIN1 | AAGACCAATTTCTTCAACACCGAAGCCAGGACAGCACAGCCGTAGCACTCTCAGACTCTA |
| ASCRP3000575 | 0.215042324 | 0.909237851 | 1.2353716 | down | hsa_circRNA_100445 | hsa_circ_0016404 | circBase | chr1 | + | 212977661 | 212977993 | exonic | NM_001042552 | TATDN3 | AAGACCTACCATCAACCTTTTACAAGAGCAAGGTTGGACTAGATTTCTCCCCCAGATTTG |
| ASCRP3000577 | 0.473229035 | 0.91119147 | 1.0301539 | down | hsa_circRNA_405953 | | 25070500 | chr2 | + | 122797766 | 122800683 | intergenic |  |  | AAGACTAATTACCCAACTGCCTCCGACTGACAAGATTGAGCATTTACAGAAGCAGGAATC |
| ASCRP3000579 | 0.552443175 | 0.919549314 | 1.0483934 | down | hsa_circRNA_100213 | hsa_circ_0000069 | circBase | chr1 | - | 47745912 | 47748131 | exonic | NM_003035 | STIL | AAGACTACTTCAGGCACAGGTCTTCCCAAAAGTTATCTTCTGGGAAGATGCCAATACATG |
| ASCRP3000583 | 0.358044398 | 0.91119147 | 1.0567659 | down | hsa_circRNA_092470 | hsa_circ_0000941 | circBase | chr19 | + | 45781180 | 45781873 | exonic | NM_031417 | MARK4 | AAGAGAATTGGAGCTGCGGGAGCGAGTACTCAGAGGGAAGTACCGGGTCCCTTTCTACAT |
| ASCRP3000587 | 0.332941293 | 0.909237851 | 1.0373429 | down | hsa_circRNA_007672 | hsa_circ_0007672 | circBase | chr7 | + | 65705311 | 65706257 | exonic | NM_003596 | TPST1 | AAGAGATGATTGGGAAAGCTGGGGGAGTGTCTCTGTCAAAATGTTGGTTATCTTTCTGAA |
| ASCRP3000588 | 0.838285451 | 0.978181218 | 1.0084754 | down | hsa_circRNA_407261 | | 25070500 | chrX | - | 2828699 | 2856298 | sense overlapping | NM_000047 | ARSE | AAGAGATGGACACAGCCAGTTAGGGGGATGGAACGGAATTTACAAAGGTGGGAAGGGCAT |
| ASCRP3000593 | 0.323950743 | 0.909237851 | 1.1020906 | down | hsa_circRNA_058188 | hsa_circ_0058188 | circBase | chr2 | + | 217329319 | 217332769 | exonic | NM_014140 | SMARCAL1 | AAGAGCTTGAGAGAAAGAAACAGCAGCAGAAAGATGCCCTCATTCTCTTCTTCAACAGAA |
| ASCRP3000607 | 0.113951179 | 0.909237851 | 1.1464274 | down | hsa_circRNA_401965 | | 25242744 | chr18 | + | 42529845 | 42533305 | exonic | NM_015559 | SETBP1 | AAGATGAACCGCAAGGAGAGAAGTTCTTATGACTCCTCCATGTCTCCAGGCTTACGAGAG |
| ASCRP3000608 | 0.655565707 | 0.933214341 | 1.0404784 | down | hsa_circRNA_402819 | | 25242744 | chr3 | - | 33114035 | 33118729 | exonic | uc011axk.1 | GLB1 | AAGATGAAGATGGCTGGGCTGAACGCCATCCAGACTCTCGATTCCTTCCTTGGGCTTTTC |
| ASCRP3000614 | 0.524936785 | 0.916971445 | 1.0395226 | down | hsa_circRNA_066970 | hsa_circ_0066970 | circBase | chr3 | + | 121563299 | 121576003 | exonic | NM_018456 | EAF2 | AAGATGTCCCCAGCATCTCCAATAGATGATATCGAAAGAGATGACTTCAAACCTGCTTCT |
| ASCRP3000616 | 0.671602053 | 0.938080316 | 1.006893 | down | hsa_circRNA_401876 | | 25242744 | chr17 | + | 72285732 | 72287272 | exonic | NM_023036 | DNAI2 | AAGATTCCCACGTACTCCTGGGTGGCTGCTACAATGGACAGATAGGGACCCCCAGGAAAT |
| ASCRP3000617 | 0.460516138 | 0.91119147 | 1.0356316 | down | hsa_circRNA_404026 | | 25242744 | chr8 | + | 42401611 | 42403923 | exonic | NM_138436 | SMIM19 | AAGATTCGTTTAAGACAACAACTGGAAATGTATTCCATTTCCCCATGGCTGGGGGTTATG |
| ASCRP3000627 | 0.712217855 | 0.946024376 | 1.026679 | down | hsa_circRNA_011588 | hsa_circ_0011588 | circBase | chr1 | - | 36211051 | 36212593 | exonic | NM_022111 | CLSPN | AAGCACTTGCTCTTTGCTCAGGCTCTTTTCCCACAGACAAGTTCTTAAATGTTAGAAACC |
| ASCRP3000628 | 0.629519039 | 0.929544944 | 1.0136713 | down | hsa_circRNA_000104 | hsa_circ_0000266 | circBase | chr10 | - | 126336614 | 126337010 | intronic | ENST00000337318 | FAM53B | AAGCAGATTTTCAGCATCCTAGGAAGAACCAAGAATGCAGAAGTCTGGGGGTGGTGGAGA |
| ASCRP3000635 | 0.863578518 | 0.979863126 | 1.0218209 | down | hsa_circRNA_000624 | hsa_circ_0000751 | circBase | chr17 | - | 27620262 | 27620746 | intronic | ENST00000225388 | NUFIP2 | AAGCCCACCTGTGGCCTTGGGGCTCCCCGTCTCGATGGGGGTGGGGCTGGATCCGAAAAG |
| ASCRP3000642 | 0.27113277 | 0.909237851 | 1.1159307 | down | hsa_circRNA_049851 | hsa_circ_0049851 | circBase | chr19 | + | 15939756 | 15946230 | exonic | TCONS_00026750 | XLOC_012981 | AAGCTAATATGCCTGATTGACATTCTTCTGGACAATGAGTCCCATCATCTCTCCACCATG |
| ASCRP3000643 | 0.788390692 | 0.967199246 | 1.0128725 | down | hsa_circRNA_103700 | hsa_circ_0070421 | circBase | chr4 | + | 89396991 | 89400658 | exonic | NM_016323 | HERC5 | AAGCTCTCCTAGAAATGTTGAAGAAGCTGCACAGGATAACCACCTGCCTCAAAGATAATC |
| ASCRP3000644 | 0.037871021 | 0.909237851 | 1.0502819 | down | hsa_circRNA_103773 | hsa_circ_0003437 | circBase | chr4 | + | 184114751 | 184130178 | exonic | NM_024949 | WWC2 | AAGCTGAGATCTCCACTACAAGATTAAGGGTTAACGAAGCCCTTGTCATTTGCTGATTGT |
| ASCRP3000647 | 0.38533627 | 0.91119147 | 1.078123 | down | hsa_circRNA_104396 | hsa_circ_0002690 | circBase | chr7 | + | 73510951 | 73511519 | exonic | NM_002314 | LIMK1 | AAGCTGTACTGGTGTTGTGACTGCAGTGCCTCCCTGTCGCACCAGTACTATGAGAAGGAT |
| ASCRP3000649 | 0.168925567 | 0.909237851 | 1.0361916 | down | hsa_circRNA_104527 | hsa_circ_0083054 | circBase | chr7 | - | 152007050 | 152009031 | exonic | NM_170606 | KMT2C | AAGCTGTTGTCTCAGGGAGCACAGAAAGAACGATCTCCTCAGCAGAATATAGTATCTTGT |
| ASCRP3000651 | 0.141228635 | 0.909237851 | 1.0723926 | down | hsa_circRNA_100800 | hsa_circ_0021773 | circBase | chr11 | + | 43421437 | 43429127 | exonic | NM_018259 | TTC17 | AAGCTTTGGCCATCAATAGCTCTGAGATCTCTCCAGCGAACACTGAATGAGTTAAAAGAG |
| ASCRP3000657 | 0.889282167 | 0.979863126 | 1.0032122 | down | hsa_circRNA_003850 | hsa_circ_0003850 | circBase | chr4 | + | 3088665 | 3117990 | exonic | NM_002111 | HTT | AAGGAAAGAAATGGAAGTCTCTCCTTCTGCAGAGCAGCTTGTCCAGAAAGAAAGAACTTT |
| ASCRP3000658 | 0.460530904 | 0.91119147 | 1.0231121 | down | hsa_circRNA_402621 | | 25242744 | chr20 | - | 49558567 | 49571822 | exonic | NM_003859 | DPM1 | AAGGAAATGGAGGTGTATATGGCTGGGATTTGAAAAGAAAAATAATCAGTGGAATCAACT |
| ASCRP3000661 | 0.695328471 | 0.943480789 | 1.0290885 | down | hsa_circRNA_008289 | hsa_circ_0008289 | circBase | chr6 | + | 155448623 | 155451551 | exonic | NM_012454 | TIAM2 | AAGGAAGAAAAGGAAACTCCAGGCTCACTTCATGGACTCACTTTGCGTGCTTGTTAAATG |
| ASCRP3000665 | 0.699194619 | 0.944102043 | 1.0200562 | down | hsa_circRNA_007877 | hsa_circ_0007877 | circBase | chr1 | - | 21573713 | 21585332 | exonic | NM_001397 | ECE1 | AAGGAATACCTTGAGCAGATCTCCACTCTCATCAACACCACCGACAGATGCTCGGGGGCT |
| ASCRP3000677 | 0.797797868 | 0.971336981 | 1.0074194 | down | hsa_circRNA_019061 | hsa_circ_0019061 | circBase | chr10 | + | 90661412 | 90682193 | exonic | NM_020799 | STAMBPL1 | AAGGAGCCCAGGCTGTTCAGTATGAAGTGATTGAGAAGAAACAGTGAACATCCTCATTTC |
| ASCRP3000680 | 0.406105299 | 0.91119147 | 1.0220332 | down | hsa_circRNA_103407 | hsa_circ_0007761 | circBase | chr3 | + | 63898263 | 63898901 | exonic | NM_000333 | ATXN7 | AAGGAGTTTGGGAAAAACCGCGAAGTCATGGGGCTCTGTCGGGAAGGAGCGGAAAGAATG |
| ASCRP3000684 | 0.523058787 | 0.916971445 | 1.0710903 | down | hsa_circRNA_101275 | hsa_circ_0030428 | circBase | chr13 | - | 75915260 | 75915723 | exonic | NM_014832 | TBC1D4 | AAGGCCCTTCTGTCTCTACCCACCAAGAGCCAAGCTGGTGATACAGAGGCATCTCTCATC |
| ASCRP3000689 | 0.96784724 | 0.995499551 | 1.0021238 | down | hsa_circRNA_102447 | hsa_circ_0005360 | circBase | chr19 | + | 11221327 | 11222315 | exonic | NM_000527 | LDLR | AAGGCTGTGGGGACCAACGAATGCTTGGACAACAACGGCGGCTGTTCCCACGTCTGCAAT |
| ASCRP3000709 | 0.929472362 | 0.985609588 | 1.0024969 | down | hsa_circRNA_001621 | hsa_circ_0001621 | circBase | chr6 | + | 90556280 | 90566918 | exonic | NM_012115 | CASP8AP2 | AAGTAATCTTCACCAAAGGTGGTGACTCTCAAAAGGAAATAGGATCATGGCAGCAGATGA |
| ASCRP3000712 | 0.571588493 | 0.922333971 | 1.0303507 | down | hsa_circRNA_100172 | hsa_circ_0011615 | circBase | chr1 | + | 36354027 | 36360003 | exonic | NM_012199 | AGO1 | AAGTACCGCGTGTGTAATGTTACCCGTCGCCCTGCTAGCCATCAGACCTGCGGGCGCTTA |
| ASCRP3000717 | 0.680909463 | 0.941064238 | 1.0250659 | down | hsa_circRNA_000459 | hsa_circ_0000459 | circBase | chr12 | + | 124091978 | 124101150 | exonic | NM_020936 | DDX55 | AAGTCAACTGGGTTTTGCAGTATGACCCTCCCAGCAATGCAAGGTTGGAGCCATAATCAT |
| ASCRP3000720 | 0.079484109 | 0.909237851 | 1.0404147 | down | hsa_circRNA_100749 | hsa_circ_0020927 | circBase | chr11 | + | 3988781 | 4045217 | exonic | NM_003156 | STIM1 | AAGTCATCAGAAGAGTTTTGCCGAATTGACAAGCCCCTGTGTCACAGTGAGGATGAGAAA |
| ASCRP3000732 | 0.907542705 | 0.982379023 | 1.0033504 | down | hsa_circRNA_086441 | hsa_circ_0086441 | circBase | chr9 | - | 19063005 | 19063578 | exonic | NM_017645 | HAUS6 | AAGTGATCCATTTCAAAAAGAGCAAGATCATCTGGTAGAAGAGCCCTGCCTCTTTCTTGT |
| ASCRP3000746 | 0.018260135 | 0.909237851 | 1.0813888 | down | hsa_circRNA_100828 | hsa_circ_0022308 | circBase | chr11 | - | 61096834 | 61097546 | exonic | NM_001923 | DDB1 | AAGTTCCTATATGGTTGCCAAGCACCTACTATTTGCTTTGTCTACCAGGGGGAGAGCAAG |
| ASCRP3000763 | 0.608097845 | 0.929544944 | 1.0191293 | down | hsa_circRNA_039830 | hsa_circ_0039830 | circBase | chr16 | - | 67471916 | 67477081 | exonic | NM_004691 | ATP6V0D1 | AATAAAACTGCCCTCTCTAAGGCCAACTGTCCGGCTTTTTTCCAGGACTGCATTTCAGAG |
| ASCRP3000768 | 0.057488235 | 0.909237851 | 1.0594018 | down | hsa_circRNA_043366 | hsa_circ_0043366 | circBase | chr17 | + | 36909402 | 36920478 | exonic | uc002hqr.3 | PSMB3 | AATAAAATAGCCTGTCTTTCATCTATTATGTCCTATAACGGAGGGGCCGTCATGGCCATG |
| ASCRP3000770 | 0.525691479 | 0.916971445 | 1.0421701 | down | hsa_circRNA_023617 | hsa_circ_0023617 | circBase | chr11 | + | 75116657 | 75116733 | intronic | ENST00000527446 | RPS3 | AATAAAATTTTGTACAAAGACAGGTCTCCTTGGCAGCTGTATTCTGGAGTCTGGATGTTG |
| ASCRP3000778 | 0.834176217 | 0.978181218 | 1.0133151 | down | hsa_circRNA_104584 | hsa_circ_0083861 | circBase | chr8 | - | 30557597 | 30560757 | exonic | NM_000637 | GSR | AATAACCAGCGATGGATTTTTTCAGCTGGAAGAATTGCCCGGTCCCATATAGAAATCATC |
| ASCRP3000793 | 0.540738615 | 0.919549314 | 1.0162477 | down | hsa_circRNA_042882 | hsa_circ_0042882 | circBase | chr17 | + | 29483000 | 29533389 | exonic | NM_000267 | NF1 | AATACGAATGGCACCGCTTCCAATAAAAACAGGACAGCAGAACACACATACCAAAGTCAG |
| ASCRP3000797 | 0.069714736 | 0.909237851 | 1.1226674 | down | hsa_circRNA_102898 | hsa_circ_0005307 | circBase | chr2 | + | 204154487 | 204157049 | exonic | NM_177538 | CYP20A1 | AATACTTGGCCATCTCCACACAAATATTGTCAGCATGTGCTTTGTGAAACTGTTCGAACT |
| ASCRP3000800 | 0.457357491 | 0.91119147 | 1.0261174 | down | hsa_circRNA_001035 | hsa_circ_0001035 | circBase | chr2 | - | 85554139 | 85554349 | sense overlapping | NM_006464 | TGOLN2 | AATAGACGGGCCCAGCAAGTCGGGTTCGGAGGCGCAGACCACAAAAGATGTCCCTAATAA |
| ASCRP3000801 | 0.707128974 | 0.945024804 | 1.0626327 | down | hsa_circRNA_400455 | | 25242744 | chr1 | - | 235409734 | 235424067 | exonic | NM_016374 | ARID4B | AATAGAGGAAGAAGATCTAATCATATGCCCTTGATGAGCCTCCCTATTTGACAGTGGGCA |
| ASCRP3000809 | 0.08275422 | 0.909237851 | 1.0690782 | down | hsa_circRNA_024596 | hsa_circ_0024596 | circBase | chr11 | - | 119545868 | 119549475 | exonic | NM_002855 | PVRL1 | AATATCACAGGCGTCCACTCCCAGGTGGTCCAGGTGAACGACTCCATGTATGGCTTCATC |
| ASCRP3000819 | 0.85377676 | 0.978900086 | 1.0093838 | down | hsa_circRNA_101869 | hsa_circ_0005016 | circBase | chr16 | + | 71954641 | 71957283 | exonic | NM_014761 | IST1 | AATATTCATCCACCTCAGATACCAGCAACTCCCCCATCGTATGAATCTGTTGCTGATCAG |
| ASCRP3000823 | 0.44979623 | 0.91119147 | 1.0406356 | down | hsa_circRNA_406971 | | 25070500 | chr7 | + | 79818266 | 79842185 | exonic | NM_002069 | GNAI1 | AATCAAAAAGAGCCCTCTCACTATATGCTATCCAGAATATGCAGGTGCTGGTGAATCTGG |
| ASCRP3000828 | 0.174939861 | 0.909237851 | 1.0740424 | down | hsa_circRNA_043614 | hsa_circ_0043614 | circBase | chr17 | - | 39738530 | 39743147 | exonic | NM_000526 | KRT14 | AATCAATACAGCTTCATTATCTGAGTTGCATAAACCCGAGCACCTTCTCTTCACTCAGCC |
| ASCRP3000832 | 0.296115759 | 0.909237851 | 1.1548061 | down | hsa_circRNA_404514 | | 25070500 | chr1 | + | 53222129 | 53250725 | exonic | NM_024646 | ZYG11B | AATCACCAGCAGGAGGAGGCGTCTCCCTATTCCTTACTTGATATCTGCTTGAATTTCTTG |
| ASCRP3000834 | 0.329712899 | 0.909237851 | 1.0506579 | down | hsa_circRNA_104463 | hsa_circ_0082002 | circBase | chr7 | + | 116339124 | 116340338 | exonic | NM_000245 | MET | AATCATGAGCACTGCTTTAATAGGATAAACCTCTCATAATGAAGGCCCCCGCTGTGCTTG |
| ASCRP3000835 | 0.018321998 | 0.909237851 | 1.0532912 | down | hsa_circRNA_076155 | hsa_circ_0076155 | circBase | chr6 | - | 35586872 | 35604935 | exonic | NM_004117 | FKBP5 | AATCCAAACGAAGGAGCAACAGTAGAAAATTGTCAAAAGAGTGGGGAATGGTGAGGAAAC |
| ASCRP3000843 | 0.450667972 | 0.91119147 | 1.0445289 | down | hsa_circRNA_055957 | hsa_circ_0055957 | circBase | chr2 | - | 107459490 | 107460490 | exonic | NM_032528 | ST6GAL2 | AATCCTCAACTCTTCCTTGGGCGAGGAAATAGGTTCATTCTGCCACCCATCTGCATTAAG |
| ASCRP3000852 | 0.083737873 | 0.909237851 | 1.0735575 | down | hsa_circRNA_054654 | hsa_circ_0054654 | circBase | chr2 | - | 56097854 | 56151298 | exonic | NM_001039348 | EFEMP1 | AATCTGGAAATGAAAATGGAGAGTTCTACCTACGACTAGAACCCTCTGGTCTCTGAGGGA |
| ASCRP3000856 | 0.079342886 | 0.909237851 | 1.1201761 | down | hsa_circRNA_010015 | hsa_circ_0010015 | circBase | chr1 | - | 12627938 | 12632881 | exonic | NM_004753 | DHRS3 | AATGAAAAATAAAGAGAGATGGCTTTTGTGAGTTTCCCAACCTCTTTCCCCCACTGAAGC |
| ASCRP3000857 | 0.979173429 | 0.997082313 | 1.0007041 | down | hsa_circRNA_103522 | hsa_circ_0068135 | circBase | chr3 | + | 180685838 | 180688146 | exonic | NM_005087 | FXR1 | AATGAAAATGGGCTAGGTACAAATTCTGAGCTGTCTAACCCCTCTGAAACGGAATCTGAG |
| ASCRP3000860 | 0.024332319 | 0.909237851 | 1.0554126 | down | hsa_circRNA_006899 | hsa_circ_0006899 | circBase | chr15 | + | 85180577 | 85184493 | sense overlapping | NR_003654 | SCAND2P | AATGAAGATCTCCCGGACTTCAAGGAGATTCAGAAAACGTCCAGTGCAGCCCTGCCTGAT |
| ASCRP3000862 | 0.199398677 | 0.909237851 | 1.046193 | down | hsa_circRNA_407043 | | 25070500 | chr8 | + | 16955966 | 16977910 | sense overlapping | NM_181723 | MICU3 | AATGAAGGAAAAGGTACTTAAAACAGATGCTGAGGAACTTGTCTCCAGAAGCTATTGGGA |
| ASCRP3000867 | 0.732834651 | 0.952949359 | 1.0588721 | down | hsa_circRNA_001795 | hsa_circ_0001795 | circBase | chr8 | - | 42294506 | 42323435 | exonic | NM_006749 | SLC20A2 | AATGACGTGAGGTTCCGCTGTGTGGCAGCTGATTGCTTCCTTCCTGAGGCTTCCAATCTC |
| ASCRP3000869 | 0.53672449 | 0.919549314 | 1.0187122 | down | hsa_circRNA_102479 | hsa_circ_0004071 | circBase | chr19 | + | 17308600 | 17311638 | exonic | NM_004145 | MYO9B | AATGAGCTCAAGTACCTGGACGAGTTCCTGCTCAACAAGGGAGAGGCGCACCTCCTTCTC |
| ASCRP3000878 | 0.343977658 | 0.91119147 | 1.0518255 | down | hsa_circRNA_104205 | hsa_circ_0007769 | circBase | chr6 | + | 144086397 | 144086935 | exonic | NM_014721 | PHACTR2 | AATGCAAAATGCTGGCTCCTCTCATTCAAAAAAAACAACTGGCTCTAAAGCATCAGCTTC |
| ASCRP3000886 | 0.69932828 | 0.944102043 | 1.0322853 | down | hsa_circRNA_404980 | | 25070500 | chr12 | - | 26780965 | 26808785 | exonic | NM_002223 | ITPR2 | AATGCGGAGACATCTGCCAGTGGATCTCCAGACACTTTACTACCATCAGATATGATTCTA |
| ASCRP3000888 | 0.574677454 | 0.922626849 | 1.0164821 | down | hsa_circRNA_103054 | hsa_circ_0060205 | circBase | chr20 | - | 35312815 | 35317187 | exonic | NM_022477 | NDRG3 | AATGCTGCCTCCTGTTCTTACCCACCTAAGGAACATGATATAGAAACAACTCATGGTGTG |
| ASCRP3000890 | 0.13694381 | 0.909237851 | 1.0447279 | down | hsa_circRNA_405339 | | 25070500 | chr15 | - | 50209130 | 50226379 | exonic | NM_024837 | ATP8B4 | AATGCTGTGGAAGTGAGAGAAGAACTCAGGAAAAAGAGCCTGTGGATTTCTCAGTCAAAT |
| ASCRP3000900 | 0.183147736 | 0.909237851 | 1.0382727 | down | hsa_circRNA_016598 | hsa_circ_0016598 | circBase | chr1 | + | 224918163 | 224918276 | exonic | NM_152495 | CNIH3 | AATGTCCCTCTACTTTTCTATCACTTCTGGAGCTGGTGCTGCCAGAATACTCCATCCATA |
| ASCRP3000905 | 0.707538104 | 0.945024804 | 1.0109036 | down | hsa_circRNA_065768 | hsa_circ_0065768 | circBase | chr3 | + | 50000008 | 50114685 | exonic | NM_005777 | RBM6 | AATGTGATTAGTACTGCTATAACCAGAATTTGGTAGAAAAAGGATTTACTTGTTGGGGCC |
| ASCRP3000907 | 0.545203389 | 0.919549314 | 1.0188265 | down | hsa_circRNA_406821 | | 25070500 | chr6 | + | 109175427 | 109197553 | exonic | NM_032131 | ARMC2 | AATGTTCAAAGGAACAACATCTTTACCATCTCATCTCAAGAATGGAGGGGTGTGGTGTCT |
| ASCRP3000913 | 0.309486309 | 0.909237851 | 1.0389899 | down | hsa_circRNA_091053 | hsa_circ_0091053 | circBase | chrX | - | 71492452 | 71496084 | exonic | NM_001007 | RPS4X | AATTAAAAATATTGTGGCAGGATTAATAGCAGCTCGTGGTCCCAAGAAGCATCTGAAGCG |
| ASCRP3000919 | 0.571941571 | 0.922333971 | 1.0284936 | down | hsa_circRNA_403615 | | 25242744 | chr6 | - | 45073658 | 45290704 | exonic | NM_181356 | SUPT3H | AATTAATCTGAAACTGCTTATCAATAACAGCTGTAGCACTGTGGCTTTTCTCCATGGACT |
| ASCRP3000926 | 0.673101682 | 0.938080316 | 1.0206386 | down | hsa_circRNA_023948 | hsa_circ_0023948 | circBase | chr11 | + | 85961337 | 85968638 | exonic | NM_003797 | EED | AATTCCATCCAAGAGATCCAAATCTTCTCCTGTCAGTAAGTAAAGGATGACGCTGTCAGT |
| ASCRP3000937 | 0.622572435 | 0.929544944 | 1.0198916 | down | hsa_circRNA_407020 | | 25070500 | chr7 | - | 140449086 | 140494267 | exonic | NM_004333 | BRAF | AATTGATGACTGGACAGTTACCTTATTCAAACATCAACAACAGGGACCAGGCCCCAAATT |
| ASCRP3000958 | 0.879518751 | 0.979863126 | 1.0110557 | down | hsa_circRNA_042688 | hsa_circ_0042688 | circBase | chr17 | - | 27041298 | 27043076 | exonic | NM_031934 | RAB34 | AATTTCTTTGTTACGAGCACTTATTTAAGATCTCCAAGGTCATTGTGGTGGGGGACCTGT |
| ASCRP3000970 | 0.842779967 | 0.978270321 | 1.0287189 | down | hsa_circRNA_059550 | hsa_circ_0059550 | circBase | chr20 | + | 19867164 | 19983103 | exonic | NM_001242581 | RIN2 | AATTTTTGAAATATCCTTCTTTCAGTGGTGTTGTTGAAAGGGCTTGGCAGCTCCCAAATG |
| ASCRP3000973 | 0.014737427 | 0.909237851 | 1.0867989 | down | hsa_circRNA_011717 | hsa_circ_0011717 | circBase | chr1 | + | 38273472 | 38275126 | exonic | NM_198446 | C1orf122 | ACAAAACACCTTCAGTAGAGCCTTGATTAAAAGGAAACCTGCAGACTCTCTCGGCCACGC |
| ASCRP3000981 | 0.640819349 | 0.929544944 | 1.0236648 | down | hsa_circRNA_100226 | hsa_circ_0005567 | circBase | chr1 | - | 51868106 | 51874004 | exonic | NM_001981 | EPS15 | ACAAACTTGGATTTTTTCCAGTCTGATCCTTTTGTTGGCAATCTCTTCTCTGAAAGCTGA |
| ASCRP3000986 | 0.417300784 | 0.91119147 | 1.0321545 | down | hsa_circRNA_058197 | hsa_circ_0058197 | circBase | chr2 | - | 217536827 | 217542954 | exonic | NM_000599 | IGFBP5 | ACAAAGCATTTATTTAATAAAGTTATGCATTCAGTTAGGCCCCTGCCGCAGACACATGGA |
| ASCRP3001017 | 0.643058632 | 0.929872547 | 1.0179037 | down | hsa_circRNA_101891 | hsa_circ_0005687 | circBase | chr16 | + | 84872027 | 84872341 | exonic | NM_031476 | CRISPLD2 | ACAAGCTTCGGGGCCAGGTGCAGCCTCAGGCCTCCAACATGGAGTACATGAGCTCAAGCG |
| ASCRP3001018 | 0.647096705 | 0.931689789 | 1.0200576 | down | hsa_circRNA_407041 | | 25070500 | chr8 | - | 16353301 | 16372347 | sense overlapping | ENST00000518026 | MSR1 | ACAAGGAAAGAAATCTCTCTGGGCCCTGCTTCTGTTTCTCAAAAGGTTACTTTTGTTGTT |
| ASCRP3001019 | 0.399780484 | 0.91119147 | 1.0316099 | down | hsa_circRNA_092389 | hsa_circ_0000329 | circBase | chr11 | + | 66281876 | 66287219 | exonic | NM_024649 | BBS1 | ACAAGGAGCTCCTGGTGCTTGACCCCGAGGCCTTCACCATTTTAGCCAAGCTGGTGGTAG |
| ASCRP3001025 | 0.462515883 | 0.91119147 | 1.0252221 | down | hsa_circRNA_102034 | hsa_circ_0005397 | circBase | chr17 | + | 30500849 | 30503232 | exonic | NM_018307 | RHOT1 | ACAAGTCGATGGATTCCTCTCATAAATGAAAGAACAGACAAAGACAGCAGGTTCCTCCCC |
| ASCRP3001038 | 0.039596797 | 0.909237851 | 1.1143686 | down | hsa_circRNA_008584 | hsa_circ_0008584 | circBase | chr18 | + | 7754370 | 7755918 | intronic | ENST00000332175 | PTPRM | ACACAAAACTTGTTCTCCCTCTAGTGTTCCACTTGCACAGGGGTACTTGAATCTAAAACA |
| ASCRP3001057 | 0.320572645 | 0.909237851 | 1.1923765 | down | hsa_circRNA_100033 | hsa_circ_0009581 | circBase | chr1 | - | 8555122 | 8601377 | exonic | NM_012102 | RERE | ACACCACTCTGAATGCACTGAACACAAGGGAAGTGTAACATCTCCCATTTTTCTGACATA |
| ASCRP3001060 | 0.863149183 | 0.979863126 | 1.0142243 | down | hsa_circRNA_004466 | hsa_circ_0004466 | circBase | chr12 | + | 112303053 | 112308984 | exonic | NM_003668 | MAPKAPK5 | ACACCCCAGTTCACCCCTTATTATGTAGCACCCCAGGAAACTTCCATTTTAGAAGAATAC |
| ASCRP3001065 | 0.502333523 | 0.914489113 | 1.0469161 | down | hsa_circRNA_034804 | hsa_circ_0034804 | circBase | chr15 | + | 42579888 | 42589854 | exonic | NM_198141 | GANC | ACACCTCTCAGGTTCCTCTCCTGGCTGAAATTTATGGTATAGAAGGAAACATTTTCAGGC |
| ASCRP3001074 | 0.265672856 | 0.909237851 | 1.0306858 | down | hsa_circRNA_103350 | hsa_circ_0065220 | circBase | chr3 | - | 47468936 | 47470160 | exonic | NM_012235 | SCAP | ACACTCAAAGTATGTGGGTGCCCCGGTGGCTTATGTCCAGCAGATATTTGTGAAGTCCTC |
| ASCRP3001095 | 0.089151174 | 0.909237851 | 1.0509116 | down | hsa_circRNA_101372 | hsa_circ_0006278 | circBase | chr14 | - | 68151731 | 68157138 | exonic | NM_016026 | RDH11 | ACAGAAGGTCTTGAGATTCTAAGTGGGAATCATTTCAGGTCACTTCCTCCTAACCCATCT |
| ASCRP3001102 | 0.817427173 | 0.976440085 | 1.0358113 | down | hsa_circRNA_405555 | | 25070500 | chr17 | - | 26918715 | 26926848 | intronic | ENST00000481916 | RP11-192H23.4 | ACAGACTGACACATCTCACAGTGGGGTTTCCCGTGCATAGGAGATCCAAGACAGCAGAAG |
| ASCRP3001114 | 0.198233728 | 0.909237851 | 1.052329 | down | hsa_circRNA_406688 | | 25070500 | chr5 | + | 141765092 | 141770616 | intronic | ENST00000510311 | AC005592.2 | ACAGATGGAAAAGGCGGCTCTCTGACTAGTGAAAGGGATCACAGTATAAGACAGTGAATA |
| ASCRP3001128 | 0.937897262 | 0.988165332 | 1.0051443 | down | hsa_circRNA_008046 | hsa_circ_0008046 | circBase | chr14 | + | 31103152 | 31112611 | exonic | NM_016106 | SCFD1 | ACAGCCTCTTCTGCTTTTTTGTTACTCTGGGCTTTTACACTCTGATCGAGATCCTATTCC |
| ASCRP3001129 | 0.081577298 | 0.909237851 | 1.0572012 | down | hsa_circRNA_406479 | | 25070500 | chr4 | - | 54811039 | 54817857 | antisense | uc003haa.3 | PDGFRA | ACAGCGAAAAGACCTACAGCACTCTGCAAAAGGCATTCTTCACCAAAGTGTGTCTCCTTG |
| ASCRP3001138 | 0.194853453 | 0.909237851 | 1.0650773 | down | hsa_circRNA_028319 | hsa_circ_0028319 | circBase | chr12 | - | 112370389 | 112381173 | exonic | NM_138341 | TMEM116 | ACAGGACACCAAGCTTCACATGGCCCTTTATGTTCTCCAGATATTCTACATTTCCTCATT |
| ASCRP3001159 | 0.621276027 | 0.929544944 | 1.0176413 | down | hsa_circRNA_079518 | hsa_circ_0079518 | circBase | chr7 | - | 17890448 | 17937069 | exonic | NM_015132 | SNX13 | ACAGTATCTTAGATCTCTAGATACAGCTGGTGATGGCCAGTCTATCCATATGGGGATGGG |
| ASCRP3001160 | 0.071160374 | 0.909237851 | 1.0593129 | down | hsa_circRNA_104736 | hsa_circ_0086414 | circBase | chr9 | - | 16435552 | 16437522 | exonic | NM_017637 | BNC2 | ACAGTGGCTGGTTGCAATGCTGCATTCCCCTCTCGCCGAAGCCGAGACAGGATGCTGCTG |
| ASCRP3001161 | 0.382863098 | 0.91119147 | 1.0322225 | down | hsa_circRNA_030788 | hsa_circ_0030788 | circBase | chr13 | - | 101997616 | 102031004 | exonic | NM_052867 | NALCN | ACAGTGGCTTTAATGAGATAGGGGGATAGTTCCTATGTGAAAGATCGCTGGTGTGTTTTT |
| ASCRP3001162 | 0.070100007 | 0.909237851 | 1.0709286 | down | hsa_circRNA_100423 | hsa_circ_0006267 | circBase | chr1 | + | 198247090 | 198266370 | exonic | NM_133494 | NEK7 | ACAGTGTGACTACCCACCTCTTCCTTCAGATCACTATTCAGAAGAACATTTTAAGAAGCA |
| ASCRP3001163 | 0.064459491 | 0.909237851 | 1.0395459 | down | hsa_circRNA_405952 | | 25070500 | chr2 | - | 122125405 | 122135069 | sense overlapping | NM_015282 | CLASP1 | ACAGTGTGCTGTGCTGCAGGTTATCCCTAGGCTAAAAGAGAACTCTGTATGAGTTAAATG |
| ASCRP3001171 | 0.921878624 | 0.9834955 | 1.0130027 | down | hsa_circRNA_406007 | | 25070500 | chr2 | + | 190584297 | 190585513 | exonic | NM_144708 | ANKAR | ACATAAAGGCCTCCCATATCTTATCAGATTTCTGAGTTCTGATTCAGGCACCATTCCTGC |
| ASCRP3001175 | 0.046941453 | 0.909237851 | 1.1658907 | down | hsa_circRNA_402346 | | 25242744 | chr2 | + | 113258782 | 113260758 | exonic | NM_153712 | TTL | ACATATAATAAGGTGAAGGCATTCTCATCTCCTCAGAGGCTTCAGAGCTTCTCGATTTCA |
| ASCRP3001177 | 0.056638732 | 0.909237851 | 1.0666801 | down | hsa_circRNA_035484 | hsa_circ_0035484 | circBase | chr15 | + | 59397283 | 59417244 | exonic | NM_004701 | CCNB2 | ACATATATTGAGGAAAAATAAAGCGATTGGTTTTTCTTAAGGTAGGGCCGCCCAATGGGG |
| ASCRP3001178 | 0.029499387 | 0.909237851 | 1.0849687 | down | hsa_circRNA_070520 | hsa_circ_0070520 | circBase | chr4 | - | 103226145 | 103236987 | exonic | NM_022154 | SLC39A8 | ACATATGGTCAGTGTTTAACTGCTGAAGAGATCTTTTCCCTTCATGGCTTTTCAAATGCT |
| ASCRP3001188 | 0.703565712 | 0.944417633 | 1.0132914 | down | hsa_circRNA_009725 | hsa_circ_0009725 | circBase | chr1 | + | 10460449 | 10480201 | exonic | uc001arc.3 | PGD | ACATCAGACTGAGTCTGTGCTTTTAATAGGACTGTCTCCAAAGTTGATGATTTCTTGGCC |
| ASCRP3001208 | 0.833968702 | 0.978181218 | 1.0103951 | down | hsa_circRNA_104327 | hsa_circ_0001971 | circBase | chr7 | - | 23015828 | 23023664 | exonic | NM_032581 | FAM126A | ACATGCCCAGTGTTTCTCTTCAGTCACTGTGTCAAATTTGTTCAAGACATTACCAGAAAC |
| ASCRP3001210 | 0.973495274 | 0.996425773 | 1.0018783 | down | hsa_circRNA_006471 | hsa_circ_0006471 | circBase | chr19 | - | 11623870 | 11625036 | exonic | NM_016581 | ECSIT | ACATGGAGCCTGACCTTAGTGCCAGGGTCACCATCTACCAGGTCCCTCGCCGGCTCCCTC |
| ASCRP3001254 | 0.658670256 | 0.933884861 | 1.0230838 | down | hsa_circRNA_001991 | hsa_circ_0001991 | circBase | chr5 | + | 80299959 | 80338802 | sense overlapping | NM_006909 | RASGRF2 | ACCAAGCCAGGCACCTCTGATCACAGCCTAAGAGCATGTAAGGTTCTCTGGAAGGATTTG |
| ASCRP3001256 | 0.379126599 | 0.91119147 | 1.0873214 | down | hsa_circRNA_048085 | hsa_circ_0048085 | circBase | chr19 | + | 676388 | 683392 | exonic | NM_005860 | FSTL3 | ACCAAGGAAATAAAGACTCAAGCCATTTGGGAAGTCGGTGCCGCTGCCGTCTCTGCGTTC |
| ASCRP3001262 | 0.344412249 | 0.91119147 | 1.063416 | down | hsa_circRNA_004646 | hsa_circ_0004646 | circBase | chr1 | + | 162546566 | 162567648 | exonic | NM_003115 | UAP1 | ACCAATCCAATGTGAAATCTCTCCTCTTATCTCCTATGCTGGAGAAGACTTTTCCAGATT |
| ASCRP3001274 | 0.857035532 | 0.978983332 | 1.0080183 | down | hsa_circRNA_103337 | hsa_circ_0065052 | circBase | chr3 | - | 44986659 | 45000952 | exonic | NM_016598 | ZDHHC3 | ACCACTGCAGATGCCAGTATGGACAGAATAGCTTATGATGCTTATCCCCACCCACCACTT |
| ASCRP3001275 | 0.16012054 | 0.909237851 | 1.1381582 | down | hsa_circRNA_403676 | | 25242744 | chr6 | - | 99930628 | 99958106 | exonic | NM_001080481 | USP45 | ACCACTTTCTCCTAAAGTTCTTTTTAATCAGCTTTGTCAGAATAAATAACAGATGCGGGT |
| ASCRP3001285 | 0.473580723 | 0.91119147 | 1.0424502 | down | hsa_circRNA_104179 | hsa_circ_0077705 | circBase | chr6 | - | 116966876 | 116982009 | exonic | NM_145062 | ZUFSP | ACCAGATATTGGCAGTAGAGGGTGCTCTTTCTCTAGAGGAGAAACTTACTGTGATCAACC |
| ASCRP3001298 | 0.743199926 | 0.95565436 | 1.0110398 | down | hsa_circRNA_087331 | hsa_circ_0087331 | circBase | chr9 | - | 84267098 | 84300811 | exonic | NM_001303103 | TLE1 | ACCAGGTCTACCTCCTACACCTTAAATTGGAATGTGAGAAACTGGCAAGTGAAAAGACAG |
| ASCRP3001300 | 0.927680185 | 0.985498678 | 1.0025711 | down | hsa_circRNA_058695 | hsa_circ_0058695 | circBase | chr2 | + | 232952196 | 233199209 | exonic | NM_152383 | DIS3L2 | ACCAGTCTCTTCTTTGCTGTTCTGGTCAAGGTAGTTAAACCAGAGAGCAATGACAAAGAA |
| ASCRP3001309 | 0.004008592 | 0.870215632 | 1.0730847 | down | hsa_circRNA_101096 | hsa_circ_0027491 | circBase | chr12 | + | 69210591 | 69218431 | exonic | NM_002392 | MDM2 | ACCATCTACCTCATCTAGAAGGAGAGCAATTAGTGAGACAGGTTCTTTTTTATCTTGGCC |
| ASCRP3001333 | 0.292995597 | 0.909237851 | 1.0365041 | down | hsa_circRNA_407188 | | 25070500 | chr9 | - | 74522734 | 74523039 | intronic | ENST00000333421 | ABHD17B | ACCCATCACCCAGTTAAACAAACAAAACTACCAATTAAAACGAAGTCCACTTCACTGTTT |
| ASCRP3001339 | 0.674593073 | 0.93829504 | 1.0157212 | down | hsa_circRNA_007084 | hsa_circ_0007084 | circBase | chr10 | + | 22002700 | 22016857 | exonic | NM_004641 | MLLT10 | ACCCCGAGGAAGTCTCTCGCCACGGTATTTATAACAGCAATGATGTAGCAGTATCGTTTC |
| ASCRP3001340 | 0.324388553 | 0.909237851 | 1.0512164 | down | hsa_circRNA_011538 | hsa_circ_0011538 | circBase | chr1 | + | 35824525 | 35855699 | exonic | NM_005095 | ZMYM4 | ACCCGCCTTCACAAAATAATGCAGGTGGTGGTATCATGGATACAGAAATGTCTGAAGATA |
| ASCRP3001341 | 0.434375157 | 0.91119147 | 1.0266479 | down | hsa_circRNA_000596 | hsa_circ_0000661 | circBase | chr15 | + | 100645330 | 100645426 | antisense | NM_139057 | ADAMTS17 | ACCCGGGAGATGGTGCTGTTGCCACCCACCAGGATGGTGGCTCTTTTGTAAAGACTGGTG |
| ASCRP3001344 | 0.466052125 | 0.91119147 | 1.0279122 | down | hsa_circRNA_103523 | hsa_circ_0068142 | circBase | chr3 | + | 182602540 | 182605501 | exonic | NM_014616 | ATP11B | ACCCTCATGTGTTACAAAATAAGCCCACCCTTTATCGGAATCATGGGTAAAGAAGGAAGA |
| ASCRP3001345 | 0.102224564 | 0.909237851 | 1.0836651 | down | hsa_circRNA_102830 | hsa_circ_0001073 | circBase | chr2 | + | 148653869 | 148657467 | exonic | NM_001616 | ACVR2A | ACCCTCCTGTACTTGTTCCAACTCAAGTGCTATACTTGGTAGATCAGAAACTCAGGAGTG |
| ASCRP3001346 | 0.536756959 | 0.919549314 | 1.0291455 | down | hsa_circRNA_023420 | hsa_circ_0023420 | circBase | chr11 | - | 71808337 | 71808960 | exonic | NM_017907 | LAMTOR1 | ACCCTCGATAAAGGAAGTATATTCATCCAGTCTCCAGGATAGCTGCTTATGCCTACAGTG |
| ASCRP3001363 | 0.822609139 | 0.976440085 | 1.0242741 | down | hsa_circRNA_401999 | | 25242744 | chr19 | - | 430683 | 440932 | exonic | NM_012435 | SHC2 | ACCGGTACAGTACATGGGCTGCATCGAGGTTCTCCGCTCTATGCGCTCCCTGGACTTTAA |
| ASCRP3001365 | 0.215120447 | 0.909237851 | 1.1267526 | down | hsa_circRNA_018818 | hsa_circ_0018818 | circBase | chr10 | - | 75264607 | 75265184 | exonic | NM_152586 | USP54 | ACCGTTGTCTCAGAGAGGGAGGAAGCTCCGGTTTCTTCCCACAGTTTTGCTCTGCAGATG |
| ASCRP3001377 | 0.325040773 | 0.909237851 | 1.0921781 | down | hsa_circRNA_104572 | hsa_circ_0007618 | circBase | chr8 | + | 25265498 | 25266456 | exonic | NM_024940 | DOCK5 | ACCTCCACTCACTCCCAAAGCCACCAGGACCCTAACTCAATCTTGGAGCCACTTTTGGAG |
| ASCRP3001391 | 0.950702201 | 0.992077624 | 1.0018685 | down | hsa_circRNA_101695 | hsa_circ_0007146 | circBase | chr16 | - | 5077135 | 5078186 | exonic | NM_016256 | NAGPA | ACCTGAAGCCACCCTGAGGGCGGGAGAACTCTCCTTTTTCACCAGCCAGGACAACATGTG |
| ASCRP3001394 | 0.353631323 | 0.91119147 | 1.0232476 | down | hsa_circRNA_053294 | hsa_circ_0053294 | circBase | chr2 | + | 27820936 | 27830906 | exonic | NM_001271286 | ZNF512 | ACCTGATGATCGAAAGCAGGACCCAGTGCTCCATAAAGGATAATAGTTTCCAGTACACTA |
| ASCRP3001397 | 0.859322802 | 0.97947761 | 1.0057836 | down | hsa_circRNA_009031 | hsa_circ_0009031 | circBase | chr4 | + | 4650703 | 4651269 | exonic | NR_037888 | STX18-AS1 | ACCTGCCCCTAGATCTTCTATTTCTGATCATCTTCTATGCAAGGATGCAGAGATTCAGAG |
| ASCRP3001405 | 0.179278508 | 0.909237851 | 1.0469603 | down | hsa_circRNA_059711 | hsa_circ_0059711 | circBase | chr20 | + | 30330343 | 30389603 | exonic | NM_012112 | TPX2 | ACCTGGCCTGGAATAAATACGTTTTGTCTTTCCCTCGTTCTTGATACATATTTGCCAGAC |
| ASCRP3001408 | 0.526088817 | 0.916971445 | 1.0251017 | down | hsa_circRNA_007475 | hsa_circ_0007475 | circBase | chr12 | - | 51639798 | 51640615 | sense overlapping | NM_001031628 | SMAGP | ACCTGTACAAGAACAAAGGCAGCTACGTCACCTATGAACCTACAGAAGAAGAACTGATGA |
| ASCRP3001415 | 0.446641905 | 0.91119147 | 1.0730075 | down | hsa_circRNA_100683 | hsa_circ_0020028 | circBase | chr10 | + | 112723882 | 112745523 | exonic | NM_007373 | SHOC2 | ACCTTGACTTGCAGCACAATGAACTGCTAGACCTCCCAGATACTATAGGAACTCTAATGA |
| ASCRP3001421 | 0.384113395 | 0.91119147 | 1.0578854 | down | hsa_circRNA_001100 | hsa_circ_0000877 | circBase | chr19 | + | 4445536 | 4447603 | sense overlapping | NR_039901 | MIR4746 | ACCTTGTTCTGCAGCTTGATCTTCCGGTACTTCTCCTCCTCGGGGTGCAGGATGGAGTCC |
| ASCRP3001432 | 0.900243959 | 0.981257955 | 1.0043577 | down | hsa_circRNA_406800 | | 25070500 | chr6 | - | 77921697 | 77983024 | intergenic |  |  | ACGAAGATTGAGCCCAGCATCTTGCTATTAAGGAAAAGTATGAGCCTTGCAAGAAGACAT |
| ASCRP3001444 | 0.620842806 | 0.929544944 | 1.0292163 | down | hsa_circRNA_103081 | hsa_circ_0002168 | circBase | chr20 | - | 48744511 | 48747484 | exonic | NM_199129 | TMEM189 | ACGAGACCTACTTCTGCATCACCACAGTTGCAGGGGCTCTCATTGCTGACTTCTTGTCTG |
| ASCRP3001449 | 0.091075625 | 0.909237851 | 1.1050369 | down | hsa_circRNA_404904 | | 25070500 | chr11 | + | 71369656 | 71370884 | intergenic |  |  | ACGAGTTTGTCATCATGTGAGAGAGAATAGGTCTGTTCTTCCCCTGGATTGTAAACTCCT |
| ASCRP3001453 | 0.676869432 | 0.939319494 | 1.0205733 | down | hsa_circRNA_101722 | hsa_circ_0038111 | circBase | chr16 | + | 16146580 | 16150152 | exonic | NM_004996 | ABCC1 | ACGCCCTTTCTGAATCTGGGCCCTTCCGTCCTGGCTGGAGTGGCGGTGATGGTCCTCATG |
| ASCRP3001454 | 0.330555211 | 0.909237851 | 1.1047464 | down | hsa_circRNA_100685 | hsa_circ_0020080 | circBase | chr10 | + | 116590610 | 116608496 | exonic | NM_020940 | FAM160B1 | ACGCTCATAGGCAGCTTGCACCTTCTCTTCCTTTACAAGAAGATTTTGTTTATCACTGGA |
| ASCRP3001460 | 0.245733057 | 0.909237851 | 1.0366815 | down | hsa_circRNA_090181 | hsa_circ_0090181 | circBase | chrX | - | 32715986 | 32717410 | exonic | NM_000109 | DMD | ACGGAGCCCATTTCCTTCACAGATGTTGATACCACCTATCCAGATAAGAAGTCCATCTTA |
| ASCRP3001476 | 0.1749585 | 0.909237851 | 1.0665773 | down | hsa_circRNA_060918 | hsa_circ_0060918 | circBase | chr20 | - | 52192265 | 52199707 | exonic | uc002xwq.4 | ZNF217 | ACGTTAGAAGGGTTTGGAAATCCCTTGTCTCCAGGTTGCTGGGATTGACTTCTTGCTCAA |
| ASCRP3001486 | 0.268773109 | 0.909237851 | 1.0211078 | down | hsa_circRNA_005895 | hsa_circ_0005895 | circBase | chr5 | + | 40852275 | 40854198 | intronic | ENST00000381677 | CARD6 | ACTAAGACCTGCTTCTCAGCAAGGAGTCCAGATGAAGACACAAGAAAGAAAAAAGGTGTT |
| ASCRP3001495 | 0.035151768 | 0.909237851 | 1.0991074 | down | hsa_circRNA_404147 | | 25242744 | chr9 | + | 20715320 | 20789607 | exonic | NM_017794 | FOCAD | ACTACAGAATTAGCCCAAGCAGATTCCTCCCAGAAGCTGCACACATACATGTAAGAGAAG |
| ASCRP3001508 | 0.617144266 | 0.929544944 | 1.0329218 | down | hsa_circRNA_101486 | hsa_circ_0034682 | circBase | chr15 | + | 41535864 | 41555081 | exonic | NM_007236 | CHP1 | ACTCAACAGCCGAAGCAACAAACTGCACTTTTCCCACAGTCAAATCACTCGCCTCTACAG |
| ASCRP3001524 | 0.433440795 | 0.91119147 | 1.076059 | down | hsa_circRNA_090446 | hsa_circ_0090446 | circBase | chrX | + | 47441689 | 47446190 | exonic | NM_003254 | TIMP1 | ACTCCCATCTTTCTTCCGGACAATGAAATAAAGAGTTACCACCCAGCAGATTTCGTCGGC |
| ASCRP3001525 | 0.195205557 | 0.909237851 | 1.0487724 | down | hsa_circRNA_068697 | hsa_circ_0068697 | circBase | chr3 | - | 196846280 | 196869639 | exonic | NM_004087 | DLG1 | ACTCCCCAGTTTCTAAAGCAGTACTTGGAGATGATGAAATTACAAGGTTAATGGCACAGA |
| ASCRP3001527 | 0.951105872 | 0.992077624 | 1.0021575 | down | hsa_circRNA_060342 | hsa_circ_0060342 | circBase | chr20 | + | 37159809 | 37161502 | exonic | NM_020336 | RALGAPB | ACTCCCGATAGTGAGAGACCTGCTCAAGCTCTCTTAAGAGATTATGGGGCAATGTTAAAT |
| ASCRP3001537 | 0.455201244 | 0.91119147 | 1.0739299 | down | hsa_circRNA_405032 | | 25070500 | chr12 | + | 85459039 | 85460676 | exonic | NM_001079910 | LRRIQ1 | ACTCGAATTGGTTACAACAGTTACATTTCAAGATTTGCCAGGCTGTGTTCTCTCCACACT |
| ASCRP3001539 | 0.917732097 | 0.98346125 | 1.0123953 | down | hsa_circRNA_404833 | | 25070500 | chr11 | - | 3726429 | 3733958 | exonic | NM_016320 | NUP98 | ACTCGTTCACTAGGTCTCCCATTTTTCTAAGTATGGCCTTCAGGATTCTGATGAAGAGGA |
| ASCRP3001544 | 0.153164596 | 0.909237851 | 1.0451052 | down | hsa_circRNA_101189 | hsa_circ_0029033 | circBase | chr12 | + | 122380439 | 122380881 | exonic | NM_144668 | WDR66 | ACTCTCGAACTCCCCACAGAGTACGGTGTTCAGTATTCCTGTGCACACAATATTTGACAG |
| ASCRP3001553 | 0.150971522 | 0.909237851 | 1.0815545 | down | hsa_circRNA_100806 | hsa_circ_0007061 | circBase | chr11 | - | 46515157 | 46534363 | exonic | NM_017749 | AMBRA1 | ACTCTTCTGTTAACAGGGTCCTGGCAGGAGGCTGCTGGAATCTTCCCTCATTTCATTATC |
| ASCRP3001563 | 0.809699799 | 0.974638647 | 1.0057364 | down | hsa_circRNA_007335 | hsa_circ_0007335 | circBase | chr10 | + | 76978820 | 76979114 | exonic | NM_003375 | VDAC2 | ACTGACATTTGATACTACCTTCTCACCAAACACAGGGAATTTTCAACGTCCGGTTCATCT |
| ASCRP3001577 | 0.216089215 | 0.909237851 | 1.0377425 | down | hsa_circRNA_405082 | | 25070500 | chr12 | + | 122337539 | 122341013 | exonic | NM_002813 | PSMD9 | ACTGCATAACATTGGCAGTGTGGTGCAGCACAGTGAGGGGGCCTGCAGAATGATCACAAG |
| ASCRP3001584 | 0.423484515 | 0.91119147 | 1.0226126 | down | hsa_circRNA_100269 | hsa_circ_0013048 | circBase | chr1 | + | 82302569 | 82372915 | exonic | NM_012302 | LPHN2 | ACTGCTACCTCCCCGATGCCTTCAAAATTATGACTCAAAGATATGAAGATCAATGATGCA |
| ASCRP3001599 | 0.523896718 | 0.916971445 | 1.0265562 | down | hsa_circRNA_101182 | hsa_circ_0004479 | circBase | chr12 | - | 121220457 | 121229360 | exonic | NM_139015 | SPPL3 | ACTGGCCATTGGCTTCTCATGGATGGTCCCTTAATATGGACTTTGAAAATCAAGATAAGG |
| ASCRP3001609 | 0.282105444 | 0.909237851 | 1.0465402 | down | hsa_circRNA_004390 | hsa_circ_0004390 | circBase | chr1 | - | 85331067 | 85331821 | exonic | NM_012152 | LPAR3 | ACTGTCTTAGGATGTTCACTTCTCCACAATGAATGAGTGTCACTATGACAAGCACATGGA |
| ASCRP3001618 | 0.734929679 | 0.952949359 | 1.0125905 | down | hsa_circRNA_406499 | | 25070500 | chr4 | - | 87275682 | 87292020 | exonic | uc003hpv.3 | MAPK10 | ACTGTTGAATTAGACCCGTTTCCTATAGATGAGAAACCATACAAGCTGTGAACCATAATT |
| ASCRP3001619 | 0.699479551 | 0.944102043 | 1.0310835 | down | hsa_circRNA_086368 | hsa_circ_0086368 | circBase | chr9 | - | 14081841 | 14113080 | exonic | NM_001190737 | NFIB | ACTGTTTAAACAATAAAATGAGCTATGCTACAGACTCTGCCTACACAGCCTCAGGCACAT |
| ASCRP3001624 | 0.862561633 | 0.979863126 | 1.0062375 | down | hsa_circRNA_104328 | hsa_circ_0005251 | circBase | chr7 | - | 23015828 | 23030758 | exonic | NM_032581 | FAM126A | ACTTACATGCCCAGTGTTTCTCTTCAGTCACTGTGTCAAATTTGTTCAAGGTTGAAGGTT |
| ASCRP3001633 | 0.49989679 | 0.91447302 | 1.0292372 | down | hsa_circRNA_089921 | hsa_circ_0089921 | circBase | chrX | - | 15444028 | 15477863 | exonic | NM_003662 | PIR | ACTTCAAATTGGACCCAGGAGCCAAACATTCCCAACCTATCCCTAAAGGTATCCTACCTC |
| ASCRP3001643 | 0.474142723 | 0.91119147 | 1.1930935 | down | hsa_circRNA_001788 | hsa_circ_0001788 | circBase | chr8 | + | 37623043 | 37623873 | exonic | NM_007198 | PROSC | ACTTCATTGGCCACCTACAGAAACAAAATGTCAACAAATTGATGGGATCTCCCAGCCATC |
| ASCRP3001644 | 0.769619468 | 0.96364966 | 1.0124534 | down | hsa_circRNA_100604 | hsa_circ_0009172 | circBase | chr10 | - | 70218860 | 70229920 | exonic | NR_102264 | DNA2 | ACTTCCCTCAGATGCAGCTCTCTCTATTTCAGAAGAAAGTGGTAGCTTCCTTTCCAAGAA |
| ASCRP3001666 | 0.160805624 | 0.909237851 | 1.0499184 | down | hsa_circRNA_100372 | hsa_circ_0015004 | circBase | chr1 | + | 161293403 | 161326630 | exonic | NM_003001 | SDHC | ACTTGTCTTCCCTCTCATGTATCATACCTGGAATGGGATCCGACACTTGACACGTTGGTC |
| ASCRP3001668 | 0.068357802 | 0.909237851 | 1.1051767 | down | hsa_circRNA_405543 | | 25070500 | chr17 | + | 15907201 | 15907487 | intronic | ENST00000261647 | TTC19 | ACTTGTTATGAGTAAGGACATTGCCTAGTATTGGCCCCTCACTGAAGCAGTTTTCAAAAC |
| ASCRP3001673 | 0.959268056 | 0.993354918 | 1.0009134 | down | hsa_circRNA_406672 | | 25070500 | chr5 | - | 136566487 | 136566586 | intronic | ENST00000282223 | SPOCK1 | ACTTTCTGGGGAGGTTGGGTAAAGGAAAATGGAATTTTTGTAAGAAGTCACTTTCTGGGG |
| ASCRP3001695 | 0.618457582 | 0.929544944 | 1.034381 | down | hsa_circRNA_406725 | | 25070500 | chr5 | - | 180286875 | 180286984 | intronic | ENST00000359141 | ZFP62 | AGAAACTAAACAGCCATCTATTCGGGGGCATTTGGGTTGTCTCCAATTTGTTGTTACTTC |
| ASCRP3001698 | 0.309254675 | 0.909237851 | 1.090667 | down | hsa_circRNA_103686 | hsa_circ_0070348 | circBase | chr4 | - | 85781564 | 85781775 | exonic | NM_014991 | WDFY3 | AGAAACTGTATATGATGCTGCCAGTGTTTAACAGGAATTAGAAGTGCGTCTCACCAACCA |
| ASCRP3001700 | 0.269611085 | 0.909237851 | 1.0224579 | down | hsa_circRNA_403312 | | 25242744 | chr5 | - | 31406959 | 31410994 | exonic | NM_013235 | DROSHA | AGAAAGAATAGGCTGTGGGAAAGGACCAAGTTGTTGCGAAGCTCTTTGGTGAATAATAGA |
| ASCRP3001706 | 0.142820977 | 0.909237851 | 1.0516955 | down | hsa_circRNA_404707 | | 25070500 | chr10 | + | 3150990 | 3151271 | intronic | ENST00000381075 | PFKP | AGAAAGGCTGTGTTTCTATTGGGAAGGGTTGGGCATCTTCACAAGGTCTCTAGGAGGAAG |
| ASCRP3001710 | 0.269267865 | 0.909237851 | 1.0352124 | down | hsa_circRNA_400651 | | 25242744 | chr10 | + | 115636279 | 115644139 | exonic | NM_198514 | NHLRC2 | AGAACAACAACCCATTAGTTCCCCTTGGGATGTAGTTTTTGGAACATCAGATGGTCTTCT |
| ASCRP3001754 | 0.548271759 | 0.919549314 | 1.0198365 | down | hsa_circRNA_405776 | | 25070500 | chr19 | + | 37579940 | 37587017 | exonic | ENST00000587082 | ZNF420 | AGAAGTCTGCCATCTGTTTCATTGTTATTCCTTTTCTCTGCATTCTCCAGACTCTGTGCT |
| ASCRP3001756 | 0.877200596 | 0.979863126 | 1.0038406 | down | hsa_circRNA_001835 | hsa_circ_0001835 | circBase | chr9 | + | 2643266 | 2643521 | sense overlapping | NM_003383 | VLDLR | AGAAGTGGCGATGTGATGGGGACCCTGACTGCAAGGATGGCAGTGATGAGCTGGACTGTG |
| ASCRP3001759 | 0.468531944 | 0.91119147 | 1.0287922 | down | hsa_circRNA_013093 | hsa_circ_0013093 | circBase | chr1 | + | 89206670 | 89251896 | exonic | NM_006256 | PKN2 | AGAAGTTTACACTGGAACTGGACAGGGGGGATTCCCGAAGTCTTCCGTTTTCTGAGAATG |
| ASCRP3001760 | 0.368315011 | 0.91119147 | 1.0355733 | down | hsa_circRNA_104232 | hsa_circ_0078357 | circBase | chr6 | + | 155063089 | 155099179 | exonic | NM_001286189 | SCAF8 | AGAAGTTTATTCAGAAAGATTGTGCCTCTACTCCTGCTTGTACAGACTGCTTTTCCAGCA |
| ASCRP3001767 | 0.384422179 | 0.91119147 | 1.0570505 | down | hsa_circRNA_103813 | hsa_circ_0072255 | circBase | chr5 | - | 37326002 | 37328522 | exonic | NM_004298 | NUP155 | AGAATATTCAAGAGTGGCAACAGAGAGATCACTGCAGTTCTCCTGTTCCTAGTGGTAGTC |
| ASCRP3001787 | 0.007150437 | 0.879037174 | 1.2033957 | down | hsa_circRNA_405709 | | 25070500 | chr19 | - | 334399 | 336173 | exonic | NM_017550 | MIER2 | AGACAAGCCCAAGGAGGAGCTGGAGAAGGACTTCATCTCCCAGGCCTCCTCGCTGGGGAG |
| ASCRP3001789 | 0.047655261 | 0.909237851 | 1.1141926 | down | hsa_circRNA_104838 | hsa_circ_0087641 | circBase | chr9 | - | 99284787 | 99327765 | exonic | NM_003671 | CDC14B | AGACAATCCAAAACAAACGCTATTCCTCTCACATCGCCTTTGTTTTGCCATTCTCTACAG |
| ASCRP3001794 | 0.057551621 | 0.909237851 | 1.111686 | down | hsa_circRNA_028152 | hsa_circ_0028152 | circBase | chr12 | - | 110397651 | 110399490 | exonic | NM_014776 | GIT2 | AGACAGCAGGGCAGTTCTCTCTCGGGTTCAAAAGCTAAGTAATCATTTGTTTGAAGAACT |
| ASCRP3001795 | 0.695441401 | 0.943480789 | 1.0122002 | down | hsa_circRNA_404815 | | 25070500 | chr10 | + | 122395277 | 122395661 | intronic | NR_049879 | MIR5694 | AGACAGCAGTTGTCAGGGAGCCCAGCTTTCAGATGTGATGTCTAAATGAGAATTGAAATA |
| ASCRP3001797 | 0.854723739 | 0.978900086 | 1.0079308 | down | hsa_circRNA_103143 | hsa_circ_0003342 | circBase | chr21 | + | 45497573 | 45500008 | exonic | NM_003274 | TRAPPC10 | AGACAGCCCAGGATTTGTCCCATGCCACCATTGAAATGTATACAAGCATTGGGAGGATTC |
| ASCRP3001801 | 0.843893491 | 0.978270321 | 1.006087 | down | hsa_circRNA_103689 | hsa_circ_0007324 | circBase | chr4 | + | 87693930 | 87696805 | exonic | NM_006264 | PTPN13 | AGACATGTCACTTCCTTCATTGGTATTGAAAGCAACAAGTAATCCTTCCCCTCTACCACC |
| ASCRP3001809 | 0.069170147 | 0.909237851 | 1.1668338 | down | hsa_circRNA_102628 | hsa_circ_0052767 | circBase | chr2 | - | 15601324 | 15651474 | exonic | NM_015909 | NBAS | AGACCACTTCTCCACATGAATATTCTGTTTTGCTGCCCGAAGCTTGACTTTTACTTGTTG |
| ASCRP3001810 | 0.764459597 | 0.962339655 | 1.0222154 | down | hsa_circRNA_104414 | hsa_circ_0080797 | circBase | chr7 | + | 76062243 | 76062964 | exonic | NM_007155 | ZP3 | AGACCAGAATGCCTCCCCTTATCACACCATCGTGGACTTCCATGGGCAGGGCAATGTGAG |
| ASCRP3001811 | 0.448336963 | 0.91119147 | 1.0322148 | down | hsa_circRNA_401289 | | 25242744 | chr14 | - | 77812691 | 77818094 | exonic | NM_213601 | TMED8 | AGACCAGGCCCAGGTCCTCAATGAGAGAATGAAGATCTAGAAAACAAGGATACCTCTTTA |
| ASCRP3001816 | 0.058198981 | 0.909237851 | 1.0519866 | down | hsa_circRNA_101947 | hsa_circ_0041481 | circBase | chr17 | - | 3853804 | 3854067 | exonic | NM_005173 | ATP2A3 | AGACCCCAGAGCTGTGAACCAGGACAAGAAGAACATGCTGTTTTCTTGGGGGACAAAGTG |
| ASCRP3001817 | 0.838912582 | 0.978181218 | 1.0092205 | down | hsa_circRNA_405108 | | 25070500 | chr12 | + | 132512561 | 132529498 | exonic | NM_015409 | EP400 | AGACCCCTCAGGAGGAAAAGACCAGACTCTTGAAAGAGCGCCTGGATCAGATTTATTTAG |
| ASCRP3001818 | 0.465610366 | 0.91119147 | 1.0278621 | down | hsa_circRNA_031655 | hsa_circ_0031655 | circBase | chr14 | - | 35564253 | 35568590 | exonic | NM_017917 | PPP2R3C | AGACCGTGTTTTCCAGGAGTGTCTCACTTATGATGGAGAAATGGATTTAGAAAACTACAT |
| ASCRP3001829 | 0.118766169 | 0.909237851 | 1.1718691 | down | hsa_circRNA_077032 | hsa_circ_0077032 | circBase | chr6 | - | 75823317 | 75875495 | exonic | NM_004370 | COL12A1 | AGACTACAAACCACAAGTTGGAGTGATTGCAGATCAACGTGGTTCTCCTCAAGATTTAGT |
| ASCRP3001830 | 0.516745493 | 0.916971445 | 1.0365186 | down | hsa_circRNA_025951 | hsa_circ_0025951 | circBase | chr12 | + | 46230371 | 46233279 | exonic | NM_152641 | ARID2 | AGACTCATGTAGCATCTGCCCCAGTTTTGGAAAGACATCGTTGATGATAATGAAGTTCGT |
| ASCRP3001837 | 0.729800857 | 0.952520716 | 1.0141396 | down | hsa_circRNA_407203 | | 25070500 | chr9 | + | 99220665 | 99220823 | sense overlapping | NM_014282 | HABP4 | AGACTTCACAGCTGAGAAGTTTCCAGATGAAAAAAGCGGACTCCTAGAAGAGGGGAGCAG |
| ASCRP3001840 | 0.638121141 | 0.929544944 | 1.02406 | down | hsa_circRNA_400850 | | 25242744 | chr11 | + | 118890859 | 118892596 | exonic | NM_016146 | TRAPPC4 | AGACTTTGCCCTCAAGAATCCATTCTATTCCTTAGAAATGCCTATCAGGCTCTTTGCCAT |
| ASCRP3001841 | 0.477889799 | 0.91119147 | 1.0344473 | down | hsa_circRNA_001030 | hsa_circ_0000333 | circBase | chr11 | + | 68229037 | 68229117 | intronic | ENST00000265636 | PPP6R3 | AGACTTTTTAATTATAGGTGAACGGGATGGGTCTTTGGATGTGTTTCCTGTTGAGGTCCT |
| ASCRP3001844 | 0.798940127 | 0.972140311 | 1.0121092 | down | hsa_circRNA_101043 | hsa_circ_0025887 | circBase | chr12 | + | 40696590 | 40697936 | exonic | NM_198578 | LRRK2 | AGAGAAACTGCATCTTTCTCACAATAAACTGAAAGAGCTGCTATGCCTTTCTTGCCTCCT |
| ASCRP3001846 | 0.257147453 | 0.909237851 | 1.0275223 | down | hsa_circRNA_047040 | hsa_circ_0047040 | circBase | chr18 | + | 13387339 | 13438383 | exonic | NM_181481 | LDLRAD4 | AGAGAACTGTCTCCTGGTGACCGAGCACCCGCCTCCGGGCATCTTCAACTTTTCAGAGGC |
| ASCRP3001852 | 0.640399717 | 0.929544944 | 1.0544749 | down | hsa_circRNA_101470 | hsa_circ_0034326 | circBase | chr15 | - | 31947298 | 31981684 | exonic | uc001zfs.1 | OTUD7A | AGAGACCTGCTGGAAGAGTTCTCAGGCGCTGTGGTCTCACCTTCCACTGGGGCAACAATG |
| ASCRP3001856 | 0.673004746 | 0.938080316 | 1.010581 | down | hsa_circRNA_404459 | | 25070500 | chr1 | + | 26594973 | 26596105 | exonic | NM_022778 | CEP85 | AGAGAGAAGCAGAATTCTCCTCCGCTGGACATAGGTCAAAGGTCGTGATAAACATATCAA |
| ASCRP3001864 | 0.962884188 | 0.99396088 | 1.0017184 | down | hsa_circRNA_103465 | hsa_circ_0067231 | circBase | chr3 | - | 128356641 | 128363826 | exonic | NM_002950 | RPN1 | AGAGATGTGCCTGCCTATAGTCAGGTAAAGGGAGAAGATGAGGAAGAGAACAATTTGGAA |
| ASCRP3001870 | 0.606022018 | 0.929544944 | 1.0134026 | down | hsa_circRNA_407284 | | 25070500 | chrX | + | 47028897 | 47030400 | intronic | ENST00000329236 | RBM10 | AGAGCCAGCGGCCAGCTCCTAGGCCTGCCCAGACTGACTGCCCGTCTGGTGTGAGGAGGG |
| ASCRP3001873 | 0.392439264 | 0.91119147 | 1.0237538 | down | hsa_circRNA_000519 | hsa_circ_0000262 | circBase | chr10 | - | 121048086 | 121048266 | antisense | NM_005308 | GRK5 | AGAGCCCACCATGTTGCTGCAGCAGTGACACAGTGAGGTACAGCAGAGATGAGGAAGAGC |
| ASCRP3001877 | 0.87772412 | 0.979863126 | 1.0312343 | down | hsa_circRNA_083776 | hsa_circ_0083776 | circBase | chr8 | + | 27514298 | 27530537 | exonic | NM_016240 | SCARA3 | AGAGCTATTTCCAGGAGTTTTTCAGAAAAGTGGACTCTCTCTCCGAAGACATCTCCTTGA |
| ASCRP3001878 | 0.071740382 | 0.909237851 | 1.0940452 | down | hsa_circRNA_026787 | hsa_circ_0026787 | circBase | chr12 | - | 56182858 | 56189860 | exonic | NM_033082 | SARNP | AGAGCTCAAAGATTTGGTTTGAATGTCTCTTCAATCTCCAGAAAGGGCAGCAGAGAAGAA |
| ASCRP3001880 | 0.974286052 | 0.996425773 | 1.0012398 | down | hsa_circRNA_406796 | | 25070500 | chr6 | + | 74175931 | 74202075 | exonic | NM_012123 | MTO1 | AGAGCTGGCTGAAAGACTGAAAATAGAAGGTCAGATGTCATGTAATCCTTCCTTTGGTGG |
| ASCRP3001885 | 0.154388549 | 0.909237851 | 1.0418307 | down | hsa_circRNA_101643 | hsa_circ_0036750 | circBase | chr15 | - | 90446447 | 90454051 | exonic | NM_182616 | ARPIN | AGAGGACGAGGAGTGGGGGAAATGGTGTCCTGCTGGAGGGAGAACTGATCGATGTATCTC |
| ASCRP3001886 | 0.973045243 | 0.996425773 | 1.0011722 | down | hsa_circRNA_007344 | hsa_circ_0007344 | circBase | chr2 | - | 10740978 | 10747437 | exonic | NM_024894 | NOL10 | AGAGGAGAAGCAGAAATCTACATGGAAAAAGAAAGTTAAGGTTTTGGGTCCTGCTCCTCG |
| ASCRP3001893 | 0.324742052 | 0.909237851 | 1.0389703 | down | hsa_circRNA_104148 | hsa_circ_0008236 | circBase | chr6 | - | 83667030 | 83754378 | exonic | NM_198920 | UBE3D | AGAGTAATGCCAATCTGCCTTCATCCCTTCGCCGTGTGAATTCCTTTCAGGAAGCTCCTC |
| ASCRP3001898 | 0.569334977 | 0.92124149 | 1.0220858 | down | hsa_circRNA_406315 | | 25070500 | chr3 | - | 101371339 | 101384652 | exonic | NM_014415 | ZBTB11 | AGAGTATAGGAGACACATGAACAACCATGAAGGTTTTTGTAAGGCCAGCTTCCTTCCTTT |
| ASCRP3001911 | 0.641792747 | 0.929581513 | 1.0277719 | down | hsa_circRNA_404910 | | 25070500 | chr11 | + | 76234198 | 76239510 | exonic | NM_020193 | C11orf30 | AGATAGTAGTTCCTCTTCTACAGAGTCCTCCCAGAGTTCCCAAGGAACGACTACCAAAAT |
| ASCRP3001914 | 0.532648699 | 0.919549314 | 1.0251913 | down | hsa_circRNA_101243 | hsa_circ_0029855 | circBase | chr13 | + | 28834583 | 28835595 | exonic | NM_175854 | PAN3 | AGATATGCCAGGTGTTTCCAAACTATCATATTTATCCTCCAACTGCACCTCACGTTGCTT |
| ASCRP3001931 | 0.040152699 | 0.909237851 | 1.0612094 | down | hsa_circRNA_001738 | hsa_circ_0001738 | circBase | chr7 | - | 107002477 | 107053077 | exonic | NM_006348 | COG5 | AGATCCTGTTTCTCACATTTGTTTCATTGAAGAAATAGTTAAGATTATCTTTCTCAAGGA |
| ASCRP3001932 | 0.207870607 | 0.909237851 | 1.0534998 | down | hsa_circRNA_103000 | hsa_circ_0006704 | circBase | chr20 | - | 17933230 | 17934761 | exonic | NM_014426 | SNX5 | AGATCGCAACTTTCATGTTTTCCTGGAATATGATCAGGATATTCCACCTGCTCCTACGAA |
| ASCRP3001934 | 0.927710665 | 0.985498678 | 1.0131076 | down | hsa_circRNA_002919 | hsa_circ_0002919 | circBase | chr5 | + | 115202366 | 115205825 | exonic | NM_001284 | AP3S1 | AGATCTAATTCAAAGTGAAGATACACAACAGCAAATCATCAGGGAGACTTTCCATTTGGT |
| ASCRP3001937 | 0.172594242 | 0.909237851 | 1.057664 | down | hsa_circRNA_405133 | | 25070500 | chr13 | + | 36939636 | 36943737 | sense overlapping | NR_045180 | SPG20-AS1 | AGATCTCTCTGCCCAGGCTTGGAAATACATAAGGATCAGATTACTAGGAAAGCCAAGTCT |
| ASCRP3001943 | 0.813166489 | 0.97589665 | 1.0092749 | down | hsa_circRNA_043415 | hsa_circ_0043415 | circBase | chr17 | - | 37571278 | 37576171 | exonic | NM_004774 | MED1 | AGATGACTTCATTGCCAAAGTTGTTCAAAGGCTCTTCCTGGTCAGCAGCACTGCTATTTC |
| ASCRP3001945 | 0.164634722 | 0.909237851 | 1.0668916 | down | hsa_circRNA_013573 | hsa_circ_0013573 | circBase | chr1 | + | 113633939 | 113662145 | exonic | NM_014813 | LRIG2 | AGATGAGCGGTGGGATTTCAGTCATAATCGGTTGTCTAACTGGAACATCAGCTTGGAATC |
| ASCRP3001947 | 0.329581622 | 0.909237851 | 1.0356179 | down | hsa_circRNA_001805 | hsa_circ_0001659 | circBase | chr6 | + | 157431605 | 157435966 | intronic | ENST00000414678 | ARID1B | AGATGATGGAGAGTGAGAGATGATGCAACCTACTTCTCTCTAGAGGTAGTCAGATGCCTC |
| ASCRP3001963 | 0.621111343 | 0.929544944 | 1.0133838 | down | hsa_circRNA_100848 | hsa_circ_0022812 | circBase | chr11 | + | 65055186 | 65063461 | exonic | NM_002689 | POLA2 | AGATGTCCTCATCATCCCGTCAGAGCTGAGGTACTTCGTGAAGACTTTGAGCAAAGCATG |
| ASCRP3001981 | 0.081941633 | 0.909237851 | 1.0435865 | down | hsa_circRNA_404711 | | 25070500 | chr10 | + | 4872866 | 4951798 | sense overlapping | NR_073125 | AKR1E2 | AGCAAAAATTCTGCATCATTTTGGTGGCGTGTACAGGGACATGAGGAAGGGCTTCTCCAG |
| ASCRP3001989 | 0.504245872 | 0.914489113 | 1.0787313 | down | hsa_circRNA_049948 | hsa_circ_0049948 | circBase | chr19 | + | 17311447 | 17313692 | exonic | NM_004145 | MYO9B | AGCAAAGCTCAGAAGAAGAAGCGGAAGCAGGAGCGTGCTCCCCCTCCGGACAGCAGCATC |
| ASCRP3001990 | 0.583381968 | 0.926021592 | 1.0250452 | down | hsa_circRNA_406133 | | 25070500 | chr21 | - | 27374748 | 27376050 | intronic | ENST00000346798 | APP | AGCAAAGTGAAGCTGTAGATGTTTGTTACAACTGAAGGAGAGGGGTTGCATGTTAAGAGA |
| ASCRP3002001 | 0.194583851 | 0.909237851 | 1.1537352 | down | hsa_circRNA_101287 | hsa_circ_0008274 | circBase | chr13 | - | 96485180 | 96489456 | exonic | NM_020121 | UGGT2 | AGCAAGATACAAGATCTCCCCAATAATATGATTTACCAAGTCGCCATTAAGTCTCTTCCT |
| ASCRP3002002 | 0.48600551 | 0.91119147 | 1.017924 | down | hsa_circRNA_007899 | hsa_circ_0007899 | circBase | chr12 | + | 6630963 | 6631203 | exonic | NM_014865 | NCAPD2 | AGCAAGATGATGTATGAAAACACAACTACAGGCCCAGCAGCTTCCACACAAGAAAAGAAT |
| ASCRP3002019 | 0.430744622 | 0.91119147 | 1.0158812 | down | hsa_circRNA_002117 | hsa_circ_0000332 | circBase | chr11 | - | 66626276 | 66626387 | intronic | ENST00000393955 | PC | AGCACCCGCAGTTCTACTCGGGCTGTGGCCTCACCAGCAGGGTTGGCGGCGATGTCCGAG |
| ASCRP3002027 | 0.709625895 | 0.945045213 | 1.0196687 | down | hsa_circRNA_059354 | hsa_circ_0059354 | circBase | chr20 | - | 4760668 | 4766974 | exonic | NM_170774 | RASSF2 | AGCACTTTTCAAAGAGAGTGGCCCAGTATATAAAGTTCGAGATGCCGGTACTTAAAAGCT |
| ASCRP3002028 | 0.230703891 | 0.909237851 | 1.0562673 | down | hsa_circRNA_100050 | hsa_circ_0009759 | circBase | chr1 | - | 11130956 | 11137005 | exonic | NM_002685 | EXOSC10 | AGCAGAACAGGCCATCTCCGTCCGACAGCAGGTCGTGGATCTGTGCCAGTTCAGAAGCAG |
| ASCRP3002036 | 0.416857785 | 0.91119147 | 1.0252349 | down | hsa_circRNA_102900 | hsa_circ_0057953 | circBase | chr2 | + | 208432186 | 208442379 | exonic | NM_004379 | CREB1 | AGCAGCACGAAAGAGAGAGGTCCGTCTAATGAAGAACAGATTTCAACTATTGCAGAAAGT |
| ASCRP3002051 | 0.862082679 | 0.979863126 | 1.0082748 | down | hsa_circRNA_100874 | hsa_circ_0003376 | circBase | chr11 | + | 70251370 | 70253697 | exonic | NM_005231 | CTTN | AGCATATCAAACGGAATCAGTCCCCAATGCCTGGAAATTCCTCATTGGATTACTGTGTTT |
| ASCRP3002072 | 0.08105963 | 0.909237851 | 1.078008 | down | hsa_circRNA_406752 | | 25070500 | chr6 | + | 31592304 | 31592917 | intronic | ENST00000376007 | PRRC2A | AGCCAGAGTCTTCCACTTTTATAGCATGTCCTCAGGAAATGTCTTCTTGCAGCACTTAAT |
| ASCRP3002075 | 0.793612585 | 0.968582289 | 1.0134475 | down | hsa_circRNA_405243 | | 25070500 | chr14 | + | 62462593 | 62463260 | exonic | uc010tsd.1 | SYT16 | AGCCAGCTCCCTGGTACTTTAGAAACTGTTAATGGAAAAAAGCAAGCTGGAAGTTTTGAG |
| ASCRP3002078 | 0.008538766 | 0.882339103 | 1.0798134 | down | hsa_circRNA_005839 | hsa_circ_0005839 | circBase | chr5 | + | 68400457 | 68410323 | exonic | NM_022902 | SLC30A5 | AGCCATTGCCTTCTTAGGTGTGGCAGATCACAAGTGGATCAAAATATTTAAACATGCAGT |
| ASCRP3002081 | 0.287234224 | 0.909237851 | 1.0893898 | down | hsa_circRNA_402563 | | 25242744 | chr20 | - | 32681480 | 32686439 | exonic | NM_003908 | EIF2S2 | AGCCCAAACATCTCCTTGCATTTTTGTTGGCTGAATTGGGTACAAGGATCTTAAGATTGA |
| ASCRP3002083 | 0.230703528 | 0.909237851 | 1.0447779 | down | hsa_circRNA_043368 | hsa_circ_0043368 | circBase | chr17 | + | 36912135 | 36920478 | exonic | uc002hqr.3 | PSMB3 | AGCCCACTTTTTTTTCTTTTTTTGAAATAAAATAGCCTGTCTTTCATGCCCAGCGCCTCA |
| ASCRP3002086 | 0.11300887 | 0.909237851 | 1.06988 | down | hsa_circRNA_100564 | hsa_circ_0017953 | circBase | chr10 | + | 22002700 | 22002879 | exonic | NM_004641 | MLLT10 | AGCCCCTGCTGTTGCTACAACTCAGGTATTTATAACAGCAATGATGTAGCAGTATCGTTT |
| ASCRP3002090 | 0.168329116 | 0.909237851 | 1.0524876 | down | hsa_circRNA_403788 | | 25242744 | chr7 | - | 26883648 | 26894509 | exonic | NM_003930 | SKAP2 | AGCCTCAGAACGATATGATAAAGACGATGAAGCCCCCTCTGATGATGTTGAAACATTTGT |
| ASCRP3002093 | 0.05761051 | 0.909237851 | 1.0743898 | down | hsa_circRNA_001831 | hsa_circ_0001831 | circBase | chr8 | + | 144894444 | 144895014 | antisense | NM_015356 | SCRIB | AGCCTCCGCAGCAGGTTCTCCGTGAGGATCAGCTCAGAGAGGTTCTCACAGTCCCCGATG |
| ASCRP3002094 | 0.789599385 | 0.967199246 | 1.0069341 | down | hsa_circRNA_104756 | hsa_circ_0086694 | circBase | chr9 | - | 33932559 | 33933626 | exonic | NM_018449 | UBAP2 | AGCCTCTCTGCACATGCAGCCCTCTCCTCGAGCACGTCACACACACTCCAAAGACAACAG |
| ASCRP3002110 | 0.864247996 | 0.979863126 | 1.0091319 | down | hsa_circRNA_018315 | hsa_circ_0018315 | circBase | chr10 | + | 49609654 | 49613024 | exonic | NM_002750 | MAPK8 | AGCGGGCCTACAGAGAGCTAGTTCTTATGAAATGTGTTAATCACAAAAATCTTCTTGGTG |
| ASCRP3002112 | 0.297077009 | 0.909237851 | 1.0441182 | down | hsa_circRNA_005152 | hsa_circ_0005152 | circBase | chr16 | - | 9009110 | 9011013 | exonic | NM_003470 | USP7 | AGCTAAGTATCAAAGGAAAGAAAAATAGCTGTGTACATGATGCCAACCGAGGGGGATGAT |
| ASCRP3002118 | 0.264435316 | 0.909237851 | 1.0582427 | down | hsa_circRNA_406707 | | 25070500 | chr5 | + | 162890861 | 162891808 | exonic | NM_012484 | HMMR | AGCTAGAAAAGTTAAGTCTTCGGAATCAAAGGTTGTGCACCATCTCCAGGTGCTTATGAT |
| ASCRP3002129 | 0.482146181 | 0.91119147 | 1.039328 | down | hsa_circRNA_050374 | hsa_circ_0050374 | circBase | chr19 | - | 33106563 | 33117733 | exonic | NM_032139 | ANKRD27 | AGCTCAGAAGGGCAGGCATCCCTCATCGACCTCCTGGTTTCCAAGGGCGCCATGGTAAAT |
| ASCRP3002139 | 0.39154288 | 0.91119147 | 1.0417154 | down | hsa_circRNA_101995 | hsa_circ_0042415 | circBase | chr17 | - | 19835117 | 19866335 | exonic | NM_007202 | AKAP10 | AGCTCTGCGTCTCAGCTTCAATATCCGTACATTCCCCACAAAAAAGCACTAAAAATCATG |
| ASCRP3002146 | 0.542897831 | 0.919549314 | 1.0278932 | down | hsa_circRNA_400296 | | 25242744 | chr1 | - | 109952549 | 109964548 | exonic | NM_002790 | PSMA5 | AGCTGAATGCAACAAACATTGAGGGGCGTGAATACTTTTTCTCCCGAAGGAAGATTATTT |
| ASCRP3002165 | 0.463577302 | 0.91119147 | 1.0373014 | down | hsa_circRNA_103182 | hsa_circ_0003720 | circBase | chr22 | + | 24730377 | 24734445 | exonic | NM_015330 | SPECC1L | AGCTGTGTCCCCTATGCAGGCAAGAGGAGGAGCGAGGCCGGGTATACAATTACATGAATG |
| ASCRP3002176 | 0.472893962 | 0.91119147 | 1.0446911 | down | hsa_circRNA_000585 | hsa_circ_0000515 | circBase | chr14 | - | 20811305 | 20811534 | sense overlapping | NR_002312 | RPPH1 | AGCTTGGAACAGACTCACGGCCAGCGAACTGAGTGCGTCCTGTCACTCCACTCCCATGTC |
| ASCRP3002179 | 0.405083247 | 0.91119147 | 1.1903789 | down | hsa_circRNA_008068 | hsa_circ_0008068 | circBase | chr13 | - | 30801548 | 30857928 | exonic | NM_032116 | KATNAL1 | AGCTTTGCGAAGAAGGTTAGAAAAAAGGATATATATACCTCTCCCAACAGGTCTCTGAAA |
| ASCRP3002182 | 0.16435183 | 0.909237851 | 1.0449119 | down | hsa_circRNA_104548 | hsa_circ_0001777 | circBase | chr7 | - | 158580694 | 158591763 | exonic | NM_020728 | ESYT2 | AGCTTTGTCTATCTTCTTCCTTAGGAAACCAGTTCATTTTCCAGACACTGAAAGAGCAGA |
| ASCRP3002205 | 0.632821518 | 0.929544944 | 1.0156056 | down | hsa_circRNA_406690 | | 25070500 | chr5 | + | 143586380 | 143586631 | sense overlapping | NM_020768 | KCTD16 | AGGAAGACTGAAAAGGGAAGCTGAATACTTCCAGCTCCCAGACTTGTTTATTTTACTCGC |
| ASCRP3002208 | 0.382764353 | 0.91119147 | 1.0297713 | down | hsa_circRNA_404628 | | 25070500 | chr1 | + | 186375214 | 186381025 | exonic | ENST00000478571 | C1orf27 | AGGAAGCAAAACAGCAATTTGGAAAGTTTAGATTCTGAAAAAGAGTTCCACGTCCTCCCT |
| ASCRP3002210 | 0.370286022 | 0.91119147 | 1.0374016 | down | hsa_circRNA_102140 | hsa_circ_0044927 | circBase | chr17 | - | 58275620 | 58292135 | exonic | NM_032582 | USP32 | AGGAAGCTAGTAATCATGCCCAGGATTGGAAATGTACCTTCTCCGAATGCACCTTTAAAG |
| ASCRP3002239 | 0.922081677 | 0.9834955 | 1.0035252 | down | hsa_circRNA_102289 | hsa_circ_0046760 | circBase | chr18 | - | 6237962 | 6312055 | exonic | NM_173464 | L3MBTL4 | AGGAGAATGGAAGAACTCTGATAGCACCCCAAGATTGCTATGCTATGCCTTGTATGACTA |
| ASCRP3002260 | 0.605217994 | 0.929544944 | 1.0437346 | down | hsa_circRNA_103639 | hsa_circ_0007646 | circBase | chr4 | + | 52729602 | 52758017 | exonic | NM_015115 | DCUN1D4 | AGGAGTGGTTAAAAGGAATGACTTCTCTCCAATTTTCAGCTGAACTCTCATCTCTCAACA |
| ASCRP3002273 | 0.015392576 | 0.909237851 | 1.0919827 | down | hsa_circRNA_103349 | hsa_circ_0065217 | circBase | chr3 | - | 47468646 | 47470160 | exonic | NM_012235 | SCAP | AGGATGGTCTCCTACACCATCACCCTGGTCTTCCAGCACTACCATGCCAATATGTGGGTG |
| ASCRP3002278 | 0.299673204 | 0.909237851 | 1.0305034 | down | hsa_circRNA_406237 | | 25070500 | chr3 | + | 16336362 | 16345099 | exonic | uc003cax.3 | OXNAD1 | AGGCAGAAAAAATAAAGAGGGTTGATTTCTTTATTCCAGGAGTCTCTGTGGTTGGTGGGT |
| ASCRP3002288 | 0.179811647 | 0.909237851 | 1.0976783 | down | hsa_circRNA_001357 | hsa_circ_0001357 | circBase | chr3 | - | 167217940 | 167254788 | exonic | NM_178824 | WDR49 | AGGCCCCGTCACTTTTTAAAAGGCTATTACTGTGTTTCGACCCCAAAACTTCAATCAATT |
| ASCRP3002301 | 0.432588605 | 0.91119147 | 1.0585346 | down | hsa_circRNA_063681 | hsa_circ_0063681 | circBase | chr22 | - | 43455389 | 43460330 | exonic | NM_012263 | TTLL1 | AGGCTGTGGCGGTTTGTGTCTCAATCTAATAAGGAAGCCTACGTGATCTCTCTCTATATT |
| ASCRP3002320 | 0.02338236 | 0.909237851 | 1.0566642 | down | hsa_circRNA_100219 | hsa_circ_0004619 | circBase | chr1 | - | 51121113 | 51210447 | exonic | NM_007051 | FAF1 | AGGGCTGGCCAACTTCTGCTACAGACGACTCAGAGAGATTAAACAGATTCTAGAAAATGA |
| ASCRP3002326 | 0.269401125 | 0.909237851 | 1.0249586 | down | hsa_circRNA_101795 | hsa_circ_0000688 | circBase | chr16 | + | 29845053 | 29845387 | exonic | NM_005115 | MVP | AGGGTGACAGGACATCACACCCCTGCAGGTGGTTCTGCCCAACACTGCCCTCCATCTAAA |
| ASCRP3002336 | 0.703819761 | 0.944417633 | 1.0255881 | down | hsa_circRNA_104040 | hsa_circ_0075410 | circBase | chr6 | + | 304627 | 311962 | exonic | NM_020185 | DUSP22 | AGGTGACACATATTCTGTCTGTCCACGATAGTGCCAGGCCTATGTTGGAGATCCTGCCCG |
| ASCRP3002337 | 0.825277194 | 0.97720472 | 1.0066381 | down | hsa_circRNA_103879 | hsa_circ_0072857 | circBase | chr5 | - | 70294550 | 70297970 | exonic | NM_004536 | NAIP | AGGTGATGACCCATTAGACGATCACACCAGATGTTTTCCCAAGGAGAACATTTTGTGAAT |
| ASCRP3002341 | 0.950700117 | 0.992077624 | 1.0035197 | down | hsa_circRNA_089954 | hsa_circ_0089954 | circBase | chrX | - | 16716358 | 16721064 | exonic | NM_019857 | CTPS2 | AGGTGCCGGTGGATGGTAATAAGGAAGAGCCCCAAATATGCGTTATTGAGATTATATCTG |
| ASCRP3002342 | 0.217085113 | 0.909237851 | 1.0563281 | down | hsa_circRNA_103330 | hsa_circ_0004194 | circBase | chr3 | + | 41265511 | 41268843 | exonic | NM_001904 | CTNNB1 | AGGTGCTATCTGTCTGCTCTAGTAATAAGCCGGCTATTGTAGAAGCTGGGTATTTGAAGT |
| ASCRP3002370 | 0.424032753 | 0.91119147 | 1.0831264 | down | hsa_circRNA_025501 | hsa_circ_0025501 | circBase | chr12 | + | 12870301 | 12875305 | exonic | uc001rat.2 | CDKN1B | AGTAATCAATAAAGAAAACTTCCATAGCTATTCTTCTTCGTCAGCCTCCCTTCCACCGCC |
| ASCRP3002379 | 0.154381118 | 0.909237851 | 1.0562422 | down | hsa_circRNA_104715 | hsa_circ_0085794 | circBase | chr8 | - | 142225857 | 142231864 | exonic | NM_001080431 | SLC45A4 | AGTACCATGACATCAAGCAGGCCTTCCGGAGCAGTACTACAGCCTCACCTGGTTCCTGAG |
| ASCRP3002383 | 0.444840822 | 0.91119147 | 1.0267327 | down | hsa_circRNA_035208 | hsa_circ_0035208 | circBase | chr15 | - | 50596162 | 50596330 | exonic | NM_002041 | GABPB1 | AGTAGAGGTTTTACTTAAGCTGGGAACTTCTCCACTTCATCTAGCAGCACAGTATGGTCA |
| ASCRP3002385 | 0.300214975 | 0.909237851 | 1.0414773 | down | hsa_circRNA_007273 | hsa_circ_0007273 | circBase | chr2 | - | 73315177 | 73316443 | exonic | NM_015470 | RAB11FIP5 | AGTAGCTGGTTTGGCTTGAGAGAAGCCAAGGACCCGACTCAGAAACCCAGGTGGTACAAG |
| ASCRP3002397 | 0.746259744 | 0.95565436 | 1.0140291 | down | hsa_circRNA_103999 | hsa_circ_0074854 | circBase | chr5 | + | 162940560 | 162944680 | exonic | NM_013283 | MAT2B | AGTCACTTAAGACCTCCCCATGTTATAGTACATTGTGCAGCAGAGAGAAGACCAGATGTT |
| ASCRP3002402 | 0.395066693 | 0.91119147 | 1.064342 | down | hsa_circRNA_064277 | hsa_circ_0064277 | circBase | chr3 | - | 10342614 | 10359778 | exonic | uc003bvm.3 | SEC13 | AGTCATATTGCTTATAAGTGTCAGTAATTAACACTGTGGATACCTCCCATGAGGACATGA |
| ASCRP3002408 | 0.133897122 | 0.909237851 | 1.1038015 | down | hsa_circRNA_103905 | hsa_circ_0008226 | circBase | chr5 | + | 80388619 | 80422982 | exonic | NM_006909 | RASGRF2 | AGTCCACCAGGTTCTCTTATTCAAGTACCTTCCGTTGAGAGGGGGAAACTTAGTAAAGTT |
| ASCRP3002410 | 0.453078288 | 0.91119147 | 1.0704422 | down | hsa_circRNA_100375 | hsa_circ_0006758 | circBase | chr1 | + | 165859440 | 165860559 | exonic | NM_012474 | UCK2 | AGTCCAGATCCCCGTGTATGACTTTGTCTCCCATTCCCGTCTTCCGTGTGTGCTAAGATC |
| ASCRP3002413 | 0.907050459 | 0.982324123 | 1.0038938 | down | hsa_circRNA_103175 | hsa_circ_0001211 | circBase | chr22 | + | 23615267 | 23615961 | exonic | NM_004327 | BCR | AGTCCATGACGGTGAAGAAGGGAGAGCTCTGCTCTACAAGCCTGTGGACCGTGTGACGAG |
| ASCRP3002414 | 0.170912877 | 0.909237851 | 1.0238524 | down | hsa_circRNA_001176 | hsa_circ_0001176 | circBase | chr20 | - | 62197441 | 62197777 | sense overlapping | NM_033405 | HELZ2 | AGTCCCAGCTGGTGGTAGCGGGACCTAGGCCAGGTGTCTGTCGGCAGTTTTGAGATCCTG |
| ASCRP3002415 | 0.109537162 | 0.909237851 | 1.0385631 | down | hsa_circRNA_407094 | | 25070500 | chr8 | - | 90659967 | 90660841 | intronic | ENST00000519655 | RP11-37B2.1 | AGTCCCCAGCAAAGCCCTAAAATAATGAATTTATTTTCTGGTAGAGGAGAACGTCGCAGA |
| ASCRP3002420 | 0.958354992 | 0.993354918 | 1.0005284 | down | hsa_circRNA_406464 | | 25070500 | chr4 | - | 38828305 | 38831158 | intronic | ENST00000506146 | TLR1 | AGTCCTCGGAGTGCCCTGTGAATCATATTGGTCTTTGGAATTTGGACTCATATCAAGATG |
| ASCRP3002422 | 0.160437759 | 0.909237851 | 1.1361262 | down | hsa_circRNA_405483 | | 25070500 | chr16 | - | 70299035 | 70299440 | intronic | ENST00000261772 | AARS | AGTCCTTTGGAGTCCCTTTTCTCCTACTTTACGGTAAAGAACTTGGAGAGTGAACAGGAA |
| ASCRP3002427 | 0.319365848 | 0.909237851 | 1.0900762 | down | hsa_circRNA_002032 | hsa_circ_0002032 | circBase | chr1 | - | 63042932 | 63052295 | exonic | NM_033407 | DOCK7 | AGTCTCATTGGAAAAACCACCACAGGCTTATTCTGTACTGTCTCCTGAGAAATCTTCTCT |
| ASCRP3002433 | 0.719968212 | 0.949318546 | 1.0199906 | down | hsa_circRNA_102205 | hsa_circ_0045862 | circBase | chr17 | + | 76082583 | 76083174 | exonic | NM_018996 | TNRC6C | AGTCTTCACCCAACACCTTTGCTCCTTACCCTCTCGGCATACCAACGTTTACAAATCCAG |
| ASCRP3002434 | 0.239778996 | 0.909237851 | 1.1196638 | down | hsa_circRNA_403250 | | 25242744 | chr4 | + | 152487289 | 152510078 | exonic | NM_001109977 | FAM160A1 | AGTCTTGGCTCCTGCTCTCCATAAGGTTTTGGAAGGTGACAATGAAATGTGAAGAAGTTA |
| ASCRP3002443 | 0.165734824 | 0.909237851 | 1.1161843 | down | hsa_circRNA_017969 | hsa_circ_0017969 | circBase | chr10 | - | 22856779 | 22862386 | exonic | NM_005028 | PIP4K2A | AGTGACAAAGAAAAGTACATAGTGGAATGTCATGGGATCACCCTTCTTCCCCAGTTCTTG |
| ASCRP3002444 | 0.753563857 | 0.957534606 | 1.010754 | down | hsa_circRNA_103238 | hsa_circ_0063534 | circBase | chr22 | - | 41664100 | 41677086 | exonic | NM_002883 | RANGAP1 | AGTGACATGTTCACGGGAAGGCTGCGGACCGAGATCCCACCAGCCCTGTCTGCAGATCTC |
| ASCRP3002454 | 0.152530144 | 0.909237851 | 1.0530543 | down | hsa_circRNA_102767 | hsa_circ_0055323 | circBase | chr2 | - | 74655974 | 74656069 | exonic | NM_033046 | RTKN | AGTGCAAGATGGATTCCGCACACATGACCTCACCCTTGCCAGTCATGTGGTCCTCGTTAC |
| ASCRP3002465 | 0.080388913 | 0.909237851 | 1.066308 | down | hsa_circRNA_002132 | hsa_circ_0002132 | circBase | chr6 | - | 56716194 | 56765419 | exonic | NM_001144769 | DST | AGTGCTTCGGATAGCAGGGATTCAGAGCAAGCCCTCGGCATCTTAGAAGACGAGTTGCAG |
| ASCRP3002467 | 0.00269879 | 0.870215632 | 1.0686751 | down | hsa_circRNA_404954 | | 25070500 | chr12 | - | 2790819 | 2791778 | intronic | ENST00000501371 | CACNA1C-AS1 | AGTGGAAGGAAAGATGAGAGGTCTCATCCTGAGACGTCTCCTCCTGACCCGCCATGGCTG |
| ASCRP3002480 | 0.373100567 | 0.91119147 | 1.0221721 | down | hsa_circRNA_007854 | hsa_circ_0007854 | circBase | chr3 | - | 169840378 | 169843795 | sense overlapping | NM_024947 | PHC3 | AGTGGGGAGAGGAGAAGATTTGACTTCTGAACATCCTTTGTTAGTTTATTTAGGAAAGGG |
| ASCRP3002484 | 0.061282502 | 0.909237851 | 1.0485056 | down | hsa_circRNA_105053 | hsa_circ_0092235 | circBase | chrY | + | 14832521 | 14834120 | exonic | NM_004654 | USP9Y | AGTGTAAAAGACTTCATCACCTGATTCTTCCAATGAGAATTCCGTAGCAACTCCTCCTCC |
| ASCRP3002495 | 0.145180405 | 0.909237851 | 1.0887232 | down | hsa_circRNA_406695 | | 25070500 | chr5 | + | 145379620 | 145383716 | exonic | NM_152550 | SH3RF2 | AGTTAGGAGATAAAGTAGGCATCTTCCCTATCTTGTTTGTAGAGGTGCCTCGAGCAAAGG |
| ASCRP3002496 | 0.280997158 | 0.909237851 | 1.0329906 | down | hsa_circRNA_404595 | | 25070500 | chr1 | - | 156303463 | 156304503 | intronic | ENST00000295688 | CCT3 | AGTTAGGGCCAAGCTTCATGAGTTCAGAGGTCTTTGAATAGTGTGTGTTTTGTTTTTGGT |
| ASCRP3002500 | 0.321426068 | 0.909237851 | 1.025057 | down | hsa_circRNA_405896 | | 25070500 | chr2 | - | 61708319 | 61713097 | exonic | NM_003400 | XPO1 | AGTTCAAATAAAGGTCGCTGAAAATTTTGTTCCCCCTCTGTTGGATGCAGTTCTCATTGA |
| ASCRP3002501 | 0.638908528 | 0.929544944 | 1.0179551 | down | hsa_circRNA_003057 | hsa_circ_0003057 | circBase | chr5 | - | 14741935 | 14758707 | exonic | NM_054027 | ANKH | AGTTCACCTTCGTCTGCATGGCTCTGTCACTCACGCTTATAGTGATTTAGGATACTACAT |
| ASCRP3002507 | 0.059929923 | 0.909237851 | 1.1225452 | down | hsa_circRNA_024081 | hsa_circ_0024081 | circBase | chr11 | - | 95620775 | 95621425 | exonic | NM_001243571 | MTMR2 | AGTTCCTACACATCTCATTATAGTTTTGCCTCCACTTCTCATTCAGAGAATTCAGTGCAT |
| ASCRP3002509 | 0.419717381 | 0.91119147 | 1.0875882 | down | hsa_circRNA_051825 | hsa_circ_0051825 | circBase | chr19 | + | 49506535 | 49519182 | exonic | NM_006666 | RUVBL2 | AGTTCTGACACTGTGACTCTGTATAAAATGGTTGGGAAGCTGCGTGCCCACTCCCACATC |
| ASCRP3002510 | 0.276192179 | 0.909237851 | 1.102508 | down | hsa_circRNA_103998 | hsa_circ_0074817 | circBase | chr5 | - | 158204420 | 158223486 | exonic | NM_001290360 | EBF1 | AGTTCTGCAAAGGAACACCAGGCAGATTCATTTATACAGCAGCTACTCCCTGTATCAAAG |
| ASCRP3002514 | 0.233316258 | 0.909237851 | 1.0766478 | down | hsa_circRNA_000595 | hsa_circ_0000595 | circBase | chr15 | + | 44615162 | 44630515 | exonic | NM_138423 | CASC4 | AGTTGATGATCTTCCCCCTGGTTAAACTACAGAACAACATATCGTATCAGATGGCAGACA |
| ASCRP3002516 | 0.573667319 | 0.922626849 | 1.0818795 | down | hsa_circRNA_092372 | hsa_circ_0000166 | circBase | chr1 | + | 186375214 | 186388209 | sense overlapping | NM_017847 | C1orf27 | AGTTGCAGTCCTTGCTGCGGGTATCTCCTTTCATTACTTCAGTGATTAGGATTCTGAAAA |
| ASCRP3002519 | 0.678678375 | 0.940534337 | 1.0379202 | down | hsa_circRNA_004045 | hsa_circ_0004045 | circBase | chr18 | - | 43668074 | 43675097 | exonic | NM_004046 | ATP5A1 | AGTTGGTGAAGAGACTTACAGATGCAGGTCTCCAGAAATGCTTTGGGTTCATCTTTCATT |
| ASCRP3002520 | 0.087456621 | 0.909237851 | 1.2682813 | down | hsa_circRNA_101408 | hsa_circ_0032704 | circBase | chr14 | + | 76173360 | 76187046 | exonic | NM_015072 | TTLL5 | AGTTGTTTTGCCAAACCAGATCTCCCTGGAAGAGAACATTTTGGTCTCCCGTTACATTAA |
| ASCRP3002534 | 0.62758254 | 0.929544944 | 1.0275731 | down | hsa_circRNA_092148 | hsa_circ_0092148 | circBase | chrX | - | 154006958 | 154019343 | exonic | NM_002436 | MPP1 | ATAAAAATGCTGTGTTGAAATGCCAAGCTCCCTTCACGTGGGGGATGAGATCCTAGAAAT |
| ASCRP3002549 | 0.998897988 | 0.99972787 | 1.0000978 | down | hsa_circRNA_065384 | hsa_circ_0065384 | circBase | chr3 | + | 48491442 | 48495818 | exonic | NM_032166 | ATRIP | ATAAACTGGCTGCTCCCTCTGTTTCCCATGTCAGGTGATCATAAGGTCCACAGATTATTA |
| ASCRP3002564 | 0.8273238 | 0.977459664 | 1.0168964 | down | hsa_circRNA_404957 | | 25070500 | chr12 | + | 4763457 | 4794491 | exonic | NM_005002 | NDUFA9 | ATAACAAGGGATAAAGTGGAGCGGGTTCTGCCATTACTGCAATAGCCACATCTGTGTGTC |
| ASCRP3002581 | 0.110944479 | 0.909237851 | 1.1314909 | down | hsa_circRNA_020964 | hsa_circ_0020964 | circBase | chr11 | - | 5274420 | 5275744 | exonic | NM_000184 | HBG2 | ATAATAAATCTATTCTGCTAAGAGATCACGCTCCTGGTTGTCTACCCATGGACCCAGAGG |
| ASCRP3002585 | 0.692686088 | 0.943480789 | 1.0120799 | down | hsa_circRNA_103091 | hsa_circ_0060927 | circBase | chr20 | - | 52773707 | 52788209 | exonic | NM_000782 | CYP24A1 | ATAATACGCCTCAGGGAAGGGGAAGACTGGCAGCGGGTCCGGAGTGCCTTTCAAAAGAAA |
| ASCRP3002588 | 0.484964117 | 0.91119147 | 1.0496014 | down | hsa_circRNA_407100 | | 25070500 | chr8 | + | 94767247 | 94828743 | sense overlapping | NM_153704 | TMEM67 | ATAATCATGGCCAAAAAAAAGTCATGATATCAGGCCCAGACCTTCTCTTTCCCTTTCCAG |
| ASCRP3002605 | 0.88305912 | 0.979863126 | 1.0087851 | down | hsa_circRNA_400710 | | 25242744 | chr11 | - | 11348632 | 11362551 | exonic | NM_198516 | GALNT18 | ATACAGAGAATGTCCCCATCATGTACATCTGCCATGGGATGACGCCTCAGGTGTGGCAGT |
| ASCRP3002613 | 0.296094526 | 0.909237851 | 1.0440728 | down | hsa_circRNA_400959 | | 25242744 | chr12 | + | 51076920 | 51080465 | exonic | NM_173602 | DIP2B | ATACATTGAGGTAGCCCTGGTTTACCCCAACAATGATCCAGTCATGTTTATGGTGGCTTT |
| ASCRP3002618 | 0.784671315 | 0.966801146 | 1.0110198 | down | hsa_circRNA_401195 | | 25242744 | chr13 | - | 101277788 | 101322823 | exonic | NM_032813 | TMTC4 | ATACCCAGACTGTTACTACAACCTCGGGCGTCTGTTGTAATTTAGATTCGAGAAGTGGTT |
| ASCRP3002637 | 0.097090174 | 0.909237851 | 1.0900542 | down | hsa_circRNA_101746 | hsa_circ_0038349 | circBase | chr16 | + | 19627435 | 19693712 | exonic | NM_020314 | C16orf62 | ATAGACAAAGGTGGGAATGGAAGTGGCCCCACATCTCAAAGAAACCCTAAATAAGAACTT |
| ASCRP3002643 | 0.928334814 | 0.985498678 | 1.0055801 | down | hsa_circRNA_001300 | hsa_circ_0001393 | circBase | chr4 | - | 4452322 | 4452402 | intronic | ENST00000306200 | STX18 | ATAGATCAAATAATAAATACAGAAGGAGGCTGGATAGGGATGGAGAGGGGTGGTAGAGGC |
| ASCRP3002648 | 0.138455831 | 0.909237851 | 1.0729505 | down | hsa_circRNA_012287 | hsa_circ_0012287 | circBase | chr1 | + | 46463395 | 46466651 | exonic | uc001cox.1 | MAST2 | ATAGCCACAGAGCTGACAGGTAACAGTCCTTTGGACAGCCCCCGGAATTTCTCTCCAAAT |
| ASCRP3002649 | 0.156240517 | 0.909237851 | 1.1364559 | down | hsa_circRNA_104747 | hsa_circ_0086563 | circBase | chr9 | + | 20907148 | 20926416 | exonic | NM_017794 | FOCAD | ATAGCCATTACCAACCCAGAGGGCAACTTCTCTCCTGGTTTTATTATGTTCATATCCAGC |
| ASCRP3002650 | 0.804729917 | 0.974037816 | 1.007788 | down | hsa_circRNA_102879 | hsa_circ_0057552 | circBase | chr2 | + | 196544755 | 196583056 | exonic | NM_020342 | SLC39A10 | ATAGCCGTCTTCTGTCATGAACTGCCACATGAATTAGGAAAAATAGAAATGAAGGTACAT |
| ASCRP3002653 | 0.122028436 | 0.909237851 | 1.070453 | down | hsa_circRNA_001208 | hsa_circ_0001208 | circBase | chr22 | - | 22055264 | 22065197 | sense overlapping | NM_013313 | YPEL1 | ATAGCTCTTGACACTCTCGGCCATCTCTGTGGGTTACGTTTTTCTCTCGTCCCAGCTGTG |
| ASCRP3002657 | 0.722586904 | 0.949785489 | 1.0090952 | down | hsa_circRNA_001656 | hsa_circ_0001656 | circBase | chr6 | - | 157150420 | 157150544 | antisense | NM_017519 | ARID1B | ATAGGAACCTCCTGGGTACGGACTGCTCTGCTGAGGCTGAGAATGAGGGGTACTGCATTC |
| ASCRP3002660 | 0.231450398 | 0.909237851 | 1.0468153 | down | hsa_circRNA_406125 | | 25070500 | chr20 | - | 50516660 | 50530353 | antisense | ENST00000516658 | RNU6-347P | ATAGGGATGGAGAAGGAAGCAGATGCCCTACAAAGCCCATGTATAGTCACCCAACAAAAT |
| ASCRP3002664 | 0.892331928 | 0.979863126 | 1.004193 | down | hsa_circRNA_001229 | hsa_circ_0001229 | circBase | chr22 | + | 38089338 | 38089486 | sense overlapping | NM_024313 | NOL12 | ATAGTGTTTACCAGTATTAAATTCTCAGCCACTCCTTTCCATGTGTGCCTACCGCATGTT |
| ASCRP3002679 | 0.671045284 | 0.938080316 | 1.008751 | down | hsa_circRNA_404849 | | 25070500 | chr11 | + | 12462922 | 12465283 | intronic | ENST00000334956 | PARVA | ATATCAGATGGGAACATGAGGTGCTTGGATGGAAACAGATCACTCTCCAAGTGAGATCAT |
| ASCRP3002688 | 0.470146684 | 0.91119147 | 1.0458501 | down | hsa_circRNA_407270 | | 25070500 | chrX | + | 15969938 | 16026822 | intergenic |  |  | ATATCTCCACTCCTCAAATTGAAGTTCAGTATCTGCTGAACCTCTTACTCTCCTGGAGCC |
| ASCRP3002691 | 0.620270892 | 0.929544944 | 1.0613635 | down | hsa_circRNA_103490 | hsa_circ_0004565 | circBase | chr3 | + | 149563797 | 149619949 | exonic | NM_007282 | RNF13 | ATATGAAAAAGGGTGATTTTACAACGAGATGCTGCTCTCCATAGGGATGCTCATGCTGTC |
| ASCRP3002693 | 0.111484302 | 0.909237851 | 1.1275863 | down | hsa_circRNA_404558 | | 25070500 | chr1 | - | 108697604 | 108724650 | exonic | NM_013386 | SLC25A24 | ATATGAACAGGAAAAATTGAGGCTTCAGAAATTGTCCAGTCTCTCCAGACACTGGGTCTG |
| ASCRP3002694 | 0.547552796 | 0.919549314 | 1.0429382 | down | hsa_circRNA_406705 | | 25070500 | chr5 | + | 159437506 | 159446038 | sense overlapping | NM_003314 | TTC1 | ATATGAAGAAGATGAAGGAACTTTGAACTTATCCAGCTTTAGCGTCACCTCCCTCACTGG |
| ASCRP3002707 | 0.543797497 | 0.919549314 | 1.0537494 | down | hsa_circRNA_402931 | | 25242744 | chr3 | + | 122525703 | 122545912 | exonic | NM_032839 | DIRC2 | ATATTAAAGATCGCATAGAGGCTGTGTTATATGCAGGTCTCCGGATAACTGTGCTCCTGA |
| ASCRP3002709 | 0.387558124 | 0.91119147 | 1.0519678 | down | hsa_circRNA_401126 | | 25242744 | chr13 | + | 26343183 | 26349097 | exonic | NM_016529 | ATP8A2 | ATATTATTGAGAGTGTCTCCTCTGCAGAAGTCTGAGATAGTGGATGTGGTGAAGAAGCGG |
| ASCRP3002719 | 0.023151352 | 0.909237851 | 1.0535938 | down | hsa_circRNA_103838 | hsa_circ_0072464 | circBase | chr5 | - | 53409031 | 53450469 | exonic | NM_019087 | ARL15 | ATATTGGCCAATCATCAAGACAAGCCAGCAGCTCGCTCAGTACAAGAGGTTTTAGTATTA |
| ASCRP3002722 | 0.253373404 | 0.909237851 | 1.0402069 | down | hsa_circRNA_103042 | hsa_circ_0005587 | circBase | chr20 | - | 34243123 | 34246936 | exonic | uc002xdn.1 | CPNE1 | ATATTGTAGGGGGTGAACTGGTCTAAAAGTTGGTTTTAATTGGTTGCCCACAGGATTGAC |
| ASCRP3002734 | 0.653277725 | 0.933189368 | 1.0214618 | down | hsa_circRNA_405289 | | 25070500 | chr14 | - | 102810157 | 102810726 | sense overlapping | uc001ylu.1 | CINP | ATCAAAGAACTGGGCTTCTACCCTGAGCAGCATCTTGCTGTGGACCTCAAGATCCCAGGA |
| ASCRP3002736 | 0.055632946 | 0.909237851 | 1.192987 | down | hsa_circRNA_104268 | hsa_circ_0078738 | circBase | chr6 | - | 170033042 | 170058454 | exonic | NM_182552 | WDR27 | ATCAAATCTGCCAAAATAAACTCCTGTGTCCTACCGACCTCTCCACTGTATCTGGGAATT |
| ASCRP3002737 | 0.734277567 | 0.952949359 | 1.0190977 | down | hsa_circRNA_406628 | | 25070500 | chr5 | + | 80160631 | 80173542 | sense overlapping | NM_002439 | MSH3 | ATCAACACTGAAGCAGGTGAAATCCTTAACCCTGTTTGTCACCCATTATCCGCCAGTTTG |
| ASCRP3002744 | 0.418809577 | 0.91119147 | 1.0207254 | down | hsa_circRNA_038343 | hsa_circ_0038343 | circBase | chr16 | + | 19627435 | 19644494 | exonic | NM_020314 | C16orf62 | ATCAAGCACATGACTCCAGATCGTGCATTTGAAGATTCCTACCCCCAGGTGGGAATGGAA |
| ASCRP3002747 | 0.715431366 | 0.948442621 | 1.018478 | down | hsa_circRNA_100675 | hsa_circ_0003839 | circBase | chr10 | + | 104119940 | 104120152 | exonic | NM_004193 | GBF1 | ATCAAGGATGAGATGTGCCGTCACTTATTCCAGGCACAGCTTTGGTCCCCTATGGTCTTC |
| ASCRP3002749 | 0.878319042 | 0.979863126 | 1.0055586 | down | hsa_circRNA_026715 | hsa_circ_0026715 | circBase | chr12 | + | 54676862 | 54679030 | exonic | NM_031157 | HNRNPA1 | ATCAATAAACGATTTAATTCTCTTGAAGTGGTTATGGAGGAGGCGGCCCTGGTTACTCTG |
| ASCRP3002765 | 0.026878585 | 0.909237851 | 1.2676815 | down | hsa_circRNA_072697 | hsa_circ_0072697 | circBase | chr5 | + | 64863339 | 64868113 | exonic | NM_015342 | PPWD1 | ATCACTAAGCTCTTAGAGTTTGAAAGAGTCTATCTTGATAATCTCCCCAGTGCATCCATG |
| ASCRP3002766 | 0.212436635 | 0.909237851 | 1.0355781 | down | hsa_circRNA_015808 | hsa_circ_0015808 | circBase | chr1 | + | 198248083 | 198266370 | exonic | NM_133494 | NEK7 | ATCACTATTCAGAAGAAATATAAAACCAGCTAATGTGTTCATTACAGCCACTGGGGTGGT |
| ASCRP3002768 | 0.231849358 | 0.909237851 | 1.0520257 | down | hsa_circRNA_100111 | hsa_circ_0007283 | circBase | chr1 | - | 27432375 | 27436268 | exonic | NM_003047 | SLC9A1 | ATCACTGTCATCTTCTTCACCGTCTTTGTGCAGGTCCTGTATCACCTCTTTGAGGAGTTT |
| ASCRP3002770 | 0.739804046 | 0.953746099 | 1.0092947 | down | hsa_circRNA_102301 | hsa_circ_0046909 | circBase | chr18 | + | 9931806 | 9950565 | exonic | NM_003574 | VAPA | ATCAGAAGAAAATCGGCACCTGAGAGCCCCTTCACAGATGTAGTCACTACAAATCTTAAA |
| ASCRP3002776 | 0.829991586 | 0.978181218 | 1.0088912 | down | hsa_circRNA_102478 | hsa_circ_0008942 | circBase | chr19 | + | 17273159 | 17278874 | exonic | NM_004145 | MYO9B | ATCAGCAAGAAACCCACGGGCCTCTTCTACCTGCTGGACGAGGAGAGCAAGCCATCACTG |
| ASCRP3002777 | 0.322368717 | 0.909237851 | 1.0879153 | down | hsa_circRNA_037798 | hsa_circ_0037798 | circBase | chr16 | - | 8946801 | 8949183 | sense overlapping | NM_014316 | CARHSP1 | ATCAGCCATCTTTCTCCCAATAAATTTTTGTTTTGTGCAAGGTGTGGAAGGGGAGTATGT |
| ASCRP3002784 | 0.807946752 | 0.974037816 | 1.0069188 | down | hsa_circRNA_102961 | hsa_circ_0058916 | circBase | chr2 | - | 240929490 | 240951113 | exonic | NM_004544 | NDUFA10 | ATCAGTTCAGAGAGCCACATGAAATGAAGATCACCTCTGCCTATCTACAGGACATTGAGA |
| ASCRP3002788 | 0.216157277 | 0.909237851 | 1.0581978 | down | hsa_circRNA_405789 | | 25070500 | chr19 | - | 44429476 | 44434152 | sense overlapping | NM_003425 | ZNF45 | ATCATCAAGCCATGTGAAGCTGCAGACTCCCAGCAAAAATGGACTAAGACATCACTTGAA |
| ASCRP3002789 | 0.688931874 | 0.943480789 | 1.0194458 | down | hsa_circRNA_401886 | | 25242744 | chr17 | - | 76115051 | 76115473 | exonic | NM_007267 | TMC6 | ATCATCAAGCTGCTGCTCGTCTTCTATGTCAAGAAGGATTATCTCCGAGAAGAAGCTGAA |
| ASCRP3002792 | 0.075519208 | 0.909237851 | 1.0796946 | down | hsa_circRNA_404615 | | 25070500 | chr1 | + | 176524542 | 176526377 | exonic | NM_020318 | PAPPA2 | ATCATCGCAGGAACCCACAAGACTCCCAGAAGGTGAAGTTAAGAGCTCCCAGACTCATAA |
| ASCRP3002795 | 0.085964673 | 0.909237851 | 1.0577646 | down | hsa_circRNA_045986 | hsa_circ_0045986 | circBase | chr17 | - | 78111184 | 78113939 | exonic | NM_014740 | EIF4A3 | ATCATGAAGGAGTTCCGGTCGGGCGCCAGGGGCTGCTTGCTCTCGGTGACTACATGAATG |
| ASCRP3002796 | 0.182623192 | 0.909237851 | 1.0651068 | down | hsa_circRNA_000272 | hsa_circ_0000272 | circBase | chr11 | - | 1491500 | 1492649 | sense overlapping | NM_053005 | MOB2 | ATCATGGACGACCTCACCGAGACAGTACTACTGGTATGACGAGCGGGGGAAGAAGGTCAA |
| ASCRP3002806 | 0.155438353 | 0.909237851 | 1.1221481 | down | hsa_circRNA_104341 | hsa_circ_0006010 | circBase | chr7 | - | 32672154 | 32678977 | exonic | NR_036680 | DPY19L1P1 | ATCATTTATATACACATGTGTACCTGTGTAATGTGGACACCACCTCTCCGCGAAAGCTTC |
| ASCRP3002811 | 0.234646722 | 0.909237851 | 1.0579267 | down | hsa_circRNA_102043 | hsa_circ_0043244 | circBase | chr17 | - | 35578644 | 35583373 | exonic | NM_198834 | ACACA | ATCCAAACAGGATCAGTTGTGTGGCCGGGACCCTACTCTCACTGATGAGCTGCTGAATAT |
| ASCRP3002815 | 0.607070433 | 0.929544944 | 1.0698569 | down | hsa_circRNA_102955 | hsa_circ_0007713 | circBase | chr2 | + | 238933982 | 238944581 | exonic | NM_080678 | UBE2F | ATCCACTGAATATTGAAGCTGCAGAACATCATTTGCGGGACAAGCCTCCCAAAGTGAAAT |
| ASCRP3002821 | 0.608233026 | 0.929544944 | 1.068068 | down | hsa_circRNA_023216 | hsa_circ_0023216 | circBase | chr11 | + | 68190956 | 68193655 | exonic | NM_002335 | LRP5 | ATCCATGCAGTGGAGGAAGTCAGCCTGGAGGAGTTCTCCCTTTGTTTTGACCTCTCTGAG |
| ASCRP3002833 | 0.205634327 | 0.909237851 | 1.0510844 | down | hsa_circRNA_020990 | hsa_circ_0020990 | circBase | chr11 | - | 6243671 | 6245937 | exonic | NM_032127 | FAM160A2 | ATCCTCGACACCCTCGTTGCTCGTATTGGCAGTAACTCCCGGATTTAGAAATCTGGACTG |
| ASCRP3002835 | 0.076756103 | 0.909237851 | 1.0874429 | down | hsa_circRNA_405813 | | 25070500 | chr19 | + | 54245398 | 54251644 | sense overlapping | NR_030215 | MIR520H | ATCCTGCCCTATTCTCTTGGTGAAATATAAGATTCCATTTTGGATGCTTCCATCGGGACT |
| ASCRP3002840 | 0.653695679 | 0.933189368 | 1.0142579 | down | hsa_circRNA_035301 | hsa_circ_0035301 | circBase | chr15 | + | 52311410 | 52358462 | exonic | NM_002748 | MAPK6 | ATCCTGTTTGAAGAAAAAGGACAAATAAAACATGGCCAGCAAATACACCCCTCTTCCTCG |
| ASCRP3002843 | 0.568309983 | 0.92124149 | 1.0294383 | down | hsa_circRNA_402893 | | 25242744 | chr3 | + | 62177142 | 62204657 | exonic | NM_002841 | PTPRG | ATCCTTCTCATTGCTGTGCTCGTTTACTGGAGTTTGCAGCTCTCCACCCATCCACATGAA |
| ASCRP3002846 | 0.089772535 | 0.909237851 | 1.164565 | down | hsa_circRNA_101238 | hsa_circ_0004372 | circBase | chr13 | + | 28748408 | 28752072 | exonic | NM_175854 | PAN3 | ATCCTTGTTTAATGACTTTGGTGCCCTCAACATCTCTCAGAGACGAAAGTTCCAGGAATG |
| ASCRP3002847 | 0.188851586 | 0.909237851 | 1.034708 | down | hsa_circRNA_101989 | hsa_circ_0006600 | circBase | chr17 | + | 16979023 | 16981390 | exonic | NM_015134 | MPRIP | ATCCTTTACGAGCACGGCCTCTTGCGCTACGCCCTGGATGAGATGGCAAAACCCATTTAT |
| ASCRP3002849 | 0.786101282 | 0.966895211 | 1.0057961 | down | hsa_circRNA_406528 | | 25070500 | chr4 | - | 125422050 | 125477665 | sense overlapping | NR_110838 | LOC101927087 | ATCGAAACTAATGATAAACTTCAATCAATGACATTGAGGGGATGGGGGACGAGACACACA |
| ASCRP3002857 | 0.563818139 | 0.920978531 | 1.0338375 | down | hsa_circRNA_406408 | | 25070500 | chr3 | + | 185146284 | 185155418 | exonic | NM_004721 | MAP3K13 | ATCGCATTCAAGTTTTGGAGCCCTCTCTTAAGTCAGAACTCTGTCCCAAAAATCTTCTGA |
| ASCRP3002858 | 0.03074068 | 0.909237851 | 1.0629363 | down | hsa_circRNA_401530 | | 25242744 | chr16 | - | 4953886 | 4960950 | exonic | NM_002705 | PPL | ATCGCCGAGGACATCTCTAACAAGGAGCTCTCGGAGCTGATCGAGCAGCTGCAGAAGAAT |
| ASCRP3002875 | 0.521581824 | 0.916971445 | 1.035733 | down | hsa_circRNA_101072 | hsa_circ_0026524 | circBase | chr12 | + | 53421565 | 53431406 | exonic | NM_001417 | EIF4B | ATCTCAGAGCTCAGACACAGAGCAGCAGTCCCCTACAAGAGTATCGAGATCGTTATGATT |
| ASCRP3002878 | 0.649357032 | 0.932467957 | 1.0242379 | down | hsa_circRNA_100782 | hsa_circ_0000284 | circBase | chr11 | + | 33307958 | 33309057 | exonic | NM_005734 | HIPK3 | ATCTCGGTACTACAGGTATGGCCTCACAAGTCTTGGTCTACCCACCATATGTTTATCAAA |
| ASCRP3002883 | 0.271627292 | 0.909237851 | 1.0244013 | down | hsa_circRNA_065887 | hsa_circ_0065887 | circBase | chr3 | + | 51317536 | 51393924 | exonic | NM_004947 | DOCK3 | ATCTCTCGGTGGTTTGAAGTGGAGAGGAGGGAACTGGGCGAGTATGTGTCCTGCCTTCTC |
| ASCRP3002886 | 0.252138706 | 0.909237851 | 1.0928018 | down | hsa_circRNA_080302 | hsa_circ_0080302 | circBase | chr7 | + | 64526630 | 64527428 | exonic | NR_033416 | CCT6P3 | ATCTGAAACTGATACGAGGGCCTTCATCCCAGAATAATCACTGAAGGATTTGAAGCTGTG |
| ASCRP3002894 | 0.902702959 | 0.981257955 | 1.0047673 | down | hsa_circRNA_004196 | hsa_circ_0004196 | circBase | chr13 | + | 24164288 | 24190184 | exonic | NM_018647 | TNFRSF19 | ATCTGCGGGGACTGCTTGCCAGGAACTCTCCAACAATAAATACATTTGATAAGAAAGATG |
| ASCRP3002906 | 0.231368851 | 0.909237851 | 1.1626927 | down | hsa_circRNA_103714 | hsa_circ_0070610 | circBase | chr4 | - | 108565957 | 108622441 | exonic | NM_005443 | PAPSS1 | ATCTTCCCATCTCCCATGATGTATGCTGGACCAACTGAGGGAATGCAGAGAGCAACCAAT |
| ASCRP3002910 | 0.580346565 | 0.925542365 | 1.0321419 | down | hsa_circRNA_406652 | | 25070500 | chr5 | + | 119952789 | 119954260 | exonic | ENST00000505123 | PRR16 | ATCTTCTCAATCAAATTCTACTTCAGAGAAGCTCCATTACCTACTCCCACCTCCCCACAT |
| ASCRP3002913 | 0.204338022 | 0.909237851 | 1.0588721 | down | hsa_circRNA_007579 | hsa_circ_0007579 | circBase | chr12 | - | 24982756 | 25002883 | exonic | NM_005504 | BCAT1 | ATCTTGAGCAAATTAACTGATATCCAGCCTTCTCTTGGAGTCAAGAAGCCTACCAAAGCC |
| ASCRP3002914 | 0.27333246 | 0.909237851 | 1.0304253 | down | hsa_circRNA_001128 | hsa_circ_0001128 | circBase | chr20 | + | 4763319 | 4763528 | antisense | NM_014737 | RASSF2 | ATCTTGCTTGGTCAATCTCCATAGCCAGGCACCCAGGGCAGAAACGTCTCCACCAAAAAA |
| ASCRP3002916 | 0.319149028 | 0.909237851 | 1.0465902 | down | hsa_circRNA_100916 | hsa_circ_0008773 | circBase | chr11 | - | 85692787 | 85695016 | exonic | NM_007166 | PICALM | ATCTTGGCATCGGAAATGGAACCACTAAGAAGATTCACTCCTTCTCCAGTTGCACAGCCA |
| ASCRP3002927 | 0.218879766 | 0.909237851 | 1.0802201 | down | hsa_circRNA_100053 | hsa_circ_0009910 | circBase | chr1 | + | 12049221 | 12052747 | exonic | NM_014874 | MFN2 | ATGAAAGTGGCTTTTTTTGGCCGCGCAATGTCCCTGCTCTTCTCTCGATGCAACTCTATC |
| ASCRP3002933 | 0.176200663 | 0.909237851 | 1.0519243 | down | hsa_circRNA_010402 | hsa_circ_0010402 | circBase | chr1 | - | 19683128 | 19684057 | exonic | NM_004930 | CAPZB | ATGAACCTCGGAGGCAGCCTTACCAGACAGGTATTTTGAAGGTGGCGTCTCATCTGTCTA |
| ASCRP3002938 | 0.371077932 | 0.91119147 | 1.0342629 | down | hsa_circRNA_100938 | hsa_circ_0008648 | circBase | chr11 | + | 94554695 | 94564707 | exonic | NM_130847 | AMOTL1 | ATGAAGACAAAGCTGCAGAGGGGCATTATGCTTCCCAGACCGCCCATGCCAACTTCCGTT |
| ASCRP3002939 | 0.987239333 | 0.998148217 | 1.0005905 | down | hsa_circRNA_103524 | hsa_circ_0068162 | circBase | chr3 | - | 183097084 | 183107567 | exonic | NM_015078 | MCF2L2 | ATGAAGACTTCCTGAATGTCATGACCTACCTGACTAGCATCCCCAGATGAAATTATGCAG |
| ASCRP3002942 | 0.226390268 | 0.909237851 | 1.0393234 | down | hsa_circRNA_405407 | | 25070500 | chr15 | + | 81419930 | 81436161 | exonic | uc010blp.1 | C15orf26 | ATGAAGGCTTCCCCGTCCCGGTGGGCTTATCTAAATCAGATCCCAAATAGTATTTAAAAA |
| ASCRP3002944 | 0.510752288 | 0.916805276 | 1.0277507 | down | hsa_circRNA_104530 | hsa_circ_0005181 | circBase | chr7 | + | 152480271 | 152522207 | exonic | NM_020445 | ACTR3B | ATGAAGTAATACAGAACTGCCCCATCGATGTGCGGCGCCCGCTGTATAAGGTATACCAAG |
| ASCRP3002952 | 0.026290309 | 0.909237851 | 1.1073411 | down | hsa_circRNA_103395 | hsa_circ_0008107 | circBase | chr3 | - | 57561274 | 57570191 | exonic | NM_001660 | ARF4 | ATGACAGATAAACTAGGGCTTCAGTCTCTTCGTAACAGAACATTGGATTGGATGCTGCTG |
| ASCRP3002957 | 0.670123314 | 0.938080316 | 1.0153045 | down | hsa_circRNA_079387 | hsa_circ_0079387 | circBase | chr7 | + | 6629914 | 6641771 | exonic | uc003sqo.1 | C7orf26 | ATGACCTGAGAACCTTGTGCTCCAGGCTGCCCCATAATAAATGAGCGACATCCGCCACTC |
| ASCRP3002963 | 0.044363985 | 0.909237851 | 1.0762715 | down | hsa_circRNA_406618 | | 25070500 | chr5 | + | 68470081 | 68470236 | intronic | ENST00000508407 | CCNB1 | ATGACTGTCTCCATTATTGATCGGTTCATGCAGAGCAAGCAGTCAGACCAAAATACCTAC |
| ASCRP3002974 | 0.373391871 | 0.91119147 | 1.0806364 | down | hsa_circRNA_404870 | | 25070500 | chr11 | + | 28250417 | 28252532 | intronic | ENST00000303459 | METTL15 | ATGAGATCAGCTGGTTCCTCCCTCTTTGAACTCATCTGGATCTGGCTTATTTCAAATGTC |
| ASCRP3002979 | 0.214522939 | 0.909237851 | 1.039672 | down | hsa_circRNA_101839 | hsa_circ_0039943 | circBase | chr16 | + | 68300495 | 68300624 | exonic | NM_003983 | SLC7A6 | ATGAGGAGGTGGCTTATGAAAGTGTGATGTTCGCGTATTTCTTGACAGAGTTTATGTGGC |
| ASCRP3002980 | 0.092950653 | 0.909237851 | 1.1132593 | down | hsa_circRNA_007482 | hsa_circ_0007482 | circBase | chr9 | + | 137582757 | 137593179 | exonic | NM_000093 | COL5A1 | ATGAGGAGGTGTTTGAGCTCAGCCAGCAGATCTCCTGAAGGTTCTAGATTTTCACAACTT |
| ASCRP3002981 | 0.0365295 | 0.909237851 | 1.1148565 | down | hsa_circRNA_006005 | hsa_circ_0006005 | circBase | chr2 | + | 207705830 | 207746615 | sense overlapping | ENST00000438070 | AC008269.2 | ATGAGGCAAAGCACACTCTGAAGTTTTCCTCTTCTCCTGTGTTAGAAAGCGTGAAGAATA |
| ASCRP3002983 | 0.885105118 | 0.979863126 | 1.0042924 | down | hsa_circRNA_009000 | hsa_circ_0009000 | circBase | chr7 | + | 30661008 | 30662078 | exonic | NM_002047 | GARS | ATGAGTGCTACATTACAGAAATGGAGATGCTGCTGAATGAGAAAGGGGTTGGATTGAGAT |
| ASCRP3002999 | 0.548139466 | 0.919549314 | 1.018446 | down | hsa_circRNA_001381 | hsa_circ_0001381 | circBase | chr3 | - | 196386979 | 196388380 | antisense | NM_198565 | NRROS | ATGATGGTGTTCCCCGCCAGGGACACGGACCGCAGCGAGGAGAGGTTGCAGGTGACCATG |
| ASCRP3003004 | 0.59026517 | 0.927905266 | 1.0608642 | down | hsa_circRNA_001153 | hsa_circ_0001455 | circBase | chr4 | + | 164514761 | 164515093 | antisense | NM_017923 | MARCH1 | ATGCAAGGTTTTATAAGCTCATCCTCAACCATGACTTCCTTCCAATTTTGTCCACTCATT |
| ASCRP3003013 | 0.003942465 | 0.870215632 | 1.0538286 | down | hsa_circRNA_032393 | hsa_circ_0032393 | circBase | chr14 | + | 71434914 | 71445365 | exonic | NM_014982 | PCNX | ATGCAGTCAGTGGAGATTGGATCTGGTTCCTCGCGTCTTGGAACAGCAGCAACTATTAAA |
| ASCRP3003024 | 0.692078558 | 0.943480789 | 1.0213749 | down | hsa_circRNA_404463 | | 25070500 | chr1 | - | 27440281 | 27440777 | intronic | ENST00000545949 | SLC9A1 | ATGCCCATGTCCAAACGGTTTCCATGTGATCCCCACTATCTCAAGCATCGTCCCGGAGAG |
| ASCRP3003030 | 0.438878341 | 0.91119147 | 1.0422251 | down | hsa_circRNA_002172 | hsa_circ_0000514 | circBase | chr14 | - | 20811305 | 20811436 | sense overlapping | NR_002312 | RPPH1 | ATGCCTCCTTTGCCGGAGCTTGGAACAGACTCACGGCCAGCGAAGTGAGTTCCCAGAGAA |
| ASCRP3003037 | 0.103279881 | 0.909237851 | 1.0524356 | down | hsa_circRNA_102300 | hsa_circ_0006990 | circBase | chr18 | + | 9931806 | 9937063 | exonic | NM_003574 | VAPA | ATGCGTATTTGAAATGCCCAATGAAAATGATAAATTGGCCCCTTCACAGATGTAGTCACT |
| ASCRP3003045 | 0.007001931 | 0.879037174 | 1.1017037 | down | hsa_circRNA_089739 | hsa_circ_0089739 | circBase | chr9 | + | 140671069 | 140676849 | exonic | NM_024757 | EHMT1 | ATGCTGGTTCAGGGTAATTTTATGGAGTGTCAGCCCGAGAGCAGCATCTCTCACCGTTTC |
| ASCRP3003048 | 0.444026603 | 0.91119147 | 1.069567 | down | hsa_circRNA_103846 | hsa_circ_0002512 | circBase | chr5 | - | 59934576 | 59943360 | exonic | NM_018369 | DEPDC1B | ATGCTTTTGTCAGTGTACTGGATTTCCTCCTTCTTCACCCCTGAAACCATATCCAAAGAA |
| ASCRP3003049 | 0.079728492 | 0.909237851 | 1.0783241 | down | hsa_circRNA_103744 | hsa_circ_0006552 | circBase | chr4 | - | 143029245 | 143045913 | exonic | NM_003866 | INPP4B | ATGGAAAAGATGCCTCCTGATTAGGACTGGTATGAACAGTTGTATCCCCTCATCCTTACC |
| ASCRP3003051 | 0.482676741 | 0.91119147 | 1.0957893 | down | hsa_circRNA_086412 | hsa_circ_0086412 | circBase | chr9 | - | 16435031 | 16437522 | exonic | uc003zmi.3 | BNC2 | ATGGAAAGAACCATAGGTCCAGGACATCGAGACAGCCTGCATCTCCGTAGGATGCTGCTG |
| ASCRP3003073 | 0.381829503 | 0.91119147 | 1.0684826 | down | hsa_circRNA_104950 | hsa_circ_0089252 | circBase | chr9 | - | 134514021 | 134526336 | exonic | NM_005312 | RAPGEF1 | ATGGAGTGAAGACTCTCAGCGTTCTCATCTCTCTTCCTTCACCATGAAGCTGATGGACAA |
| ASCRP3003076 | 0.473994281 | 0.91119147 | 1.0268203 | down | hsa_circRNA_055290 | hsa_circ_0055290 | circBase | chr2 | - | 74531615 | 74542152 | exonic | NM_021196 | SLC4A5 | ATGGATCTTATCAGCAGGACTCTCATGAAGGTGAAGGAGGAGAAGGCTGGGGTAGGAAAG |
| ASCRP3003078 | 0.595117829 | 0.929544944 | 1.0447324 | down | hsa_circRNA_103297 | hsa_circ_0064416 | circBase | chr3 | + | 13542212 | 13544480 | exonic | NM_024827 | HDAC11 | ATGGATGTCTACAACCGCCACATCTACCCAGGGGACCGCTTTGCCAAGCGGGGTGGCTTC |
| ASCRP3003088 | 0.985072079 | 0.998148217 | 1.0006203 | down | hsa_circRNA_100208 | hsa_circ_0012283 | circBase | chr1 | + | 46383010 | 46425149 | exonic | uc001coz.1 | MAST2 | ATGGCCACACAGAATCTCTACCCTTTGCCGAGGAGCTGAAGTAAACCAGCACATGTTTTC |
| ASCRP3003091 | 0.09341302 | 0.909237851 | 1.1883155 | down | hsa_circRNA_401636 | | 25242744 | chr16 | + | 69687138 | 69693802 | exonic | NM_006599 | NFAT5 | ATGGCTCCACTTTGACACTGCAAACACCCTCTTCTCCAATTTTGTGTAAACATTGGAAAA |
| ASCRP3003095 | 0.409076231 | 0.91119147 | 1.0230319 | down | hsa_circRNA_000760 | hsa_circ_0000760 | circBase | chr17 | + | 36865777 | 36868169 | sense overlapping | NM_005937 | MLLT6 | ATGGGGGGAGGTGGACAACGTCAAGTACTGCGGCTACTGCAAATACCACTTCAGCAAGAT |
| ASCRP3003096 | 0.075436669 | 0.909237851 | 1.0344958 | down | hsa_circRNA_071127 | hsa_circ_0071127 | circBase | chr4 | - | 149356255 | 149358014 | exonic | NM_000901 | NR3C2 | ATGGGTATCCGGTCTTAGAATACATTCCAGAAAATGTATCAAGCGATGGAGACCAAAGGC |
| ASCRP3003107 | 0.440268437 | 0.91119147 | 1.0458703 | down | hsa_circRNA_012173 | hsa_circ_0012173 | circBase | chr1 | + | 45241705 | 45244412 | exonic | NM_001012 | RPS8 | ATGTAATAAAGGTGTTTATTGTTTTGTTCCCACAGCATCTCTCGGGACAACTGGCACAAG |
| ASCRP3003109 | 0.895548082 | 0.980902785 | 1.0142014 | down | hsa_circRNA_002453 | hsa_circ_0002453 | circBase | chr3 | - | 8977554 | 8990254 | exonic | NM_020165 | RAD18 | ATGTACAATGCCCAATGCGATGCTTTGCATCCTAAATCAGACTGCTCTCTCTGTATAAGA |
| ASCRP3003111 | 0.993260024 | 0.998961292 | 1.0006811 | down | hsa_circRNA_101215 | hsa_circ_0029531 | circBase | chr12 | - | 133245396 | 133248908 | exonic | NM_006231 | POLE | ATGTACCCCAACATCATCCTGACCAACCGCCTGCAGAATCCTGCCGCCTTTGACTTCCTG |
| ASCRP3003113 | 0.904441296 | 0.981658611 | 1.0059217 | down | hsa_circRNA_080790 | hsa_circ_0080790 | circBase | chr7 | - | 75956107 | 75988342 | exonic | NM_012479 | YWHAG | ATGTAGCTGTCGTACATTGAGCAAATAAACTTACAGATCTGACGCCAGTGAGGGGGTTGT |
| ASCRP3003122 | 0.460756212 | 0.91119147 | 1.0260186 | down | hsa_circRNA_028671 | hsa_circ_0028671 | circBase | chr12 | - | 118682698 | 118693460 | exonic | NM_016281 | TAOK3 | ATGTCCTATAGTGGGAAGCAGACCCATGAGGTACTGAAGATTTACCCCCCAAAAAAAATT |
| ASCRP3003134 | 0.077089234 | 0.909237851 | 1.0716936 | down | hsa_circRNA_100163 | hsa_circ_0011542 | circBase | chr1 | + | 35846859 | 35855699 | exonic | NM_005095 | ZMYM4 | ATGTGACCCGCCTTCACAAAATAATGCAGAGACATTTTAAATCCAAAGGATGTGATCAGT |
| ASCRP3003137 | 0.261710163 | 0.909237851 | 1.1062077 | down | hsa_circRNA_000792 | hsa_circ_0000291 | circBase | chr11 | + | 35163017 | 35163328 | intronic | ENST00000263398 | CD44 | ATGTGCTTGTAAAAAGAGGGCCCTGGTGGTTTCCCTATAGTCTCTAGGGAAGTTTATTTG |
| ASCRP3003140 | 0.607117197 | 0.929544944 | 1.0331718 | down | hsa_circRNA_104106 | hsa_circ_0006232 | circBase | chr6 | - | 42235891 | 42237586 | exonic | NM_033502 | TRERF1 | ATGTGGCCCCAGGTGTGAGCGACGTCTCCTCACCACAGTGCTGTGTGGTCTATACCTCAG |
| ASCRP3003155 | 0.154381664 | 0.909237851 | 1.0548572 | down | hsa_circRNA_100412 | hsa_circ_0015454 | circBase | chr1 | + | 179316734 | 179319566 | exonic | NM_003101 | SOAT1 | ATGTTCTTTGGAAGTGTGCTGATTCTCTTCCTTACTTTTTTTGCCTTTTTGCACTGCTGG |
| ASCRP3003163 | 0.360274881 | 0.91119147 | 1.0402189 | down | hsa_circRNA_091743 | hsa_circ_0091743 | circBase | chrX | + | 152771320 | 152775004 | exonic | NM_001711 | BGN | ATTAAAGAAACACCGTGCAACGTGGCCCTCGTCCTGGTGAACAACAAGATCTCCAAGATC |
| ASCRP3003164 | 0.07241475 | 0.909237851 | 1.076703 | down | hsa_circRNA_405043 | | 25070500 | chr12 | - | 95498782 | 95535315 | exonic | NM_018351 | FGD6 | ATTAAAGATTGAAAGTGTAGAACGTTCCTTCATTCTCTCAGCCAGGATTTCCGGGATGCA |
| ASCRP3003169 | 0.198038079 | 0.909237851 | 1.0502459 | down | hsa_circRNA_036286 | hsa_circ_0036286 | circBase | chr15 | + | 74882160 | 74885577 | exonic | NM_006465 | ARID3B | ATTAACATGTCTGTGGACATCGATGGCACCACCTATGCAGGGACCCCCATCAACCGAATC |
| ASCRP3003177 | 0.673818383 | 0.938080316 | 1.0139596 | down | hsa_circRNA_100340 | hsa_circ_0014186 | circBase | chr1 | + | 151611363 | 151655921 | exonic | NM_030918 | SNX27 | ATTACAGAAGCTATACGAACAAAGAAAAATGGTCATGGAACCACGTGAATGTTGAGGGGG |
| ASCRP3003180 | 0.0633312 | 0.909237851 | 1.0566351 | down | hsa_circRNA_400230 | | 25242744 | chr1 | - | 52289335 | 52293541 | exonic | NM_002525 | NRD1 | ATTACATGACTTTAGTGGTTCAATCCAAAGATGGGCGCAGTTCTTCATCCACCCACTAAT |
| ASCRP3003184 | 0.61724134 | 0.929544944 | 1.0278203 | down | hsa_circRNA_007004 | hsa_circ_0007004 | circBase | chr7 | + | 102039994 | 102047945 | exonic | NM_024653 | PRKRIP1 | ATTACTGGCAAAGAAGATGAAACTTGAACAGAAGAAACAAGAAGGTTCAAGTGCTGGGGC |
| ASCRP3003198 | 0.726449037 | 0.951019156 | 1.036359 | down | hsa_circRNA_102100 | hsa_circ_0044226 | circBase | chr17 | - | 45214517 | 45221348 | exonic | NM_001256 | CDC27 | ATTATAATGCATGTACAACACCTCAGGTATTGAGCCCCACTATTACATCTCCCCCAAACG |
| ASCRP3003199 | 0.955008509 | 0.992245164 | 1.0023024 | down | hsa_circRNA_021467 | hsa_circ_0021467 | circBase | chr11 | - | 18568426 | 18588029 | exonic | NM_018314 | UEVLD | ATTATAGTCAACACAGTGTCCTGCTCGTTGCATCTCAACCAGCCTAAATCTGTCATTGTT |
| ASCRP3003210 | 0.849328652 | 0.978900086 | 1.0077513 | down | hsa_circRNA_092540 | hsa_circ_0001408 | circBase | chr4 | - | 42554521 | 42583749 | exonic | NM_006095 | ATP8A1 | ATTATTTATCAAGCAGCATCTCCAGCACCGTTCCACTGGGAGCAGATCAGATTCTTCTTC |
| ASCRP3003222 | 0.55290097 | 0.919549314 | 1.0189416 | down | hsa_circRNA_004798 | hsa_circ_0004798 | circBase | chr11 | + | 123464789 | 123466745 | exonic | NM_020716 | GRAMD1B | ATTCAGAAAAGGTGTTAAGCCCCACCTACAAGCAGAGAAATGAAGACTTCAGAAAGCTCT |
| ASCRP3003227 | 0.514104293 | 0.916971445 | 1.0278086 | down | hsa_circRNA_103034 | hsa_circ_0059998 | circBase | chr20 | - | 33356266 | 33356389 | exonic | NM_014071 | NCOA6 | ATTCAGTTAGGATGGAGGCGGGATTTCCTATGGCAAGTGGTCCAGGGGAAGGTGCTATTA |
| ASCRP3003234 | 0.082757732 | 0.909237851 | 1.0525328 | down | hsa_circRNA_007514 | hsa_circ_0007514 | circBase | chr13 | - | 114817526 | 114822949 | exonic | uc001vuj.3 | RASA3 | ATTCCATCATAGAGACTGGCTCCAGCTTTCCTTGAAATCCTGCAGAGCCTTCATTAAAAT |
| ASCRP3003237 | 0.891961161 | 0.979863126 | 1.0062309 | down | hsa_circRNA_051334 | hsa_circ_0051334 | circBase | chr19 | - | 44010870 | 44015718 | exonic | NM_014297 | ETHE1 | ATTCCCTGGAGCTCCCTAAATAAAACTTTTTTTAACGTGAGCGTTGGAGACCAGGGCCAG |
| ASCRP3003238 | 0.458447676 | 0.91119147 | 1.0099078 | down | hsa_circRNA_101205 | hsa_circ_0006078 | circBase | chr12 | + | 125587224 | 125591814 | exonic | NM_023928 | AACS | ATTCCGCTGGGGGTGTGCTGGACCGGTTTTCTCAAATTCAGCCAAAGCTCATCTTCTCTG |
| ASCRP3003251 | 0.238950061 | 0.909237851 | 1.1143378 | down | hsa_circRNA_100726 | hsa_circ_0002456 | circBase | chr10 | + | 128923737 | 128926028 | exonic | NM_001380 | DOCK1 | ATTCTGAACTCATTACTGCAGAGAGATCCTGCTTCCCATGATGACCGATCAGCTCAAGTA |
| ASCRP3003266 | 0.312350104 | 0.909237851 | 1.0396428 | down | hsa_circRNA_400734 | | 25242744 | chr11 | - | 33163212 | 33163532 | exonic | NM_001326 | CSTF3 | ATTGAAGCAGAGGCAGCTGAGTATGTCCCAGAGAAGGTGAAGAAAGCGGAAAAGAAATTA |
| ASCRP3003267 | 0.563085178 | 0.920978531 | 1.0165758 | down | hsa_circRNA_009062 | hsa_circ_0009062 | circBase | chr2 | + | 44436348 | 44445676 | exonic | NM_002706 | PPM1B | ATTGAAGCTGTTTATAGTAGACTGAATCCACATAGAGAAAGTGATGGGGGAAGTCGAGAT |
| ASCRP3003274 | 0.016322091 | 0.909237851 | 1.0888305 | down | hsa_circRNA_104936 | hsa_circ_0089033 | circBase | chr9 | + | 131891263 | 131893918 | exonic | NM_021131 | PPP2R4 | ATTGACTACGGCACAGGCCATTGAGAAACTAGTCGCTCTTCTCAACACGCTGGACAGGTG |
| ASCRP3003275 | 0.064381881 | 0.909237851 | 1.0411743 | down | hsa_circRNA_003179 | hsa_circ_0003179 | circBase | chr3 | - | 101389973 | 101391057 | exonic | NM_014415 | ZBTB11 | ATTGAGAAAGGAGCTGTTTCCAGTCATGAGGCTGTGGTGGATCTTTCTGGTATATTGAAG |
| ASCRP3003292 | 0.062416874 | 0.909237851 | 1.1744126 | down | hsa_circRNA_103598 | hsa_circ_0069104 | circBase | chr4 | + | 6925099 | 7002978 | exonic | NM_020773 | TBC1D14 | ATTGGCAACGAGTTAAATATCACCCACGTTTCTCCTTGGACCAAGATGACTGATGGAAAA |
| ASCRP3003308 | 0.329189407 | 0.909237851 | 1.0179753 | down | hsa_circRNA_101647 | hsa_circ_0036768 | circBase | chr15 | + | 90763024 | 90764997 | exonic | NM_020210 | SEMA4B | ATTGTGTCCCGCATTGCCCGCATCTGCAAGCGCGACTGTCAAAACTACATCAAGATCCTC |
| ASCRP3003313 | 0.612630805 | 0.929544944 | 1.0973164 | down | hsa_circRNA_400062 | hsa_circ_0092291 | circBase | chr20 | - | 32677771 | 32678011 | intronic | ENST00000374980 | EIF2S2 | ATTTAAGAAGGTTGGCATGTCTGTCAACATGTTTGAGTGGCTCTCCTCTAGACAGAAACG |
| ASCRP3003324 | 0.074185564 | 0.909237851 | 1.0356898 | down | hsa_circRNA_101009 | hsa_circ_0025388 | circBase | chr12 | + | 9020437 | 9021799 | exonic | NM_144670 | A2ML1 | ATTTACTTGGATGAGGATACTGTAGTTGCTCTCCAAGCTCTTGCCAAATATGCCACTACC |
| ASCRP3003344 | 0.42708612 | 0.91119147 | 1.0298904 | down | hsa_circRNA_101767 | hsa_circ_0038648 | circBase | chr16 | + | 24043456 | 24105618 | exonic | NM_002738 | PRKCB | ATTTCTGAACTTCAGAAAGCCAGTGTTGATGGCTGGACCCCCGCAGCAAACACAAGTTTA |
| ASCRP3003346 | 0.633841261 | 0.929544944 | 1.0235454 | down | hsa_circRNA_104254 | hsa_circ_0001663 | circBase | chr6 | + | 159004985 | 159010814 | exonic | NM_020823 | TMEM181 | ATTTGAACACCTGAAGCTCCCCATCAAGGGAATGAACTTCACACTAAAGCCAATTCAAAT |
| ASCRP3003350 | 0.736712033 | 0.953233938 | 1.0194842 | down | hsa_circRNA_104503 | hsa_circ_0082582 | circBase | chr7 | + | 138203933 | 138255748 | exonic | NM_003852 | TRIM24 | ATTTGAGCTCACCAGTGGGAGGGTCTTATAATCTTCCCTCTCTTCCGGATAGGCAGTTGG |
| ASCRP3003371 | 0.096673626 | 0.909237851 | 1.0744608 | down | hsa_circRNA_102234 | hsa_circ_0046292 | circBase | chr17 | + | 79941429 | 79943483 | exonic | NM_024083 | ASPSCR1 | ATTTTCCACAGATCAGGTTTCAGAGGAGCGTGCTCGACCTTTCTCTCCAGTGGAGATTTG |
| ASCRP3003378 | 0.833626168 | 0.978181218 | 1.0062839 | down | hsa_circRNA_406110 | | 25070500 | chr20 | - | 43133561 | 43135467 | intronic | ENST00000255175 | SERINC3 | ATTTTGGAAAGTCAGCCCTTGGCAGTCCCTGTATGATTGTTTACTACTTCTTTCTCCTGT |
| ASCRP3003395 | 0.011614738 | 0.909237851 | 1.083092 | down | hsa_circRNA_004129 | hsa_circ_0004129 | circBase | chr9 | - | 72082738 | 72091059 | exonic | NM_001163 | APBA1 | CAAAAACGTGCGCATGATGCAGGCCCAGGAAGCCGTAAGCAGGATCAAGTCTCCGTCTCC |
| ASCRP3003396 | 0.282225909 | 0.909237851 | 1.0460116 | down | hsa_circRNA_405275 | | 25070500 | chr14 | - | 89123719 | 89131833 | exonic | NM_183387 | EML5 | CAAAAAGAACCCTCAATTGATGAAAGGTGGGAGGTGTTGCTGTTTTTCTCCTGATGGTAA |
| ASCRP3003400 | 0.918837911 | 0.98346125 | 1.0040736 | down | hsa_circRNA_406682 | | 25070500 | chr5 | - | 139421954 | 139422721 | sense overlapping | NM_004883 | NRG2 | CAAAAATCTCAAGAAAGAGGTGGGCAAGATCCTGTGCACTGACTGCGCGGGCTCTGTTTC |
| ASCRP3003406 | 0.93572824 | 0.987920247 | 1.0043276 | down | hsa_circRNA_406104 | | 25070500 | chr20 | + | 34435271 | 34451322 | exonic | NM_016436 | PHF20 | CAAAAGAAAACGAGGCAGACCCCCTTCCATAGCTCCTACTGGAATTTCAAATAAATGAGC |
| ASCRP3003410 | 0.927515483 | 0.985498678 | 1.00155 | down | hsa_circRNA_007352 | hsa_circ_0007352 | circBase | chrX | + | 1712336 | 1714425 | exonic | NM_005088 | AKAP17A | CAAAAGCGCAGAGAGAAGGAAGCGGAGGAGAGGCAGCGAGCGGAGGAAAGGCCCAAGGTC |
| ASCRP3003412 | 0.966388442 | 0.995283777 | 1.0030476 | down | hsa_circRNA_103365 | hsa_circ_0005435 | circBase | chr3 | - | 47770514 | 47814426 | exonic | NM_003074 | SMARCC1 | CAAAAGCTTCCCACCACATTTACCCATATTCTTCCTCACAAGACGATGTATGTTCATGCG |
| ASCRP3003413 | 0.401898786 | 0.91119147 | 1.0518593 | down | hsa_circRNA_092558 | hsa_circ_0001594 | circBase | chr6 | - | 27775963 | 27782476 | sense overlapping | NM_021066 | HIST1H2AJ | CAAAAGGCCTATTATCTATACGGCCAAGACCCGCTCTTCTCGGGCCGGGCTTCAGTTTCC |
| ASCRP3003417 | 0.065597504 | 0.909237851 | 1.1821754 | down | hsa_circRNA_089863 | hsa_circ_0089863 | circBase | chrX | + | 9905173 | 9905725 | exonic | NM_001649 | SHROOM2 | CAAAATCCCCTCTCCTAGAAGCACAGAGGAGAGAATTGAGCGGGTGATGGACAACAACAC |
| ASCRP3003428 | 0.903214497 | 0.981257955 | 1.0074761 | down | hsa_circRNA_403818 | | 25242744 | chr7 | + | 64377407 | 64378670 | exonic | NR_003099 | ZNF273 | CAAACCCCCAGGCCAGAAAAACTGGGAAAATCACAGGCTCTTCCACTTACTGGATGTTTG |
| ASCRP3003433 | 0.469009369 | 0.91119147 | 1.0293645 | down | hsa_circRNA_101756 | hsa_circ_0038487 | circBase | chr16 | + | 22236974 | 22237296 | exonic | NM_013302 | EEF2K | CAAACTCCTTCCACTTCAAGGACCTTCGCCTCTGCATTTGTCCAGTAACTCTGGCTGTGC |
| ASCRP3003438 | 0.219018815 | 0.909237851 | 1.0692949 | down | hsa_circRNA_103605 | hsa_circ_0009006 | circBase | chr4 | - | 16587544 | 16597498 | exonic | NM_001290 | LDB2 | CAAACTTCACCCTCAACTACCTCAGGCTATCGGCAGGACCCTCATCCCCCGTTACTTTAG |
| ASCRP3003439 | 0.180560342 | 0.909237851 | 1.0744683 | down | hsa_circRNA_022963 | hsa_circ_0022963 | circBase | chr11 | + | 66050718 | 66050862 | exonic | NM_182553 | CNIH2 | CAAACTTGCCTTCTACCTGCTCTCCTTCTTCTATTACCTGTACAGGTACTTCCACCGTCC |
| ASCRP3003441 | 0.855121822 | 0.978900086 | 1.0037288 | down | hsa_circRNA_406963 | | 25070500 | chr7 | + | 77282987 | 77285143 | intergenic |  |  | CAAAGAAATTGTCTGAAAGGAGGTGGCCGTCAGTCAGAGTGAAGAAGTCAACTTGATCAG |
| ASCRP3003444 | 0.534144994 | 0.919549314 | 1.0573386 | down | hsa_circRNA_069397 | hsa_circ_0069397 | circBase | chr4 | - | 36212011 | 36231267 | exonic | NM_015230 | ARAP2 | CAAAGAAGGTTAAATCAGGATGGCTGGATAAACTCTCTCCTCAAGGGCTTAAGCAGGTAA |
| ASCRP3003450 | 0.186670257 | 0.909237851 | 1.1315825 | down | hsa_circRNA_102889 | hsa_circ_0004757 | circBase | chr2 | - | 200245086 | 200246543 | exonic | NM_015265 | SATB2 | CAAAGAGATGAACCAGAGCACATTAGCCAAAGAATGCCCTCTCTCCCAGGAATAATCAAG |
| ASCRP3003453 | 0.156788208 | 0.909237851 | 1.0215652 | down | hsa_circRNA_101928 | hsa_circ_0005351 | circBase | chr17 | - | 706921 | 708487 | exonic | NM_022463 | NXN | CAAAGAGGAGGAGGCACCCCTTCTGTTCTTCGTAGCCGGGGAGGCATCCCCACGCTCATC |
| ASCRP3003454 | 0.245041808 | 0.909237851 | 1.0434082 | down | hsa_circRNA_101154 | hsa_circ_0002488 | circBase | chr12 | + | 112321384 | 112321572 | exonic | NM_003668 | MAPKAPK5 | CAAAGATGTTGTGAGGAAAGCTGTGACTTGTGGTCCCTAGGGGTGATTATCTATGTGATG |
| ASCRP3003462 | 0.002063091 | 0.870215632 | 1.0639173 | down | hsa_circRNA_104366 | hsa_circ_0006229 | circBase | chr7 | - | 47384352 | 47385954 | exonic | NM_022748 | TNS3 | CAAAGGAGGACGTGGCTCCTCTGCTGAACAGCCCCTGGGCGGGAGACTCAGGAAGCTGAG |
| ASCRP3003473 | 0.519444239 | 0.916971445 | 1.0432719 | down | hsa_circRNA_003943 | hsa_circ_0003943 | circBase | chr7 | - | 6854394 | 6862991 | exonic | NM_198097 | CCZ1B | CAAATACCTTACCACCTCCCTTTTCCCAAGGCACATCGAACCTGAGGACAAGGTTTATAG |
| ASCRP3003474 | 0.409065345 | 0.91119147 | 1.1009048 | down | hsa_circRNA_104297 | hsa_circ_0004703 | circBase | chr7 | + | 5941287 | 5949747 | exonic | NM_015622 | CCZ1 | CAAATACCTTACCACCTCCCTTTTTCCAAGGCACATCGAACCTGAGGACAAGGTTTATAG |
| ASCRP3003485 | 0.050224098 | 0.909237851 | 1.1036081 | down | hsa_circRNA_103442 | hsa_circ_0003763 | circBase | chr3 | - | 119562101 | 119582452 | exonic | NM_002093 | GSK3B | CAAATGCCACAGCAGCGTCAGGTCTTCCGACCCCGAACTCCACCGGAGGCAATTGCACTG |
| ASCRP3003494 | 0.244185034 | 0.909237851 | 1.0355471 | down | hsa_circRNA_405534 | | 25070500 | chr17 | + | 7402357 | 7402812 | sense overlapping | NM_000937 | POLR2A | CAAATTCACCAAGAGAGACGTCTTCCTGGAGCGGGTGTGGAACGGCACATGTGTGATGGG |
| ASCRP3003502 | 0.558916451 | 0.920978531 | 1.0150347 | down | hsa_circRNA_038669 | hsa_circ_0038669 | circBase | chr16 | + | 24572932 | 24576186 | exonic | NM_006910 | RBBP6 | CAACAAATTAGAAGAGGGGAGAGGAGCTGCTACAGGTATAAGAACAGCACTCCTGGAATC |
| ASCRP3003511 | 0.888236664 | 0.979863126 | 1.0054757 | down | hsa_circRNA_101657 | hsa_circ_0005035 | circBase | chr15 | + | 99250790 | 99251336 | exonic | NM_000875 | IGF1R | CAACAATGAGTACAACTACCGCTGCTGGACCACAAACCGCTGCCAGAAAATCTGCGGGCC |
| ASCRP3003521 | 0.900920154 | 0.981257955 | 1.0047494 | down | hsa_circRNA_100744 | hsa_circ_0020913 | circBase | chr11 | - | 3789810 | 3789974 | exonic | NM_005387 | NUP98 | CAACACCATGGTACAACTGGATTTGGAACAAATCCAGGTGGTCTCTTTGGCCAACAGAAT |
| ASCRP3003524 | 0.569432395 | 0.92124149 | 1.0418574 | down | hsa_circRNA_028595 | hsa_circ_0028595 | circBase | chr12 | + | 117178149 | 117178899 | exonic | NM_032814 | RNFT2 | CAACACGGATAACATTCCACCTGAAAGGTTTGGAGTCTCTGGCAAGCTCCCCTGACTGTG |
| ASCRP3003527 | 0.263103694 | 0.909237851 | 1.1033622 | down | hsa_circRNA_104775 | hsa_circ_0001855 | circBase | chr9 | - | 36375930 | 36376124 | exonic | NM_022781 | RNF38 | CAACAGAAGAAGAGTGAAGATAGTCCAAGTCCTAAGAGACAGCGCCTCTCTCATTCAGTC |
| ASCRP3003528 | 0.010733917 | 0.909237851 | 1.1119172 | down | hsa_circRNA_001856 | hsa_circ_0001856 | circBase | chr9 | - | 36375930 | 36390613 | exonic | NM_022781 | RNF38 | CAACAGAAGAAGATATCTCCCGGGGCCAATTCAGCATCTCTACCTGGCCATCCTAACAAG |
| ASCRP3003529 | 0.730981001 | 0.952599775 | 1.0468484 | down | hsa_circRNA_029830 | hsa_circ_0029830 | circBase | chr13 | + | 28712642 | 28835595 | exonic | NM_175854 | PAN3 | CAACAGACATTTAATAACAATGGCTCAAATTGATCAAGCAGATATGCCAGCTCCCCGGGC |
| ASCRP3003531 | 0.113139298 | 0.909237851 | 1.0829699 | down | hsa_circRNA_073270 | hsa_circ_0073270 | circBase | chr5 | - | 88024309 | 88047860 | exonic | NM_002397 | MEF2C | CAACAGCAACACCTACATAACATGCCACCATCTGCCCTCAGTCAGTTGGGGCTGTTCCAC |
| ASCRP3003535 | 0.173917578 | 0.909237851 | 1.0467797 | down | hsa_circRNA_100145 | hsa_circ_0011377 | circBase | chr1 | + | 32689634 | 32690076 | exonic | NM_003757 | EIF3I | CAACAGCTGTCGTCTCTGGGACTGTGAAACAGATCGTCAATGTATGGTACTCTGTGAATG |
| ASCRP3003544 | 0.606993622 | 0.929544944 | 1.0709745 | down | hsa_circRNA_007630 | hsa_circ_0007630 | circBase | chr3 | + | 152163070 | 152165562 | exonic | NM_021038 | MBNL1 | CAACATGCAGTTACAACAGCATACAGCATTTCTCCCACCAGGTATGTCGAGAGTACCAAC |
| ASCRP3003546 | 0.816371515 | 0.976440085 | 1.0172618 | down | hsa_circRNA_005528 | hsa_circ_0005528 | circBase | chr2 | + | 87114895 | 87115116 | exonic | NR_037931 | ANAPC1P1 | CAACATGGCAGCTCTAAGGAAGAGAATGTTGTTTTAAAGTTCTCTGAACATGGGGGAACC |
| ASCRP3003555 | 0.418507264 | 0.91119147 | 1.050002 | down | hsa_circRNA_405528 | | 25070500 | chr17 | - | 3524281 | 3524706 | sense overlapping | NM_013276 | SHPK | CAACCATCTGTGCTGACCACCTCTGGGGCTGGACTGAGGAGCTCGGGTTTTCCTGTCCAC |
| ASCRP3003564 | 0.903897948 | 0.981382356 | 1.0044405 | down | hsa_circRNA_100091 | hsa_circ_0002402 | circBase | chr1 | - | 21599191 | 21616649 | exonic | NM_001397 | ECE1 | CAACCTCTGGGAACACAACCAAGCAATCATCAAGCACCTCCTCGATGTCGACGTACAAGC |
| ASCRP3003567 | 0.877284468 | 0.979863126 | 1.0166456 | down | hsa_circRNA_026813 | hsa_circ_0026813 | circBase | chr12 | + | 56481360 | 56482652 | exonic | NM_001982 | ERBB3 | CAACCTGGACTTTCTGATCACCGGCCTCAATGGGTCCCCCCTGTCATGAGGTTTGCAAGG |
| ASCRP3003570 | 0.069843218 | 0.909237851 | 1.0611901 | down | hsa_circRNA_003540 | hsa_circ_0003540 | circBase | chr3 | - | 65415194 | 65428524 | exonic | NM_004742 | MAGI1 | CAACGAGGAGAACTGCCTGCTGGTTGGGAAAAGATTGAAGACCCTGTCTATGGTATCTAC |
| ASCRP3003587 | 0.992781277 | 0.998961292 | 1.0007568 | down | hsa_circRNA_002561 | hsa_circ_0002561 | circBase | chr3 | - | 49094294 | 49095323 | exonic | NM_017730 | QRICH1 | CAACTCCAAGTTACTTGTTCAGTCCAGGTGCAGGTACAGCAGTCTCCGCAACAGGTCTCG |
| ASCRP3003590 | 0.720042944 | 0.949318546 | 1.0089452 | down | hsa_circRNA_017235 | hsa_circ_0017235 | circBase | chr1 | + | 243456392 | 243542165 | exonic | NM_006642 | SDCCAG8 | CAACTGCACCTCACCAGGGAAACATGCACAATTCTTGGATTACAACAGGTGAAGATTCTG |
| ASCRP3003595 | 0.186274218 | 0.909237851 | 1.0872662 | down | hsa_circRNA_104663 | hsa_circ_0085133 | circBase | chr8 | - | 101716524 | 101717901 | exonic | NM_002568 | PABPC1 | CAACTGTTTAAACCTGCTGTTCATGTACAAGGTCAGGAACCTTTGACTGCTTCCATGTTG |
| ASCRP3003600 | 0.183738923 | 0.909237851 | 1.0366234 | down | hsa_circRNA_100439 | hsa_circ_0016343 | circBase | chr1 | - | 210948689 | 210977508 | exonic | NM_002238 | KCNH1 | CAACTTGAGGAAGAGGCACTTCTCTATGCCACCATCTTCGGGAATGTGACGACTATTTTC |
| ASCRP3003607 | 0.006077497 | 0.879037174 | 1.1095586 | down | hsa_circRNA_100472 | hsa_circ_0016760 | circBase | chr1 | + | 227935392 | 227947186 | exonic | NM_053052 | SNAP47 | CAAGAACCTCTTCCCCCGCAGAGAAGAGCTGCTCAGTCTGGCATGCAGAGGCAGAAGAGG |
| ASCRP3003621 | 0.074406957 | 0.909237851 | 1.074622 | down | hsa_circRNA_102948 | hsa_circ_0058753 | circBase | chr2 | + | 234098495 | 234102607 | exonic | NM_005541 | INPP5D | CAAGACGAGGGAGAAGCTCTATGCTGAAGCCCATTATCTCTGACCCTGAGTACCTGCTAG |
| ASCRP3003623 | 0.197202534 | 0.909237851 | 1.042577 | down | hsa_circRNA_004407 | hsa_circ_0004407 | circBase | chr21 | + | 38792600 | 38798083 | intronic | ENST00000462274 | DYRK1A | CAAGACTAGGTGAACAGTATCCCCACTGACTCACATACAATGTTATAGTTTTGCCGCTGG |
| ASCRP3003629 | 0.025092468 | 0.909237851 | 1.0785531 | down | hsa_circRNA_045309 | hsa_circ_0045309 | circBase | chr17 | + | 62515438 | 62525545 | exonic | NM_138363 | CEP95 | CAAGAGAAACAGATGTCCGCCAATTCCAAGCACAGGTGCTCCTTGTCTTCTGAGATGTTG |
| ASCRP3003634 | 0.310806024 | 0.909237851 | 1.0572409 | down | hsa_circRNA_407264 | | 25070500 | chrX | - | 6995260 | 7003512 | intronic | uc010ndl.2 | HDHD1 | CAAGAGGTTCTCTCCCCCTCCTGCTATGGAGAAGGGCATCAGCTCTTCACACAGCAACAG |
| ASCRP3003635 | 0.959646245 | 0.993354918 | 1.0041001 | down | hsa_circRNA_103994 | hsa_circ_0074736 | circBase | chr5 | - | 154291286 | 154292598 | exonic | NM_015465 | GEMIN5 | CAAGATCATTCCCGGCCTCCTCAAGAGAGCAGCCCTGAGTCTCCAGTGACCATTACAGAG |
| ASCRP3003636 | 0.060090934 | 0.909237851 | 1.0950135 | down | hsa_circRNA_001606 | hsa_circ_0001606 | circBase | chr6 | - | 42980912 | 42981094 | sense overlapping | NM_014623 | MEA1 | CAAGATCCTGAACAAGAGGAGGAGACACCATGGGCCCTGAGCGTATCTTCCCCAATCAGA |
| ASCRP3003639 | 0.141980702 | 0.909237851 | 1.0453294 | down | hsa_circRNA_102971 | hsa_circ_0004924 | circBase | chr2 | + | 242343242 | 242357524 | exonic | NM_014808 | FARP2 | CAAGATTCAACTGGCAGTTTCCCACATGGGTGTACTCGTGTTCCAGCCTAAATGCGATGG |
| ASCRP3003663 | 0.364226005 | 0.91119147 | 1.0427858 | down | hsa_circRNA_102760 | hsa_circ_0055161 | circBase | chr2 | - | 72361889 | 72362548 | exonic | NM_019885 | CYP26B1 | CAAGGAGCACGGGAAGGAGATGACCATGCAGGAGCTGAAGGTCTTCTCCAAGATCTTCAG |
| ASCRP3003664 | 0.490720701 | 0.91119147 | 1.0713665 | down | hsa_circRNA_100691 | hsa_circ_0020117 | circBase | chr10 | - | 118464661 | 118466796 | exonic | NM_025015 | HSPA12A | CAAGGAGCCGGAATGCATCCATGTGATGAGAAACGGCTCCCACATCTGCATATTCATCTC |
| ASCRP3003667 | 0.232890203 | 0.909237851 | 1.0340059 | down | hsa_circRNA_100104 | hsa_circ_0000034 | circBase | chr1 | + | 26772806 | 26774151 | exonic | NM_024887 | DHDDS | CAAGGCCTGTTGGATCCCAGGGAGAAACTGCAGAAGCATGGGGTGTGTATCCGGGTCCTG |
| ASCRP3003669 | 0.627904866 | 0.929544944 | 1.0162718 | down | hsa_circRNA_104915 | hsa_circ_0088485 | circBase | chr9 | + | 127064214 | 127076264 | exonic | NM_014397 | NEK6 | CAAGGCGAGGCAGGACTGTGTCAAGGAGATCGGCCTCTTGAAGTTCGTGCCCTCGTGAGG |
| ASCRP3003671 | 0.062532364 | 0.909237851 | 1.1245519 | down | hsa_circRNA_020094 | hsa_circ_0020094 | circBase | chr10 | + | 116879948 | 116925405 | exonic | NM_207303 | ATRNL1 | CAAGGGGACCTCTCCAGAGATATGGACACTCTCTTGCTTTATATCAGGTTAACAGAACCT |
| ASCRP3003672 | 0.590610306 | 0.927905266 | 1.0185213 | down | hsa_circRNA_104955 | hsa_circ_0089392 | circBase | chr9 | - | 136671202 | 136677338 | exonic | NM_003371 | VAV2 | CAAGGTCTTCCTCGATTTCAAGGAAAGCGAGCATGACCTGGGGGAGGACATCTACGACTG |
| ASCRP3003687 | 0.073788836 | 0.909237851 | 1.0409215 | down | hsa_circRNA_089986 | hsa_circ_0089986 | circBase | chrX | - | 18917290 | 18923977 | exonic | NM_000292 | PHKA2 | CAAGTCTGCGGAGAAGAGGCTTCTGAAAGTTTGATGAACCTCAGCCCTTTCGATATGAAA |
| ASCRP3003692 | 0.510765073 | 0.916805276 | 1.0417679 | down | hsa_circRNA_100205 | hsa_circ_0012265 | circBase | chr1 | - | 46105881 | 46120991 | exonic | NM_021639 | GPBP1L1 | CAAGTGTCTATAAGAACCTGGTTCCTAAGCCTGTACCACCTCCTTCCAAGTCACCTACTG |
| ASCRP3003702 | 0.518556475 | 0.916971445 | 1.0411311 | down | hsa_circRNA_074657 | hsa_circ_0074657 | circBase | chr5 | - | 151122382 | 151122520 | exonic | NM_004045 | ATOX1 | CAATAAAATCAAGCTGCTTTTGTTGGAGATGCTGATCCTCCCGCTGGCTTCCAGACAGAC |
| ASCRP3003709 | 0.189307111 | 0.909237851 | 1.0603616 | down | hsa_circRNA_086159 | hsa_circ_0086159 | circBase | chr8 | - | 146015153 | 146017525 | exonic | NM_000973 | RPL8 | CAATAAAGTTTGTGTTTATGCCAGACCCGTCGCCATGGGCCGTGTGATCCGTGGACAGAG |
| ASCRP3003713 | 0.034080387 | 0.909237851 | 1.1311721 | down | hsa_circRNA_406022 | | 25070500 | chr2 | - | 202208892 | 202216174 | exonic | NM_139163 | ALS2CR12 | CAATAATTGAACAAATGAACAGAGACCACCAGTCTGCCCAGAAATTGCAAGAGCCTCCTG |
| ASCRP3003719 | 0.170903103 | 0.909237851 | 1.0517134 | down | hsa_circRNA_406891 | | 25070500 | chr6 | + | 158703294 | 158735300 | sense overlapping | NM_020245 | TULP4 | CAATAGCGAGTCCTGGGTCAAGCTGAGTATCTCCACGTTTAGGTGCACAAATACTATTTT |
| ASCRP3003721 | 0.641153951 | 0.929544944 | 1.015356 | down | hsa_circRNA_103240 | hsa_circ_0007704 | circBase | chr22 | + | 41721567 | 41721922 | exonic | NM_017590 | ZC3H7B | CAATAGGGCCGCCTGCTACTTCACCATGGTCGACACTACCCCTAAAGCAAGAAGAATATG |
| ASCRP3003738 | 0.161713804 | 0.909237851 | 1.1511006 | down | hsa_circRNA_012592 | hsa_circ_0012592 | circBase | chr1 | - | 53732144 | 53737018 | exonic | NM_004631 | LRP8 | CAATCGCATCTACTGGTGTGACCTCTCCTACCGTAAGATCTATAGGGCTGAACGAGTGTC |
| ASCRP3003745 | 0.130269561 | 0.909237851 | 1.0505851 | down | hsa_circRNA_405999 | | 25070500 | chr2 | - | 179334378 | 179337869 | exonic | uc002uml.3 | FKBP7 | CAATGACAGGCAGCTCTCTAAAGCCGAGGTAGTACTTGAAGAAGTCTTTCTTCTGCAAAA |
| ASCRP3003755 | 0.874805561 | 0.979863126 | 1.0296718 | down | hsa_circRNA_102299 | hsa_circ_0046882 | circBase | chr18 | - | 9588086 | 9595100 | exonic | NM_005134 | PPP4R1 | CAATGATGATGTGAAAACAGAAGCTGTGGCTCCCTGGACTTTGTCTCACAAGATGAAATG |
| ASCRP3003758 | 0.228124779 | 0.909237851 | 1.0344275 | down | hsa_circRNA_404499 | | 25070500 | chr1 | - | 45516745 | 45553909 | exonic | NM_020883 | ZSWIM5 | CAATGCACTTCCCCAGAGTGCCATTCACAGCCCAGGCTTTCATCTGAGTGGCACAGTAAC |
| ASCRP3003761 | 0.606223989 | 0.929544944 | 1.025413 | down | hsa_circRNA_104339 | hsa_circ_0079675 | circBase | chr7 | + | 28758369 | 28763949 | exonic | NM_004904 | CREB5 | CAATGCATTCAGAAGCCAAAATGGCCTGTCCCAGGCTCTCTATCTTCTCTGCTACATCTC |
| ASCRP3003767 | 0.211396923 | 0.909237851 | 1.0326325 | down | hsa_circRNA_405448 | | 25070500 | chr16 | - | 17448955 | 17451907 | sense overlapping | NM_022166 | XYLT1 | CAATGGCACAAGGATCCACACCCAAGTCCGCTCATCACCCTGGAGACTCAGGTAAGTATC |
| ASCRP3003781 | 0.248895871 | 0.909237851 | 1.1411216 | down | hsa_circRNA_404891 | | 25070500 | chr11 | - | 64760944 | 64762021 | sense overlapping | NM_138456 | BATF2 | CAATTCAGTTCCAGATGAGGGTGCTCTTCCTGACTTGACCCCAAGGAGCAACAAAGGCAG |
| ASCRP3003795 | 0.604866024 | 0.929544944 | 1.0160979 | down | hsa_circRNA_101592 | hsa_circ_0036287 | circBase | chr15 | + | 74911537 | 74912566 | exonic | NM_003992 | CLK3 | CACAAACGCCGCACCAGGTCTTGTAGCAGCGCCTCCTCGATGCATCACTGTAAGCGATAC |
| ASCRP3003798 | 0.535296503 | 0.919549314 | 1.0182529 | down | hsa_circRNA_076313 | hsa_circ_0076313 | circBase | chr6 | + | 41757268 | 41757634 | exonic | NM_001134493 | TOMM6 | CACAAAGGCAGTTCTAATGCCACCTGTCGTCTTATCATCTGATTGCAGACAAATGGAATC |
| ASCRP3003800 | 0.183946741 | 0.909237851 | 1.1658514 | down | hsa_circRNA_407287 | | 25070500 | chrX | - | 51070572 | 51099879 | sense overlapping | ENST00000425150 | RP11-348F1.3 | CACAAATCATGGATGTGGTCCCAGTGATCCATGGAAATGTGAAGTCCAGGAGTGGCTGTG |
| ASCRP3003808 | 0.792529817 | 0.967847019 | 1.0135112 | down | hsa_circRNA_101595 | hsa_circ_0000633 | circBase | chr15 | - | 74963795 | 74967483 | exonic | NM_025083 | EDC3 | CACAACTCCTTGGTCTCACGTGTGAAACATGGCTACAGATTGGCTGGGAAGTATTGTGTC |
| ASCRP3003812 | 0.614083188 | 0.929544944 | 1.0293506 | down | hsa_circRNA_001680 | hsa_circ_0001680 | circBase | chr7 | + | 18624903 | 18633652 | exonic | NM_014707 | HDAC9 | CACAAGATGCAAAGGATGATTTCCCCCTTCGAAAAACTGTGGATGTGAAGTCAGAAGTTC |
| ASCRP3003813 | 0.546763156 | 0.919549314 | 1.0167546 | down | hsa_circRNA_403189 | | 25242744 | chr4 | + | 99955380 | 99970027 | exonic | NM_015143 | METAP1 | CACAAGCTTTTTCATACAGCTCCCAATGTACCCCACTATGCTAAAGATGAAAAGGCGAAG |
| ASCRP3003824 | 0.83891408 | 0.978181218 | 1.0193526 | down | hsa_circRNA_403650 | | 25242744 | chr6 | + | 84765030 | 84772711 | exonic | NM_138409 | MRAP2 | CACACCAAGAGTCGGAGATGTCCGCCCAGAGGTTAATTTCTAACAGAACCTCCCAGCAAT |
| ASCRP3003831 | 0.938389262 | 0.988165332 | 1.0050845 | down | hsa_circRNA_405086 | | 25070500 | chr12 | + | 122669065 | 122670847 | exonic | NM_152759 | LRRC43 | CACACTCAAGAATAAGTCGCGCTTTCTTCCTCAAACTTGGCGAACTTGGAGGGAGCTTGT |
| ASCRP3003833 | 0.535415387 | 0.919549314 | 1.0250839 | down | hsa_circRNA_092520 | hsa_circ_0001240 | circBase | chr22 | - | 42807412 | 42807742 | exonic | NM_145912 | NFAM1 | CACACTCCACGGTGAGAGGCAGCGGCACCTTCATCCTGGTCAGAGGAGGACAGTCAGTGA |
| ASCRP3003844 | 0.551611205 | 0.919549314 | 1.0216898 | down | hsa_circRNA_100302 | hsa_circ_0013587 | circBase | chr1 | + | 113661854 | 113662145 | exonic | NM_014813 | LRIG2 | CACAGATGAGCGGTGGTGGCACTGGTACCCGGGTGATTTGCTCAGATTGTTATGACAATG |
| ASCRP3003847 | 0.25929355 | 0.909237851 | 1.035202 | down | hsa_circRNA_101184 | hsa_circ_0028944 | circBase | chr12 | + | 121659715 | 121659969 | exonic | NM_002560 | P2RX4 | CACAGCAACGGAGGAAAACTCCCTCTTCGTCATGACCAACGTGATCCTCACCATGAACCA |
| ASCRP3003854 | 0.036298038 | 0.909237851 | 1.0562367 | down | hsa_circRNA_064552 | hsa_circ_0064552 | circBase | chr3 | - | 17418016 | 17550097 | exonic | NM_014744 | TBC1D5 | CACAGCCCAGGTAGTTACTTTTGGTGACGCTGTCCAGTTCCCACAATGTATCATTCCTTA |
| ASCRP3003855 | 0.064640405 | 0.909237851 | 1.1748903 | down | hsa_circRNA_019875 | hsa_circ_0019875 | circBase | chr10 | - | 105382226 | 105496624 | exonic | uc010qqu.1 | SH3PXD2A | CACAGCCGAGGACACCACAAGGACTGGGAGTGGAAGATGCCTTCTCCAGGGGGAAATTAG |
| ASCRP3003860 | 0.935823655 | 0.987920247 | 1.0042009 | down | hsa_circRNA_001041 | hsa_circ_0001041 | circBase | chr2 | - | 88874183 | 88913371 | exonic | NM_004836 | EIF2AK3 | CACAGGGACCTCAAGGTCATTAGTAATTATCAGCACTTTAGATGGGAGAATTGCTGCCTT |
| ASCRP3003866 | 0.006647263 | 0.879037174 | 1.0704019 | down | hsa_circRNA_100441 | hsa_circ_0007234 | circBase | chr1 | + | 211526580 | 211527809 | exonic | NM_004619 | TRAF5 | CACAGTGCCAATCTGCCCTGTAGATAAAGAGGTCATCAAATCTCAGGAGAATGGCTTATT |
| ASCRP3003868 | 0.346742679 | 0.91119147 | 1.0370056 | down | hsa_circRNA_062547 | hsa_circ_0062547 | circBase | chr22 | - | 24025911 | 24029182 | exonic | uc002zxh.4 | GUSBP11 | CACAGTGGACTCCCAAGGGGCAGAAAATAAAGCATGCGAAGAAGTTAACTTGGTGCCATT |
| ASCRP3003878 | 0.936685673 | 0.987920247 | 1.0028458 | down | hsa_circRNA_400048 | hsa_circ_0092327 | circBase | chr19 | + | 17972481 | 17972901 | intronic | ENST00000222247 | RPL18A | CACATCGGCTTGCTGCTCTCACATCTCCCTGTGGCCTCTCCTTGGCATTAGAAAGCCAGG |
| ASCRP3003886 | 0.046891476 | 0.909237851 | 1.1437945 | down | hsa_circRNA_000956 | hsa_circ_0001489 | circBase | chr5 | + | 59770958 | 59771235 | antisense | NM_001165899 | PDE4D | CACATGTAGTTATAGCCAGAAGAGCCAGGTCTTCTCCCCCCTAATGTTATGCTCCCTAAG |
| ASCRP3003900 | 0.068241614 | 0.909237851 | 1.0657505 | down | hsa_circRNA_011480 | hsa_circ_0011480 | circBase | chr1 | - | 33794470 | 33836186 | exonic | NM_198040 | PHC2 | CACCAAGTGGAATGTAGAAGACGTCTACGAATTCATCCGCTCTCTGCCAGATCAACCTGG |
| ASCRP3003901 | 0.139498991 | 0.909237851 | 1.0637493 | down | hsa_circRNA_011477 | hsa_circ_0011477 | circBase | chr1 | - | 33794470 | 33815499 | exonic | NM_004427 | PHC2 | CACCAAGTGGAATGTAGAAGACGTCTACGAATTCATCCGCTCTCTGCCAGCATTGTCTCC |
| ASCRP3003909 | 0.588634901 | 0.927905266 | 1.0657055 | down | hsa_circRNA_104710 | hsa_circ_0006421 | circBase | chr8 | - | 141889569 | 141935848 | exonic | NM_153831 | PTK2 | CACCAGAGGAGTGGAAGCAGAACTGGGGCTCCCTTGCATCTTCCAGTTACAAATTCAGTG |
| ASCRP3003915 | 0.574479205 | 0.922626849 | 1.0262232 | down | hsa_circRNA_004226 | hsa_circ_0004226 | circBase | chr9 | + | 22046749 | 22066352 | exonic | NR_003529 | CDKN2B-AS1 | CACCATGCATGTGTCCCTTTTGATGAGAAGAATAAGCCTCATTCTGATTCAACAGCAGAG |
| ASCRP3003922 | 0.485954831 | 0.91119147 | 1.0494326 | down | hsa_circRNA_400540 | | 25242744 | chr10 | - | 30611319 | 30630569 | exonic | NM_018109 | MTPAP | CACCCCCTATTCTTCCAACACTAGATTCCTTAAAAACCCTAGCAGGCTTTGAAGACAAGA |
| ASCRP3003929 | 0.508930683 | 0.916805276 | 1.0271762 | down | hsa_circRNA_063179 | hsa_circ_0063179 | circBase | chr22 | + | 37328806 | 37330036 | exonic | NM_000395 | CSF2RB | CACCGAGTCGGTCCAGATGGCCCCTCCATCCCTCAACGTGACCAAGGATGGAGACAGCTA |
| ASCRP3003930 | 0.91338349 | 0.983418505 | 1.0071768 | down | hsa_circRNA_100385 | hsa_circ_0004370 | circBase | chr1 | + | 170688866 | 170695542 | exonic | NM_006902 | PRRX1 | CACCGATTATCTCTCCTGGGGGACAGCGTCTCCGTACAGATGACCAGCTGAACTCAGAAG |
| ASCRP3003932 | 0.088951489 | 0.909237851 | 1.0841744 | down | hsa_circRNA_000289 | hsa_circ_0000289 | circBase | chr11 | - | 34489906 | 34493056 | antisense | NM_001752 | CAT | CACCGCTTTCTTCTGGATGAAAATTTGTGCATCCTTCAGGAGAATCTTCATCCAGTGATG |
| ASCRP3003937 | 0.614845734 | 0.929544944 | 1.0288095 | down | hsa_circRNA_045556 | hsa_circ_0045556 | circBase | chr17 | - | 72517312 | 72522216 | exonic | NM_174892 | CD300LB | CACCGTTTCTTCCTGTTGATATTCAATGAATCCGTCAATCTCTCTGGAGCTGTTTCTCCA |
| ASCRP3003941 | 0.253802544 | 0.909237851 | 1.0403206 | down | hsa_circRNA_036592 | hsa_circ_0036592 | circBase | chr15 | + | 85180577 | 85181708 | exonic | NR_003654 | SCAND2P | CACCTAGAAATAACTCAGCAGAATTCTGAAAATGAGGTCACGCTGGCCCTGCCTGATGTG |
| ASCRP3003942 | 0.991651566 | 0.998490743 | 1.0007479 | down | hsa_circRNA_406419 | | 25070500 | chr3 | + | 195435004 | 195435712 | exonic | NR_122105 | LINC00969 | CACCTAGACTGGATGCAGAGCATGGCAGCTGCTCCCAACCCCAGAACCTTTGCTGTCTTG |
| ASCRP3003943 | 0.947554049 | 0.992077624 | 1.004075 | down | hsa_circRNA_101138 | hsa_circ_0028135 | circBase | chr12 | + | 109926376 | 109945540 | exonic | NM_130466 | UBE3B | CACCTCATCACCCTGAAATCCTGCAGGACTCCCGACTCATCACCCTGTACCTCACGATGC |
| ASCRP3003958 | 0.968775624 | 0.995698232 | 1.0033552 | down | hsa_circRNA_402116 | | 25242744 | chr19 | + | 46832463 | 46834530 | exonic | NM_022462 | HIF3A | CACGAAAACTTTCTGCTCTTTCCTCTCAGCCTGGATGCTGATGCTCTGGATTTGGAGATG |
| ASCRP3003961 | 0.399283104 | 0.91119147 | 1.012929 | down | hsa_circRNA_104429 | hsa_circ_0081308 | circBase | chr7 | + | 98562251 | 98569568 | exonic | NM_003496 | TRRAP | CACGACAAGTTTCTGGACACTCTCCGAGAGGTGAAGGGACCCTTATGATCCTCAAGTCTG |
| ASCRP3003965 | 0.476147376 | 0.91119147 | 1.0285432 | down | hsa_circRNA_405794 | | 25070500 | chr19 | + | 47767862 | 47768203 | sense overlapping | NM_015603 | CCDC9 | CACGAGCGGCAGTGGGAGGAGCGGCGCAGGCAGAACATTGAGAAGATGAATGAGGAGATG |
| ASCRP3003972 | 0.957917466 | 0.993354918 | 1.0013247 | down | hsa_circRNA_092457 | hsa_circ_0000868 | circBase | chr19 | - | 2428562 | 2434034 | sense overlapping | NM_032737 | LMNB2 | CACGCCTGCTGGACCAACGCGGGTTCTGGGGTGCACAGCGCCAGCTTGTCCGCCACCGGG |
| ASCRP3003976 | 0.329399411 | 0.909237851 | 1.0778293 | down | hsa_circRNA_102241 | hsa_circ_0046435 | circBase | chr17 | + | 80540616 | 80545148 | exonic | NM_004514 | FOXK2 | CACGGCCAGGTGAACAATGAATTCAATTCGCCACAATCTCTCTCTGAATCGTTATTTCAT |
| ASCRP3003980 | 0.497988277 | 0.913224439 | 1.0324531 | down | hsa_circRNA_402103 | | 25242744 | chr19 | + | 41704366 | 41704793 | exonic | NM_030622 | CYP2S1 | CACGTGACCTTGTCGATGCCTTCCTGCTGAAGATGGCACAGGACGCCCATTCGATCCCTC |
| ASCRP3003985 | 0.034217967 | 0.909237851 | 1.1004379 | down | hsa_circRNA_009541 | hsa_circ_0009541 | circBase | chr1 | + | 6880240 | 6885270 | exonic | NM_015215 | CAMTA1 | CACTAATGAGAGCGTTTCCCAAAGTGTATTCTGCGGAACTAGCACCTACTGTGTTCTCAA |
| ASCRP3003986 | 0.911118282 | 0.983376499 | 1.0047586 | down | hsa_circRNA_021555 | hsa_circ_0021555 | circBase | chr11 | - | 30601792 | 30607930 | exonic | NM_001145399 | MPPED2 | CACTACAACATCAACCAGAGCAGATTCCAGCCTCCACATGTACATATGCTCCTTTGACCC |
| ASCRP3003993 | 0.177188402 | 0.909237851 | 1.0613113 | down | hsa_circRNA_000276 | hsa_circ_0000276 | circBase | chr11 | - | 5711031 | 5717503 | intronic | ENST00000380027 | TRIM5 | CACTCACCAAGCCAGAGGGCTGGTCACCTCCTTCTCTATGTCTACCTTTACTGAGAAATC |
| ASCRP3004002 | 0.244599423 | 0.909237851 | 1.0243244 | down | hsa_circRNA_406395 | | 25070500 | chr3 | + | 172477945 | 172482290 | exonic | NM_018098 | ECT2 | CACTCCTAGATATCTCCAACACACCAGAGTCTAGCATTAACTATGGAGGTCAGGTTGGTG |
| ASCRP3004006 | 0.073049328 | 0.909237851 | 1.0863057 | down | hsa_circRNA_000887 | hsa_circ_0000887 | circBase | chr19 | + | 8615158 | 8615503 | antisense | NM_012335 | MYO1F | CACTCGGGCGTAATTCCCGTCTTCACAGAAACTGATGTTCCCCAGCGGCTGTCCATCTTG |
| ASCRP3004012 | 0.893385061 | 0.98039597 | 1.0062553 | down | hsa_circRNA_001156 | hsa_circ_0001156 | circBase | chr20 | + | 37547116 | 37547282 | sense overlapping | NM_015568 | PPP1R16B | CACTCTTAAAGTTCAAGGCCCCCATAGAGGAGATGGAGGAGAAGCAGCATGGCCAGGACG |
| ASCRP3004014 | 0.274497785 | 0.909237851 | 1.0389441 | down | hsa_circRNA_100537 | hsa_circ_0002665 | circBase | chr10 | - | 5836847 | 5842668 | exonic | NM_001494 | GDI2 | CACTGAAGCAGAAGCCCTGGCATCTAGAATGTATCCTGTCAGGTATAATGTCAGTGAATG |
| ASCRP3004018 | 0.285242242 | 0.909237851 | 1.0796179 | down | hsa_circRNA_008689 | hsa_circ_0008689 | circBase | chr4 | + | 20469374 | 20493522 | exonic | NM_004787 | SLIT2 | CACTGAGATCCCCACAAATCTTCCAGAGACCATCACAGAAATTGATCTCAGTGAAAACCA |
| ASCRP3004022 | 0.720198136 | 0.949318546 | 1.0219138 | down | hsa_circRNA_013552 | hsa_circ_0013552 | circBase | chr1 | - | 113243748 | 113249678 | exonic | NM_001042679 | RHOC | CACTGCATTTGGTCAGGGGGGCATGAATAAAGGCTACAGGCTCCAACGTGGGCGGTGGGG |
| ASCRP3004029 | 0.873405505 | 0.979863126 | 1.0066592 | down | hsa_circRNA_100360 | hsa_circ_0005758 | circBase | chr1 | - | 155891165 | 155893478 | exonic | NM_014949 | KIAA0907 | CACTGGACAGTCACCCCAAACCAGAAGGCCTGGCTGCTGCCAAGAAGCTTTGTGAGAATC |
| ASCRP3004036 | 0.150267506 | 0.909237851 | 1.0476724 | down | hsa_circRNA_101626 | hsa_circ_0036610 | circBase | chr15 | + | 85607600 | 85610435 | exonic | NM_002605 | PDE8A | CACTGTGCAGGTAGCAGTAGCTGATGTGCAGTTTGGCCCCATGAGATTTCATCAAGATCA |
| ASCRP3004040 | 0.90803871 | 0.982651988 | 1.0159731 | down | hsa_circRNA_400093 | hsa_circ_0092290 | circBase | chr8 | - | 144891235 | 144891535 | intronic | ENST00000320476 | SCRIB | CACTGTTTAGTAGGGTCTTCCTGTGGAAGCACAGGAGCTGCCTCCCTAGGTGGGGTTAAG |
| ASCRP3004042 | 0.859080667 | 0.97947761 | 1.0052129 | down | hsa_circRNA_026768 | hsa_circ_0026768 | circBase | chr12 | - | 54801432 | 54802019 | exonic | NM_002205 | ITGA5 | CACTTACGGCTATGCCAGATCCTGTCTGCCACTCAGGAGCAGATTGCAGAATCTTATTAC |
| ASCRP3004045 | 0.984749852 | 0.998148217 | 1.001089 | down | hsa_circRNA_009213 | hsa_circ_0009213 | circBase | chr1 | + | 957580 | 970704 | exonic | NM_198576 | AGRN | CACTTCACTCCAGTGCCTCCGACGCCTCCTGATGGTTCGGGTCTGGCGGTACTTGAAGGG |
| ASCRP3004046 | 0.836441753 | 0.978181218 | 1.009303 | down | hsa_circRNA_100835 | hsa_circ_0022502 | circBase | chr11 | - | 62459847 | 62460269 | exonic | NM_032667 | BSCL2 | CACTTCACTGGGCTCAGGTGATGCTGCATTACCGCTCAGACCTGCTCCAGATGCTGGACA |
| ASCRP3004067 | 0.361682579 | 0.91119147 | 1.0592671 | down | hsa_circRNA_100182 | hsa_circ_0005157 | circBase | chr1 | + | 41474465 | 41475261 | exonic | NM_001905 | CTPS1 | CAGAAAGGCTGCAGGCTCTCACCCAGGTGAATCCAGTCTGGAAAAAGTGTTTGGAAGAAC |
| ASCRP3004068 | 0.32155188 | 0.909237851 | 1.0187452 | down | hsa_circRNA_102720 | hsa_circ_0005045 | circBase | chr2 | - | 55214626 | 55214834 | exonic | NM_007008 | RTN4 | CAGAAATCAGATGAAGGCCACCCATTCAGTTGTTGACCTCCTGTACTGGAGAGACATTAA |
| ASCRP3004070 | 0.951201389 | 0.992077624 | 1.0031268 | down | hsa_circRNA_405985 | | 25070500 | chr2 | - | 166727312 | 166727530 | sense overlapping | NR_045375 | LOC100506124 | CAGAAATGAAAGAATTTTCTGTAGATCTGGGATCTGGACACCTGCCCCCCGATTTGATTG |
| ASCRP3004080 | 0.436284421 | 0.91119147 | 1.0250597 | down | hsa_circRNA_022430 | hsa_circ_0022430 | circBase | chr11 | - | 62303416 | 62303570 | exonic | NM_001620 | AHNAK | CAGAACTCCCCTGCGGCCCGCACTGGGGTGGTCAAGGAGGATGGAGAAGGAGGAGACAAC |
| ASCRP3004085 | 0.275266002 | 0.909237851 | 1.0293285 | down | hsa_circRNA_004695 | hsa_circ_0004695 | circBase | chr15 | + | 29346047 | 29347038 | exonic | NM_005503 | APBA2 | CAGAAGAGAGGCTGAAGTGGCCCCACGAGCAGTGGCTGCCTCCGGGTGATGATGGCTGTG |
| ASCRP3004087 | 0.628447964 | 0.929544944 | 1.0324676 | down | hsa_circRNA_406841 | | 25070500 | chr6 | + | 126434011 | 126496057 | antisense | NR_049880 | MIR5695 | CAGAAGAGTGAGTCGCAAAAAGAGAAAATGACAGAAATGGAGCACCCTGGGAAAAGAAAC |
| ASCRP3004089 | 0.748475331 | 0.95565436 | 1.0138301 | down | hsa_circRNA_405629 | | 25070500 | chr17 | - | 73826108 | 73827429 | exonic | NM_199242 | UNC13D | CAGAAGCACAAGAAGGACCTTCACCCATTGTTTGATGAGACCTTTGAATTCCTCCTGACC |
| ASCRP3004091 | 0.699944942 | 0.944102043 | 1.0106736 | down | hsa_circRNA_102468 | hsa_circ_0002259 | circBase | chr19 | + | 16192722 | 16192856 | exonic | NM_003290 | TPM4 | CAGAAGCTGGAGGAGGCAGAAAAAGCTGCAGATGAGAGTGAGAGGCTGAAGGTGATGTGG |
| ASCRP3004095 | 0.838430511 | 0.978181218 | 1.0043386 | down | hsa_circRNA_406186 | | 25070500 | chr22 | + | 32206506 | 32215211 | exonic | NM_014662 | DEPDC5 | CAGAAGGCGCTGGATGCACACTTTTCCTGTGGCTCTCGGGAGTCCAAAAGAATCTGAGAA |
| ASCRP3004102 | 0.866853118 | 0.979863126 | 1.005748 | down | hsa_circRNA_074047 | hsa_circ_0074047 | circBase | chr5 | + | 135468535 | 135513085 | exonic | uc003lbj.1 | SMAD5 | CAGAATATCACCGGCAGGATGTAACCAGCACCCCATGTTGGATTGAGATTCCGGGTCCTG |
| ASCRP3004108 | 0.23000698 | 0.909237851 | 1.0956999 | down | hsa_circRNA_103669 | hsa_circ_0070049 | circBase | chr4 | + | 77659913 | 77663079 | exonic | NM_020859 | SHROOM3 | CAGACAAGCGCCAGCTCCTCTACTAGTGACCTCTCCAACTATGACCATGCTTATCTAAGG |
| ASCRP3004112 | 0.120149283 | 0.909237851 | 1.1565299 | down | hsa_circRNA_102593 | hsa_circ_0052011 | circBase | chr19 | + | 50902104 | 50902741 | exonic | NM_001256849 | POLD1 | CAGACAGAGCCCCTCATCTTCCAACAGTTGGAGATTGACCATTATGTGGCAGGATGGATG |
| ASCRP3004114 | 0.560750443 | 0.920978531 | 1.0700683 | down | hsa_circRNA_406445 | | 25070500 | chr4 | + | 5754784 | 5755476 | intronic | ENST00000264956 | EVC | CAGACAGGAGAAAGCATGAGGGTCCCCACTGAAATTCTGGCTCACAGACCGTGGGCATGG |
| ASCRP3004120 | 0.317006622 | 0.909237851 | 1.0632076 | down | hsa_circRNA_406755 | | 25070500 | chr6 | + | 33365805 | 33366164 | exonic | NM_002263 | KIFC1 | CAGACCACAGAGGTCCCCCCTATTGGAAGTAAAGGGGAACATAGAACTGAAGAGACCTCT |
| ASCRP3004123 | 0.945407862 | 0.990803144 | 1.0069586 | down | hsa_circRNA_090920 | hsa_circ_0090920 | circBase | chrX | - | 65824262 | 65825068 | exonic | NM_021783 | EDA2R | CAGACCCCCACCTCTGAGGTTCAATGATTGTGGTTATGGAGAGGGTGGAGATGCCTACTG |
| ASCRP3004125 | 0.336952774 | 0.91119147 | 1.0240114 | down | hsa_circRNA_405715 | | 25070500 | chr19 | + | 3530860 | 3531684 | intronic | ENST00000313639 | FZR1 | CAGACCTGACACCGTGCAGAGGGCTTGGCCCCCACCTGGGAGATCTGATGGGGCTCTTGA |
| ASCRP3004134 | 0.558576083 | 0.920978531 | 1.0206534 | down | hsa_circRNA_405651 | | 25070500 | chr18 | + | 8783527 | 8784841 | exonic | NM_015210 | MTCL1 | CAGACTTGCAGGATGACAGTGCCGATTTGAGGTGCCAGCTCCAGTTTGCCAAAGAGGAAG |
| ASCRP3004135 | 0.247527841 | 0.909237851 | 1.1039341 | down | hsa_circRNA_104889 | hsa_circ_0005456 | circBase | chr9 | + | 118949432 | 118950495 | exonic | NM_002581 | PAPPA | CAGACTTGCTTTGACCCCGACTCTCCACACAGGGCTGTATGACAAATGTTCTTATATCTC |
| ASCRP3004146 | 0.29790839 | 0.909237851 | 1.0382309 | down | hsa_circRNA_406875 | | 25070500 | chr6 | - | 150157220 | 150164260 | exonic | NM_032832 | LRP11 | CAGAGAGTCAAATCATTCCTGTGATGCCAGGTGCCTCAATCAGGAACCCTGAAGCTGTCC |
| ASCRP3004152 | 0.204036882 | 0.909237851 | 1.0440981 | down | hsa_circRNA_104977 | hsa_circ_0089852 | circBase | chrX | + | 9469819 | 9469889 | exonic | NM_005647 | TBL1X | CAGAGCACCAGGCCCACCGTGAAAGATTTCCGTGAGGGTGGAAGAAAATCATACCATATC |
| ASCRP3004153 | 0.85660695 | 0.978983332 | 1.0107199 | down | hsa_circRNA_400947 | | 25242744 | chr12 | - | 48191166 | 48192435 | exonic | NM_015401 | HDAC7 | CAGAGCACTTCCCTCTGCGCAAGACAGCTCTCCATGGACACGCCGATGCCCGAGTTGCAG |
| ASCRP3004157 | 0.351840787 | 0.91119147 | 1.0364182 | down | hsa_circRNA_101015 | hsa_circ_0000378 | circBase | chr12 | - | 12397195 | 12397589 | exonic | NM_002336 | LRP6 | CAGAGCTATTGCCTTAGATCCTTCAAGTGGCGGCCCCTTTGTTGCTTTATGCAAACAGAC |
| ASCRP3004158 | 0.679524336 | 0.941064238 | 1.0274038 | down | hsa_circRNA_401904 | | 25242744 | chr17 | - | 80992910 | 81006661 | exonic | NM_001009905 | B3GNTL1 | CAGAGCTCAGGGTCTTACCTTTGCTTTTTGGATTCGTCTATTATCCTCCCAGTCCACAAC |
| ASCRP3004159 | 0.69481135 | 0.943480789 | 1.0141828 | down | hsa_circRNA_104428 | hsa_circ_0081258 | circBase | chr7 | + | 98513345 | 98515302 | exonic | NM_003496 | TRRAP | CAGAGCTCATGCAGCCTCACTTGCACAAGATTGTGAACAGCTCTATGGAGCTCGCGCAGA |
| ASCRP3004165 | 0.754762149 | 0.957711417 | 1.017291 | down | hsa_circRNA_045502 | hsa_circ_0045502 | circBase | chr17 | - | 66426136 | 66430767 | exonic | NM_017983 | WIPI1 | CAGAGGAACATCTGTACCCTCTCAACGGCACAGTCATCCGGGTGTTCTCTGTCCCTGATG |
| ASCRP3004168 | 0.246908557 | 0.909237851 | 1.0191841 | down | hsa_circRNA_401774 | | 25242744 | chr17 | + | 30509775 | 30510291 | exonic | NM_018307 | RHOT1 | CAGAGGAGAAGGAGGCTGCCTTTAATATTGGTTGGGAACAAATCTGATCTGGTGGAATAT |
| ASCRP3004173 | 0.66128274 | 0.936062326 | 1.0611207 | down | hsa_circRNA_104469 | hsa_circ_0082140 | circBase | chr7 | + | 127447537 | 127528065 | exonic | NM_014390 | SND1 | CAGAGGCCAGGATAAGAACAAGAAACTGCGTCCCCTGTATGACATTCCTTACATGTTTGA |
| ASCRP3004174 | 0.449757068 | 0.91119147 | 1.02403 | down | hsa_circRNA_100857 | hsa_circ_0023056 | circBase | chr11 | + | 66974980 | 66975159 | exonic | NM_012308 | KDM2A | CAGAGGCCCTCCACGGGAGTCGTCGCATGGTGGATGTCATGGACGTGAACACACAGAAAG |
| ASCRP3004176 | 0.905581537 | 0.982324123 | 1.0047935 | down | hsa_circRNA_101832 | hsa_circ_0039857 | circBase | chr16 | - | 67761229 | 67771938 | exonic | NM_020850 | RANBP10 | CAGAGGGAACCTGTGTGTGCTGCCCTCAACAGCGCCATTTTAGGTATAGCCTTCACAGAC |
| ASCRP3004179 | 0.561070405 | 0.920978531 | 1.0344212 | down | hsa_circRNA_103390 | hsa_circ_0066147 | circBase | chr3 | - | 52960046 | 52962357 | exonic | NM_016329 | SFMBT1 | CAGAGGTGCTTCCCTCCGGTTTTTGATGAGAAGTACTTTCTGGTGGAAATGGATGACTTG |
| ASCRP3004185 | 0.502667319 | 0.914489113 | 1.0293166 | down | hsa_circRNA_064090 | hsa_circ_0064090 | circBase | chr3 | + | 5249777 | 5249948 | exonic | NM_014674 | EDEM1 | CAGAGTTAGTGGAATCCACATATCTCCTCTACCAGGTGCTGATAGGAGATGTGGAAGATG |
| ASCRP3004186 | 0.018414689 | 0.909237851 | 1.1292761 | down | hsa_circRNA_092390 | hsa_circ_0001968 | circBase | chr11 | + | 68359043 | 68367962 | exonic | NM_018312 | PPP6R3 | CAGAGTTCACGTCTTCCCTGAGGGAAATATTGCCTTGTTTGAAGCATGTTGTAAGGAAAG |
| ASCRP3004196 | 0.56580233 | 0.920978531 | 1.0198033 | down | hsa_circRNA_008229 | hsa_circ_0008229 | circBase | chr12 | + | 53670403 | 53671744 | exonic | NM_012291 | ESPL1 | CAGATCCTAGCAGCCCTCTACCAGCTGGTGGCAAAGGACTCTGCGAGACAGCCTCAGTGG |
| ASCRP3004197 | 0.631474686 | 0.929544944 | 1.0436961 | down | hsa_circRNA_401955 | | 25242744 | chr18 | + | 32386182 | 32392077 | exonic | NM_001390 | DTNA | CAGATCCTGTTTCTCCCAACAGGGAAGGCCATGGTAAAATTTCAGTATTTGCTGTCAAAA |
| ASCRP3004203 | 0.523969711 | 0.916971445 | 1.0318091 | down | hsa_circRNA_036315 | hsa_circ_0036315 | circBase | chr15 | - | 75197494 | 75197572 | exonic | NM_020447 | FAM219B | CAGATGAAAACCTGGTGTCCCTCGACTCTGACAGGTCAGTGAAGTTTAACAAGGGCTATA |
| ASCRP3004215 | 0.025525885 | 0.909237851 | 1.0423109 | down | hsa_circRNA_103748 | hsa_circ_0071124 | circBase | chr4 | + | 148968042 | 148968202 | exonic | NM_024605 | ARHGAP10 | CAGATGGAGGGAGCTTTGGAGACTGGGCATCCACTATGTGACAATCCTTACCCTTCCAAG |
| ASCRP3004231 | 0.630085369 | 0.929544944 | 1.0477576 | down | hsa_circRNA_402965 | | 25242744 | chr3 | + | 136035782 | 136048826 | exonic | uc003eqz.1 | PCCB | CAGCAAGAAGGTTGTTGATGAGCGTGAATTTTTTGAGATCATGCCCAATTATGCCAAGAA |
| ASCRP3004232 | 0.564830986 | 0.920978531 | 1.0385974 | down | hsa_circRNA_051427 | hsa_circ_0051427 | circBase | chr19 | + | 45515193 | 45515534 | exonic | uc021uvp.1 | RELB | CAGCAAGACGCTGCCCGCCATCGAGAGATCATCGACGAGTACATCAAGGAGAACGGCTTC |
| ASCRP3004240 | 0.475717407 | 0.91119147 | 1.0244066 | down | hsa_circRNA_089558 | hsa_circ_0089558 | circBase | chr9 | + | 139563008 | 139563125 | exonic | NM_016215 | EGFL7 | CAGCACCTACCGCCGTAGGGTGTGTGCTGTCCGGGCTCACGGGGACCCTGTCTCCGAGTC |
| ASCRP3004242 | 0.665380653 | 0.937594692 | 1.0444089 | down | hsa_circRNA_008059 | hsa_circ_0008059 | circBase | chr2 | + | 85595808 | 85596953 | exonic | NM_032213 | ELMOD3 | CAGCACTTCCAGACTGTGGACCTTTCCCCCTTCAAGTTGTGAGTACAGAGGTGGTCAGAG |
| ASCRP3004251 | 0.028201241 | 0.909237851 | 1.0686566 | down | hsa_circRNA_404054 | | 25242744 | chr8 | + | 69039612 | 69104760 | exonic | NM_024870 | PREX2 | CAGCAGCGGTGTGCATCGAGGTAAAGTGTAGGCTACTCCTGGCTCTTCTTGAATATTCAG |
| ASCRP3004254 | 0.37996039 | 0.91119147 | 1.0442383 | down | hsa_circRNA_004895 | hsa_circ_0004895 | circBase | chr1 | - | 33160465 | 33161645 | exonic | NM_030786 | SYNC | CAGCAGTACAGGGAAAACAAGAGTAGAGGCCAATTCTCCTCTTCCAAAGAACTCTGGATC |
| ASCRP3004256 | 0.394672056 | 0.91119147 | 1.0345985 | down | hsa_circRNA_008166 | hsa_circ_0008166 | circBase | chr12 | + | 104376576 | 104379506 | exonic | NM_003211 | TDG | CAGCAGTGAACCTTGTGGCTTCTCTTCAAATGGGCTAAGGAAGTGTTTGTTTATGTCAGG |
| ASCRP3004266 | 0.232503989 | 0.909237851 | 1.0380248 | down | hsa_circRNA_092587 | hsa_circ_0001899 | circBase | chr9 | - | 137293508 | 137293613 | antisense | NM_002957 | RXRA | CAGCCATGGAGCCTCGCCCCGTCGGGGAGCTCAGGGTGCTGATGGGAGAATGCAGCTGTC |
| ASCRP3004267 | 0.350950549 | 0.91119147 | 1.0449214 | down | hsa_circRNA_404588 | | 25070500 | chr1 | - | 154061896 | 154091284 | exonic | NM_207308 | NUP210L | CAGCCATGTCCTCTGCCACAGCACAACAAATGGCTGAATAAAGATATTCAGCCTATAAAA |
| ASCRP3004270 | 0.97803866 | 0.997082313 | 1.0012605 | down | hsa_circRNA_405587 | | 25070500 | chr17 | - | 41886323 | 41888573 | exonic | NM_001932 | MPP3 | CAGCCCCATTTATACCACCAGGCCCCGAAAGAGCCATGAGAAGGAAGGAGTGGAATATCA |
| ASCRP3004272 | 0.724892931 | 0.95002228 | 1.014123 | down | hsa_circRNA_101509 | hsa_circ_0035072 | circBase | chr15 | - | 44859621 | 44861703 | exonic | NM_025137 | SPG11 | CAGCCCTGGGGTACGGCTCCTCACTGGCATTGGAAGGTACAACGAGATGACATACATATT |
| ASCRP3004283 | 0.192932349 | 0.909237851 | 1.0866298 | down | hsa_circRNA_006668 | hsa_circ_0006668 | circBase | chr12 | + | 51442816 | 51451911 | exonic | NM_015416 | LETMD1 | CAGCCTGAAAGGTCTTCAAAGCTTCACCTTTCTCCAAAGGCAGATGTGAAGAACTTGATG |
| ASCRP3004287 | 0.31126874 | 0.909237851 | 1.0574989 | down | hsa_circRNA_000960 | hsa_circ_0000960 | circBase | chr19 | + | 55758339 | 55758435 | antisense | NM_014931 | PPP6R1 | CAGCGTGTCCAGCAGCTTGCGGTTGACGACCTTGCACTCCTGCAGCACGTCTTCCTCGTC |
| ASCRP3004302 | 0.101710624 | 0.909237851 | 1.1236858 | down | hsa_circRNA_001302 | hsa_circ_0001416 | circBase | chr4 | + | 57976053 | 57976143 | sense overlapping | NR_034081 | IGFBP7-AS1 | CAGCTGGCAGCCGCTCGGGTAGGTGGTGCCCTTGCTGACCTGGGTGATGGCCTTCTCCCC |
| ASCRP3004305 | 0.907248235 | 0.982324123 | 1.0041543 | down | hsa_circRNA_003022 | hsa_circ_0003022 | circBase | chr10 | - | 3205916 | 3209234 | exonic | NM_014889 | PITRM1 | CAGCTTAAGCAGTTTCATGCCACTCACTATCACCCAAGCAATGCTAGGTGACATCTGTTC |
| ASCRP3004306 | 0.124885961 | 0.909237851 | 1.0674299 | down | hsa_circRNA_100861 | hsa_circ_0023180 | circBase | chr11 | + | 68115314 | 68125315 | exonic | NM_002335 | LRP5 | CAGCTTCATCCACCGTGCCAACCTGGACGGCTCGTTCCGCCTCGCCGCTCCTGCTATTTG |
| ASCRP3004307 | 0.382335882 | 0.91119147 | 1.035558 | down | hsa_circRNA_100803 | hsa_circ_0000296 | circBase | chr11 | - | 46098304 | 46113774 | exonic | NM_016621 | PHF21A | CAGCTTCATGAACTCCAAGCCAAAATCACAGCTTTGAGTGAGAAACAGGGGGCTAGAGAG |
| ASCRP3004311 | 0.430243913 | 0.91119147 | 1.0377822 | down | hsa_circRNA_000297 | hsa_circ_0000297 | circBase | chr11 | + | 46700538 | 46700969 | antisense | NM_004308 | ARHGAP1 | CAGGAAAGCAGTCAGGAAACGAAGCACCTGGTAGTTCTCCTCGGGCAGCTCCAAACCCGG |
| ASCRP3004316 | 0.125623672 | 0.909237851 | 1.0363986 | down | hsa_circRNA_092568 | hsa_circ_0001689 | circBase | chr7 | - | 26232870 | 26232993 | exonic | NM_002137 | HNRNPA2B1 | CAGGAACATGGGGGGACCATATGGTGGAGGAAATTATGGAAGTGGAAATTACAATGATTT |
| ASCRP3004320 | 0.055721985 | 0.909237851 | 1.0818253 | down | hsa_circRNA_007776 | hsa_circ_0007776 | circBase | chr12 | - | 112096539 | 112098479 | exonic | NM_006768 | BRAP | CAGGAAGAGAAAATAGATGCCTTACAGTTAGAGGTGTCCTGTTTGCCGGTACTGTCAAAC |
| ASCRP3004326 | 0.152893319 | 0.909237851 | 1.1139385 | down | hsa_circRNA_400744 | | 25242744 | chr11 | + | 45203311 | 45204674 | exonic | NR_046338 | PRDM11 | CAGGACAAATCAGCTGGCTTCTTCTCCTGGCTGGACAGAATGACCGAGAACATGAAGGAG |
| ASCRP3004331 | 0.287912894 | 0.909237851 | 1.0860374 | down | hsa_circRNA_101902 | hsa_circ_0003098 | circBase | chr16 | + | 88037900 | 88071617 | exonic | NM_001173539 | BANP | CAGGACGGTCAGCGTCGTCCCCCAGACTACAGTAATACTCAACAATGATCGGCAGAACGC |
| ASCRP3004335 | 0.77681164 | 0.965028024 | 1.0067449 | down | hsa_circRNA_006497 | hsa_circ_0006497 | circBase | chr3 | - | 119720892 | 119721086 | exonic | NM_002093 | GSK3B | CAGGAGAACTGGTCGCCATCAAGAAAGTATTGCAGGACAAGAGATTTAAGGAGACAAGGA |
| ASCRP3004337 | 0.030477682 | 0.909237851 | 1.0705223 | down | hsa_circRNA_005824 | hsa_circ_0005824 | circBase | chr12 | - | 27143382 | 27156323 | exonic | NM_016551 | TM7SF3 | CAGGAGAGGGTAGTTGTGCTTCCCTAGGTCTTATTGAATTTTCTGTGGGGAAATTTAGAT |
| ASCRP3004341 | 0.483267844 | 0.91119147 | 1.0518316 | down | hsa_circRNA_405393 | | 25070500 | chr15 | - | 75892977 | 75893559 | sense overlapping | NM_005701 | SNUPN | CAGGAGCATGAGTCAGTTTAAATTTGTGGGGCTAAAGAACTTCCCTTGCACTCCCGAAAG |
| ASCRP3004344 | 0.881447522 | 0.979863126 | 1.0074342 | down | hsa_circRNA_064810 | hsa_circ_0064810 | circBase | chr3 | + | 36524483 | 36534721 | exonic | NM_003149 | STAC | CAGGAGGCGGGTATGACCTAAGGAAACGCAGCAACAGCGGAACAAATGCTAAGCATGGAC |
| ASCRP3004345 | 0.83480196 | 0.978181218 | 1.0115258 | down | hsa_circRNA_405791 | | 25070500 | chr19 | - | 45885784 | 45889438 | exonic | NM_006663 | PPP1R13L | CAGGAGGGCTACGTGCCGCGGAACTACTTCGGGGAGATGCGCTCTGTGCTGCGGAAGGCG |
| ASCRP3004360 | 0.820427993 | 0.976440085 | 1.0101466 | down | hsa_circRNA_402549 | | 25242744 | chr20 | + | 31376659 | 31381401 | exonic | NM_006892 | DNMT3B | CAGGCAGTAGGAAATTAGAATCAAGGAAATACGGATGGGAAGGAGTTTGGAATAGGGGAC |
| ASCRP3004368 | 0.193551901 | 0.909237851 | 1.079412 | down | hsa_circRNA_029021 | hsa_circ_0029021 | circBase | chr12 | - | 122237752 | 122239714 | exonic | NR_002809 | LINC01089 | CAGGCCTAGTTTGACTCTGACAGTCCTCCCACTAAGAGCAGTAAACAGTCCTCAGCGAAG |
| ASCRP3004370 | 0.776666496 | 0.965028024 | 1.0197007 | down | hsa_circRNA_400068 | hsa_circ_0092314 | circBase | chr22 | + | 20113099 | 20113439 | intronic | ENST00000331821 | RANBP1 | CAGGCTGATCTCTTCCCTGCTCAGAGGTCTTTCCAGTCCAGATCGTCCATGCTGTTTGGG |
| ASCRP3004374 | 0.535421008 | 0.919549314 | 1.0347913 | down | hsa_circRNA_406223 | | 25070500 | chr3 | - | 10354443 | 10357007 | sense overlapping | NM_030673 | SEC13 | CAGGGAGCCATTTAGTTCTGTCCAAAGTAGTGCCTGCAGAGGCACTGTGGGGGGTAAGTG |
| ASCRP3004378 | 0.788691646 | 0.967199246 | 1.0074829 | down | hsa_circRNA_060633 | hsa_circ_0060633 | circBase | chr20 | + | 46267751 | 46276110 | exonic | NM_006534 | NCOA3 | CAGGGCCAGCAGGTTTGAAAAGTTCACAGTCTGTGCAGTCTATTCGTCCTCCATATAACC |
| ASCRP3004381 | 0.870256592 | 0.979863126 | 1.0154479 | down | hsa_circRNA_404617 | | 25070500 | chr1 | + | 179054762 | 179057224 | exonic | NM_022371 | TOR3A | CAGGGCTGAGTCTCCATGGACTATCTTTCTGTTTCTCAGGCTTAGAGTGGGACCTGAATG |
| ASCRP3004385 | 0.617623641 | 0.929544944 | 1.0139831 | down | hsa_circRNA_002630 | hsa_circ_0002630 | circBase | chr4 | + | 2883624 | 2896458 | exonic | NM_001119 | ADD1 | CAGGGGCTGCGGCTTTCTGTGAAGAATTGGAATCAATGATACAGGAGCAATTTAAGAAGG |
| ASCRP3004386 | 0.094067472 | 0.909237851 | 1.0579766 | down | hsa_circRNA_405955 | | 25070500 | chr2 | + | 122797769 | 122798857 | intergenic |  |  | CAGGGGTGATGGAGTGCAGGAATCAGGAAAATATAAACTGCTTGAAGTACCAGTCTTCTT |
| ASCRP3004387 | 0.656339848 | 0.933363463 | 1.0102527 | down | hsa_circRNA_103922 | hsa_circ_0073568 | circBase | chr5 | + | 110782383 | 110784901 | exonic | NM_001744 | CAMK4 | CAGGGTACTGCGTATCTACATGAAAATGGGATTGTCCATCGTGATCTCAAACCAGAGAAT |
| ASCRP3004395 | 0.453248497 | 0.91119147 | 1.0310081 | down | hsa_circRNA_104860 | hsa_circ_0003965 | circBase | chr9 | - | 111812562 | 111826847 | exonic | NM_032012 | TMEM245 | CAGGTCCTTCTTCTAATATTATTGGCCAGTCTGTGGAAGAAGCTATCAGTTGGCTTCCTG |
| ASCRP3004400 | 0.385477687 | 0.91119147 | 1.0241916 | down | hsa_circRNA_103258 | hsa_circ_0063822 | circBase | chr22 | - | 46829289 | 46832186 | exonic | NM_014246 | CELSR1 | CAGGTGCAGTACTACAACAAGGTTTGCCACTCAGGAAAGGAACGGCTTGCTTCTCTACAA |
| ASCRP3004404 | 0.178468539 | 0.909237851 | 1.045205 | down | hsa_circRNA_101305 | hsa_circ_0002807 | circBase | chr13 | + | 114149816 | 114164739 | exonic | NM_017905 | TMCO3 | CAGGTTCCTCATGGGCAGTGCTCGGGGTGACAAAGAAGCTGAAAATGTGTGTCTGACATG |
| ASCRP3004405 | 0.19366264 | 0.909237851 | 1.0900758 | down | hsa_circRNA_404497 | | 25070500 | chr1 | + | 45241812 | 45242313 | intronic | ENST00000372209 | RPS8 | CAGGTTCCTCTCCCTCACTTGCCTTGCTCTCCTTGGTGGGTGCGAGCGTGGGCCTGTCCG |
| ASCRP3004407 | 0.888418188 | 0.979863126 | 1.0082583 | down | hsa_circRNA_405011 | | 25070500 | chr12 | + | 56334222 | 56334387 | intronic | ENST00000331886 | DGKA | CAGGTTGAGAGGAGACAGGGTTACCTTCGTGATCTCTCTTTTGGGAGCCATCCCTTCTGG |
| ASCRP3004410 | 0.534652869 | 0.919549314 | 1.0462334 | down | hsa_circRNA_103562 | hsa_circ_0007345 | circBase | chr3 | - | 196812450 | 196846401 | exonic | NM_004087 | DLG1 | CAGTAAACGCAGCTTCTTCTCAGCCTGTTGATAACCATGTTAGCCCATCTTCCTTCTTGG |
| ASCRP3004411 | 0.337274823 | 0.91119147 | 1.0965259 | down | hsa_circRNA_406551 | | 25070500 | chr4 | - | 152069073 | 152080504 | exonic | NM_001009555 | SH3D19 | CAGTAAATACATGGTTTACATGAGGATCCACAAAGTCCACCTCCTCTCCCTGCTGAAAAA |
| ASCRP3004429 | 0.489915318 | 0.91119147 | 1.0235326 | down | hsa_circRNA_008264 | hsa_circ_0008264 | circBase | chr1 | + | 201786215 | 201786413 | exonic | NM_020443 | NAV1 | CAGTCAAAGACAGCACCCCAAGTTCTCTGGACTCAGATCCTCTGGTCCATGGACAGAAAG |
| ASCRP3004431 | 0.422160374 | 0.91119147 | 1.0235404 | down | hsa_circRNA_101920 | hsa_circ_0007027 | circBase | chr16 | + | 89961445 | 89970613 | exonic | NM_014972 | TCF25 | CAGTCACCGCTTCTTTGGACCCAATGCTGAAATAAGGTTCTGCTCCAGACGAGCCCTTAC |
| ASCRP3004432 | 0.244377812 | 0.909237851 | 1.0653408 | down | hsa_circRNA_103529 | hsa_circ_0009131 | circBase | chr3 | + | 183454505 | 183480067 | exonic | NM_018023 | YEATS2 | CAGTCAGCAAAGGTTCAGCCCTCCAAGAGAGCCTCCTTTTCACCTGACCAGAAGAGGCTG |
| ASCRP3004445 | 0.921036462 | 0.98346125 | 1.0041372 | down | hsa_circRNA_102148 | hsa_circ_0045006 | circBase | chr17 | + | 59152280 | 59161925 | exonic | NM_017679 | BCAS3 | CAGTCTCTGATCGAAGGGGAGTTTCCACAGTGATTGATGCTGCCTCAGTGGTTCCCCCTG |
| ASCRP3004449 | 0.287261318 | 0.909237851 | 1.0532895 | down | hsa_circRNA_403202 | | 25242744 | chr4 | - | 111026652 | 111119523 | exonic | NM_001130721 | ELOVL6 | CAGTCTTCAGAGAACACGTAGCGACTCCGAAGATCAGCCCCAATGAACATGTCAGTGTTG |
| ASCRP3004463 | 0.249017754 | 0.909237851 | 1.0245674 | down | hsa_circRNA_104097 | hsa_circ_0076070 | circBase | chr6 | + | 34835001 | 34835393 | exonic | NM_017754 | UHRF1BP1 | CAGTGCCCAGAGTCTGGTCCAGAATCTGTTCCACCAGGATCTCTTTCAAATGTCTCAGAT |
| ASCRP3004466 | 0.230978599 | 0.909237851 | 1.0332542 | down | hsa_circRNA_101781 | hsa_circ_0006012 | circBase | chr16 | - | 28123137 | 28137169 | exonic | NM_015171 | XPO6 | CAGTGCTGTTGCCCCACAGAGAAAGATGCCACTGGATGACAGACACAGGTTGAACATCAC |
| ASCRP3004469 | 0.772287158 | 0.964012 | 1.0083648 | down | hsa_circRNA_003188 | hsa_circ_0003188 | circBase | chr12 | + | 78400198 | 78452895 | exonic | NM_014903 | NAV3 | CAGTGGATGCAGACAGATTCCTCCAAAGGACCTCAATCGTCTTCAGGTGTAAATGGTAAC |
| ASCRP3004471 | 0.038897547 | 0.909237851 | 1.0730221 | down | hsa_circRNA_100440 | hsa_circ_0006123 | circBase | chr1 | - | 211092981 | 211093411 | exonic | NM_002238 | KCNH1 | CAGTGGCCATCATGATGATTGGCTGGCATCAGCAGCCTGTTCAGCTCTCTAAAAGTTGTC |
| ASCRP3004473 | 0.812515344 | 0.97543349 | 1.0134855 | down | hsa_circRNA_104540 | hsa_circ_0005939 | circBase | chr7 | - | 156619298 | 156629579 | exonic | NM_022458 | LMBR1 | CAGTGGCTAAATGGCTCCCTGATTCATGATATGTTTCCTTCTTTTTGCCATTCTCTACGT |
| ASCRP3004476 | 0.235743482 | 0.909237851 | 1.0330881 | down | hsa_circRNA_103966 | hsa_circ_0006716 | circBase | chr5 | + | 138994170 | 138994551 | exonic | NM_003339 | UBE2D2 | CAGTGGTCTCCAGCACTAACTATTTCAAAAGTGTTCCATTGGCAAGCTACAATAATGGGG |
| ASCRP3004487 | 0.836622195 | 0.978181218 | 1.004534 | down | hsa_circRNA_100490 | hsa_circ_0006415 | circBase | chr1 | - | 233344263 | 233372726 | exonic | NM_014801 | PCNXL2 | CAGTGTTTCTACAATCAGCTAGGGACTACTTAATAGAGAGATGCAGGTCAGCTCCTCCAG |
| ASCRP3004494 | 0.266199498 | 0.909237851 | 1.0703758 | down | hsa_circRNA_405621 | | 25070500 | chr17 | + | 66337415 | 66352945 | sense overlapping | NM_014960 | ARSG | CAGTTCATCCAGCGTGCAAGGGTCTGCTTGTTTTCCCCTTGTGGTTTAATTCTACTTTTT |
| ASCRP3004495 | 0.69188071 | 0.943480789 | 1.022586 | down | hsa_circRNA_102195 | hsa_circ_0045714 | circBase | chr17 | + | 73808192 | 73809959 | exonic | NM_001080419 | UNK | CAGTTCCACCCCGAGGTGCCCATTCCTGCACAGAACCACAGGGGACACTGAGCGCAGGTA |
| ASCRP3004501 | 0.158964472 | 0.909237851 | 1.0855285 | down | hsa_circRNA_101743 | hsa_circ_0006797 | circBase | chr16 | + | 19627435 | 19659204 | exonic | NM_020314 | C16orf62 | CAGTTGATTCATGTGGGAATGGAAGTGGCCCCACATCTCAAAGAAACCCTAAATAAGAAC |
| ASCRP3004504 | 0.785435968 | 0.966801146 | 1.011475 | down | hsa_circRNA_102326 | hsa_circ_0047232 | circBase | chr18 | + | 21461899 | 21470081 | exonic | NM_000227 | LAMA3 | CAGTTGTAACAGCAATGGCCAGCTGGGCAGCTGTCATCCCCTGACTGGAGAACTGTCAGC |
| ASCRP3004514 | 0.129012479 | 0.909237851 | 1.1126173 | down | hsa_circRNA_084725 | hsa_circ_0084725 | circBase | chr8 | - | 70588843 | 70594578 | exonic | NM_030958 | SLCO5A1 | CAGTTTGTTTTGTTGCGAACACTTGACCTTCTCTCACCATGCCCCATAGGAATCTGACAG |
| ASCRP3004515 | 0.485376328 | 0.91119147 | 1.0534201 | down | hsa_circRNA_001096 | hsa_circ_0000866 | circBase | chr19 | - | 1439001 | 1439241 | antisense | NM_001018 | RPS15 | CAGTTTTCTGGGCGCAAGACCACACTCAGTTCCAAACCCGCGCGGAGACCATGCCCACCA |
| ASCRP3004516 | 0.440615434 | 0.91119147 | 1.0245504 | down | hsa_circRNA_103985 | hsa_circ_0007551 | circBase | chr5 | + | 149921131 | 149922533 | exonic | NM_001543 | NDST1 | CAGTTTTTTAATGGCCACAACTATCACAAAGGCATCGACTGGACCCCTGCGAGGACAAAC |
| ASCRP3004519 | 0.414293978 | 0.91119147 | 1.1102504 | down | hsa_circRNA_062898 | hsa_circ_0062898 | circBase | chr22 | + | 31806997 | 31830172 | exonic | NM_004147 | DRG1 | CATAAAAGATCTGGTAGGCTGGTCAGCTACATGCACTCCTGGATCTCCCAGGTATCATTG |
| ASCRP3004520 | 0.533167487 | 0.919549314 | 1.0822432 | down | hsa_circRNA_023525 | hsa_circ_0023525 | circBase | chr11 | - | 73685715 | 73687787 | exonic | NM_003355 | UCP2 | CATAAAGCAAGCTCAACCTTGGGGACCTCTCCCAATGTTGCTCGTAATGCCATTGTCAAC |
| ASCRP3004528 | 0.052941392 | 0.909237851 | 1.0807185 | down | hsa_circRNA_104058 | hsa_circ_0075629 | circBase | chr6 | + | 10961537 | 10964076 | exonic | NM_001040274 | SYCP2L | CATAAGAAAGCCAAAGCCTGGTTTTATGATTGCAGCCCTCAGACTCAATAAACTAGAGCG |
| ASCRP3004541 | 0.208260847 | 0.909237851 | 1.1115661 | down | hsa_circRNA_104771 | hsa_circ_0002702 | circBase | chr9 | + | 35546426 | 35548532 | exonic | NM_014806 | RUSC2 | CATACCCAGAGGGATGCAAGAGCTAGAGCTGACGGTGCCAAGCTCTCCTGATGAAATGTG |
| ASCRP3004547 | 0.937254919 | 0.988003486 | 1.0029897 | down | hsa_circRNA_104632 | hsa_circ_0084595 | circBase | chr8 | + | 62366699 | 62371101 | exonic | NM_173519 | CLVS1 | CATACGTCCTCGAATCTGGAGAGAGAATGCTCACCCAAGCTGATGAAAAGGACAGCTTTC |
| ASCRP3004550 | 0.494390841 | 0.91167947 | 1.052647 | down | hsa_circRNA_406073 | | 25070500 | chr2 | + | 242396161 | 242415402 | exonic | NM_014808 | FARP2 | CATAGAGAACCTCATTGCTCCTGGCAGGGCCTTTCCACGAAGAGTCCTCAGCCTTCTCCC |
| ASCRP3004553 | 0.673627146 | 0.938080316 | 1.015609 | down | hsa_circRNA_004440 | hsa_circ_0004440 | circBase | chr19 | + | 45781180 | 45783992 | exonic | NM_031417 | MARK4 | CATAGCGATTTCTGAGCTGCGGGAGCGAGTACTCAGAGGGAAGTACCGGGTCCCTTTCTA |
| ASCRP3004556 | 0.050837046 | 0.909237851 | 1.064415 | down | hsa_circRNA_000716 | hsa_circ_0000716 | circBase | chr16 | - | 75654163 | 75656362 | exonic | NM_012091 | ADAT1 | CATAGGACAGTCCAAAATGAGGAAGAACGGTCGATGGCCAGGTGGTCTTCATAAACTATA |
| ASCRP3004560 | 0.88020605 | 0.979863126 | 1.0045898 | down | hsa_circRNA_102943 | hsa_circ_0006879 | circBase | chr2 | + | 232099936 | 232104754 | exonic | NM_025139 | ARMC9 | CATAGTGTGGACTTCACGAGGCCTGGGACGAAATCTTGCAGCAGCTCCACCAGCAGCTGG |
| ASCRP3004572 | 0.271840283 | 0.909237851 | 1.0404994 | down | hsa_circRNA_064679 | hsa_circ_0064679 | circBase | chr3 | + | 32483331 | 32493965 | exonic | NM_138410 | CMTM7 | CATATGGCTGTCCTATAAGATCTCGTGTGTAACCCAGTCCACAGGTCACCCTGCTGATTG |
| ASCRP3004573 | 0.755591693 | 0.957859239 | 1.0177649 | down | hsa_circRNA_102751 | hsa_circ_0055033 | circBase | chr2 | + | 69304539 | 69318051 | exonic | NM_018153 | ANTXR1 | CATATGTGCAGGAGCTGGCCCGGATTGCGGACAGTAAGGATCATGTGTTTCCCGTGAATG |
| ASCRP3004579 | 0.942226013 | 0.990259757 | 1.004771 | down | hsa_circRNA_405467 | | 25070500 | chr16 | - | 56518672 | 56536368 | sense overlapping | NM_031885 | BBS2 | CATCAACACACTGTTCAAAATCATGCGAGTGGGGACAGCTTCTTCCTAGTCCGGGGCTAC |
| ASCRP3004581 | 0.503442577 | 0.914489113 | 1.0446991 | down | hsa_circRNA_102377 | hsa_circ_0047818 | circBase | chr18 | + | 56601664 | 56606853 | exonic | NM_018181 | ZNF532 | CATCAAGATAGGAGAACCAAAAAGACTTGCACTATCTGCCAGATGCTGCTTCCTAACCAG |
| ASCRP3004582 | 0.035977901 | 0.909237851 | 1.1291809 | down | hsa_circRNA_103987 | hsa_circ_0004104 | circBase | chr5 | - | 151043647 | 151049345 | exonic | NM_003118 | SPARC | CATCAAGCAGAGTGTGCAGCAATGACAACAAGACCTTCGACTCTTCCTGCCACTTCTTTG |
| ASCRP3004584 | 0.560075124 | 0.920978531 | 1.0278894 | down | hsa_circRNA_020924 | hsa_circ_0020924 | circBase | chr11 | - | 3848207 | 3862213 | exonic | NM_001665 | RHOG | CATCAATAAAACCTCCTGTCTCCAGTGCACTTCCTCCGGCCTCCCGCTCTCACTTCCTTC |
| ASCRP3004585 | 0.181108346 | 0.909237851 | 1.123208 | down | hsa_circRNA_020923 | hsa_circ_0020923 | circBase | chr11 | - | 3848207 | 3849436 | exonic | NM_001665 | RHOG | CATCAATAAAACCTCCTGTCTCCAGTGGTCACTGCAGCCAGAGGGGTCCAGAAGAGAGAG |
| ASCRP3004588 | 0.680430861 | 0.941064238 | 1.016687 | down | hsa_circRNA_006555 | hsa_circ_0006555 | circBase | chr10 | - | 103344358 | 103345913 | exonic | NM_013274 | POLL | CATCAATGCCCTCAAGAGCTTCCATAAGCCTGTCACCTCGTACCAGAGTGGCTGAGCTCC |
| ASCRP3004590 | 0.518660445 | 0.916971445 | 1.0678728 | down | hsa_circRNA_028143 | hsa_circ_0028143 | circBase | chr12 | + | 110028575 | 110032986 | exonic | NM_000431 | MVK | CATCACACTCCTCAAGCCAGGTCGCCAGCTCTCCAGATCCTGCTGACCAACACCAAAGTC |
| ASCRP3004615 | 0.191511918 | 0.909237851 | 1.1043358 | down | hsa_circRNA_402026 | | 25242744 | chr19 | + | 7523397 | 7523576 | exonic | NM_015318 | ARHGEF18 | CATCCAAAGGGCTGTGGAGAGGACTCAAAGCCACCCGTCATCTCGTTACAAAAGCTCATC |
| ASCRP3004620 | 0.157017175 | 0.909237851 | 1.1348497 | down | hsa_circRNA_406718 | | 25070500 | chr5 | - | 176954915 | 176958522 | intronic | ENST00000508298 | FAM193B | CATCCACGTCTCCAACTGCTACTGGACACCTCCTTGTGGACGTCCCACAGCCACCCGCTG |
| ASCRP3004621 | 0.0062482 | 0.879037174 | 1.0564381 | down | hsa_circRNA_102206 | hsa_circ_0045863 | circBase | chr17 | + | 76082583 | 76089844 | exonic | NM_018996 | TNRC6C | CATCCAGGATGTCAACCGCTACCTCCTCAAGAGTGGAGGCATACCAACGTTTACAAATCC |
| ASCRP3004628 | 0.089661261 | 0.909237851 | 1.0706809 | down | hsa_circRNA_406343 | | 25070500 | chr3 | + | 126707363 | 126710409 | sense overlapping | NM_032242 | PLXNA1 | CATCCGCAAGGGCTGAAGCTCCTGGCACCATGATGCTCACCCCAGCAGGACCAGAGCACC |
| ASCRP3004632 | 0.87897913 | 0.979863126 | 1.0116766 | down | hsa_circRNA_406180 | | 25070500 | chr22 | + | 31008859 | 31009242 | sense overlapping | NM_000355 | TCN2 | CATCCTTGACCCAGCTTTTCCCGCGCTGCACACATACTATTGACAGGTGTCTGCCTTCAG |
| ASCRP3004637 | 0.894196465 | 0.98039597 | 1.0172848 | down | hsa_circRNA_401433 | | 25242744 | chr15 | - | 65041198 | 65043837 | exonic | NM_194272 | RBPMS2 | CATCGCACGGGACCCCTGTCCGGACACTGTTTGTCAGCGGCCTCCCTGTGGACATTAAAC |
| ASCRP3004646 | 0.168163066 | 0.909237851 | 1.0889948 | down | hsa_circRNA_100878 | hsa_circ_0023409 | circBase | chr11 | + | 71693806 | 71701763 | exonic | NM_018320 | RNF121 | CATCTACCATAGGGATGGTGACCCTCTTTCAGATGTGGGTTGTTCCCCTCTATTTCACAG |
| ASCRP3004647 | 0.161595661 | 0.909237851 | 1.0423655 | down | hsa_circRNA_102589 | hsa_circ_0051861 | circBase | chr19 | + | 49838970 | 49842193 | exonic | NM_001774 | CD37 | CATCTACCGCGAGGTCCTCGGCAGCCTGATCTTCTGCTTCGGCATCTGGATCCTCATTGA |
| ASCRP3004658 | 0.809002611 | 0.974638647 | 1.0117112 | down | hsa_circRNA_088030 | hsa_circ_0088030 | circBase | chr9 | - | 114337013 | 114348445 | exonic | NM_012212 | PTGR1 | CATCTCTACATATAACAGAACCGGCCCACTTCCCCCAGAGTTGTGGAAAGTAAAAATGTA |
| ASCRP3004663 | 0.279087915 | 0.909237851 | 1.0359932 | down | hsa_circRNA_078444 | hsa_circ_0078444 | circBase | chr6 | + | 158454486 | 158476060 | exonic | NM_003898 | SYNJ2 | CATCTGAGACTCCACAGAGGCCTGGAAGCCAATGCCCCTGCTTTCGACAGGAACCAGCTG |
| ASCRP3004667 | 0.602504415 | 0.929544944 | 1.0119891 | down | hsa_circRNA_101201 | hsa_circ_0029325 | circBase | chr12 | - | 125270902 | 125284788 | exonic | NM_005505 | SCARB1 | CATCTGCCAAATCCGGAGCCAAGTGCCCCCTTGTTTCTCTCCCATCCTCACTTCCTCAAC |
| ASCRP3004676 | 0.488148673 | 0.91119147 | 1.0299179 | down | hsa_circRNA_103290 | hsa_circ_0064324 | circBase | chr3 | - | 11643306 | 11744521 | exonic | NM_014667 | VGLL4 | CATCTTCAACCCCCATCTGCTCCAACCAGGAATGGAGACGCCATTGGATGTTTTGTCCAG |
| ASCRP3004681 | 0.922208579 | 0.9834955 | 1.002601 | down | hsa_circRNA_007755 | hsa_circ_0007755 | circBase | chr1 | - | 145592632 | 145595220 | sense overlapping | NM_006468 | POLR3C | CATCTTCCTCAGAAGATCTGGGGGGATGGAAAGCAAAATAAAGGAGGAAATTCCCAAAAC |
| ASCRP3004684 | 0.198313902 | 0.909237851 | 1.0643191 | down | hsa_circRNA_104828 | hsa_circ_0087564 | circBase | chr9 | + | 97041039 | 97055351 | exonic | NM_003448 | ZNF169 | CATCTTCTGGACCTTTGTCCAGATTTGCCTCTGCAACTTGACTCTCCTCTAGGAAGAGTA |
| ASCRP3004685 | 0.884734844 | 0.979863126 | 1.0061312 | down | hsa_circRNA_103551 | hsa_circ_0068563 | circBase | chr3 | - | 194947437 | 194947585 | exonic | NM_152531 | XXYLT1 | CATCTTCTTCCTCTCGGTCGCCATGCATCAGATCATGCCCAAAGGTCATCTTCCACGATG |
| ASCRP3004688 | 0.806599787 | 0.974037816 | 1.006035 | down | hsa_circRNA_007221 | hsa_circ_0007221 | circBase | chr10 | - | 75331178 | 75336119 | exonic | uc010qkl.1 | USP54 | CATCTTTTGCGCTCTCAAGCTCTCTGACACAGCTGGCTATTCTAGTCCCAGAAGAAAGAG |
| ASCRP3004690 | 0.129145667 | 0.909237851 | 1.0430398 | down | hsa_circRNA_104156 | hsa_circ_0001626 | circBase | chr6 | - | 90916287 | 90981660 | exonic | NM_021813 | BACH2 | CATGAACAAGCGCGTTCGTTCACATAGCTCCCAGTTTTAACATTTCGCCACCTACTGAAG |
| ASCRP3004692 | 0.237064408 | 0.909237851 | 1.1173781 | down | hsa_circRNA_005411 | hsa_circ_0005411 | circBase | chr8 | + | 28595035 | 28600731 | exonic | NM_001440 | EXTL3 | CATGAACTTCCTTGTCTCCCACATCACTCGGAAGCCCCCCATCAAGGGTGTGGAGAGAAG |
| ASCRP3004697 | 0.809279199 | 0.974638647 | 1.0107283 | down | hsa_circRNA_002983 | hsa_circ_0002983 | circBase | chr1 | + | 9786966 | 9787830 | sense overlapping | NM_005026 | PIK3CD | CATGAAGGCAAAAGCAGGACTCCCTGGCACTGGGGAAAACAGAGGAGGAGGCACTGAAGC |
| ASCRP3004699 | 0.169743723 | 0.909237851 | 1.0406147 | down | hsa_circRNA_003982 | hsa_circ_0003982 | circBase | chr17 | + | 18574179 | 18575615 | antisense | NR_026718 | FOXO3B | CATGAATCGACTATGCAGTGACAGGTTGTGCCGGATGGAGGTCTGCTTTGCCCACTTCCC |
| ASCRP3004700 | 0.684746786 | 0.942462277 | 1.0178051 | down | hsa_circRNA_102455 | hsa_circ_0049547 | circBase | chr19 | - | 12800185 | 12800444 | exonic | NM_032301 | FBXW9 | CATGACAATGGGCTCAATAGGTCCTTTGATGTGGGCCACAGCTTTCCCATCACTGGGATC |
| ASCRP3004705 | 0.172595329 | 0.909237851 | 1.0599857 | down | hsa_circRNA_102399 | hsa_circ_0004891 | circBase | chr19 | + | 1032390 | 1032695 | exonic | NM_004368 | CNN2 | CATGACGCAGGTGCAGGTGTCTCTTCTCGCCCTGGCGGGGAAGACTCATGAACAAGCTAC |
| ASCRP3004706 | 0.002438368 | 0.870215632 | 1.1067489 | down | hsa_circRNA_400982 | | 25242744 | chr12 | - | 63083478 | 63114069 | exonic | NM_020700 | PPM1H | CATGACTCCAACATCTACATTAAACCATTCCTGTCTTCAGCTCCAGAGGCATTCATGCAG |
| ASCRP3004708 | 0.111232469 | 0.909237851 | 1.0257791 | down | hsa_circRNA_001503 | hsa_circ_0001191 | circBase | chr21 | + | 38792600 | 38828610 | intronic | ENST00000462274 | DYRK1A | CATGAGAACAACACAGGAGGCTGCAGGTGGAGTGTTATAGTTTTGCCGCTGGACTCTTCC |
| ASCRP3004709 | 0.223673172 | 0.909237851 | 1.0205069 | down | hsa_circRNA_020302 | hsa_circ_0020302 | circBase | chr10 | - | 125557491 | 125558715 | exonic | NM_198148 | CPXM2 | CATGAGAATGGAGATCCTGGGCTGCCCACTGCCAGGAGTGACTGGGTGACATCCTATAAG |
| ASCRP3004719 | 0.657019108 | 0.933363463 | 1.0326669 | down | hsa_circRNA_053173 | hsa_circ_0053173 | circBase | chr2 | + | 27440257 | 27444215 | exonic | NM_004341 | CAD | CATGAGTGGCTGCAGCAGCATGGCATCCCTGGCTTGCAAGGCGCGCCCGAGGCTCCTACG |
| ASCRP3004722 | 0.887411495 | 0.979863126 | 1.0072996 | down | hsa_circRNA_100191 | hsa_circ_0012077 | circBase | chr1 | + | 44054401 | 44054671 | exonic | NM_002840 | PTPRF | CATGATCGAGGCCACAGCCCAGGTCACAGTGAAAGTGCGCCGCGTGGCTCCTCGTTTCTC |
| ASCRP3004723 | 0.397325702 | 0.91119147 | 1.0322265 | down | hsa_circRNA_405721 | | 25070500 | chr19 | - | 5713144 | 5715051 | intronic | ENST00000586617 | LONP1 | CATGATCGTCATGGGACACAGAAGGCGTTCCCAAACCATGTTGATTAGTATCCAGGATGC |
| ASCRP3004731 | 0.685037758 | 0.942540855 | 1.010414 | down | hsa_circRNA_401507 | | 25242744 | chr16 | - | 983990 | 1004666 | exonic | NR_033646 | LMF1 | CATGATTGTCGGCATCTCACAGTCGTGGCATTCCTGGTGGCTTTCCATCAGAACAAGCAG |
| ASCRP3004734 | 0.415255155 | 0.91119147 | 1.0309375 | down | hsa_circRNA_100703 | hsa_circ_0020303 | circBase | chr10 | - | 125771848 | 125806240 | exonic | NM_015892 | CHST15 | CATGCACAAGGTCTTCCAGTTTCTGAACCTAGGAAATCTGGCATTTTTTAAAGTTTGCGC |
| ASCRP3004736 | 0.144525263 | 0.909237851 | 1.0424122 | down | hsa_circRNA_100631 | hsa_circ_0006148 | circBase | chr10 | + | 76910271 | 76910864 | exonic | NM_144660 | SAMD8 | CATGCAGACCTATCCACCACTCCCAGATATATTCTTAGACAGGCAGCGGAGGAGGAAATG |
| ASCRP3004740 | 0.428391639 | 0.91119147 | 1.0593171 | down | hsa_circRNA_101741 | hsa_circ_0005835 | circBase | chr16 | + | 19627435 | 19628130 | exonic | NM_020314 | C16orf62 | CATGCCCCCGAGGTGGGAATGGAAGTGGCCCCACATCTCAAAGAAACCCTAAATAAGAAC |
| ASCRP3004741 | 0.512850955 | 0.916805276 | 1.0160014 | down | hsa_circRNA_104909 | hsa_circ_0088457 | circBase | chr9 | - | 126371714 | 126641300 | exonic | NM_020946 | DENND1A | CATGCCCTACCTCATAGGAATCCATTTAAGTTTAATGGAGGCAGAATCCAGAGACCACAT |
| ASCRP3004743 | 0.63939013 | 0.929544944 | 1.0476491 | down | hsa_circRNA_052165 | hsa_circ_0052165 | circBase | chr19 | + | 55420603 | 55420882 | exonic | NM_004829 | NCR1 | CATGCCTGGTCTTTCCCCAGTGAGCCAGTGAAGCTCCTGGTCACAGAAATGTATGACACA |
| ASCRP3004747 | 0.038440763 | 0.909237851 | 1.0595257 | down | hsa_circRNA_405385 | | 25070500 | chr15 | + | 71761715 | 71762097 | intronic | ENST00000261862 | THSD4 | CATGCTGGTCTCGCTGTTATTTTCCAGCTCTCAGAATGCAGCTGCAGGTTCACAAGTCAG |
| ASCRP3004749 | 0.389592371 | 0.91119147 | 1.0297793 | down | hsa_circRNA_407179 | | 25070500 | chr9 | - | 37511920 | 37512718 | sense overlapping | NM_012166 | FBXO10 | CATGGAAAGGCTGCCCCAGGGATGACAAGAGGGACATGCTGAAACAGGTCTGATACGTGG |
| ASCRP3004757 | 0.585876206 | 0.927905266 | 1.0135235 | down | hsa_circRNA_104935 | hsa_circ_0003362 | circBase | chr9 | - | 131860291 | 131860929 | exonic | NM_000755 | CRAT | CATGGACTCACTCACCTTTGTCAAGGCCATGGATGACTCCAGCGTCACGGAAGAAACCCG |
| ASCRP3004762 | 0.538875964 | 0.919549314 | 1.05544 | down | hsa_circRNA_008587 | hsa_circ_0008587 | circBase | chr8 | - | 90734242 | 90737869 | exonic | NR_125822 | LOC101929709 | CATGGAGATGCACCTCTCTCAAGTCTTGAACAATGTTTGAGTTCATTGGAAATTTCTGCT |
| ASCRP3004764 | 0.927366303 | 0.985498678 | 1.0054069 | down | hsa_circRNA_400092 | hsa_circ_0092287 | circBase | chr7 | + | 101189031 | 101190071 | intronic | ENST00000313669 | COL26A1 | CATGGAGGGCTTCCCCAAGCCCTGCTTTTGGCCCCAAAGTGGCCTGTCTCTGCCTCAGTG |
| ASCRP3004765 | 0.156618011 | 0.909237851 | 1.0647815 | down | hsa_circRNA_104311 | hsa_circ_0007140 | circBase | chr7 | - | 6736969 | 6744854 | exonic | NM_006956 | ZNF12 | CATGGATAGTAGAAGGAGAATTCCTACTTCAGAGCTATCCAGATCTGCCTCCGCTTTCAC |
| ASCRP3004767 | 0.997658136 | 0.99972787 | 1.0001242 | down | hsa_circRNA_100616 | hsa_circ_0004606 | circBase | chr10 | + | 72462067 | 72468534 | exonic | NM_080722 | ADAMTS14 | CATGGCAAGGAGCATGTGCAGAACTATGTCCTCACCCTCATGAATATCGCGGGCCTCATC |
| ASCRP3004768 | 0.01637544 | 0.909237851 | 1.0408082 | down | hsa_circRNA_000663 | hsa_circ_0000663 | circBase | chr16 | - | 424004 | 427848 | exonic | NM_021259 | TMEM8A | CATGGCACGGCTCAAGACAGTCCTGAAATACAGGTGGGGCTGGTGTCCGAGCACTTCTCG |
| ASCRP3004771 | 0.16677867 | 0.909237851 | 1.0683161 | down | hsa_circRNA_104720 | hsa_circ_0006566 | circBase | chr8 | + | 144771373 | 144772293 | exonic | NM_173831 | ZNF707 | CATGGCCCAGTTGCTGAACCTGTTTGCATGAGTTGCTCCTGACGGCCCTTTAGGATACTT |
| ASCRP3004777 | 0.047288557 | 0.909237851 | 1.0835614 | down | hsa_circRNA_050125 | hsa_circ_0050125 | circBase | chr19 | - | 18650180 | 18654383 | exonic | NM_012181 | FKBP8 | CATGGTCACTGCTGACTCCAAGTACTGCTACGGCCCCCAAGGCAGGGCCGAACCTGGTTC |
| ASCRP3004780 | 0.310552647 | 0.909237851 | 1.1046943 | down | hsa_circRNA_051638 | hsa_circ_0051638 | circBase | chr19 | - | 47285639 | 47291842 | exonic | NM_005628 | SLC1A5 | CATGGTTCTGGTCTCCTGGATCATGTGCGGCACGCCCGGGAGGCTTTCTCTGGCTGGTAA |
| ASCRP3004781 | 0.341453938 | 0.91119147 | 1.0259278 | down | hsa_circRNA_001955 | hsa_circ_0001202 | circBase | chr22 | + | 17525762 | 17528316 | exonic | NR_015352 | CECR7 | CATGTAAAGTCAGTGACAGCCCCATGGTAACCCAGAGAACACAGCCGAAGTGGAATGTGG |
| ASCRP3004783 | 0.010897633 | 0.909237851 | 1.1380546 | down | hsa_circRNA_103862 | hsa_circ_0072758 | circBase | chr5 | + | 68467096 | 68470236 | exonic | NM_031966 | CCNB1 | CATGTACATGACTGTCTCCATTATTGATCGGTTCATGCAGGTTGATACTGCCTCTCCAAG |
| ASCRP3004787 | 0.832429349 | 0.978181218 | 1.0059627 | down | hsa_circRNA_100724 | hsa_circ_0020460 | circBase | chr10 | + | 128850944 | 128860040 | exonic | NM_001380 | DOCK1 | CATGTCAGACCAGACCGTCCGGGTGAAGGAAGTTGACAAAAGTGTTGAAGAACTACGTGG |
| ASCRP3004789 | 0.588128841 | 0.927905266 | 1.0169407 | down | hsa_circRNA_000987 | hsa_circ_0001827 | circBase | chr8 | + | 142139086 | 142139206 | intronic | ENST00000262585 | DENND3 | CATGTCTTTTGAGAGCTGCGAACTTGGTCCGCCGGCCGGGAATCTGGGGGTGCCCTGGAG |
| ASCRP3004794 | 0.012764798 | 0.909237851 | 1.1721124 | down | hsa_circRNA_102804 | hsa_circ_0056139 | circBase | chr2 | + | 113258782 | 113278002 | exonic | NM_153712 | TTL | CATGTGCTCAGTGAAGGCATTCTCATCTCCTCAGAGGCTTCAGAGCTTCTCGATTTCATA |
| ASCRP3004801 | 0.607069451 | 0.929544944 | 1.0159008 | down | hsa_circRNA_070605 | hsa_circ_0070605 | circBase | chr4 | + | 107246141 | 107253040 | exonic | NM_004757 | AIMP1 | CATGTTCCTCTTGAACAGGATTTTCTGCCGTCTCTTGGCAAAAATGGCAAATAATGATGC |
| ASCRP3004805 | 0.164157761 | 0.909237851 | 1.0801018 | down | hsa_circRNA_105044 | hsa_circ_0001952 | circBase | chrX | - | 154018228 | 154020560 | exonic | NM_002436 | MPP1 | CATTAAAAGTAATTCCCAACCAGCAAAGCCGTCTTCCTGCACTACAGGCTGTATCGCATC |
| ASCRP3004808 | 0.21130553 | 0.909237851 | 1.057276 | down | hsa_circRNA_103038 | hsa_circ_0060055 | circBase | chr20 | - | 33866724 | 33872064 | sense overlapping | NM_002212 | EIF6 | CATTAAAGTGCAGTTCCCTCCGGTGTGTTCGAGGGCGAGCTCTCCGATACCATCCCCGTG |
| ASCRP3004810 | 0.197667288 | 0.909237851 | 1.0837609 | down | hsa_circRNA_092108 | hsa_circ_0092108 | circBase | chrX | - | 153759605 | 153764260 | exonic | uc004flx.1 | G6PD | CATTAAATCCGCAAACAGCCGTGGCTGTTCCGGGATGGCCTTCTGCCCGAAAACACCTTC |
| ASCRP3004813 | 0.787801366 | 0.967199246 | 1.0122289 | down | hsa_circRNA_000133 | hsa_circ_0000133 | circBase | chr1 | - | 153637695 | 153641033 | exonic | NM_004515 | ILF2 | CATTACAACAGTGCCACCCAATCTTCGAAAACTGGATCCAGAACTCCATTTGTGAAATGG |
| ASCRP3004824 | 0.080240016 | 0.909237851 | 1.058319 | down | hsa_circRNA_092534 | hsa_circ_0001372 | circBase | chr3 | - | 185643369 | 185644451 | sense overlapping | NM_004593 | TRA2B | CATTATACCCGGTCACGGTCTCGCTCCAAGGAAGATTCCAGGCGTTCCAGATCAAAGTCC |
| ASCRP3004835 | 0.383972634 | 0.91119147 | 1.0806117 | down | hsa_circRNA_002122 | hsa_circ_0002122 | circBase | chr16 | + | 67644726 | 67646024 | exonic | NM_006565 | CTCF | CATTCAGAACAGTCACCCTCCTGAGGAATCACCTTAACACACACACAGGCAGGGGAAATG |
| ASCRP3004837 | 0.818806262 | 0.976440085 | 1.0059491 | down | hsa_circRNA_101636 | hsa_circ_0036667 | circBase | chr15 | - | 88726648 | 88727530 | exonic | NM_002530 | NTRK3 | CATTCAGCCCAGAGCCTTTGCCAAGAACCCCCATTTGCGTTATATACACATAGAGAACTG |
| ASCRP3004843 | 0.138591259 | 0.909237851 | 1.0321332 | down | hsa_circRNA_103953 | hsa_circ_0074101 | circBase | chr5 | + | 137708362 | 137708530 | exonic | NM_016604 | KDM3B | CATTCGAGTCTCCATTGCACAATGGCCAGCCCTGATCTTTGTAGAATTTGATGGCTGTAA |
| ASCRP3004861 | 0.021574266 | 0.909237851 | 1.1508916 | down | hsa_circRNA_092474 | hsa_circ_0000959 | circBase | chr19 | + | 55748067 | 55748333 | antisense | NM_014931 | PPP6R1 | CATTGAGGGAGAAGGTGATGTTGGCTGTCTTGTCAAAAGGCGCCATCTTCTCCATCAGAC |
| ASCRP3004862 | 0.922832228 | 0.983668077 | 1.0059885 | down | hsa_circRNA_004046 | hsa_circ_0004046 | circBase | chr3 | + | 15070092 | 15071959 | exonic | NM_003298 | NR2C2 | CATTGAGGTTGAAGGCCCCCTCCTTTCAGACACACACGTCACATTTAAGGCTGAAACAAG |
| ASCRP3004865 | 0.549843823 | 0.919549314 | 1.0984817 | down | hsa_circRNA_102053 | hsa_circ_0043284 | circBase | chr17 | + | 35802664 | 35804870 | exonic | NM_001488 | TADA2A | CATTGATTTTGTTGAAGATGACTCGGACATTTTACATGCTACAGATGACCCTCCCCGACC |
| ASCRP3004866 | 0.231989723 | 0.909237851 | 1.0532506 | down | hsa_circRNA_103547 | hsa_circ_0003759 | circBase | chr3 | + | 188326948 | 188327632 | exonic | NM_005578 | LPP | CATTGCAGCCAAAGAGCTCCACTGGTTCAACAGCCTCTCCTCCAGTTTCGACCCCAGTCA |
| ASCRP3004871 | 0.204335847 | 0.909237851 | 1.1036199 | down | hsa_circRNA_041100 | hsa_circ_0041100 | circBase | chr16 | + | 89961445 | 89977071 | exonic | NM_014972 | TCF25 | CATTGCTCTCTTCTTCCGGTCACTGTTGCCAAACTATACCATGGAGGTTCTGCTCCAGAC |
| ASCRP3004875 | 0.013550528 | 0.909237851 | 1.0923891 | down | hsa_circRNA_028935 | hsa_circ_0028935 | circBase | chr12 | - | 121220457 | 121221555 | exonic | NM_139015 | SPPL3 | CATTGGCTTCTCATGGATGTTCTTGCAACGATAGCTTTTGCTTTTCTTCTCCTCCCGATG |
| ASCRP3004876 | 0.486745556 | 0.91119147 | 1.0403404 | down | hsa_circRNA_104816 | hsa_circ_0087493 | circBase | chr9 | - | 95018961 | 95048121 | exonic | NM_002161 | IARS | CATTGGCTTCTTTGAGACTGAAATGGCAGGTGGGTCTTCAAACAACTCTATGATAAAGGC |
| ASCRP3004902 | 0.310481795 | 0.909237851 | 1.0492418 | down | hsa_circRNA_076138 | hsa_circ_0076138 | circBase | chr6 | + | 35436177 | 35438558 | exonic | NM_007104 | RPL10A | CATTTGAATAAATTCTATTACCAGTTCAGTCTCTTTTCCGGTTAGCGCGGCGTGAGAAGC |
| ASCRP3004905 | 0.074865564 | 0.909237851 | 1.0707786 | down | hsa_circRNA_000158 | hsa_circ_0000158 | circBase | chr1 | - | 169673708 | 169673894 | exonic | NM_000655 | SELL | CATTTGGAAACTGGTCATCTCCAGAACCAACCTGTCAAGTGATTCAGTGTGAGCCTTTGG |
| ASCRP3004907 | 0.658987941 | 0.933884861 | 1.0122619 | down | hsa_circRNA_100595 | hsa_circ_0007016 | circBase | chr10 | + | 51579127 | 51581378 | exonic | NM_005437 | NCOA4 | CATTTGGGTCTCTCAAAACCATTGAGCAGTGAGGAGAATGAATACCTTCCAAGACCAGAG |
| ASCRP3004910 | 0.666430639 | 0.937594692 | 1.0204629 | down | hsa_circRNA_005187 | hsa_circ_0005187 | circBase | chr5 | + | 96767447 | 96775322 | sense overlapping | ENST00000504578 | RP11-155G15.2 | CATTTTCAGTTTGACAAGAGGTAGAATATGGTGATGGGTGCTGTGCAAGAAGAAGGAGGG |
| ASCRP3004912 | 0.202630199 | 0.909237851 | 1.0834672 | down | hsa_circRNA_001169 | hsa_circ_0001169 | circBase | chr20 | + | 48804894 | 48805015 | antisense | NR_125739 | CEBPB-AS1 | CATTTTGCCTAAGAACACACAGCAAATCCTCCAATTAGCTTCAGTTCCCCCATATCTCCC |
| ASCRP3004927 | 0.697632487 | 0.944102043 | 1.0120943 | down | hsa_circRNA_103371 | hsa_circ_0002023 | circBase | chr3 | - | 48215773 | 48219478 | exonic | NM_001789 | CDC25A | CCAAAGAGTCAACTAATCCAGAGAAGGCCCATGAGCTTTCCTCAAATGAAAGAGATAGCA |
| ASCRP3004937 | 0.704707335 | 0.945024804 | 1.011749 | down | hsa_circRNA_104850 | hsa_circ_0087855 | circBase | chr9 | + | 110062421 | 110068928 | exonic | NM_002874 | RAD23B | CCAACAGCAACTGACAGGTGAAAGCACTGAAAGAGAAGATTGAATCTGAAAAGGGGAAAG |
| ASCRP3004938 | 0.760325765 | 0.960870011 | 1.0175552 | down | hsa_circRNA_100391 | hsa_circ_0015243 | circBase | chr1 | + | 171548492 | 171556890 | exonic | NM_015172 | PRRC2C | CCAACATCTAGTCCCTTCCGCTCAAATCCCAGCCTTCTATATGGACACAAGTCATTTATT |
| ASCRP3004940 | 0.175004595 | 0.909237851 | 1.0499571 | down | hsa_circRNA_100649 | hsa_circ_0019223 | circBase | chr10 | + | 95790439 | 95792009 | exonic | NM_016341 | PLCE1 | CCAACGTCTGTCAGAAGCCCAGTGGTATCCTGAAACAGGAATCATTCAAACTGGATTTTA |
| ASCRP3004945 | 0.874464595 | 0.979863126 | 1.0068094 | down | hsa_circRNA_101067 | hsa_circ_0003729 | circBase | chr12 | + | 52380601 | 52385777 | exonic | NM_004302 | ACVR1B | CCAACTGGTGGCAGAGTTATGAGATACATGGCCCCTGAAGTACTTGATGAAACCATTAAT |
| ASCRP3004947 | 0.855386581 | 0.978900086 | 1.0056225 | down | hsa_circRNA_103014 | hsa_circ_0003209 | circBase | chr20 | + | 30370051 | 30385318 | exonic | NM_012112 | TPX2 | CCAAGAAAGAGAAGAAATCAGTTGCTGACCTGTTACCCTCCAAATCTTCTGTGACCAAGA |
| ASCRP3004950 | 0.519944915 | 0.916971445 | 1.0283272 | down | hsa_circRNA_004498 | hsa_circ_0004498 | circBase | chr10 | - | 121335202 | 121340050 | sense overlapping | NM_003252 | TIAL1 | CCAAGCCGGATATGCTGCCCAAACTCCAAAATGATGGTAGAGAATTGACCAAGAAAAATA |
| ASCRP3004952 | 0.790021633 | 0.967422419 | 1.0275746 | down | hsa_circRNA_405635 | | 25070500 | chr17 | + | 78022381 | 78032779 | exonic | NM_017950 | CCDC40 | CCAAGCTGAGGACACCCGGATTTTAAGGAAAGCAGTGAGTGAGTGATCCCCCCAGGGGTG |
| ASCRP3004956 | 0.774467392 | 0.964265101 | 1.0177 | down | hsa_circRNA_406178 | | 25070500 | chr22 | + | 29240114 | 29242500 | sense overlapping | ENST00000458080 | CTA-292E10.6 | CCAAGGTGGATTCACTATGGCCCTGATCTCATGATTGGGGTTTTATTCTGGGCATATATA |
| ASCRP3004961 | 0.518332737 | 0.916971445 | 1.0206168 | down | hsa_circRNA_088478 | hsa_circ_0088478 | circBase | chr9 | - | 126531792 | 126641300 | exonic | NM_020946 | DENND1A | CCAAGTTTTGTTTCCCCTTCTATGTGGACAGGCAGAATCCAGAGACCACATTTGAAGTAT |
| ASCRP3004964 | 0.993772489 | 0.999076026 | 1.0007201 | down | hsa_circRNA_003779 | hsa_circ_0003779 | circBase | chr10 | + | 104352338 | 104359301 | exonic | NM_016169 | SUFU | CCAATCAACCCTCAGCGGCAGAATGGCCTCGCCCACGACCGGGCCCCAGAACACCTTCTG |
| ASCRP3004965 | 0.001699177 | 0.870215632 | 1.0481788 | down | hsa_circRNA_401845 | | 25242744 | chr17 | + | 58012553 | 58018304 | exonic | NM_003161 | RPS6KB1 | CCAATCAGGTCTTTCTGCCCCCATTCACTGGGGAGAATAGAAAGAAAACAATTGACAAAA |
| ASCRP3004967 | 0.480065333 | 0.91119147 | 1.027008 | down | hsa_circRNA_005600 | hsa_circ_0005600 | circBase | chr17 | + | 57430575 | 57430887 | exonic | NM_001005404 | YPEL2 | CCAATCATGATGAACTAATTTCCAAGGCTGCTGAGAACTAGCCCTAGACCTCTGCGTGAG |
| ASCRP3004970 | 0.586678704 | 0.927905266 | 1.0277867 | down | hsa_circRNA_037076 | hsa_circ_0037076 | circBase | chr15 | + | 101549041 | 101569437 | exonic | NM_024652 | LRRK1 | CCAATGACAGGCTCTCCGTGTGAAATGGTCCCATCTCAGACTGCCCTGGGTAGACCTAGA |
| ASCRP3004972 | 0.066299298 | 0.909237851 | 1.0627304 | down | hsa_circRNA_100252 | hsa_circ_0007009 | circBase | chr1 | + | 65131739 | 65141666 | exonic | NM_020925 | CACHD1 | CCAATGCAGAGAACCGGTATCTCCATGCAGTAGCTAATCCAGGGTTGATTTCTTTGACTG |
| ASCRP3004985 | 0.513567039 | 0.916971445 | 1.0209492 | down | hsa_circRNA_400020 | hsa_circ_0092372 | circBase | chr11 | + | 9450336 | 9450536 | intronic | ENST00000379719 | IPO7 | CCACACACAGAACTCAGGGAATAGAGATTAGCTTGGGGGAATTTAGGTTTCATCTGTGTC |
| ASCRP3004990 | 0.940749119 | 0.989616014 | 1.0043185 | down | hsa_circRNA_401823 | | 25242744 | chr17 | + | 49340634 | 49350811 | exonic | NM_016001 | UTP18 | CCACATCAACTTCTCTTCCAAGAGGAATCTTGAAGGTTCAAGAACATGAAGACTCGGGTG |
| ASCRP3004998 | 0.949268159 | 0.992077624 | 1.001749 | down | hsa_circRNA_101347 | hsa_circ_0008002 | circBase | chr14 | - | 50130032 | 50141145 | exonic | NM_002692 | POLE2 | CCACCCACTGAGCCCTCTAGTACTACTAGAGAGCACGTTTTCAATATCATAGGAGCATTT |
| ASCRP3004999 | 0.102255799 | 0.909237851 | 1.0642103 | down | hsa_circRNA_046598 | hsa_circ_0046598 | circBase | chr17 | - | 80914570 | 80923622 | exonic | NM_001009905 | B3GNTL1 | CCACCCTTCGTCATCTGCGTGAAGCTGGTTTTCACCTCAAATGGCCCCACGGTGATCATG |
| ASCRP3005008 | 0.522070357 | 0.916971445 | 1.1014744 | down | hsa_circRNA_401432 | | 25242744 | chr15 | - | 65040617 | 65043837 | exonic | NM_194272 | RBPMS2 | CCACGCTCAGGTCCGGACACTGTTTGTCAGCGGCCTCCCTGTGGACATTAAACCCAGAGA |
| ASCRP3005010 | 0.099863886 | 0.909237851 | 1.0490444 | down | hsa_circRNA_092391 | hsa_circ_0000361 | circBase | chr11 | - | 118764529 | 118765102 | sense overlapping | ENST00000334801 | BCL9L | CCACGGCCAGGGTGTCCAGGAAGATGACGATGTGGTAGGGTGACCAGCAGAGGAAGAAGA |
| ASCRP3005013 | 0.074184558 | 0.909237851 | 1.0483285 | down | hsa_circRNA_406013 | | 25070500 | chr2 | - | 198281464 | 198285857 | exonic | NM_012433 | SF3B1 | CCACTCCCAAAAAACTATCAAGTTGGGATCAGGCAGAGGATGACGATGACTATTCATCAT |
| ASCRP3005014 | 0.120176834 | 0.909237851 | 1.124161 | down | hsa_circRNA_004163 | hsa_circ_0004163 | circBase | chr18 | + | 662145 | 671451 | exonic | NM_001071 | TYMS | CCACTGAAAATTCAGGGATCCACAAATGCTAAAGAGCTGTCTTCCAAGGGAGTGAAAATC |
| ASCRP3005024 | 0.056465544 | 0.909237851 | 1.1303418 | down | hsa_circRNA_102404 | hsa_circ_0048259 | circBase | chr19 | - | 1510148 | 1510916 | exonic | uc010dsl.2 | ADAMTSL5 | CCAGAAGACCTACCAGTGGGTGCCCTTCCATGGGGGCCCCACCTCTTCCAGAACCTCCTG |
| ASCRP3005033 | 0.44643911 | 0.91119147 | 1.0246896 | down | hsa_circRNA_092432 | hsa_circ_0000684 | circBase | chr16 | + | 27373960 | 27374248 | intronic | ENST00000565352 | IL4R | CCAGACACCTGGAGGAATCATGCCTTCTTCCACCTTCGGGAAGTACGAGTGCTCACATGC |
| ASCRP3005034 | 0.204819946 | 0.909237851 | 1.0934092 | down | hsa_circRNA_405094 | | 25070500 | chr12 | + | 123078822 | 123109235 | exonic | NM_014708 | KNTC1 | CCAGACACTGCTCCCTGTGAAATTCTGAAGATTTCTTTTCAACCAGTTTTCAGGCAACAT |
| ASCRP3005035 | 0.958874903 | 0.993354918 | 1.0012186 | down | hsa_circRNA_001882 | hsa_circ_0001142 | circBase | chr20 | - | 33148403 | 33245340 | sense overlapping | NM_080476 | PIGU | CCAGACAGGAAGGAGAAGAGGCAGGCATAAATGAGACATTTTTGAGAGTTGGTAAGAGTT |
| ASCRP3005037 | 0.125327342 | 0.909237851 | 1.0488512 | down | hsa_circRNA_000736 | hsa_circ_0000736 | circBase | chr17 | + | 3839681 | 3840741 | antisense | NM_005173 | ATP2A3 | CCAGACGACCTCGCCAACATTGGAGGAGATGAGGCCGTCTGTCACCAGGTTCACCCAGAG |
| ASCRP3005049 | 0.051756988 | 0.909237851 | 1.120741 | down | hsa_circRNA_104165 | hsa_circ_0077526 | circBase | chr6 | - | 105563560 | 105564743 | exonic | NM_007073 | BVES | CCAGCATGTCCTCTCTTCGTCACCATTATTGCAGATGATAACTGCAGATTTTTATGCTGG |
| ASCRP3005053 | 0.186779711 | 0.909237851 | 1.1256121 | down | hsa_circRNA_406252 | | 25070500 | chr3 | + | 32774914 | 32778982 | exonic | NM_015442 | CNOT10 | CCAGCTCCACCTTCTTCTCCATTGAGAAAACAGGAATTAGAAAACTTAAAACTTCTGAAC |
| ASCRP3005058 | 0.620458012 | 0.929544944 | 1.0164603 | down | hsa_circRNA_007848 | hsa_circ_0007848 | circBase | chr1 | + | 231930987 | 231954263 | exonic | NM_018662 | DISC1 | CCAGGAGACTGCCTATGCCTCCAGGAAAGAATAAAATCCCTCAACTTGTCACTTAAAGAA |
| ASCRP3005060 | 0.653279899 | 0.933189368 | 1.026551 | down | hsa_circRNA_103456 | hsa_circ_0067127 | circBase | chr3 | - | 125843206 | 125856803 | exonic | NM_012190 | ALDH1L1 | CCAGGATCTGGCTGGTGGAGGAAGTGAAGGAGCTGTGTGATGGCCTGGAGTTAGAAAATG |
| ASCRP3005061 | 0.645862309 | 0.931243329 | 1.01134 | down | hsa_circRNA_073716 | hsa_circ_0073716 | circBase | chr5 | - | 123974825 | 124036962 | exonic | NM_020747 | ZNF608 | CCAGGCATCTGCATCTGGAATGTTTCCTGGACAAAGAAGGTTGACCCCCTGTTTACAGTG |
| ASCRP3005063 | 0.146264282 | 0.909237851 | 1.1082742 | down | hsa_circRNA_101834 | hsa_circ_0039914 | circBase | chr16 | + | 68087466 | 68088520 | exonic | NM_017803 | DUS2 | CCAGGCTTGTAGGTGCTCAGCACAGTGGACTTTGTCGCCCCTGATGATCGAGTTGTCTTC |
| ASCRP3005070 | 0.630957384 | 0.929544944 | 1.0543742 | down | hsa_circRNA_102562 | hsa_circ_0051258 | circBase | chr19 | - | 42482087 | 42482950 | exonic | NM_152296 | ATP1A3 | CCAGGTTAACCCCCGCTCTCCATCCATGAGACCGAGGACCCCAACGACAACCGATACCTG |
| ASCRP3005073 | 0.19325327 | 0.909237851 | 1.0468817 | down | hsa_circRNA_102038 | hsa_circ_0043082 | circBase | chr17 | + | 33310020 | 33313150 | exonic | NM_002311 | LIG3 | CCAGTACCAATCCCCGGAAATTTTCTGGCTTTTCAGCTATATGTCTTTGGCTTTCAAGAT |
| ASCRP3005077 | 0.22784077 | 0.909237851 | 1.0575471 | down | hsa_circRNA_103856 | hsa_circ_0072684 | circBase | chr5 | - | 64587155 | 64769779 | exonic | NM_197941 | ADAMTS6 | CCAGTATGGAGCAACCTCCCGCCAATGTAAATATGGGGTTAAAAAATGGCCATACTTTAG |
| ASCRP3005086 | 0.470465265 | 0.91119147 | 1.0605152 | down | hsa_circRNA_101864 | hsa_circ_0040356 | circBase | chr16 | - | 71710349 | 71713438 | exonic | NM_015020 | PHLPP2 | CCAGTTCCAACAATTTTCTCAACTGAAGGGCCTGAACTTGTCCCATAATAAACTTGGGTT |
| ASCRP3005093 | 0.73777914 | 0.953746099 | 1.0087512 | down | hsa_circRNA_405940 | | 25070500 | chr2 | - | 101876363 | 101877994 | antisense | NM_017546 | CNOT11 | CCATACCCAGGAGACACACTTTCACACGCGCCCCAGAATAATGTGCAAGAATGTTCACAG |
| ASCRP3005096 | 0.035320415 | 0.909237851 | 1.0600037 | down | hsa_circRNA_404489 | | 25070500 | chr1 | + | 41461588 | 41463198 | exonic | NM_001905 | CTPS1 | CCATCAACCACAAATTGGAAATCAAGGTGATCTGTGTCCACGATGTCTCATCCATCTACC |
| ASCRP3005097 | 0.810639051 | 0.974895666 | 1.0123513 | down | hsa_circRNA_006700 | hsa_circ_0006700 | circBase | chr15 | + | 31200320 | 31206294 | exonic | NM_014967 | FAN1 | CCATCAATCGGAAAACCCACATCTTCCAAGACAGAGATGATCTTATCAGCTACTGGTCAG |
| ASCRP3005102 | 0.106202475 | 0.909237851 | 1.217872 | down | hsa_circRNA_406487 | | 25070500 | chr4 | + | 68664356 | 68668137 | intronic | ENST00000500538 | UBA6-AS1 | CCATCCTCTCCTGAGTGCTGAGTAAGAAGTTTTCTAGCAATCAGTGTCTGACCAGATGAT |
| ASCRP3005107 | 0.331434384 | 0.909237851 | 1.0433481 | down | hsa_circRNA_104282 | hsa_circ_0008039 | circBase | chr7 | - | 716865 | 751164 | exonic | NM_002735 | PRKAR1B | CCATCTCCAAGAACGTGCTCTTCGCTCACCTGGATGACAACGAGAGGAGGAAGGAAGCAG |
| ASCRP3005108 | 0.464740734 | 0.91119147 | 1.0301166 | down | hsa_circRNA_100477 | hsa_circ_0016867 | circBase | chr1 | + | 230798886 | 230807386 | exonic | NM_007357 | COG2 | CCATCTCCAGAGCCTTAGATCGTCTGTCAGTGAAGGAATTCGGGCAGTTGATGAACGAAT |
| ASCRP3005119 | 0.22362616 | 0.909237851 | 1.0339812 | down | hsa_circRNA_100059 | hsa_circ_0010020 | circBase | chr1 | - | 12632755 | 12639440 | exonic | NM_004753 | DHRS3 | CCATGGACAATGCATGCCCTCGTTATCTTGAAAAGGTGGGTGACATCACCATCCTGGTGA |
| ASCRP3005121 | 0.696602569 | 0.944102043 | 1.015754 | down | hsa_circRNA_404211 | | 25242744 | chr9 | - | 99589361 | 99607298 | exonic | NM_001001662 | ZNF782 | CCATGGTTATTAGAGAAAGAGAAAGGATTTCTAAGCAGGAACTCCCCAGGCATCAGTGTC |
| ASCRP3005131 | 0.123299831 | 0.909237851 | 1.1796919 | down | hsa_circRNA_401576 | | 25242744 | chr16 | + | 25151491 | 25162936 | exonic | NM_016309 | LCMT1 | CCATTCTAGAACTGCATTCAGAGGACACACTTCAGATGGGATGAAGATCTTCTCCCAAGT |
| ASCRP3005151 | 0.306883336 | 0.909237851 | 1.0798577 | down | hsa_circRNA_055953 | hsa_circ_0055953 | circBase | chr2 | - | 107446521 | 107450602 | exonic | NM_032528 | ST6GAL2 | CCCACCATCTTCTGGTTTCATTGATTCTCATGATGCGGTTTTGAGATTTAACTCTGCTCC |
| ASCRP3005152 | 0.225132684 | 0.909237851 | 1.0862682 | down | hsa_circRNA_065215 | hsa_circ_0065215 | circBase | chr3 | - | 47466974 | 47484581 | exonic | NM_012235 | SCAP | CCCACCCTCAATGGCGGGTACCTGCACATGTTGTTCTTTGTCAGTGCTGTCAAGTGTGTG |
| ASCRP3005154 | 0.590999262 | 0.927905266 | 1.023183 | down | hsa_circRNA_091840 | hsa_circ_0091840 | circBase | chrX | - | 153576899 | 153583440 | exonic | NM_001456 | FLNA | CCCACTAGTTCTCTTCTCCAGCCAAGAGGAATAAAGTTTTGCTTCCATTGTGCTGGCATC |
| ASCRP3005167 | 0.869192491 | 0.979863126 | 1.0112865 | down | hsa_circRNA_007647 | hsa_circ_0007647 | circBase | chr2 | - | 242572331 | 242573494 | exonic | NM_015963 | THAP4 | CCCAGCCGCGGTTCCCCCTAAAGGACTCAAAACGTCTAATCCAATGGTTAAAAGCTGTTC |
| ASCRP3005172 | 0.432209805 | 0.91119147 | 1.0507 | down | hsa_circRNA_000675 | hsa_circ_0001117 | circBase | chr2 | + | 240910625 | 240910719 | antisense | NM_004544 | NDUFA10 | CCCAGGTACATCGCAAGGGTCTCCAAGTAGATATATCTCAGGAAATGTCATTCAGGAGAA |
| ASCRP3005174 | 0.121340923 | 0.909237851 | 1.0552551 | down | hsa_circRNA_035537 | hsa_circ_0035537 | circBase | chr15 | - | 59963381 | 59964938 | exonic | NM_004330 | BNIP2 | CCCAGTCTGGGATGGCTCAGGAAATGTTATCAGCAAATTGATAGAAGGATATTATGGGGA |
| ASCRP3005175 | 0.843370129 | 0.978270321 | 1.0084052 | down | hsa_circRNA_403966 | | 25242744 | chr7 | + | 148863249 | 148864022 | exonic | NM_020781 | ZNF398 | CCCAGTGAAGGTGCCTGTGGCATTTGATGATGTCTCCATCTACTTTTCCACTCCAGAGTG |
| ASCRP3005183 | 0.208259169 | 0.909237851 | 1.0616518 | down | hsa_circRNA_103283 | hsa_circ_0064220 | circBase | chr3 | + | 10088263 | 10091189 | exonic | NM_033084 | FANCD2 | CCCATCTGCTATGATGATGAATGCTGTCTTTGTAAAGGTGTTTGACCTGGTGATGCTTTT |
| ASCRP3005186 | 0.361622168 | 0.91119147 | 1.0278387 | down | hsa_circRNA_063226 | hsa_circ_0063226 | circBase | chr22 | + | 38071612 | 38075809 | exonic | NM_002305 | LGALS1 | CCCATGGCCCCCAATAAAGGCAGCTGCCTCTGCTCCCTCTGAAAGTTAAAAGGGTGGGAG |
| ASCRP3005193 | 0.782812375 | 0.966660917 | 1.0127112 | down | hsa_circRNA_051096 | hsa_circ_0051096 | circBase | chr19 | + | 41083063 | 41089623 | exonic | uc010xvl.1 | SHKBP1 | CCCCAAGACCAATCCTGCGGGAGGGGGACGCATTCCTCACCCGCTCCTCTTCATCTCTTG |
| ASCRP3005194 | 0.853880648 | 0.978900086 | 1.0056926 | down | hsa_circRNA_101757 | hsa_circ_0008284 | circBase | chr16 | + | 22260075 | 22262643 | exonic | NM_013302 | EEF2K | CCCCAAGCAGGTACAACGCCGTCACCGGGGAATGGCTGGATGATGAAGTTCTGATCAAGA |
| ASCRP3005195 | 0.492371905 | 0.91119147 | 1.0296622 | down | hsa_circRNA_104660 | hsa_circ_0085061 | circBase | chr8 | + | 100146859 | 100182391 | exonic | NM_015243 | VPS13B | CCCCACATGCTGCTCACAGAAATGCAAGTTGAGAGTAGTTATTACAGTCCACAGAAAGTA |
| ASCRP3005196 | 0.578964752 | 0.925149861 | 1.0275968 | down | hsa_circRNA_006187 | hsa_circ_0006187 | circBase | chr6 | + | 73843143 | 73879577 | exonic | NM_019842 | KCNQ5 | CCCCACCACTTAAAACTGTCATTCGAGCTATCAGTCAGAAGCTAAGTTTTAAGGAGCGAG |
| ASCRP3005199 | 0.846632828 | 0.978900086 | 1.0074441 | down | hsa_circRNA_406221 | | 25070500 | chr3 | + | 10268054 | 10280723 | exonic | NM_001570 | IRAK2 | CCCCAGGATGGTGTTGGCCGAGGTCCTCACGGGCATCCCTGCAATGGATAACAACCGAAG |
| ASCRP3005200 | 0.329556871 | 0.909237851 | 1.0318561 | down | hsa_circRNA_104580 | hsa_circ_0004283 | circBase | chr8 | - | 29927157 | 29927575 | exonic | NM_016127 | SARAF | CCCCAGGCTTTAAGTCTGAGTTCACAGTGGGAATGTAAGACGGACTTAGATATTGCATAC |
| ASCRP3005201 | 0.48847692 | 0.91119147 | 1.0360576 | down | hsa_circRNA_405327 | | 25070500 | chr15 | - | 43294752 | 43314999 | exonic | NM_174916 | UBR1 | CCCCATTATTCCTTTGCAACCTCAAAAGATAAACAGGCTTTTCATATTCTGGCATTGGGT |
| ASCRP3005202 | 0.151748767 | 0.909237851 | 1.0935584 | down | hsa_circRNA_400135 | | 25242744 | chr1 | + | 14042035 | 14075982 | exonic | NM_012231 | PRDM2 | CCCCCAAGAGCCGGAAAGGGTTCATGTAATCAAAGAAGTTTCTTTGTTGTGTGTATCTTT |
| ASCRP3005203 | 0.550150509 | 0.919549314 | 1.0256913 | down | hsa_circRNA_086760 | hsa_circ_0086760 | circBase | chr9 | - | 34613547 | 34614765 | exonic | uc003zuw.1 | DCTN3 | CCCCCAATCAGTGTTCTTATTTCAGTGACAATAAACCATAGAGATGACCGTTCCTGAGCA |
| ASCRP3005210 | 0.666599017 | 0.937594692 | 1.021985 | down | hsa_circRNA_405644 | | 25070500 | chr17 | + | 80443372 | 80446231 | sense overlapping | NM_012336 | NARF | CCCCTCAGGCAGTTTCATGTGGTGCTATCTTCATAATAGAAACAAAGACTTCCAAGAGGT |
| ASCRP3005212 | 0.135986294 | 0.909237851 | 1.0360716 | down | hsa_circRNA_404911 | | 25070500 | chr11 | + | 76696686 | 76722419 | exonic | ENST00000527508 | ACER3 | CCCCTGAGAGCTGAAGATTGGCCATGGTTTGAATGTTTCAAGATCAAGAACTCAGTAAAC |
| ASCRP3005213 | 0.650691125 | 0.932533867 | 1.0159661 | down | hsa_circRNA_103267 | hsa_circ_0001258 | circBase | chr22 | + | 50810448 | 50832564 | exonic | NM_014678 | PPP6R2 | CCCCTGGACATGGAGGAGAAGGTCCGCTTCAAGTAAAGAGATTATAAATCTTCCACTGAA |
| ASCRP3005216 | 0.501246683 | 0.914489113 | 1.0191159 | down | hsa_circRNA_067567 | hsa_circ_0067567 | circBase | chr3 | + | 140675368 | 140678382 | exonic | NM_018155 | SLC25A36 | CCCCTTCCAGATGTGGTGGTACAGTGGGAGCTATTCTGACATGTCCACTGGAAGTTGTAA |
| ASCRP3005217 | 0.304355224 | 0.909237851 | 1.0261091 | down | hsa_circRNA_001642 | hsa_circ_0001642 | circBase | chr6 | + | 135507040 | 135524462 | exonic | NM_005375 | MYB | CCCCTTGCAGCATATATAGCAGTGACGAGGATGATGAGGACTTTGAGATGTGTGACCATG |
| ASCRP3005222 | 0.058295278 | 0.909237851 | 1.0541239 | down | hsa_circRNA_084753 | hsa_circ_0084753 | circBase | chr8 | - | 71499129 | 71499411 | exonic | NM_014294 | TRAM1 | CCCGCCTGTTTTATTTTAGCAATGAAAAGTATCAGAAAGGGAAGATATTCCTCGTCAGCT |
| ASCRP3005226 | 0.953374947 | 0.992077624 | 1.0018701 | down | hsa_circRNA_405796 | | 25070500 | chr19 | + | 47861122 | 47865950 | exonic | NM_014681 | DHX34 | CCCGTCCCAGAAATTCGGAGGGTGGCCCTGGACTCGTTGGTGCTGCAGGTTGTGTACCAG |
| ASCRP3005227 | 0.423373804 | 0.91119147 | 1.0776219 | down | hsa_circRNA_050444 | hsa_circ_0050444 | circBase | chr19 | + | 33608730 | 33610023 | exonic | NM_018025 | GPATCH1 | CCCGTCTTCTGCCCCAACCGTCAACAAAGATGTGGACGCACAGGCTGAAGGAGAAGGGAG |
| ASCRP3005230 | 0.043800272 | 0.909237851 | 1.0831072 | down | hsa_circRNA_100968 | hsa_circ_0024553 | circBase | chr11 | + | 118997644 | 119003929 | exonic | NM_015517 | HINFP | CCCGTTTTCGGGTGAAGGCCATGCCGCCTCCTGGGAAAGTTCCCCGAAAGGAGAATCTGT |
| ASCRP3005234 | 0.184207896 | 0.909237851 | 1.0804209 | down | hsa_circRNA_006766 | hsa_circ_0006766 | circBase | chr7 | - | 75104453 | 76279715 | sense overlapping | NM_002991 | CCL24 | CCCTATCCCTTTTCCATATCTCCCAATTCTGATCATGTTGCCATTACAGGGGTAAGTTAG |
| ASCRP3005242 | 0.396652418 | 0.91119147 | 1.035666 | down | hsa_circRNA_404855 | | 25070500 | chr11 | - | 17948284 | 18031686 | sense overlapping | NM_012139 | SERGEF | CCCTCCAGGTGGATTCTGCATCAAGATAGTCTTTTGGAGTAATAGCAAGGTGTCTTCAAA |
| ASCRP3005245 | 0.230306106 | 0.909237851 | 1.0500236 | down | hsa_circRNA_403634 | | 25242744 | chr6 | + | 71242862 | 71248104 | exonic | NM_020819 | FAM135A | CCCTCGCCAAACTTTTTTATATAAGCTTAGTAACAAAGCAGGAAACAGTGCAGATCTCCG |
| ASCRP3005247 | 0.262556532 | 0.909237851 | 1.0702522 | down | hsa_circRNA_100486 | hsa_circ_0001958 | circBase | chr1 | - | 233313547 | 233344435 | exonic | NM_014801 | PCNXL2 | CCCTCTCCCCAAGAAGATGAAAGATTCAGTGGGACACAACCAAATCATAACATATAGCAG |
| ASCRP3005260 | 0.568985169 | 0.92124149 | 1.0080488 | down | hsa_circRNA_103550 | hsa_circ_0001374 | circBase | chr3 | + | 191074876 | 191087825 | exonic | NM_174908 | CCDC50 | CCCTGCAACCCGTGCTTATGCAGATAGTTACTATTATGAAGATGGAGTATGCCGAGATTT |
| ASCRP3005270 | 0.070396372 | 0.909237851 | 1.0456234 | down | hsa_circRNA_406901 | | 25070500 | chr7 | + | 2564328 | 2566555 | exonic | NM_002304 | LFNG | CCCTTCTCGGTGGAGGCCGACCCATCCAGACGTTCATCTTCACTGACGGGGAAGATGAGG |
| ASCRP3005272 | 0.295361652 | 0.909237851 | 1.0848413 | down | hsa_circRNA_082317 | hsa_circ_0082317 | circBase | chr7 | - | 129519407 | 129520811 | exonic | NM_003344 | UBE2H | CCCTTTCAAATCTCCATCTATAGCATCGAGAGTAAACATGAGGTTACGATCCTGGGAGGA |
| ASCRP3005273 | 0.901823159 | 0.981257955 | 1.007023 | down | hsa_circRNA_101753 | hsa_circ_0002696 | circBase | chr16 | + | 21623965 | 21636436 | exonic | NM_016025 | METTL9 | CCCTTTCATCCCTATGTGGAAAACGTGGTATGTGTGCAACAGAGAGAAATTATGCGAATC |
| ASCRP3005274 | 0.066206167 | 0.909237851 | 1.0885292 | down | hsa_circRNA_004210 | hsa_circ_0004210 | circBase | chr7 | + | 156758963 | 156759786 | exonic | NM_138400 | NOM1 | CCCTTTCCATCTTAAAGGCTTGGACTTAAGGATCAGCAGGAGAGAGAAATCATTCACGTT |
| ASCRP3005277 | 0.865675906 | 0.979863126 | 1.008851 | down | hsa_circRNA_005896 | hsa_circ_0005896 | circBase | chr5 | + | 68882182 | 69736583 | sense overlapping | NM_000344 | SMN1 | CCCTTTTTCCCCCTACAGGGGTATTTCAGTTTGGACTAATTTATATCTGTTATAAGTGGT |
| ASCRP3005284 | 0.724476574 | 0.949785489 | 1.0195831 | down | hsa_circRNA_001920 | hsa_circ_0001920 | circBase | chrX | - | 48681622 | 48681706 | antisense | NM_006044 | HDAC6 | CCGACAGCCTCCTCTGAGGTGGTCTGGTCCAGAATGGCTCCTCCCACAGTCTCCTCTGAG |
| ASCRP3005285 | 0.633372324 | 0.929544944 | 1.0634472 | down | hsa_circRNA_400692 | | 25242744 | chr11 | + | 596922 | 598502 | exonic | NM_020901 | PHRF1 | CCGACGGAAGAGGAAGACAAGTACCACATGGAATGCTTGGACCCCCCTCTCCAGGAGGTG |
| ASCRP3005290 | 0.960268082 | 0.993354918 | 1.0021016 | down | hsa_circRNA_104064 | hsa_circ_0007132 | circBase | chr6 | - | 16326624 | 16328701 | exonic | NM_000332 | ATXN1 | CCGAGCCCAGCATCCAGAGCTGCTGTTGGCGGATTGTACCCACGGGGAGATGATTCCTCA |
| ASCRP3005291 | 0.988168388 | 0.998148217 | 1.0005879 | down | hsa_circRNA_401205 | | 25242744 | chr13 | - | 113832487 | 113833403 | exonic | NM_018386 | PCID2 | CCGAGGACGGGTATTTGTTACTGAAAACACACCAGCTGTCTCTGGATGCTTTTCTGGTTG |
| ASCRP3005304 | 0.289027677 | 0.909237851 | 1.0354123 | down | hsa_circRNA_029532 | hsa_circ_0029532 | circBase | chr12 | - | 133245396 | 133249863 | exonic | NM_006231 | POLE | CCGCCTGCAGACTCTGGCCACGTATTCTGTGTCAGATGCTGTCGCCACTTACTACCTGTA |
| ASCRP3005306 | 0.532234627 | 0.919549314 | 1.0321024 | down | hsa_circRNA_407266 | | 25070500 | chrX | + | 10058810 | 10084547 | exonic | NM_015691 | WWC3 | CCGCTTAACATCCTACCTCCAGTCCCAGTTAAAAAGGATTGATCGGAAGATGTCAAGTAC |
| ASCRP3005307 | 0.485115428 | 0.91119147 | 1.1595403 | down | hsa_circRNA_067327 | hsa_circ_0067327 | circBase | chr3 | + | 129811943 | 129813356 | exonic | NM_001136152 | ALG1L2 | CCGCTTCTAGAGTTTGAACAACTGACTCTTGACGGACAGAACCTTCCTTCTCTCGTCTGT |
| ASCRP3005308 | 0.32145479 | 0.909237851 | 1.1817691 | down | hsa_circRNA_020845 | hsa_circ_0020845 | circBase | chr11 | + | 3424822 | 3426193 | exonic | NR_024248 | TSSC2 | CCGCTTCTAGAGTTTGAACAACTGACTCTTGATGGACACAACCTTCCTTCTCTCGTCTGT |
| ASCRP3005313 | 0.227373794 | 0.909237851 | 1.0603111 | down | hsa_circRNA_000662 | hsa_circ_0001171 | circBase | chr20 | - | 55844499 | 55844644 | antisense | uc010gir.3 | BC037891 | CCGGTCTCTTTAGGTCTTGGATATGAAGAGCATGTTGTCTCTTCCTCTTGCATGAGAAAA |
| ASCRP3005315 | 0.583840529 | 0.926329659 | 1.0217343 | down | hsa_circRNA_031900 | hsa_circ_0031900 | circBase | chr14 | + | 51712030 | 51716483 | exonic | NM_030755 | TMX1 | CCGTACCCATACCCTTCAAGACTGAGTGGACGGTTTATCATAACTGCTCTTCCTACTATT |
| ASCRP3005319 | 0.217424939 | 0.909237851 | 1.0599953 | down | hsa_circRNA_401357 | | 25242744 | chr15 | - | 26825465 | 26866681 | exonic | NM_000814 | GABRB3 | CCGTCTGGTCTCGAGGAATGTTGTCTTCGCCACAGGATTATACCTTAACCATGTATTTTC |
| ASCRP3005324 | 0.338157685 | 0.91119147 | 1.0502963 | down | hsa_circRNA_092385 | hsa_circ_0000294 | circBase | chr11 | - | 45880295 | 45904786 | antisense | NM_021117 | CRY2 | CCGTTCTTTCCCAAAGGGTTCAGAGTCATATTCAAAGGTCAAGCGGGTCAGACATTTATA |
| ASCRP3005325 | 0.379212422 | 0.91119147 | 1.1483082 | down | hsa_circRNA_404184 | | 25242744 | chr9 | + | 74533340 | 74562028 | exonic | uc010mov.2 | C9orf85 | CCTAAAAAGTGTCTTTATCTGCAAACTCTAGTCATGATGTTTGAGTTTGTCTCCCTGTGG |
| ASCRP3005335 | 0.906212881 | 0.982324123 | 1.0098655 | down | hsa_circRNA_100551 | hsa_circ_0017728 | circBase | chr10 | + | 12123470 | 12143180 | exonic | NM_018706 | DHTKD1 | CCTACATCCCCCTGAACCATATGGACCCAAATCAGAAGGGGTTTCTAGAGTTGATCATGG |
| ASCRP3005338 | 0.061047252 | 0.909237851 | 1.0853293 | down | hsa_circRNA_101670 | hsa_circ_0037160 | circBase | chr16 | - | 359972 | 364683 | exonic | NM_003502 | AXIN1 | CCTACCTCACATTCCCGTATGGATCCTGGCGGGAGCCAGTCAACCCCTATTATGTCAATG |
| ASCRP3005343 | 0.346593507 | 0.91119147 | 1.0079682 | down | hsa_circRNA_101967 | hsa_circ_0041732 | circBase | chr17 | + | 6350782 | 6351078 | exonic | NM_019013 | FAM64A | CCTACTCCTCAACAGAGCCCCTCTGCTCTCCCAGAGAATCCAGGAGTCCTGCCAAAGTGG |
| ASCRP3005350 | 0.326850723 | 0.909237851 | 1.0532415 | down | hsa_circRNA_406306 | | 25070500 | chr3 | + | 93722502 | 93722752 | exonic | NM_182896 | ARL13B | CCTAGGATATCGGGAAAGCCTATATTGGTAATACCCTGAAGATGTAGCTCCTACTGTTGG |
| ASCRP3005355 | 0.159234655 | 0.909237851 | 1.0606577 | down | hsa_circRNA_068147 | hsa_circ_0068147 | circBase | chr3 | - | 182804480 | 182810333 | exonic | NM_020166 | MCCC1 | CCTATCTATGGAGAAAATCATTCAAGTGGCCAAGACCTCTGCTGCACAGGAAGAAACATT |
| ASCRP3005378 | 0.270977105 | 0.909237851 | 1.0621749 | down | hsa_circRNA_007120 | hsa_circ_0007120 | circBase | chr1 | + | 2234416 | 2236024 | exonic | NM_003036 | SKI | CCTCCACCAGGTCTCCTCTGAGCCTCCGGCCTCCATAAGACCCAAAACAGATGACACCTC |
| ASCRP3005381 | 0.22905044 | 0.909237851 | 1.0614999 | down | hsa_circRNA_104070 | hsa_circ_0075723 | circBase | chr6 | - | 17648037 | 17649531 | exonic | NM_005124 | NUP153 | CCTCCATCATCTGAAATGGAAGTTCCAGTATTACCGAAAATCTCTCTACCGATCACCAGT |
| ASCRP3005382 | 0.152055349 | 0.909237851 | 1.0605622 | down | hsa_circRNA_007711 | hsa_circ_0007711 | circBase | chr10 | + | 46248035 | 46248685 | exonic | NM_015262 | FAM21C | CCTCCCAGGATCGGCAAGCTGGAGCCTCTGTTAAGGAGGATGAAGAGGATAACTTATTCG |
| ASCRP3005383 | 0.111930343 | 0.909237851 | 1.1278991 | down | hsa_circRNA_406192 | | 25070500 | chr22 | - | 36681397 | 36681703 | intronic | ENST00000216181 | MYH9 | CCTCCCCAAGGTGGGTGCCAGGACTTCTCCCACTCTGGGCTCTGGCTGGAGCCACCACGG |
| ASCRP3005384 | 0.278984496 | 0.909237851 | 1.0557611 | down | hsa_circRNA_000865 | hsa_circ_0000706 | circBase | chr16 | - | 66764014 | 66766408 | intronic | ENST00000379482 | DYNC1LI2 | CCTCCCGTGAGAAAGTGAATTGATTTTTCTTGAGCAGATTGATTAGATGGAGCTGCCTTG |
| ASCRP3005385 | 0.918933746 | 0.98346125 | 1.015472 | down | hsa_circRNA_406918 | | 25070500 | chr7 | - | 23381682 | 23391205 | exonic | NM_006547 | IGF2BP3 | CCTCCCTACCCGCAGTTTGAGAGCACTAGACAAACTGAATGGATTTCAGTTAGAGAATTT |
| ASCRP3005387 | 0.350861944 | 0.91119147 | 1.0386341 | down | hsa_circRNA_102417 | hsa_circ_0048607 | circBase | chr19 | + | 4408899 | 4409756 | exonic | NM_005483 | CHAF1A | CCTCCGCAGACCCGTCTGCCGTTTAAGCGCCTGAATCTTGTCCCAAAGGGGAAAGCCGAT |
| ASCRP3005393 | 0.274862474 | 0.909237851 | 1.0344423 | down | hsa_circRNA_101371 | hsa_circ_0032261 | circBase | chr14 | + | 67736417 | 67770316 | exonic | NM_022474 | MPP5 | CCTCCTGCCAAGGAAACAGTAAATAAGAAAGCCTTGAGTTTTGTGAAAAACCGGAGAAGA |
| ASCRP3005399 | 0.453279058 | 0.91119147 | 1.0219997 | down | hsa_circRNA_405460 | | 25070500 | chr16 | + | 28592375 | 28597041 | exonic | NM_138414 | CCDC101 | CCTCTACACAACCGCCAAGGCCGATGCAGAGGCTGAGTGCAAGTGCCCCTGTAGACAATG |
| ASCRP3005405 | 0.05765491 | 0.909237851 | 1.0812404 | down | hsa_circRNA_092512 | hsa_circ_0001201 | circBase | chr21 | + | 46306732 | 46308705 | antisense | NM_000211 | ITGB2 | CCTCTGAGTCCCTCTCCTTGCAGATGACCAGCAGGAGAATGCCGATCAGCACGATGCCTG |
| ASCRP3005407 | 0.774906338 | 0.964265101 | 1.0120243 | down | hsa_circRNA_102155 | hsa_circ_0045114 | circBase | chr17 | + | 60598133 | 60613698 | exonic | NM_006852 | TLK2 | CCTCTGCTTCACTTTTGTTTCAGGACCACTTAATAGTGAGTCTTCCAACCAGAGCTTGTG |
| ASCRP3005409 | 0.682880799 | 0.941949564 | 1.0154525 | down | hsa_circRNA_083913 | hsa_circ_0083913 | circBase | chr8 | + | 35406809 | 35425759 | exonic | NM_080872 | UNC5D | CCTCTGTGCGCATAGCCTGAACTGACAATGGCGAAGCCCTTCCCGAATCCATCCCATCAG |
| ASCRP3005413 | 0.030155026 | 0.909237851 | 1.1249044 | down | hsa_circRNA_104092 | hsa_circ_0076041 | circBase | chr6 | - | 34100754 | 34101636 | exonic | NM_000841 | GRM4 | CCTCTTCAAGGTTGGTAGGAGGAGAGGATTGGAGCTGTTTTCTCCTTGATGCCAAGATAC |
| ASCRP3005424 | 0.337738146 | 0.91119147 | 1.0625468 | down | hsa_circRNA_406491 | | 25070500 | chr4 | - | 82088314 | 82126214 | exonic | NM_006259 | PRKG2 | CCTGAAGATAAATTAACCAAGATCATTGACTGCTTGGAAGTGGTCCCTGAGCAAAATGGG |
| ASCRP3005430 | 0.196450461 | 0.909237851 | 1.0445477 | down | hsa_circRNA_103159 | hsa_circ_0062239 | circBase | chr22 | - | 19363153 | 19365589 | exonic | NM_003325 | HIRA | CCTGACCAGCATGACTCCGACAGCTGTGGAAAGGGACTTCTCCACGGCATTCTTTAACAG |
| ASCRP3005431 | 0.818384736 | 0.976440085 | 1.0084187 | down | hsa_circRNA_001765 | hsa_circ_0001765 | circBase | chr7 | + | 142563794 | 142563950 | sense overlapping | NM_004445 | EPHB6 | CCTGACTGAGAGCCGAGTGTTACTGGGGGGTCGAGGGGACCTGCTCTTCAATGTCGTGTG |
| ASCRP3005432 | 0.872695611 | 0.979863126 | 1.0198403 | down | hsa_circRNA_100367 | hsa_circ_0014879 | circBase | chr1 | - | 160206924 | 160231148 | exonic | NM_015726 | DCAF8 | CCTGAGACAAGACCGCCCAGCGTCTCCTTCAGTGAATCTACAGACCTATTTTCTCAGGAG |
| ASCRP3005433 | 0.393121746 | 0.91119147 | 1.0533992 | down | hsa_circRNA_052258 | hsa_circ_0052258 | circBase | chr19 | + | 56146824 | 56156989 | exonic | uc002qln.3 | ZNF581 | CCTGAGACTGATCAAACAATAAACACGTTTCCCACTCTGAGGGAGGTGAGGGTGCTGGAG |
| ASCRP3005435 | 0.633288287 | 0.929544944 | 1.0121008 | down | hsa_circRNA_407343 | | 25070500 | chrX | - | 153227786 | 153228670 | intronic | ENST00000310441 | HCFC1 | CCTGAGCTAGCTTGAATCCAGGGAACGACCTGTGTGCAGTCTACCTTGTCAGGGTGGTAG |
| ASCRP3005443 | 0.208353706 | 0.909237851 | 1.1359098 | down | hsa_circRNA_104929 | hsa_circ_0088807 | circBase | chr9 | + | 131271154 | 131271376 | exonic | NM_001499 | GLE1 | CCTGCAACACCAAATGGAACCAAGGATGTTTTAGAAGAATGTATGTCTCTTCCCAAGCTA |
| ASCRP3005445 | 0.539456786 | 0.919549314 | 1.0227628 | down | hsa_circRNA_102816 | hsa_circ_0004645 | circBase | chr2 | - | 128747137 | 128754065 | exonic | NM_024545 | SAP130 | CCTGCACCTATGGGTACTCAGCAGCCTCAGCCTGAAGGAAAGACTTCAGCCAAGGTGGTG |
| ASCRP3005447 | 0.144559569 | 0.909237851 | 1.0511705 | down | hsa_circRNA_405542 | | 25070500 | chr17 | - | 15341020 | 15343599 | sense overlapping | NR_037924 | TVP23C-CDRT4 | CCTGCAGCCATTCCCCAAGAACGTACAAAAGAATCTGAACCTGTGATGTTAAGAAATCAG |
| ASCRP3005448 | 0.082773838 | 0.909237851 | 1.0807203 | down | hsa_circRNA_000797 | hsa_circ_0000797 | circBase | chr17 | + | 62500850 | 62500949 | antisense | NM_004396 | DDX5 | CCTGCCCTACTTCCTCCAAATCGAGGCAGCTCATCAAGATTCCACTTCTTTTTAACTAAT |
| ASCRP3005450 | 0.649275927 | 0.932467957 | 1.0103686 | down | hsa_circRNA_083369 | hsa_circ_0083369 | circBase | chr8 | + | 12388451 | 12388540 | exonic | NR_040091 | LOC100506990 | CCTGCCCTGATAATCAGAAACTCCTGGAGGGCAGAGGCATGCCTGCCATCTTCATCATTG |
| ASCRP3005453 | 0.649701707 | 0.932467957 | 1.021398 | down | hsa_circRNA_052228 | hsa_circ_0052228 | circBase | chr19 | + | 55805384 | 55805765 | exonic | NM_032430 | BRSK1 | CCTGCTCGTGCCACAGAGACCTAAAGCCCGAGAACCTGCTTTTGGATGAGAAAAACAACA |
| ASCRP3005458 | 0.021929748 | 0.909237851 | 1.1006263 | down | hsa_circRNA_102763 | hsa_circ_0004699 | circBase | chr2 | - | 73215386 | 73228707 | exonic | NM_144579 | SFXN5 | CCTGCTGTAGGTAGTCGGTCTTCTCTTGCCCAACCAGACACTGGCATCCACTGTCTTCTG |
| ASCRP3005472 | 0.172552982 | 0.909237851 | 1.0349366 | down | hsa_circRNA_401312 | | 25242744 | chr14 | + | 100728640 | 100728803 | exonic | NM_003403 | YY1 | CCTGGCATTGACCTCTCAGATCCCAAACAACTGGCAGAATTTGCTAGATGAAAAAAAAGA |
| ASCRP3005474 | 0.545578368 | 0.919549314 | 1.0259231 | down | hsa_circRNA_101228 | hsa_circ_0029642 | circBase | chr13 | - | 21006240 | 21013893 | exonic | NM_015974 | CRYL1 | CCTGGCGGCTAGTGGAGGAATGTGTTCCAGAAGATCTAGAACTGAAGAAGAAGATTTTTG |
| ASCRP3005475 | 0.341879684 | 0.91119147 | 1.06031 | down | hsa_circRNA_400098 | hsa_circ_0092281 | circBase | chr9 | - | 130884818 | 130885018 | intronic | ENST00000277462 | PTGES2 | CCTGGCTTCCACACCTCCCCCCTCTCCCCAGAGGAGACCCCAGCAGGGCTGGCCTTGAAG |
| ASCRP3005477 | 0.266097079 | 0.909237851 | 1.1596947 | down | hsa_circRNA_400629 | | 25242744 | chr10 | - | 101645443 | 101646389 | exonic | NM_015221 | DNMBP | CCTGGGCCTGAAGGAGAGGACAGAGCGGCTTGTCATCTCCCCCTTAAATCAGTTACTGAG |
| ASCRP3005481 | 0.34808664 | 0.91119147 | 1.0255127 | down | hsa_circRNA_090135 | hsa_circ_0090135 | circBase | chrX | + | 24512858 | 24523415 | exonic | NM_005391 | PDK3 | CCTGTAACGTGGCGGATGTGGTGAAAGGGAGAGATAATGCATGTGAGAAAACTTCATATA |
| ASCRP3005483 | 0.456877616 | 0.91119147 | 1.0196512 | down | hsa_circRNA_100195 | hsa_circ_0012144 | circBase | chr1 | - | 44773981 | 44785416 | exonic | NM_024066 | ERI3 | CCTGTGGAGACTGGGACTTAAAAGTCATGAAATCATCGAGTTCCCCATCCTAAAGCTAAA |
| ASCRP3005484 | 0.503526773 | 0.914489113 | 1.0480764 | down | hsa_circRNA_104714 | hsa_circ_0007116 | circBase | chr8 | + | 142195237 | 142200516 | exonic | NM_014957 | DENND3 | CCTGTGGTGCTGGCATCTTGACCCAGCCGAAAAAGTTGAAGATGCTCACCCCAAGTTATG |
| ASCRP3005497 | 0.189531295 | 0.909237851 | 1.0386762 | down | hsa_circRNA_103013 | hsa_circ_0059703 | circBase | chr20 | + | 30147413 | 30149539 | exonic | NM_030789 | HM13 | CCTTACCATCTTCATCATGCACATCTTCAAGCATGCTCAGGGATCTTCATTGCCTTGCTG |
| ASCRP3005500 | 0.981755259 | 0.998148217 | 1.0020808 | down | hsa_circRNA_069059 | hsa_circ_0069059 | circBase | chr4 | - | 5664833 | 5699374 | exonic | NM_147127 | EVC2 | CCTTAGAAGAGACTTGCCCTGTATGATTTGGCCCAAAGTGGAATGCTGTCACTTTAAGAC |
| ASCRP3005507 | 0.317786303 | 0.909237851 | 1.0260997 | down | hsa_circRNA_104605 | hsa_circ_0084140 | circBase | chr8 | - | 42725146 | 42729149 | exonic | NM_030954 | RNF170 | CCTTCCCGGTGGAGACCAACTGTGGACATCTTTTTTGTGAAATGTACATCAAAACATTCA |
| ASCRP3005513 | 0.016067073 | 0.909237851 | 1.0713249 | down | hsa_circRNA_102139 | hsa_circ_0044922 | circBase | chr17 | - | 58267923 | 58292135 | exonic | NM_032582 | USP32 | CCTTCGCTATCAAACATCCCAGGAAAGGGAAATGTACCTTCTCCGAATGCACCTTTAAAG |
| ASCRP3005520 | 0.789463135 | 0.967199246 | 1.0138721 | down | hsa_circRNA_045404 | hsa_circ_0045404 | circBase | chr17 | - | 65147161 | 65186471 | exonic | NM_014877 | HELZ | CCTTGAACCTGATTTTTAGAACAAGGAATCTGTAGGTATGGTGCCCAGTGTACTTCAGCA |
| ASCRP3005527 | 0.046791221 | 0.909237851 | 1.0961527 | down | hsa_circRNA_100783 | hsa_circ_0008887 | circBase | chr11 | + | 33307958 | 33350179 | exonic | NM_005734 | HIPK3 | CCTTGGAGTATGATCAGGTATGGCCTCACAAGTCTTGGTCTACCCACCATATGTTTATCA |
| ASCRP3005529 | 0.363582218 | 0.91119147 | 1.0401229 | down | hsa_circRNA_022517 | hsa_circ_0022517 | circBase | chr11 | + | 62530338 | 62534187 | exonic | NM_002696 | POLR2G | CCTTGGTTCTCATGGAAGTATGGCTTTGTAATTGCTGTCACCACCATTGACAATATTGGT |
| ASCRP3005543 | 0.243085834 | 0.909237851 | 1.0672461 | down | hsa_circRNA_102011 | hsa_circ_0042521 | circBase | chr17 | + | 26512204 | 26512291 | exonic | NM_016231 | NLK | CCTTTGCAGGATGTTGGTCTTTGATCCACCATCTCTTCCTGTACTCTATACCCTGTCTAG |
| ASCRP3005551 | 0.311118425 | 0.909237851 | 1.0176302 | down | hsa_circRNA_000295 | hsa_circ_0000969 | circBase | chr19 | - | 59027203 | 59027598 | intronic | ENST00000354590 | ZBTB45 | CGAAAAGCTGTGGGGAGGGGAAAGCCCCACTCTGTGGAGCAGCAGGCACTGGCTTCTGTG |
| ASCRP3005560 | 0.157763494 | 0.909237851 | 1.0826382 | down | hsa_circRNA_400011 | hsa_circ_0092374 | circBase | chr1 | + | 68151221 | 68151421 | intronic | uc009wbd.2 | GADD45A | CGAACGAGCCCCGCGTGAGTCGGCCTGCGGACTCTTCCGGCCCGAACTTCTCTTACCTAC |
| ASCRP3005564 | 0.94267353 | 0.990347843 | 1.0039113 | down | hsa_circRNA_103451 | hsa_circ_0067063 | circBase | chr3 | - | 123512523 | 123595464 | exonic | NM_053025 | MYLK | CGAAGGGCGGGAAGGATTCTTCAAAATTAACAGAAACCAATTCGGGCCAGCTGAAGAGAA |
| ASCRP3005568 | 0.83255068 | 0.978181218 | 1.0060321 | down | hsa_circRNA_006853 | hsa_circ_0006853 | circBase | chr14 | - | 20811282 | 20811360 | sense overlapping | NR_002312 | RPPH1 | CGAAGTGAGTTCAATGGCTGAGGTGAGGGGCCCGGCGGATGCCTCCTTTGCCGGAGCTTG |
| ASCRP3005569 | 0.835634673 | 0.978181218 | 1.0048865 | down | hsa_circRNA_050900 | hsa_circ_0050900 | circBase | chr19 | + | 39191239 | 39200116 | exonic | NM_004924 | ACTN4 | CGAAGTGGCTGAGAAATACCTCGACATCCCCAAGATGCTGGATGCAGAGGACCTTCACGG |
| ASCRP3005575 | 0.420841608 | 0.91119147 | 1.0176807 | down | hsa_circRNA_103627 | hsa_circ_0069559 | circBase | chr4 | - | 40892380 | 40895428 | exonic | NM_004307 | APBB2 | CGACAATGGCCGTGTTTTGCTGTGCGTTCTCTGGGATGGGTAGAGATGGCAGAAGAGGAC |
| ASCRP3005576 | 0.212625956 | 0.909237851 | 1.045653 | down | hsa_circRNA_101910 | hsa_circ_0040987 | circBase | chr16 | + | 89799889 | 89804313 | exonic | NM_152287 | ZNF276 | CGACATTCGGGAGCCAAGCCTTTGCAGGAGGAGCTTCCCACCATCTACAAGTGTCCTTAC |
| ASCRP3005578 | 0.295019521 | 0.909237851 | 1.0644524 | down | hsa_circRNA_406333 | | 25070500 | chr3 | - | 121354581 | 121356103 | exonic | NM_005335 | HCLS1 | CGACCACAGCTTATAAGAAGACGACGCCCATAGAAGCCGATTACTCTCGTGGCTTTGGTG |
| ASCRP3005583 | 0.894412129 | 0.98039597 | 1.0089885 | down | hsa_circRNA_102746 | hsa_circ_0054971 | circBase | chr2 | + | 65228581 | 65237897 | exonic | NM_003038 | SLC1A4 | CGACGATGGTGCTGGTGTCCTGGATTATGTGAAACCTGTTTCCCTCCAATCTTGTGGTTG |
| ASCRP3005586 | 0.868878024 | 0.979863126 | 1.0039869 | down | hsa_circRNA_104286 | hsa_circ_0079135 | circBase | chr7 | - | 1937835 | 1938026 | exonic | NM_003550 | MAD1L1 | CGACTGCCTCATCTTCAAGAGCTGAAGAAGCAGGTGGAGAGTGCCGAGCTGAAGAACCAG |
| ASCRP3005599 | 0.246350864 | 0.909237851 | 1.0356645 | down | hsa_circRNA_103577 | hsa_circ_0007179 | circBase | chr4 | - | 891820 | 898567 | exonic | NM_005255 | GAK | CGAGCCCTGGTGGAGGAAGAGGGCAGCTGGTGGAATTTTTGAAGAAAATGGAATCTCGAG |
| ASCRP3005600 | 0.971128012 | 0.996425773 | 1.0015555 | down | hsa_circRNA_000944 | hsa_circ_0000944 | circBase | chr19 | + | 47767859 | 47768203 | exonic | NM_015603 | CCDC9 | CGAGCGGCAGGAGTGGGAGGAGCGGCGCAGGCAGAACATTGAGAAGATGAATGAGGAGAT |
| ASCRP3005605 | 0.186190911 | 0.909237851 | 1.039804 | down | hsa_circRNA_092500 | hsa_circ_0001108 | circBase | chr2 | + | 231226328 | 231307814 | sense overlapping | NM_003113 | SP100 | CGAGGGCCTCCGTGATCGTGATCTCATCACAAATAAAATGTTTGAACAATCCAAGACAAA |
| ASCRP3005610 | 0.161096873 | 0.909237851 | 1.0446835 | down | hsa_circRNA_070578 | hsa_circ_0070578 | circBase | chr4 | + | 106816596 | 106816880 | exonic | NM_001033047 | NPNT | CGAGTTCGACGGGAGTAGAAGGGAGCGGGAGGGGGCTCCGGGCGCCGCGCAGCAGACCTG |
| ASCRP3005616 | 0.649076982 | 0.932467957 | 1.0097553 | down | hsa_circRNA_100453 | hsa_circ_0008659 | circBase | chr1 | - | 214564330 | 214571344 | exonic | NM_005401 | PTPN14 | CGATCCTCTCTGGACAATCATGGAAACTGTGTACACCTTGGCATTTTCTTTATGGGGATT |
| ASCRP3005617 | 0.501478288 | 0.914489113 | 1.0142533 | down | hsa_circRNA_406263 | | 25070500 | chr3 | - | 41705097 | 41795980 | exonic | NM_017886 | ULK4 | CGATGACTGAACACAACCCAACTTTCACAAGGGTTTTGTCTCCACAATTATCCGTTTACT |
| ASCRP3005623 | 0.185201988 | 0.909237851 | 1.0955321 | down | hsa_circRNA_101433 | hsa_circ_0033049 | circBase | chr14 | + | 94008811 | 94009042 | exonic | NM_020818 | UNC79 | CGATGTCATGGTCATGTGCCTTCTTCCTAAACCCATGGAATTTGCCAGGGTGAGCCTCTG |
| ASCRP3005644 | 0.072679526 | 0.909237851 | 1.0877165 | down | hsa_circRNA_033191 | hsa_circ_0033191 | circBase | chr14 | - | 100826890 | 100828258 | exonic | NM_004184 | WARS | CGCAGAGGCATCTTCTTCTCACACAGGATGAAATTGATTCTGCAGTAAAGATGTTGGTGT |
| ASCRP3005651 | 0.256856288 | 0.909237851 | 1.044818 | down | hsa_circRNA_102264 | hsa_circ_0046555 | circBase | chr17 | + | 80851422 | 80861354 | exonic | NM_005993 | TBCD | CGCCACGCAAGGGCACTTTCCCTCATGGTATTGATATTTTGACCACAGCTGACTATTTTG |
| ASCRP3005653 | 0.936578877 | 0.987920247 | 1.0041714 | down | hsa_circRNA_401635 | | 25242744 | chr16 | - | 69404385 | 69406258 | exonic | NM_005652 | TERF2 | CGCCGAGCCCTACCTCCTCACGGCTGTCATTATTTGTATCAAAAACAAAGAATTTGAAAA |
| ASCRP3005658 | 0.286665296 | 0.909237851 | 1.0846032 | down | hsa_circRNA_101873 | hsa_circ_0004315 | circBase | chr16 | - | 74491771 | 74493687 | exonic | NM_012201 | GLG1 | CGCGGGCGTCATCTCCAGTCTATGTGCTGAAGAAGCAGCAGCCCAAGAGCAGACAGGTCA |
| ASCRP3005661 | 0.643805259 | 0.929872547 | 1.0193776 | down | hsa_circRNA_103276 | hsa_circ_0064136 | circBase | chr3 | + | 9482139 | 9506356 | exonic | NM_001080517 | SETD5 | CGCTGCAACACTCCTCTACAGTTTGAGAATTCTCCCTCTGAAGCACAGAATTTAGATGAG |
| ASCRP3005662 | 0.093070779 | 0.909237851 | 1.1119922 | down | hsa_circRNA_406584 | | 25070500 | chr5 | - | 2749196 | 2749495 | intronic | ENST00000302057 | IRX2 | CGCTGTCCCCTCGTGAGCGGGCAGCGGCCGGCGCAAGGTCTCGGGGCGCTTTCTTCCCAG |
| ASCRP3005675 | 0.028246824 | 0.909237851 | 1.1035656 | down | hsa_circRNA_102571 | hsa_circ_0008615 | circBase | chr19 | - | 45901262 | 45901597 | exonic | NM_006663 | PPP1R13L | CGGACCCCCTTCTAGGGCGCCCGCTCCGGCCGGCACCATGGACAGCGAGGCATTCCAGAG |
| ASCRP3005680 | 0.707424505 | 0.945024804 | 1.008326 | down | hsa_circRNA_006099 | hsa_circ_0006099 | circBase | chr20 | + | 18278628 | 18287037 | exonic | NM_003434 | ZNF133 | CGGCAACCTGTCCAGATCTACCTTCTGAGATATCATCCTTCTTCAGGGAGATAAGGAAAA |
| ASCRP3005686 | 0.190554079 | 0.909237851 | 1.0561941 | down | hsa_circRNA_405656 | | 25070500 | chr18 | - | 12493070 | 12546903 | exonic | NM_020148 | SPIRE1 | CGGCCTGTATCACCAGAGGAGATTAGACGTAGCAGATTAGGTCATTGAATCTTTGGGAAT |
| ASCRP3005690 | 0.978950588 | 0.997082313 | 1.001084 | down | hsa_circRNA_405688 | | 25070500 | chr18 | - | 46855947 | 46860223 | intronic | ENST00000442713 | DYM | CGGCTGGATGAGTAGTGAACCACTTGCTTGGACCCTCCTATGAAGATATTACATATGAAA |
| ASCRP3005696 | 0.454289319 | 0.91119147 | 1.0353178 | down | hsa_circRNA_101677 | hsa_circ_0008480 | circBase | chr16 | + | 1774557 | 1779579 | exonic | NM_015133 | MAPK8IP3 | CGGGGCGGAGAAATTCATTGAGTTTGAAGATGCTCTGGAACAAGAGAAGAAAGAGCTGCA |
| ASCRP3005698 | 0.567670142 | 0.920978531 | 1.0447703 | down | hsa_circRNA_000122 | hsa_circ_0000122 | circBase | chr1 | + | 145439832 | 145440140 | intronic | NM_001039703 | NBPF10 | CGGGTGTCTGTCTCTGCTCGAATTGACAGAAAAGGATTCTGTGAAGGCTTTTCTTGACCG |
| ASCRP3005705 | 0.312881587 | 0.909237851 | 1.0454913 | down | hsa_circRNA_104953 | hsa_circ_0089310 | circBase | chr9 | + | 135917473 | 135919313 | exonic | NM_012087 | GTF3C5 | CGGTGGACTACTTCTACCGACCAGAGACCCAGCACCGATCTACGCAGACCCCACCAAGAG |
| ASCRP3005707 | 0.028777022 | 0.909237851 | 1.1377459 | down | hsa_circRNA_404445 | | 25070500 | chr1 | - | 19638320 | 19638629 | sense overlapping | NM_003689 | AKR7A2 | CGGTGGCGACTGCAGAGGCGCCGCTGCTATGCTGAGTGCCGCGTCTCGCGTAGTCTCCCG |
| ASCRP3005715 | 0.711659832 | 0.946024376 | 1.0100755 | down | hsa_circRNA_406127 | | 25070500 | chr20 | - | 57416991 | 57417600 | exonic | NR_002785 | GNAS-AS1 | CGTACCGCGCTCTTTGGAAACGAATTGGAAGACCACAAAAGCATCCAACCACAGTCTGCG |
| ASCRP3005717 | 0.16158659 | 0.909237851 | 1.0671604 | down | hsa_circRNA_103226 | hsa_circ_0004795 | circBase | chr22 | - | 38948670 | 38964294 | exonic | NM_007068 | DMC1 | CGTACTGGAAAAACCCAGCTTTCTCATACCCTCTGTGAAGTGATTATTTTTCTGTTGCCC |
| ASCRP3005719 | 0.796995731 | 0.970666572 | 1.0113219 | down | hsa_circRNA_102975 | hsa_circ_0004946 | circBase | chr2 | - | 242541310 | 242542494 | exonic | NM_015963 | THAP4 | CGTAGAGCAGGTTCAACTCCTTCCACCCGGACACGCGCAAGCCGATGCACAGAGAGTGTG |
| ASCRP3005729 | 0.284715556 | 0.909237851 | 1.0420472 | down | hsa_circRNA_402000 | | 25242744 | chr19 | - | 648881 | 650296 | exonic | NM_194460 | RNF126 | CGTCCCCGTCACTGAGGAGCACGTAGGATCATCCAGCAGCTCGTCAACGGCATCATCACG |
| ASCRP3005740 | 0.421618082 | 0.91119147 | 1.0845166 | down | hsa_circRNA_102449 | hsa_circ_0049392 | circBase | chr19 | + | 11226769 | 11227674 | exonic | NM_000527 | LDLR | CGTCTTTGAGCTTCATGTACTGGACTGACTGGGGAACTCCCGCCAAGATCAAGAAAGGGG |
| ASCRP3005748 | 0.888349554 | 0.979863126 | 1.0098112 | down | hsa_circRNA_008349 | hsa_circ_0008349 | circBase | chr12 | + | 1754959 | 1756341 | sense overlapping | NM_030775 | WNT5B | CGTGCTCATCATCTCTGCCCCAGGTGCTGTGTATAAGATGGCAGACGTAGCCTGCAAATG |
| ASCRP3005752 | 0.378632058 | 0.91119147 | 1.0896338 | down | hsa_circRNA_023115 | hsa_circ_0023115 | circBase | chr11 | + | 67351934 | 67352712 | exonic | NM_000852 | GSTP1 | CGTGGAGGACCTCCGCTGCAAATACATCTCCCTCATCTACACCAACTATGCCGCTGCGCG |
| ASCRP3005753 | 0.194109166 | 0.909237851 | 1.0836433 | down | hsa_circRNA_400187 | | 25242744 | chr1 | - | 34204740 | 34209207 | exonic | NM_052896 | CSMD2 | CGTGGTCTGGAACAGCGCTGTGCTGCGGTGTGAAGTCTCCTGCTTCTTCAACTTCACCAG |
| ASCRP3005756 | 0.306353257 | 0.909237851 | 1.0224928 | down | hsa_circRNA_068281 | hsa_circ_0068281 | circBase | chr3 | + | 183894738 | 183901879 | exonic | NM_004068 | AP2M1 | CGTGTGACTTCGTCCAGTTACAAACCCAATAAACTCTGTAGAGTGGAGTCTGTTCTCAGA |
| ASCRP3005772 | 0.179854167 | 0.909237851 | 1.0400037 | down | hsa_circRNA_011849 | hsa_circ_0011849 | circBase | chr1 | - | 40026484 | 40029413 | exonic | NM_003819 | PABPC4 | CGTTTCCCAATAAAGAAAAAAAATCTCCATAAAACTGGCGTTCCCACAGCTGTGCAGAAC |
| ASCRP3005775 | 0.392366428 | 0.91119147 | 1.0714475 | down | hsa_circRNA_101895 | hsa_circ_0007361 | circBase | chr16 | - | 87451065 | 87452507 | exonic | NM_015144 | ZCCHC14 | CGTTTGCACAAGTATTACCCCGTCTTTAAGCAGCTCTCCATGGAGAAGGTCCTGGCAATC |
| ASCRP3005787 | 0.724399485 | 0.949785489 | 1.0148948 | down | hsa_circRNA_102796 | hsa_circ_0055954 | circBase | chr2 | - | 107446521 | 107460490 | exonic | NM_032528 | ST6GAL2 | CTAAAGAGAAGATTCAACCAAACCCACCATCTTCTGGTTTCATTGGTTCATTCTGCCACC |
| ASCRP3005796 | 0.542918636 | 0.919549314 | 1.1022373 | down | hsa_circRNA_002178 | hsa_circ_0000519 | circBase | chr14 | - | 20811436 | 20811534 | sense overlapping | NR_002312 | RPPH1 | CTAACAGGGCTCTCCCTGAGCTTCGGGGAGCTGAGTGCGTCCTGTCACTCCACTCCCATG |
| ASCRP3005797 | 0.79928839 | 0.972258433 | 1.0464647 | down | hsa_circRNA_001846 | hsa_circ_0000520 | circBase | chr14 | - | 20811436 | 20811559 | sense overlapping | NR_002312 | RPPH1 | CTAACAGGGCTCTCCCTGAGCTTCGGGGAGGGAAGCTCATCAGTGGGGCCACGAGCTGAG |
| ASCRP3005798 | 0.779033741 | 0.966001839 | 1.0590971 | down | hsa_circRNA_104238 | hsa_circ_0078383 | circBase | chr6 | + | 155411422 | 155448734 | exonic | NM_012454 | TIAM2 | CTAACCTCCCTCACAGCAGAAACTAGACGTCAGGGCTCTGTGTATGAATGACAAGGATAC |
| ASCRP3005802 | 0.451271812 | 0.91119147 | 1.0345474 | down | hsa_circRNA_002491 | hsa_circ_0002491 | circBase | chr1 | - | 173835665 | 173835934 | exonic | NR_002578 | GAS5 | CTAACTCAAGCCATTGGCACACAGTTGTGTCCCCAAGGAAGGATGAGAATAGCTACTGAA |
| ASCRP3005803 | 0.686045712 | 0.942640375 | 1.0179683 | down | hsa_circRNA_401696 | | 25242744 | chr17 | - | 4139066 | 4145743 | exonic | NM_016376 | ANKFY1 | CTAACTTGTCTTCCACTAAAGAGTTGGACCTGTCAGAGGAGGTGGCCAAGTTGGAGAAGC |
| ASCRP3005806 | 0.526203847 | 0.916971445 | 1.0222195 | down | hsa_circRNA_103666 | hsa_circ_0008128 | circBase | chr4 | - | 77051808 | 77057565 | exonic | NM_017426 | NUP54 | CTAAGCAGCATCAAACCAGATTAGATCATTAGGTGGTCTCTTCAGTCAGCCTACACAAGC |
| ASCRP3005818 | 0.616176033 | 0.929544944 | 1.0179664 | down | hsa_circRNA_022764 | hsa_circ_0022764 | circBase | chr11 | + | 64876751 | 64879180 | sense overlapping | NM_013265 | VPS51 | CTAATAAACATGTGTGGCCTCCTCGGTGAGTTCTGCAGTCAGGGTGTCCGTGAGGGCCTC |
| ASCRP3005821 | 0.368490215 | 0.91119147 | 1.0613224 | down | hsa_circRNA_043626 | hsa_circ_0043626 | circBase | chr17 | - | 39766030 | 39766488 | exonic | NM_005557 | KRT16 | CTAATAAAGCTGACTTTCTGGTTGATGCAACCTTTCCTCCCAGCAAGCATCTGGCCAATC |
| ASCRP3005828 | 0.821410549 | 0.976440085 | 1.0132298 | down | hsa_circRNA_064170 | hsa_circ_0064170 | circBase | chr3 | + | 9834800 | 9848789 | exonic | NM_001198780 | ARPC4 | CTAATTAAAGTCTCTTTTTGCCCCTTTGGGGCTGCATGAGGTCCCGCGATGGTGAGAGAG |
| ASCRP3005835 | 0.595064048 | 0.929544944 | 1.0156895 | down | hsa_circRNA_101461 | hsa_circ_0034072 | circBase | chr15 | + | 22940727 | 22947086 | exonic | NM_014608 | CYFIP1 | CTACAACTACACCAGCGAGGAGAAGTTTGCCCTAGTGGAGATGGACGTGCACATCCTCCG |
| ASCRP3005837 | 0.640543002 | 0.929544944 | 1.0190802 | down | hsa_circRNA_003237 | hsa_circ_0003237 | circBase | chr4 | + | 186168447 | 186209236 | exonic | NM_031953 | SNX25 | CTACAATTAGCAGCTTTCCCCAACTGAAGAGGCACAAAGAGTCCTGTGTATGGAAACTCA |
| ASCRP3005838 | 0.557245172 | 0.920978531 | 1.0305167 | down | hsa_circRNA_104499 | hsa_circ_0082564 | circBase | chr7 | - | 137569740 | 137570248 | exonic | NM_194071 | CREB3L2 | CTACACAGCCTCCGTGGGACTCTCCTTCAGCAACTCCAGAAGCTTCAGACTTTGGTGATG |
| ASCRP3005842 | 0.479511236 | 0.91119147 | 1.030848 | down | hsa_circRNA_103532 | hsa_circ_0006614 | circBase | chr3 | + | 183493702 | 183508768 | exonic | NM_018023 | YEATS2 | CTACACCAAACCCCATCTCTGGGAAAGCCACAGTATCCGATCTCCAGTCTGGCTCAGCTG |
| ASCRP3005844 | 0.746778568 | 0.95565436 | 1.0223219 | down | hsa_circRNA_039190 | hsa_circ_0039190 | circBase | chr16 | + | 32761191 | 32761364 | exonic | uc010vfx.1 | AK300387 | CTACACCCTGTAGATGCCTCTGCCGTGTAGCTGTCCGACGTCTCATAAGCTTTTGTCTGG |
| ASCRP3005851 | 0.128532773 | 0.909237851 | 1.04948 | down | hsa_circRNA_083960 | hsa_circ_0083960 | circBase | chr8 | + | 37976787 | 37978667 | exonic | NM_004674 | ASH2L | CTACAGAGCCTGCTTGTATGAACGGGTTTTGTTAGCCCTACATGATCGAGGGGGAATTGC |
| ASCRP3005862 | 0.101033192 | 0.909237851 | 1.1022911 | down | hsa_circRNA_104405 | hsa_circ_0080638 | circBase | chr7 | + | 74433139 | 74434317 | exonic | uc003ubo.2 | GATSL1 | CTACATCTCCCTGGTGATGGACGTGCAGACGCAGCAGAGTCCAGAGGCCAGTCATCCACC |
| ASCRP3005864 | 0.378441473 | 0.91119147 | 1.0358629 | down | hsa_circRNA_004254 | hsa_circ_0004254 | circBase | chr20 | - | 47881252 | 47882803 | exonic | NM_021035 | ZNFX1 | CTACATGAGTGCAAAACCTATGTGGGTCTAAAAATTGTTCAGGCCCTCCTAACCAACGAG |
| ASCRP3005867 | 0.261199089 | 0.909237851 | 1.1613752 | down | hsa_circRNA_103923 | hsa_circ_0073647 | circBase | chr5 | - | 115837905 | 115840678 | exonic | NM_020796 | SEMA6A | CTACATTGCTGCTAGGGTGAAGTAGAGAAATAAAGTCTCCCCGCTGAACTACTATGAGGT |
| ASCRP3005870 | 0.476843505 | 0.91119147 | 1.0231573 | down | hsa_circRNA_102230 | hsa_circ_0046215 | circBase | chr17 | + | 79652634 | 79655857 | exonic | NM_004712 | HGS | CTACCAGATCATGAAGGTGGAGGACAAGGCGACCAGCCAGCTCCTGTTGGAGACAGATTG |
| ASCRP3005877 | 0.880233426 | 0.979863126 | 1.0257627 | down | hsa_circRNA_001871 | hsa_circ_0001871 | circBase | chr9 | + | 91940462 | 91949645 | exonic | NM_001282689 | SECISBP2 | CTACCCAAAAGAATGTTTACTCAGTGCCTGGCTCCCAGTATCTTTATAACCAACCCAGTT |
| ASCRP3005880 | 0.250999043 | 0.909237851 | 1.0203087 | down | hsa_circRNA_102553 | hsa_circ_0051133 | circBase | chr19 | + | 41239064 | 41239269 | exonic | NM_025194 | ITPKC | CTACCCTGGGCTTCCGGATCGAGGGCATCAAGGACCTATCTGGAAGAGGAGCTAGTGAAG |
| ASCRP3005883 | 0.486873956 | 0.91119147 | 1.0366069 | down | hsa_circRNA_103098 | hsa_circ_0003231 | circBase | chr20 | + | 60737807 | 60738678 | exonic | NM_198935 | SS18L1 | CTACCGGCCCTCCCAGCAAGGGCCGAGCCACGTGTCCATGCAGCAGACGGCGCCTAACAC |
| ASCRP3005889 | 0.303641643 | 0.909237851 | 1.0378687 | down | hsa_circRNA_101139 | hsa_circ_0028173 | circBase | chr12 | + | 110764194 | 110780253 | exonic | NM_001681 | ATP2A2 | CTACCTCATCTCGTCCAACGTCGGGGAAGTTGTCTGGTGAATCTGTCTCTGTCATCAAGC |
| ASCRP3005893 | 0.903302037 | 0.981257955 | 1.0041962 | down | hsa_circRNA_405737 | | 25070500 | chr19 | - | 13064953 | 13065340 | sense overlapping | NM_052850 | GADD45GIP1 | CTACCTGGCAGTACCCCTGAAGAGATCTGCGTCCTCTCAAGGGAGCAGCACATCGCAGAG |
| ASCRP3005895 | 0.536362631 | 0.919549314 | 1.0219312 | down | hsa_circRNA_400097 | hsa_circ_0092319 | circBase | chr9 | + | 130868959 | 130869179 | intronic | ENST00000373064 | SLC25A25 | CTACCTTGTCCCGGAATTCCCAGTGACAGCAGAAACAGGGCAGCCAGGCCCCTGGCAGAC |
| ASCRP3005899 | 0.605126096 | 0.929544944 | 1.0411247 | down | hsa_circRNA_102131 | hsa_circ_0044695 | circBase | chr17 | + | 53024621 | 53027487 | exonic | NM_005486 | TOM1L1 | CTACGAGGTAATGGAGTTTGATCCCTTAGCTCCTGCTGTCACTACAGAGTTTGCCCAAAG |
| ASCRP3005900 | 0.093657547 | 0.909237851 | 1.0881768 | down | hsa_circRNA_101425 | hsa_circ_0000560 | circBase | chr14 | - | 91247081 | 91252672 | exonic | NM_001010854 | TTC7B | CTACGCTACCAAAGATGACATGGCAGAGCTTCTCCTCGGGGAGTCGAAGCTGGAGCAGTA |
| ASCRP3005902 | 0.039831179 | 0.909237851 | 1.0913964 | down | hsa_circRNA_102563 | hsa_circ_0051260 | circBase | chr19 | - | 42509874 | 42510962 | exonic | NM_002088 | GRIK5 | CTACGGCATTGGCATGCCGCTGGGTGGGCCTTCACCTTGATCATCATCTCCTCCTACACG |
| ASCRP3005914 | 0.485886266 | 0.91119147 | 1.0595176 | down | hsa_circRNA_001655 | hsa_circ_0001655 | circBase | chr6 | - | 156468903 | 156489628 | intergenic |  |  | CTACTCTCCCTGGGGCTGGATAAAGAAACTGAGGCTCAATGAACTTGTAACGCTTTAGTG |
| ASCRP3005922 | 0.427860728 | 0.91119147 | 1.0356414 | down | hsa_circRNA_101785 | hsa_circ_0038821 | circBase | chr16 | - | 28181070 | 28181230 | exonic | NM_015171 | XPO6 | CTACTGGACCAGGTGCAGACAGTGCTTGGGCTACTGACAGTTGATCCAGTCCCCTGTGAC |
| ASCRP3005929 | 0.059387525 | 0.909237851 | 1.0721346 | down | hsa_circRNA_407075 | | 25070500 | chr8 | + | 51442736 | 51449368 | exonic | NM_018967 | SNTG1 | CTACTTCATTCGCGCTTCTCTCAGTATGTGCCCGGCACAGATTTGAGTCGGTGCTCCAAG |
| ASCRP3005934 | 0.144867614 | 0.909237851 | 1.0653526 | down | hsa_circRNA_101788 | hsa_circ_0038872 | circBase | chr16 | + | 28898509 | 28899043 | exonic | NM_004320 | ATP2A1 | CTACTTTAAGATTGCCGTGGCCTTGGCTGTGGCTGCCATCCCCGAAGGCGAGTCTGTATC |
| ASCRP3005936 | 0.652874888 | 0.933189368 | 1.036367 | down | hsa_circRNA_007022 | hsa_circ_0007022 | circBase | chr19 | + | 47646750 | 47673180 | exonic | NM_005500 | SAE1 | CTACTTTCTCCTTCAAGGCTGCGGGCCTCTCGGGTGCTTCTTGTCGGCTTGAAAGGACTT |
| ASCRP3005944 | 0.909369133 | 0.983376499 | 1.0105501 | down | hsa_circRNA_103316 | hsa_circ_0001279 | circBase | chr3 | - | 33109721 | 33110462 | exonic | NM_000404 | GLB1 | CTAGAGAAAGAGTCTATTCTTCTCCGCTCCTCCGACCCAGGTATGTGCCCTGGAACTTTC |
| ASCRP3005947 | 0.907037765 | 0.982324123 | 1.0022598 | down | hsa_circRNA_063280 | hsa_circ_0063280 | circBase | chr22 | + | 38463710 | 38466898 | exonic | NM_012407 | PICK1 | CTAGAGGAGCTGGAGCGGACCGCTGAGCTATACAAAGGGGGAGGTGACCATCCACTACAA |
| ASCRP3005949 | 0.858921246 | 0.97947761 | 1.0202029 | down | hsa_circRNA_002232 | hsa_circ_0002232 | circBase | chr10 | + | 89624210 | 89693008 | sense overlapping | NM_000314 | PTEN | CTAGATTTCTATGGGGAAGTAAGGACCAGAGACAAAAAGCCACAGGCTCCCAGACATGAC |
| ASCRP3005950 | 0.552104943 | 0.919549314 | 1.0557394 | down | hsa_circRNA_019664 | hsa_circ_0019664 | circBase | chr10 | - | 103868785 | 103871293 | exonic | NM_003893 | LDB1 | CTAGCACCTTCGCCCTCTCCAGCCAGGTACCTGTTGTTCCTCAAAGTCATTCAAGCTGTA |
| ASCRP3005961 | 0.025094054 | 0.909237851 | 1.0379497 | down | hsa_circRNA_101279 | hsa_circ_0030514 | circBase | chr13 | - | 77798585 | 77807398 | exonic | NM_015057 | MYCBP2 | CTAGGACATGGAGATGTCAACTCCAGGCCCCCTTGTATTTGCTGGTCCTATTTTTATGAA |
| ASCRP3005962 | 0.731088456 | 0.952599775 | 1.0126538 | down | hsa_circRNA_092513 | hsa_circ_0001205 | circBase | chr22 | + | 20099876 | 20100132 | antisense | NM_022727 | TRMT2A | CTAGGATGAGGGGAAGGTCCCAGTTTCTTGTAGGGTGTTATCTGGGGGCCCTGGGGATGG |
| ASCRP3005969 | 0.007416195 | 0.879037174 | 1.0949399 | down | hsa_circRNA_100528 | hsa_circ_0017454 | circBase | chr10 | + | 1130342 | 1132297 | exonic | NM_014023 | WDR37 | CTAGTCAAGTACGCAGGCCACGTGGGCTCAGATTGTCTCCAGCTTTAAGACCACGACATC |
| ASCRP3005970 | 0.23372492 | 0.909237851 | 1.1502527 | down | hsa_circRNA_002572 | hsa_circ_0002572 | circBase | chr1 | + | 66713142 | 66731770 | exonic | NM_002600 | PDE4B | CTAGTCAGCCTCCTGTCTCCAGAGTCAACCCACAAGCTTTGATGTGGAAAATGGCCCTTC |
| ASCRP3005971 | 0.945722539 | 0.990803144 | 1.0023076 | down | hsa_circRNA_009586 | hsa_circ_0009586 | circBase | chr1 | - | 8568685 | 8674745 | exonic | NM_012102 | RERE | CTAGTCATCAGGTCCACAACTCCCAGGCCTGTTGCAGATCTCCAACTCCTGCTTTGTGTG |
| ASCRP3005972 | 0.490943428 | 0.91119147 | 1.0170061 | down | hsa_circRNA_021866 | hsa_circ_0021866 | circBase | chr11 | - | 46567153 | 46567326 | exonic | NM_017749 | AMBRA1 | CTAGTGAGATGGAACGGGTCCGGGTGGCAGTGAAAGCTGGTTCACAGATAGCAACAATGC |
| ASCRP3005974 | 0.768425355 | 0.963602436 | 1.0096311 | down | hsa_circRNA_100403 | hsa_circ_0009064 | circBase | chr1 | - | 176132004 | 176153828 | exonic | NM_022457 | RFWD2 | CTAGTGCAGAAGAAGAAACAACTGGAAGCACCCCATCTGCTTTGATATGATTGAAGAAGC |
| ASCRP3005978 | 0.295129542 | 0.909237851 | 1.0207064 | down | hsa_circRNA_087054 | hsa_circ_0087054 | circBase | chr9 | + | 37305540 | 37305711 | exonic | NM_032226 | ZCCHC7 | CTATACAGATAAAGTTCGTCGCTGCTTCCTGTGCTCCAGGAGAGGACATCTCCTGTATTC |
| ASCRP3006002 | 0.264549118 | 0.909237851 | 1.0402458 | down | hsa_circRNA_101857 | hsa_circ_0040148 | circBase | chr16 | + | 70178323 | 70180131 | exonic | NM_017990 | PDPR | CTATGACTCCAGACCACTTCCCAAGCCTCTTTTGCAAGTTTCTTCATGATCTCTCCAACC |
| ASCRP3006017 | 0.788469803 | 0.967199246 | 1.0094448 | down | hsa_circRNA_100660 | hsa_circ_0019390 | circBase | chr10 | + | 99967857 | 99969656 | exonic | NM_014472 | R3HCC1L | CTATGTTTAACGATGATGGTGACTGCCTGGATCCACGTCTTCTACAAGAGATTGTGGTGG |
| ASCRP3006024 | 0.148874571 | 0.909237851 | 1.0572221 | down | hsa_circRNA_027046 | hsa_circ_0027046 | circBase | chr12 | + | 56975182 | 56982802 | exonic | NM_002898 | RBMS2 | CTATTCTTTCCAGTTCAACAAGTAACAGTGGCCCCATCCGATCCCTTGCTTTGCAAATTT |
| ASCRP3006032 | 0.860117974 | 0.97947761 | 1.0121686 | down | hsa_circRNA_100723 | hsa_circ_0020407 | circBase | chr10 | + | 128768965 | 129202693 | exonic | NM_001380 | DOCK1 | CTATTTTGCTGTTGGCTACTACGGACAAGGGTTCCCCACATTCCTGCGGCTTTTTATAAC |
| ASCRP3006033 | 0.882081974 | 0.979863126 | 1.012815 | down | hsa_circRNA_000186 | hsa_circ_0000186 | circBase | chr1 | + | 222803446 | 222803648 | intronic | ENST00000344507 | MIA3 | CTATTTTGTCAGCGCAGCAGGAGAGCCTGCCCTATAATATGGAAAAAGTCCTAGATAAGG |
| ASCRP3006046 | 0.464387672 | 0.91119147 | 1.0239657 | down | hsa_circRNA_102217 | hsa_circ_0000813 | circBase | chr17 | + | 78865519 | 78867665 | exonic | NM_020761 | RPTOR | CTCAACTCCCTCATCGGAGCTGGTGGTGGCTCTGAGTCATCTTGTGGTTCAGTATGAAAG |
| ASCRP3006058 | 0.103293655 | 0.909237851 | 1.0598316 | down | hsa_circRNA_103382 | hsa_circ_0007083 | circBase | chr3 | + | 50289531 | 50290616 | exonic | NM_002070 | GNAI2 | CTCAAGGGAATACCAGCTCAACGACTCAGCTGCCTAGTGCTGGGGAGTCAGGGAAGAGCA |
| ASCRP3006066 | 0.019742744 | 0.909237851 | 1.0552896 | down | hsa_circRNA_000704 | hsa_circ_0000704 | circBase | chr16 | - | 56419830 | 56423287 | exonic | NM_001144 | AMFR | CTCAATGCAATGCTCCTGTCTTCGTTCCTGGCTAGAACAAGACACCTCCTGTCCAACATG |
| ASCRP3006070 | 0.033338402 | 0.909237851 | 1.104646 | down | hsa_circRNA_000872 | hsa_circ_0000747 | circBase | chr17 | + | 27209637 | 27210251 | antisense | NM_004475 | FLOT2 | CTCACAAGCCACGGCCAGGAGTTCCTTCTCCGTCATGATCTTCACCTGGGCTGCCACCTC |
| ASCRP3006071 | 0.165526032 | 0.909237851 | 1.3501419 | down | hsa_circRNA_400082 | hsa_circ_0092371 | circBase | chr5 | + | 173662 | 173882 | intronic | ENST00000283426 | PLEKHG4B | CTCACACACTCGTGCACTCATGGACAGCTGTCCTTCACATTCAAGCACTCTCCCTTGCAC |
| ASCRP3006086 | 0.665633342 | 0.937594692 | 1.0163539 | down | hsa_circRNA_058883 | hsa_circ_0058883 | circBase | chr2 | - | 239169468 | 239186596 | exonic | NM_022817 | PER2 | CTCACGCCGGAGGAGAGCCCTTCGTTCCAGAGCCCAGCATGAATGGATACGCGGAATTTC |
| ASCRP3006087 | 0.722761947 | 0.949785489 | 1.0167371 | down | hsa_circRNA_101282 | hsa_circ_0030569 | circBase | chr13 | - | 95686858 | 95735544 | exonic | NM_005845 | ABCC4 | CTCACTGAAACAGCAAAACAGACATTGCTACAAGTGGTTGGTGTGGTCTCTGTGGCTGTG |
| ASCRP3006095 | 0.497506619 | 0.913224439 | 1.0260193 | down | hsa_circRNA_054474 | hsa_circ_0054474 | circBase | chr2 | + | 48018065 | 48023202 | exonic | NM_000179 | MSH6 | CTCAGAGCCAGAAGAGGAAGAAGAGATGGAGTTGTGACTTCTCACCAGGAGATTTGGTTT |
| ASCRP3006110 | 0.418865867 | 0.91119147 | 1.0204943 | down | hsa_circRNA_075501 | hsa_circ_0075501 | circBase | chr6 | - | 3284085 | 3298452 | exonic | NM_015482 | SLC22A23 | CTCAGCGTGTTCTTCTGTGCGGAGATCACCCCGACGGTGATAAGGATATTCCCCGAGTCC |
| ASCRP3006116 | 0.945811339 | 0.990803144 | 1.0072136 | down | hsa_circRNA_000629 | hsa_circ_0000775 | circBase | chr17 | - | 43012303 | 43012398 | intronic | ENST00000339151 | KIF18B | CTCAGGCCTCTTCCTTTCTTCCTTTCTCAGATACATCCCGGCCTCAGCTCCCCTGTCCAG |
| ASCRP3006121 | 0.002590313 | 0.870215632 | 1.0754184 | down | hsa_circRNA_103059 | hsa_circ_0005759 | circBase | chr20 | + | 35812582 | 35812776 | exonic | NM_002951 | RPN2 | CTCAGGTGCCAGATGCAAAGGTTCAAGCACTGTCTTCCTGTTGGCCCTGACAATCATAGC |
| ASCRP3006124 | 0.351276648 | 0.91119147 | 1.0508617 | down | hsa_circRNA_015081 | hsa_circ_0015081 | circBase | chr1 | + | 165859440 | 165872516 | exonic | NM_012474 | UCK2 | CTCAGTACATTACGTTCGTCAAGCCTGCCTTTGAGGAATTCTGCTTGCCATCTTCCGTGT |
| ASCRP3006127 | 0.994760179 | 0.999550108 | 1.0002118 | down | hsa_circRNA_104578 | hsa_circ_0083818 | circBase | chr8 | - | 28692808 | 28695293 | exonic | NM_018250 | INTS9 | CTCAGTCTGCCTCCTTGTGGAAGAATAAGGACATTCAGAGACGGAGCTAATAGATCTGTC |
| ASCRP3006132 | 0.098235001 | 0.909237851 | 1.13395 | down | hsa_circRNA_006397 | hsa_circ_0006397 | circBase | chr5 | - | 9379934 | 9437964 | exonic | NM_003966 | SEMA5A | CTCAGTGCCAGAGAACCGAGCATCCAGTCATCTCCTATAAAGTGTTGGTGTGTGAGACTT |
| ASCRP3006144 | 0.253767161 | 0.909237851 | 1.0496845 | down | hsa_circRNA_081723 | hsa_circ_0081723 | circBase | chr7 | - | 102307536 | 102309451 | exonic | NM_032959 | POLR2J2 | CTCATCAGTGAGCTGTCCCTGCTGGAGGAGCGCTTCCGGGATCACCATTAACAAGGACAC |
| ASCRP3006147 | 0.194572692 | 0.909237851 | 1.0396202 | down | hsa_circRNA_101812 | hsa_circ_0000699 | circBase | chr16 | + | 50321822 | 50322261 | exonic | NM_001114 | ADCY7 | CTCATCATCATTGCCTTCAGCCAGGGGAGCTGAGGAACTGCGTGTGGAGTCAGCCCAGTC |
| ASCRP3006160 | 0.069668468 | 0.909237851 | 1.1232432 | down | hsa_circRNA_404787 | | 25070500 | chr10 | - | 90069143 | 90074398 | exonic | uc009xtj.3 | RNLS | CTCATGAGATTAATTTCTGAGTCATCAGAAATTGGGCCTTCCCTCGTGATTCACACCACT |
| ASCRP3006164 | 0.243566094 | 0.909237851 | 1.0345729 | down | hsa_circRNA_100337 | hsa_circ_0008704 | circBase | chr1 | - | 151400298 | 151403317 | exonic | NM_015100 | POGZ | CTCATGGCTCTCAAAGAACCAGCGGACCTGAGTCTTCAATGAAAGCTGGCAATCCTTTGG |
| ASCRP3006165 | 0.061490751 | 0.909237851 | 1.0618845 | down | hsa_circRNA_101644 | hsa_circ_0036751 | circBase | chr15 | - | 90451511 | 90454051 | exonic | NM_182616 | ARPIN | CTCATGTCGTCCTACAGGGAAATGGTGTCCTGCTGGAGGGAGAACTGATCGATGTATCTC |
| ASCRP3006166 | 0.043357905 | 0.909237851 | 1.1047939 | down | hsa_circRNA_100435 | hsa_circ_0016201 | circBase | chr1 | - | 205156545 | 205156934 | exonic | NM_015375 | DSTYK | CTCATGTTTTAGCGGAACTGGAGGTAACGATGCACCATGCTCTCTTACAGGCCAACTGAG |
| ASCRP3006167 | 0.089319618 | 0.909237851 | 1.1048953 | down | hsa_circRNA_101153 | hsa_circ_0028299 | circBase | chr12 | + | 112171726 | 112171872 | exonic | NM_025247 | ACAD10 | CTCATTACCTGCCATCCAGTTTTCCCGTGCTGAGAGGCTCGACAACCTGGTGTTTCATCC |
| ASCRP3006180 | 0.24332313 | 0.909237851 | 1.0880983 | down | hsa_circRNA_103287 | hsa_circ_0007975 | circBase | chr3 | + | 11399891 | 11406208 | exonic | NM_006395 | ATG7 | CTCCAACCTCTCTTGGGCTTGTGCCTCACCAGAATGCCAGAGGATTCAACATGAGCATAC |
| ASCRP3006183 | 0.183047734 | 0.909237851 | 1.1245979 | down | hsa_circRNA_405576 | | 25070500 | chr17 | + | 37866065 | 37872192 | exonic | NM_004448 | ERBB2 | CTCCACACTGCCAACCGGCCAGAGGACGAGTGTGGCCACCCCTGTTCTCCGATGTGTAAG |
| ASCRP3006184 | 0.787169531 | 0.967162564 | 1.0069795 | down | hsa_circRNA_100147 | hsa_circ_0004240 | circBase | chr1 | + | 32694099 | 32694809 | exonic | NM_003757 | EIF3I | CTCCACCAGGATTGGCAAGTTTGAGGCCAGTCTGGAGAGGTGTTGGTGAATGTTAAGGAG |
| ASCRP3006188 | 0.111155619 | 0.909237851 | 1.0363806 | down | hsa_circRNA_001918 | hsa_circ_0001918 | circBase | chrX | - | 47061580 | 47065804 | antisense | NM_003334 | UBA1 | CTCCACGGATGTAGTCGGAGAAGTTGGAGGTGGTACAGATGGGGATGGACTTCTCAGGTG |
| ASCRP3006195 | 0.488113727 | 0.91119147 | 1.0801036 | down | hsa_circRNA_100655 | hsa_circ_0006371 | circBase | chr10 | + | 98708764 | 98711953 | exonic | NM_032440 | LCOR | CTCCAGTACTCAAGGGAACGGACAGTCCCTGGGTCTCCGACCCCAATATTCCCCTAGTGG |
| ASCRP3006199 | 0.731429289 | 0.952599775 | 1.0661309 | down | hsa_circRNA_008554 | hsa_circ_0008554 | circBase | chr19 | - | 11289020 | 11289396 | exonic | NM_015493 | KANK2 | CTCCAGTTCGTGGGGGTCAACGGCGGGCCTCCCAGAAGTTCCTGCCGAATCGTCTTCGTC |
| ASCRP3006200 | 0.56977764 | 0.921430132 | 1.0662838 | down | hsa_circRNA_101450 | hsa_circ_0033598 | circBase | chr14 | + | 105818714 | 105821514 | exonic | NM_015197 | PACS2 | CTCCATCAGCATGGCTGAGGGCTCCAAACGAATCCTGCGGTCCCATGAGATTGTGCTGCC |
| ASCRP3006206 | 0.202353064 | 0.909237851 | 1.0665502 | down | hsa_circRNA_100674 | hsa_circ_0000257 | circBase | chr10 | + | 103916775 | 103917971 | exonic | NM_004741 | NOLC1 | CTCCCAAACCAGACACAGCAGGATGCCAATGCCTCTTCCCTCTTAGACATCTATAGCTTC |
| ASCRP3006207 | 0.339427617 | 0.91119147 | 1.0336335 | down | hsa_circRNA_075557 | hsa_circ_0075557 | circBase | chr6 | + | 7176887 | 7247454 | exonic | NM_001003698 | RREB1 | CTCCCACACAGGTTGCTCCGACTGTGTGTTCCAGGAGTGGTGGCTCTGAGGTGTGACCCT |
| ASCRP3006208 | 0.348395088 | 0.91119147 | 1.1134315 | down | hsa_circRNA_404959 | | 25070500 | chr12 | + | 7301567 | 7303659 | exonic | NM_014718 | CLSTN3 | CTCCCACAGAAACTCCAGTGCTTCAGCGAAGAGTCCTGCGTCTCCATCCCTGAAGTGGAG |
| ASCRP3006210 | 0.51098952 | 0.916805276 | 1.0679562 | down | hsa_circRNA_101693 | hsa_circ_0007788 | circBase | chr16 | - | 4516153 | 4519466 | exonic | NM_020677 | NMRAL1 | CTCCCACTTCTTGCCCCAGAAAGCCCCAGACGGAAAGAGCTACTTGCTGAGTGCCCAGGG |
| ASCRP3006211 | 0.220749782 | 0.909237851 | 1.041714 | down | hsa_circRNA_101794 | hsa_circ_0038929 | circBase | chr16 | + | 29810948 | 29811369 | exonic | NM_007317 | KIF22 | CTCCCAGAAACTCAGGACTCTCTGGGTGGCTCAGCCCACAGTATCCTTATTGCCAACATT |
| ASCRP3006216 | 0.144341544 | 0.909237851 | 1.1302763 | down | hsa_circRNA_400071 | hsa_circ_0092283 | circBase | chr22 | - | 36681395 | 36681695 | intronic | ENST00000216181 | MYH9 | CTCCCCAAGGTCAGGACTTCTCCCACTCTGGGCTCTGGCTGGAGCCACCACGGCAGAGCT |
| ASCRP3006219 | 0.818648136 | 0.976440085 | 1.0140755 | down | hsa_circRNA_039626 | hsa_circ_0039626 | circBase | chr16 | - | 58562378 | 58594266 | exonic | NM_016284 | CNOT1 | CTCCCCGACCCATTCACTCCTAATCTAAAGGAGTGTTTCTCAGGAGCTATCAGAAACTAT |
| ASCRP3006220 | 0.580857472 | 0.925542365 | 1.0207546 | down | hsa_circRNA_025460 | hsa_circ_0025460 | circBase | chr12 | - | 10865809 | 10868382 | exonic | NM_003651 | YBX3 | CTCCCCGGAATACTGCCATCAAGAAGAATAACCCACGGAAATATCTGCGCAGTGTAGGAG |
| ASCRP3006221 | 0.032840647 | 0.909237851 | 1.0471045 | down | hsa_circRNA_075320 | hsa_circ_0075320 | circBase | chr5 | + | 179249957 | 179260782 | exonic | NM_003900 | SQSTM1 | CTCCCGCCAGATGAGGACGGGGACTTGGTTGCCTTTTCCAGTGACGAGGAATTGACAATG |
| ASCRP3006225 | 0.637500986 | 0.929544944 | 1.0210047 | down | hsa_circRNA_063603 | hsa_circ_0063603 | circBase | chr22 | + | 42204878 | 42204997 | exonic | NM_024821 | CCDC134 | CTCCCTGGACCCAAGCCTGGAGATCTCTCAAGAGGTTTGGATATGGACCTTCTTCAATTC |
| ASCRP3006226 | 0.5171595 | 0.916971445 | 1.0305103 | down | hsa_circRNA_405510 | | 25070500 | chr16 | - | 89458787 | 89459087 | intronic | ENST00000301030 | ANKRD11 | CTCCCTTATACAGGCTGACATCCGAAGTGCATTACAGATGCTGAGTTGATCGTTAACTTC |
| ASCRP3006227 | 0.844031242 | 0.978270321 | 1.0088635 | down | hsa_circRNA_033628 | hsa_circ_0033628 | circBase | chr14 | + | 105953535 | 105955124 | exonic | NM_001311 | CRIP1 | CTCCCTTGTTGCCCCTAATGCTCTCAGTAAACCTGAACACTTGGAATCTCGCGCCTGCAG |
| ASCRP3006228 | 0.767344314 | 0.962763881 | 1.0149646 | down | hsa_circRNA_007586 | hsa_circ_0007586 | circBase | chr16 | - | 4029116 | 4033441 | exonic | NM_001116 | ADCY9 | CTCCGAATATGAGACCAACATACACGTCATAAAGAACTCCCCCGTGAAGACGTTTGCTAG |
| ASCRP3006230 | 0.272340052 | 0.909237851 | 1.089516 | down | hsa_circRNA_062980 | hsa_circ_0062980 | circBase | chr22 | + | 32871223 | 32881196 | exonic | NM_001033024 | FBXO7 | CTCCGCTACTCTCACCTGTGTGCCTTTGGGAAACCTGATTGTTGTAAATGGGCCAAGTGC |
| ASCRP3006231 | 0.18561361 | 0.909237851 | 1.0881063 | down | hsa_circRNA_006652 | hsa_circ_0006652 | circBase | chr5 | + | 134508189 | 134509862 | sense overlapping | NM_001277348 | C5orf66 | CTCCGGGCCAGGGCTGCTGATCTTCTCCTGAAGTCTCAGAACAGATGGCTGCCAAGTAGG |
| ASCRP3006235 | 0.685765517 | 0.942626653 | 1.0159632 | down | hsa_circRNA_102415 | hsa_circ_0007376 | circBase | chr19 | - | 4101016 | 4101278 | exonic | NM_030662 | MAP2K2 | CTCCTACATGGCTGTTCTCCGGGGCTTGGCGTACCTCCGAGAGAAGCACCAGATCATGCA |
| ASCRP3006236 | 0.731055691 | 0.952599775 | 1.0185154 | down | hsa_circRNA_103270 | hsa_circ_0064018 | circBase | chr22 | - | 51214199 | 51216409 | exonic | NM_007081 | RABL2B | CTCCTACTACCACAAGGCCCACGCCTGCATCATGACTCATGGAGAGATTTCTCATGGATG |
| ASCRP3006240 | 0.306117562 | 0.909237851 | 1.1167869 | down | hsa_circRNA_000890 | hsa_circ_0000890 | circBase | chr19 | + | 10798306 | 10799354 | sense overlapping | NM_012218 | ILF3 | CTCCTCATACCAAGGCAAACAAGGCAGCTACTCCTACTCGAACTCCTACAACTCTCCCGG |
| ASCRP3006249 | 0.401171763 | 0.91119147 | 1.0652536 | down | hsa_circRNA_000710 | hsa_circ_0000710 | circBase | chr16 | - | 67973788 | 67974381 | sense overlapping | NM_000229 | LCAT | CTCCTGAATAAAGACCTTCCTTTGCTACCGTGACAACCAGGGCATCCCCATCATGTCCAG |
| ASCRP3006250 | 0.715358372 | 0.948442621 | 1.0094091 | down | hsa_circRNA_104513 | hsa_circ_0007518 | circBase | chr7 | + | 140402665 | 140404763 | exonic | NM_004546 | NDUFB2 | CTCCTGATGATGAAGACTGAAGGTGTAGACTCAGCCTCACTCTGTACAATGCCGGTGGTG |
| ASCRP3006251 | 0.708361309 | 0.945024804 | 1.0140188 | down | hsa_circRNA_103250 | hsa_circ_0002657 | circBase | chr22 | + | 46085591 | 46125470 | exonic | NM_013236 | ATXN10 | CTCCTGATGATGAGAGAAACAGCACCCAGGACTATCTTCCAAAGAGTTCTGGATATCCTA |
| ASCRP3006252 | 0.84897169 | 0.978900086 | 1.0065798 | down | hsa_circRNA_037205 | hsa_circ_0037205 | circBase | chr16 | + | 630857 | 632309 | exonic | NM_004204 | PIGQ | CTCCTGATGCAGCTCCGGCTCCTGGTGGTCGCCGTGCAGGGCCTGATCCATCTGCTCGTG |
| ASCRP3006253 | 0.885349022 | 0.979863126 | 1.0040728 | down | hsa_circRNA_102972 | hsa_circ_0006553 | circBase | chr2 | + | 242346951 | 242350545 | exonic | NM_014808 | FARP2 | CTCCTGCAGTGGCCAAAGAATGTGGTGCTTCGCCTAGCTGTAAAATTTTTTCCACCTGAT |
| ASCRP3006255 | 0.395953106 | 0.91119147 | 1.9987018 | down | hsa_circRNA_058230 | hsa_circ_0058230 | circBase | chr2 | + | 219204505 | 219206867 | exonic | NM_015488 | PNKD | CTCCTGCCTCTTCTCAGGGGACCTGCTCTTCCTCTCTGGCTGTGGTACAGCCTGTACACC |
| ASCRP3006258 | 0.988488718 | 0.998148217 | 1.0006976 | down | hsa_circRNA_100185 | hsa_circ_0004628 | circBase | chr1 | - | 41578954 | 41582682 | exonic | NM_012236 | SCMH1 | CTCCTGGCACCAAAGGATTTCGGCTGAATGCGTCTTCTTGGCCCATGTTCCTTTTGAAGA |
| ASCRP3006259 | 0.870517206 | 0.979863126 | 1.0113415 | down | hsa_circRNA_004299 | hsa_circ_0004299 | circBase | chr7 | + | 98985661 | 98987635 | exonic | NM_005720 | ARPC1B | CTCCTGTGACTTCAAGTGTCGGCATCGACTGGGCCCCCGAGAGTAACCGTATTGTGACCT |
| ASCRP3006260 | 0.968514065 | 0.995691755 | 1.0016801 | down | hsa_circRNA_406900 | | 25070500 | chr7 | + | 2477438 | 2483381 | sense overlapping | NR_108066 | LOC101927181 | CTCCTGTTGGGTCTCTGGCGGACAGTCAGAGGGTGTAGGAGTTGTGTTCCAAGTTTGTTC |
| ASCRP3006266 | 0.578749981 | 0.925149861 | 1.0187651 | down | hsa_circRNA_100980 | hsa_circ_0024731 | circBase | chr11 | + | 123489400 | 123489932 | exonic | NM_020716 | GRAMD1B | CTCCTTGACCAGTCTGGTGCTGCTGGTCATCCTTAACATGATGCTCTTCTACAAACTCTG |
| ASCRP3006270 | 0.620261239 | 0.929544944 | 1.0170648 | down | hsa_circRNA_400325 | | 25242744 | chr1 | - | 153784206 | 153785928 | exonic | NM_020699 | GATAD2B | CTCCTTGGTATGCCAGGCAAAAGCTGTGCCTCACTTCTGCGGGTTGAACCCTTTGTATGT |
| ASCRP3006272 | 0.458102275 | 0.91119147 | 1.0347323 | down | hsa_circRNA_101365 | hsa_circ_0032202 | circBase | chr14 | + | 64908771 | 64916340 | exonic | NM_005956 | MTHFD1 | CTCCTTTATGACCTCAAGGGCACTCCAGTGTTTGTCCATGCTGGCCCGTTTGCCAACATC |
| ASCRP3006273 | 0.264689687 | 0.909237851 | 1.0350313 | down | hsa_circRNA_000113 | hsa_circ_0000292 | circBase | chr11 | - | 44961348 | 44961546 | intronic | ENST00000308212 | TP53I11 | CTCCTTTTTGTGGATGAGGAACTAAAGCCAGGGGGCGTAGGAGGGTAGGTTGGCCTCCTG |
| ASCRP3006274 | 0.751606423 | 0.957450341 | 1.0303146 | down | hsa_circRNA_104951 | hsa_circ_0089254 | circBase | chr9 | - | 134518625 | 134526336 | exonic | NM_005312 | RAPGEF1 | CTCGAATTCAGCACAGACTCTCAGCGTTCTCATCTCTCTTCCTTCACCATGAAGCTGATG |
| ASCRP3006275 | 0.190367049 | 0.909237851 | 1.0625717 | down | hsa_circRNA_102836 | hsa_circ_0056790 | circBase | chr2 | + | 159857591 | 159954346 | exonic | NM_033394 | TANC1 | CTCGACAGTCTCACTTGGTGCAATCAAGAGTGAACAAAAAATCCCCAGGAGCTGACTGCT |
| ASCRP3006279 | 0.367066024 | 0.91119147 | 1.1055319 | down | hsa_circRNA_402033 | | 25242744 | chr19 | - | 8995634 | 8997536 | exonic | NM_024690 | MUC16 | CTCGATATTTGGCCCTTCAGCTGCCAGCCCTCTCCTGGTGCTATTCACTCTCAACTTCAC |
| ASCRP3006284 | 0.749862618 | 0.956614862 | 1.0638667 | down | hsa_circRNA_101141 | hsa_circ_0005785 | circBase | chr12 | - | 110819556 | 110834257 | exonic | NM_016238 | ANAPC7 | CTCGCACCTTGTCGCTTAGATTGTTATGAAGTGAGTTATTCTCCCCACCTCAGAAGTACC |
| ASCRP3006287 | 0.425699725 | 0.91119147 | 1.0315402 | down | hsa_circRNA_102409 | hsa_circ_0006392 | circBase | chr19 | + | 3425103 | 3435205 | exonic | NM_005597 | NFIC | CTCGCCCACGAGTAGCAGCCGCAACTGGACGGAGGACATGGAAGGAGATGCAGAGCAAAG |
| ASCRP3006290 | 0.064794875 | 0.909237851 | 1.0699028 | down | hsa_circRNA_406060 | | 25070500 | chr2 | + | 233022758 | 233075115 | sense overlapping | NM_152383 | DIS3L2 | CTCGCTGACGGTCCCTATCCATCTGGTTAAACTTGTAGTCACTGCCTATCAGCCTATCAG |
| ASCRP3006292 | 0.397276845 | 0.91119147 | 1.0593816 | down | hsa_circRNA_000049 | hsa_circ_0000049 | circBase | chr1 | + | 33429698 | 33429839 | antisense | NM_153341 | RNF19B | CTCGCTTATCTCCAGGCGCAGGTAGCGGCGCAGCATGAACTCCTCGTACTTGTGCATAAG |
| ASCRP3006294 | 0.244332908 | 0.909237851 | 1.0737382 | down | hsa_circRNA_000898 | hsa_circ_0000898 | circBase | chr19 | + | 13213456 | 13213544 | antisense | NM_005583 | LYL1 | CTCGGAAGGCCAGGATAGCCGAGGTCTCCGCGTAGGGTCCGGCCTCAATGCTCCAGGCCC |
| ASCRP3006297 | 0.902193057 | 0.981257955 | 1.0101696 | down | hsa_circRNA_400073 | hsa_circ_0092361 | circBase | chr3 | + | 42569005 | 42569345 | intronic | ENST00000325123 | VIPR1 | CTCGTATTTCAGGATGGGTCACAGGCCTCAAAGCAAACCTGGCAAGTGTCCCTCCCACAG |
| ASCRP3006301 | 0.279689043 | 0.909237851 | 1.036353 | down | hsa_circRNA_015164 | hsa_circ_0015164 | circBase | chr1 | - | 169446392 | 169446995 | exonic | NM_006996 | SLC19A2 | CTCTAAATATGGAGGAGCCTCCCGTGGAGGAACCGGTCTTCAATGAAATTTATCCAGTAT |
| ASCRP3006305 | 0.120743085 | 0.909237851 | 1.0976347 | down | hsa_circRNA_402019 | | 25242744 | chr19 | + | 5143977 | 5144913 | exonic | NM_015015 | KDM4B | CTCTACAAGGCCAAGTTCATCTCCTCCGTCACCAGCCACATCTACCAGAAATGCGTGTAC |
| ASCRP3006311 | 0.439958371 | 0.91119147 | 1.0583726 | down | hsa_circRNA_014306 | hsa_circ_0014306 | circBase | chr1 | + | 153734045 | 153737539 | exonic | NM_023015 | INTS3 | CTCTACTACCTGAGGGCCAGACACCTAGCTCCCCTGTTTGACAACCCTAAGTTGGATAAG |
| ASCRP3006325 | 0.444304574 | 0.91119147 | 1.0209692 | down | hsa_circRNA_101435 | hsa_circ_0033144 | circBase | chr14 | - | 99723807 | 99724176 | exonic | NM_022898 | BCL11B | CTCTCACCCACGAAAGGCATCTGTCCCAAGCAGGAGAACATTGCAGCAGAGGCTGACCAT |
| ASCRP3006328 | 0.403661818 | 0.91119147 | 1.0198082 | down | hsa_circRNA_017819 | hsa_circ_0017819 | circBase | chr10 | - | 13705452 | 13712528 | exonic | NM_018027 | FRMD4A | CTCTCAGATGCCCTTGTTCTTGAGGATGGAGCTCACGGGCAAGCTGCCAGTAGAATATCC |
| ASCRP3006331 | 0.548356872 | 0.919549314 | 1.0208449 | down | hsa_circRNA_400743 | | 25242744 | chr11 | + | 43852525 | 43861614 | exonic | NM_016142 | HSD17B12 | CTCTCAGTGCCTCCATGAGGAGTATAGGAGCAAGGGCGTCTTTGTGCAGATGACACAATT |
| ASCRP3006333 | 0.505547857 | 0.915072535 | 1.0374278 | down | hsa_circRNA_101744 | hsa_circ_0005699 | circBase | chr16 | + | 19627435 | 19663412 | exonic | NM_020314 | C16orf62 | CTCTCCCAAGGTGGGAATGGAAGTGGCCCCACATCTCAAAGAAACCCTAAATAAGAACTT |
| ASCRP3006340 | 0.241896699 | 0.909237851 | 1.1313967 | down | hsa_circRNA_404714 | | 25070500 | chr10 | - | 5998341 | 6002530 | exonic | NM_002189 | IL15RA | CTCTCCTGGCATGCTACCTCAAGTCAAGAGCCCGCAGCTTCATCTCCCAGCTCAAACAAC |
| ASCRP3006341 | 0.447225842 | 0.91119147 | 1.0919774 | down | hsa_circRNA_105027 | hsa_circ_0006789 | circBase | chrX | + | 118544152 | 118544325 | exonic | NM_145305 | SLC25A43 | CTCTCCTTTCCCTTTGAGACCGTGAAGAGAAAGATGCAGGTGCTCTCCCGTTCTCTGCTG |
| ASCRP3006346 | 0.008480673 | 0.882339103 | 1.0642011 | down | hsa_circRNA_104105 | hsa_circ_0076260 | circBase | chr6 | + | 38702249 | 38718365 | exonic | NM_001206927 | DNAH8 | CTCTCTATAACCATGACCTAAATGTGGTCGAACTATTGCTGGAGCAACTAAAGGGGCAAA |
| ASCRP3006352 | 0.560893185 | 0.920978531 | 1.0187675 | down | hsa_circRNA_101437 | hsa_circ_0033163 | circBase | chr14 | + | 100384118 | 100387214 | exonic | NM_004434 | EML1 | CTCTCTGTAATGCGATACTCACCAGGATCCAGCTCAGTCTTCTGGTTTTCATCCTTCAGG |
| ASCRP3006362 | 0.597409008 | 0.929544944 | 1.0245423 | down | hsa_circRNA_004546 | hsa_circ_0004546 | circBase | chr19 | - | 45871887 | 45872405 | exonic | NM_000400 | ERCC2 | CTCTGAGCTCCCGCAAAAACTTGTGTATTCACCCTGAGGGTCATGGAGTCCTGGAGATGC |
| ASCRP3006364 | 0.203541664 | 0.909237851 | 1.1985704 | down | hsa_circRNA_102173 | hsa_circ_0002056 | circBase | chr17 | - | 62782684 | 62793527 | exonic | NR_024386 | PLEKHM1P | CTCTGAGTTTGACACCACAATCAGGCTGCTCCCGGCAGATCGGCTTCTCCTTTGTACGAC |
| ASCRP3006365 | 0.154400357 | 0.909237851 | 1.0507877 | down | hsa_circRNA_089651 | hsa_circ_0089651 | circBase | chr9 | - | 140086068 | 140086626 | exonic | NM_001128228 | TPRN | CTCTGATGATGGACTCAATAAACAGCACTGGACAAGGCTCTCACACCCGCCAGTCAGAAT |
| ASCRP3006368 | 0.783220575 | 0.966660917 | 1.0130402 | down | hsa_circRNA_103378 | hsa_circ_0003795 | circBase | chr3 | + | 50142509 | 50143142 | exonic | NM_005778 | RBM5 | CTCTGCAATGACTCTGAACAGGAAGTGCCTCCTGGAACCACAGAGTCGGTTCAGTCTGTG |
| ASCRP3006375 | 0.61971976 | 0.929544944 | 1.0500778 | down | hsa_circRNA_103468 | hsa_circ_0067260 | circBase | chr3 | + | 128973510 | 128973920 | exonic | NM_016128 | COPG1 | CTCTGCCCTCGTGTCTTCCTTGCCTAACAAAAGACATGACTGGGAAAGAAGACAACTACC |
| ASCRP3006378 | 0.451511781 | 0.91119147 | 1.0184462 | down | hsa_circRNA_104296 | hsa_circ_0079284 | circBase | chr7 | - | 5680784 | 5692141 | exonic | NM_207111 | RNF216 | CTCTGGACCGATCCCACTGAAACCTGTAGGAAGTGTCAGGGACTCTGGAAAGAACATAAT |
| ASCRP3006385 | 0.873540975 | 0.979863126 | 1.0208002 | down | hsa_circRNA_406567 | | 25070500 | chr4 | - | 177630122 | 177632804 | sense overlapping | NM_005429 | VEGFC | CTCTGTGATCAGTTATTTGAAATTACAGTGCCTCTCTCTCAAGGCCCCAAACCAGTAACA |
| ASCRP3006396 | 0.115576327 | 0.909237851 | 1.2188646 | down | hsa_circRNA_104475 | hsa_circ_0082182 | circBase | chr7 | + | 128317617 | 128323309 | exonic | uc003vnl.3 | FAM71F2 | CTCTTATAATGCTCCTTTTCCACTGTGAGCCAAGTGACCTTGGGAGAGGCTTCTCCCCCT |
| ASCRP3006402 | 0.879766249 | 0.979863126 | 1.0218856 | down | hsa_circRNA_404853 | | 25070500 | chr11 | - | 16133348 | 16208501 | exonic | NM_017508 | SOX6 | CTCTTCCCCCCTGGAATAACATACAAACCAGGTACACCTGAGAGCCTGGCAGAAAAAGAA |
| ASCRP3006406 | 0.708016477 | 0.945024804 | 1.0198678 | down | hsa_circRNA_044353 | hsa_circ_0044353 | circBase | chr17 | + | 46189392 | 46196548 | exonic | NM_013323 | SNX11 | CTCTTCTCACCTGGCTAAAGGAGACCAGCCTAAGAGATGTTTCCTTCCAATGGGCTTTTG |
| ASCRP3006408 | 0.557634143 | 0.920978531 | 1.028655 | down | hsa_circRNA_406455 | | 25070500 | chr4 | - | 22399613 | 22404422 | intronic | ENST00000504617 | GPR125 | CTCTTCTTCATGTCGCTGACTCCCACTTACCTTGCAGGATTTGACGGGATCTGAACTATA |
| ASCRP3006415 | 0.498911472 | 0.913499879 | 1.0721875 | down | hsa_circRNA_000046 | hsa_circ_0000059 | circBase | chr1 | + | 40506473 | 40506637 | intronic | ENST00000340450 | CAP1 | CTCTTGGGCGCGGTCTCTTGAGGTGAGGGTGTGCAGCTTGGTAGGGATTGGGGTCCCTTC |
| ASCRP3006416 | 0.12614932 | 0.909237851 | 1.0494412 | down | hsa_circRNA_104230 | hsa_circ_0078346 | circBase | chr6 | - | 154743639 | 154744117 | exonic | NM_173515 | CNKSR3 | CTCTTGTACAGTCTCCTGCAGACAGATCTCAGAAGATTCATGCTGGTGACGAAGTCATTC |
| ASCRP3006423 | 0.170183336 | 0.909237851 | 1.0436093 | down | hsa_circRNA_104598 | hsa_circ_0002754 | circBase | chr8 | - | 41905895 | 41907225 | exonic | NM_001099412 | KAT6A | CTCTTTCCTGTTTACCTCCAGTGTCCCTTCTTCCACATGAAAAGGATAAGGATTCTTTCT |
| ASCRP3006425 | 0.425018691 | 0.91119147 | 1.0296939 | down | hsa_circRNA_102423 | hsa_circ_0006461 | circBase | chr19 | + | 5039846 | 5041262 | exonic | NM_015015 | KDM4B | CTCTTTGTATGATGACATCATCCCCCCGAAGGAGTGGAAGCCGCGGCAGACGTATGATGA |
| ASCRP3006429 | 0.06163912 | 0.909237851 | 1.0952057 | down | hsa_circRNA_092361 | hsa_circ_0000010 | circBase | chr1 | - | 2521314 | 2521523 | antisense | NM_152371 | FAM213B | CTGAAAACCCAACTCTCCTGAGTGGCCAAGAAGCTGGACACACCTAATTAGACTAAGACC |
| ASCRP3006430 | 0.665205152 | 0.937594692 | 1.0237813 | down | hsa_circRNA_100051 | hsa_circ_0009792 | circBase | chr1 | - | 11181302 | 11182183 | exonic | NM_004958 | MTOR | CTGAAAAGCCCCAGCTCCGAGCATCCAGAGATACGCTGTCATCCCTTTATCGACCAACTC |
| ASCRP3006432 | 0.713511173 | 0.947117927 | 1.0094192 | down | hsa_circRNA_101627 | hsa_circ_0005053 | circBase | chr15 | + | 85607600 | 85626875 | exonic | NM_002605 | PDE8A | CTGAAACTCAGGTAGCAGTAGCTGATGTGCAGTTTGGCCCCATGAGATTTCATCAAGATC |
| ASCRP3006435 | 0.597914723 | 0.929544944 | 1.020478 | down | hsa_circRNA_101860 | hsa_circ_0003438 | circBase | chr16 | - | 70294946 | 70296427 | exonic | NM_001605 | AARS | CTGAAAGTGGGGGATCAGGTCTGGCTGTTTATTGATGAGTATTTGAGAACACAGTGGCTA |
| ASCRP3006440 | 0.314852789 | 0.909237851 | 1.1088292 | down | hsa_circRNA_100533 | hsa_circ_0006608 | circBase | chr10 | + | 3160973 | 3162236 | exonic | NM_002627 | PFKP | CTGAACACTATCACCGACCGTTCTCCCGGGGAAGTACTTGGAAGAGATCGCCACACAGAT |
| ASCRP3006444 | 0.264569078 | 0.909237851 | 1.0383144 | down | hsa_circRNA_104495 | hsa_circ_0001973 | circBase | chr7 | - | 131193709 | 131196192 | exonic | NM_005397 | PODXL | CTGAACCTCACAGGAAACACCCTCTGTCAACCCAGACTACTACGGACTCATCTAACAAAA |
| ASCRP3006454 | 0.084736101 | 0.909237851 | 1.0993757 | down | hsa_circRNA_032377 | hsa_circ_0032377 | circBase | chr14 | - | 71209067 | 71209308 | exonic | NM_033141 | MAP3K9 | CTGAAGCTCAAGGATGGCAACCGCATCAGCCTCCCTTCTGGAACTTCGCACCTGGGAGGA |
| ASCRP3006463 | 0.083760683 | 0.909237851 | 1.0648887 | down | hsa_circRNA_407054 | | 25070500 | chr8 | - | 30412464 | 30435989 | sense overlapping | ENST00000355904 | GTF2E2 | CTGAAGTACCAACCCTTCTGCAGTGGAAGCAGCTCCAGTCTCTGATTTCCATGGGTCACG |
| ASCRP3006464 | 0.543603907 | 0.919549314 | 1.0588675 | down | hsa_circRNA_085993 | hsa_circ_0085993 | circBase | chr8 | - | 145317005 | 145318513 | exonic | uc003zbm.3 | BOP1 | CTGAAGTCAACAGAGCCTTTACCCTGTGGCTCTGACGACGGCTCCCTGCGGCTCTGGGAG |
| ASCRP3006472 | 0.443913514 | 0.91119147 | 1.0584986 | down | hsa_circRNA_100034 | hsa_circ_0006837 | circBase | chr1 | - | 8555122 | 8617582 | exonic | NM_012102 | RERE | CTGAATGCACTGAACACAAGTAAGAGGGACCATCTCCTCATGAACGTCAAATGGTACTAC |
| ASCRP3006479 | 0.916621843 | 0.98346125 | 1.0054056 | down | hsa_circRNA_102434 | hsa_circ_0049138 | circBase | chr19 | + | 9435370 | 9435456 | exonic | NM_032497 | ZNF559 | CTGACAATTCTGTCGTGTCCCGGAACAGCATCTCTGCCTTCCTGTTCACGGTGACCTTCG |
| ASCRP3006480 | 0.284844804 | 0.909237851 | 1.0328924 | down | hsa_circRNA_049192 | hsa_circ_0049192 | circBase | chr19 | - | 10246411 | 10246963 | exonic | NM_001379 | DNMT1 | CTGACACCTACCGGCTCTTCGGCAACATCCTGGACAAGCACCGGCAGCCGGCAAAGCCTG |
| ASCRP3006485 | 0.694270474 | 0.943480789 | 1.0186832 | down | hsa_circRNA_401218 | | 25242744 | chr14 | - | 23530292 | 23530831 | exonic | NM_014977 | ACIN1 | CTGACAGCCAGCTGGATTATCACCGAGGCCTCTTGGTGGACCGTCCCTCTGAAACTAAGA |
| ASCRP3006487 | 0.51528184 | 0.916971445 | 1.0439547 | down | hsa_circRNA_104577 | hsa_circ_0003885 | circBase | chr8 | + | 28570973 | 28575724 | exonic | NM_001440 | EXTL3 | CTGACATTGGCGTCCCCATCATGGAGAGCAAGCCCTGGAGGTTCACTCTTTCAAGAAGTC |
| ASCRP3006489 | 0.708416857 | 0.945024804 | 1.0173583 | down | hsa_circRNA_020235 | hsa_circ_0020235 | circBase | chr10 | - | 123274636 | 123279683 | exonic | NM_001144918 | FGFR2 | CTGACCAAACGTATCCCCCTGCGGAGACAGAGCGATCGCCTCACCGGCCCATCCTCCAAG |
| ASCRP3006493 | 0.129901986 | 0.909237851 | 1.15699 | down | hsa_circRNA_407229 | | 25070500 | chr9 | + | 114541671 | 114541925 | antisense | NM_173521 | C9orf84 | CTGACCTCTCCTAGTTATAAATGCACAGCTCTACCAATCTAACTCATAAGATGATCTCTA |
| ASCRP3006495 | 0.634170774 | 0.929544944 | 1.0218052 | down | hsa_circRNA_050124 | hsa_circ_0050124 | circBase | chr19 | - | 18650180 | 18653782 | exonic | uc010xqi.1 | FKBP8 | CTGACTCCAAGTACTGCTACGGCCCCCAAGGCAGGAATTGTGCGGGCGCGGGGAATTATG |
| ASCRP3006496 | 0.236002121 | 0.909237851 | 1.0329247 | down | hsa_circRNA_102487 | hsa_circ_0000915 | circBase | chr19 | - | 18650180 | 18650530 | exonic | NM_012181 | FKBP8 | CTGACTCCAAGTACTGCTACGGCCCCCAAGGCAGGGAACGGGCTGTTGAGGAAGAAGACG |
| ASCRP3006498 | 0.687797104 | 0.943247363 | 1.0201582 | down | hsa_circRNA_100795 | hsa_circ_0000290 | circBase | chr11 | + | 34991685 | 35006275 | exonic | NM_003477 | PDHX | CTGACTCTGTAAAGGGCACATTCACTGAAATCCCCGCCAGCAATATTCGAAGAGTTATTG |
| ASCRP3006502 | 0.46912208 | 0.91119147 | 1.0261772 | down | hsa_circRNA_062035 | hsa_circ_0062035 | circBase | chr21 | + | 47410171 | 47424963 | exonic | NM_001848 | COL6A1 | CTGACTTTTCCAAATAAAGGTTTTCACTCCTCTGGCTCCAGGGGACCCAAGGGCTACAAG |
| ASCRP3006510 | 0.005228011 | 0.879037174 | 1.1888564 | down | hsa_circRNA_100037 | hsa_circ_0002158 | circBase | chr1 | - | 8601272 | 8617582 | exonic | NM_012102 | RERE | CTGAGACAAGAGTAAGAGGGACCATCTCCTCATGAACGTCAAATGGTACTACCGTCAATC |
| ASCRP3006524 | 0.642118139 | 0.929581513 | 1.015356 | down | hsa_circRNA_002271 | hsa_circ_0002271 | circBase | chr2 | - | 20974569 | 20990158 | exonic | NM_021925 | C2orf43 | CTGAGCTCCCGGTAACCCAGGTTTTTCTGCCTTTTATGTGCCATTTGCAAAGGCTTTATA |
| ASCRP3006525 | 0.377259798 | 0.91119147 | 1.0365806 | down | hsa_circRNA_051147 | hsa_circ_0051147 | circBase | chr19 | + | 41292569 | 41292883 | exonic | NM_016154 | RAB4B | CTGAGCTCTGTGGAGCCAGAGCTGATGTTCCTGGAGACCAGCGCTCTCACAGGCGAGAAC |
| ASCRP3006530 | 0.640679775 | 0.929544944 | 1.0343598 | down | hsa_circRNA_100849 | hsa_circ_0004291 | circBase | chr11 | + | 65061623 | 65063461 | exonic | NM_002689 | POLA2 | CTGAGGTACTTCGTGAAGCTCCGGCTCCCACCTTGTCTTTGTCCCGTCATTGAGAGATGT |
| ASCRP3006532 | 0.871529693 | 0.979863126 | 1.0298725 | down | hsa_circRNA_000121 | hsa_circ_0000121 | circBase | chr1 | - | 120285606 | 120286575 | antisense | NM_006623 | PHGDH | CTGAGTCCGGAATAGGAGCAGGGGCAGGTCTCCCCATCTGACACCAGTGAAGTCTGGTAG |
| ASCRP3006549 | 0.822260762 | 0.976440085 | 1.0176896 | down | hsa_circRNA_102219 | hsa_circ_0046123 | circBase | chr17 | - | 79244717 | 79250933 | exonic | NM_138570 | SLC38A10 | CTGATCTACAAGAAAATCCACAAGAACGCACTTTCCTCCCAGCCAGGTGCTGCCCACCTA |
| ASCRP3006557 | 0.182187114 | 0.909237851 | 1.0748285 | down | hsa_circRNA_100159 | hsa_circ_0011501 | circBase | chr1 | - | 34254200 | 34276465 | exonic | NM_052896 | CSMD2 | CTGATGGCAGTGGCAGTTCCCTGGGATTCAAGGCTTCTTATGAAGCCCGCATGTGTGATG |
| ASCRP3006559 | 0.851492396 | 0.978900086 | 1.0225755 | down | hsa_circRNA_405551 | | 25070500 | chr17 | - | 19823348 | 19839743 | exonic | NM_007202 | AKAP10 | CTGATGTAAGGAAATCAAAAGGTACTTCTCCCTCCAAGCCACACATCCTCTTGGATTTGA |
| ASCRP3006561 | 0.012375638 | 0.909237851 | 1.1737496 | down | hsa_circRNA_034805 | hsa_circ_0034805 | circBase | chr15 | + | 42579888 | 42603044 | exonic | NM_198141 | GANC | CTGATGTCTTCAAACAGTACTCACACCTTACAGGTTCCTCTCCTGGCTGAAATTTATGGT |
| ASCRP3006563 | 0.202491027 | 0.909237851 | 1.0198099 | down | hsa_circRNA_101632 | hsa_circ_0005889 | circBase | chr15 | + | 85657103 | 85661070 | exonic | NM_002605 | PDE8A | CTGATGTGCTTCATGCCACTGCCTATTTTCTCTCCAAGGAGAGGATAAAGGTAATCAATA |
| ASCRP3006564 | 0.492902582 | 0.91119147 | 1.0285111 | down | hsa_circRNA_102559 | hsa_circ_0051220 | circBase | chr19 | + | 41884185 | 41884424 | exonic | NM_001042595 | TMEM91 | CTGATGTTGAGGAAACCCCTCCTGGAGTTTCCCCAAAGCCATGGACAGCCCTAGTCTTCG |
| ASCRP3006567 | 0.471121446 | 0.91119147 | 1.0511747 | down | hsa_circRNA_103809 | hsa_circ_0072088 | circBase | chr5 | - | 32379220 | 32388780 | exonic | NM_016107 | ZFR | CTGATTTTCCAAGCTGGCCCTTACGTCGTCCTGACTCATCTGATGACCGTTATGTAATGA |
| ASCRP3006569 | 0.663731092 | 0.93735608 | 1.026402 | down | hsa_circRNA_020706 | hsa_circ_0020706 | circBase | chr11 | + | 810233 | 812876 | exonic | NM_001004 | RPLP2 | CTGCAAATAAAGCCTTTTTACACATCTCGATGCGCTACGTCGCCTCCTACCTGCTGGCTG |
| ASCRP3006571 | 0.425966852 | 0.91119147 | 1.0678305 | down | hsa_circRNA_059617 | hsa_circ_0059617 | circBase | chr20 | - | 23614293 | 23618574 | exonic | uc002wtn.1 | CST3 | CTGCAATAAAATAGTAGCATCGGCCGCAGCGGGTCCTCTCTATCTAGCTCCAGCCTCTCG |
| ASCRP3006575 | 0.06346076 | 0.909237851 | 1.0699993 | down | hsa_circRNA_101758 | hsa_circ_0038508 | circBase | chr16 | + | 22319468 | 22320831 | exonic | NM_018119 | POLR3E | CTGCACTCTACAGGCAAGTACCCTGTGCGTCCAGCCTCGATGACCTACGATGACATTCCG |
| ASCRP3006584 | 0.789276362 | 0.967199246 | 1.0152238 | down | hsa_circRNA_404474 | | 25070500 | chr1 | - | 33623853 | 33625545 | exonic | NM_018207 | TRIM62 | CTGCAGTACACCATCTGGAAGTCCCTGTTCCAGGACATCCACCCAGTCTTCCACCAAGAG |
| ASCRP3006593 | 0.509883901 | 0.916805276 | 1.0357234 | down | hsa_circRNA_404522 | | 25070500 | chr1 | + | 62479014 | 62483619 | intronic | ENST00000545929 | INADL | CTGCCATTATTAAGACTGCCCCATCAAAGGTCAAGCTGGTTTTCATCAGTCACTTCGAAG |
| ASCRP3006594 | 0.524844032 | 0.916971445 | 1.0312816 | down | hsa_circRNA_400616 | | 25242744 | chr10 | - | 95110953 | 95111328 | exonic | NM_013451 | MYOF | CTGCCATTGAGGGCAAAGATGAATTTTTAGGACGAAGCATTTTCTCTCCTGTGGTGAAAC |
| ASCRP3006599 | 0.219685081 | 0.909237851 | 1.0396307 | down | hsa_circRNA_085578 | hsa_circ_0085578 | circBase | chr8 | - | 131124339 | 131374017 | exonic | NM_001247996 | ASAP1 | CTGCCTCCTAGGAACGCCGGGAAAGATATCCAGGAATGATTTTCAGAGTATTTGAGAAAA |
| ASCRP3006602 | 0.482336092 | 0.91119147 | 1.044337 | down | hsa_circRNA_104137 | hsa_circ_0076995 | circBase | chr6 | - | 74228420 | 74228571 | exonic | NM_001402 | EEF1A1 | CTGCCTCTCCAGGATGTCTACAAAATTGGTGATGCCTTGGTTCAAGGGATGGAAAGTCAC |
| ASCRP3006609 | 0.054060831 | 0.909237851 | 1.0761515 | down | hsa_circRNA_103257 | hsa_circ_0063812 | circBase | chr22 | - | 46782298 | 46785396 | exonic | NM_014246 | CELSR1 | CTGCGGCCCTTCGTCATCGTCACCGCCAACATGAAATGAGAAGCTGAGCCGCAATGAGAC |
| ASCRP3006610 | 0.255473584 | 0.909237851 | 1.0961105 | down | hsa_circRNA_061081 | hsa_circ_0061081 | circBase | chr20 | - | 60888143 | 60888306 | exonic | NM_005560 | LAMA5 | CTGCGGCTCGTGTCCTACAGCGGGGTGCTCTTCTTCCTGAAGCAGCAGCTCCAAGTCGAC |
| ASCRP3006613 | 0.238929038 | 0.909237851 | 1.1070435 | down | hsa_circRNA_048574 | hsa_circ_0048574 | circBase | chr19 | - | 3983107 | 3983289 | exonic | NM_001961 | EEF2 | CTGCGTGTCAGTGCCATCTCCCTCTTCTACGAGCTCTCGGAGAATGACTTGAACTTCATC |
| ASCRP3006623 | 0.44480826 | 0.91119147 | 1.1459307 | down | hsa_circRNA_020846 | hsa_circ_0020846 | circBase | chr11 | + | 3424822 | 3427945 | exonic | NR_024248 | TSSC2 | CTGCTCCTGGAGTTTGAACAACTGACTCTTGATGGACACAACCTTCCTTCTCTCGTCTGT |
| ASCRP3006631 | 0.849256604 | 0.978900086 | 1.0058409 | down | hsa_circRNA_102396 | hsa_circ_0048122 | circBase | chr19 | - | 1011578 | 1011662 | exonic | NM_033420 | TMEM259 | CTGCTGACCGTCATCCTGGCCCTCGTCGTGGACCTGCTGCAGATGCTGGAGATGAACATG |
| ASCRP3006633 | 0.239523572 | 0.909237851 | 1.0497653 | down | hsa_circRNA_103164 | hsa_circ_0062389 | circBase | chr22 | - | 21158587 | 21159453 | exonic | NM_058004 | PI4KA | CTGCTGATCTGCTGTCTGCAGACCTCCCGACCTCTTTTGTGAAGGAGATCCATGATTTTG |
| ASCRP3006638 | 0.007223183 | 0.879037174 | 1.095607 | down | hsa_circRNA_401474 | | 25242744 | chr15 | - | 79083481 | 79090455 | exonic | NM_014272 | ADAMTS7 | CTGCTGGAAGATGAGGAGAAAGGTGTGTTCCAGCTCTCCAACGAGGACTACTTCATTGAG |
| ASCRP3006639 | 0.435415028 | 0.91119147 | 1.0138806 | down | hsa_circRNA_000750 | hsa_circ_0001749 | circBase | chr7 | + | 132990021 | 133002144 | sense overlapping | NM_021807 | EXOC4 | CTGCTGGAAGCTCAAGTGTCACCATTCATATCCCGTTTCTTCTATCAAACTGAATCAGGA |
| ASCRP3006647 | 0.600864808 | 0.929544944 | 1.0945023 | down | hsa_circRNA_101696 | hsa_circ_0037777 | circBase | chr16 | + | 5129748 | 5131057 | exonic | NM_019109 | ALG1 | CTGCTTCTAGAGTTTGAACAACTGACTCTTGATGGACACAACCTTCCTTCTCTCGTCTGT |
| ASCRP3006654 | 0.131504409 | 0.909237851 | 1.0623242 | down | hsa_circRNA_012871 | hsa_circ_0012871 | circBase | chr1 | - | 65323338 | 65335157 | exonic | NM_002227 | JAK1 | CTGCTTTGAGAAGTCTGAGGGACAGTATGATTTGGTGAAATGCCTGGCTCCTATTCGAGA |
| ASCRP3006657 | 0.844927702 | 0.978465126 | 1.0204093 | down | hsa_circRNA_053028 | hsa_circ_0053028 | circBase | chr2 | - | 25497809 | 25505580 | exonic | NM_022552 | DNMT3A | CTGGAAAAGGGAGGTGGAAAGCGGTGACACGCCAAAGGACCCTGCGGTGATCTCCAAGTC |
| ASCRP3006660 | 0.374566644 | 0.91119147 | 1.0639295 | down | hsa_circRNA_404788 | | 25070500 | chr10 | - | 90982267 | 90988155 | exonic | NM_000235 | LIPA | CTGGAACTTCTGTGCAAAACATGTTACACTGGAGCCAGGTCCCAAACCAGTTGTCTTCCT |
| ASCRP3006662 | 0.502560855 | 0.914489113 | 1.0754367 | down | hsa_circRNA_103417 | hsa_circ_0004788 | circBase | chr3 | - | 78987750 | 78988077 | exonic | NM_002941 | ROBO1 | CTGGAAGTAGCCAGCTCCCGTCTTCGTCAGGAAGATTTTCCACCTCGCATTGTTGAACAC |
| ASCRP3006663 | 0.701423668 | 0.944102043 | 1.0403533 | down | hsa_circRNA_406200 | | 25070500 | chr22 | - | 43435781 | 43442579 | sense overlapping | NM_012263 | TTLL1 | CTGGAAGTGAGTGAATGCGTCCCCGTCTCTCACGTCCAGCACTGCCAATGACCGAATCCT |
| ASCRP3006665 | 0.08704411 | 0.909237851 | 1.0777279 | down | hsa_circRNA_103160 | hsa_circ_0062261 | circBase | chr22 | - | 19462590 | 19463125 | exonic | NM_005659 | UFD1L | CTGGACCAACTCATTCTCTTTCAACATGTTCGACCACCCTATTCCCAGGGTCTTCCAAAA |
| ASCRP3006666 | 0.367419861 | 0.91119147 | 1.1683673 | down | hsa_circRNA_406325 | | 25070500 | chr3 | - | 113114596 | 113135474 | exonic | NM_018338 | CFAP44 | CTGGACCTGTGTGTCAGTTAATGTGGTCTCCCATGTCTCATGTTCATCCACATAAAACTT |
| ASCRP3006669 | 0.747354102 | 0.95565436 | 1.0150103 | down | hsa_circRNA_062456 | hsa_circ_0062456 | circBase | chr22 | - | 22326248 | 22328860 | exonic | NM_003935 | TOP3B | CTGGACTGCGACAAGGAGGGGGAGAACATCTGCTTTGAGGGAGCCTGTCCTCACACAAAG |
| ASCRP3006674 | 0.562471134 | 0.920978531 | 1.0197169 | down | hsa_circRNA_015494 | hsa_circ_0015494 | circBase | chr1 | + | 180144455 | 180145168 | exonic | NM_002826 | QSOX1 | CTGGAGCCTGCCAATTCTTCAAGGCCTTTACCAAGAACGGCTCGGGAGCAGTATTTCCAG |
| ASCRP3006677 | 0.718902168 | 0.948649554 | 1.0126541 | down | hsa_circRNA_100963 | hsa_circ_0024479 | circBase | chr11 | + | 118484530 | 118485397 | exonic | NM_015157 | PHLDB1 | CTGGAGGAAGGAGCTCTGGAGCCTTAGGACCATGGACGCTCTCAATAGGAACCAAATAGG |
| ASCRP3006679 | 0.006817862 | 0.879037174 | 1.1012538 | down | hsa_circRNA_013433 | hsa_circ_0013433 | circBase | chr1 | - | 109865553 | 109867711 | exonic | NM_002959 | SORT1 | CTGGAGGACTTTCTCTGGTTCTCCACAGACGAAGGTCAATGCTGGCAAACCTACACGTTC |
| ASCRP3006680 | 0.606879576 | 0.929544944 | 1.0358622 | down | hsa_circRNA_038815 | hsa_circ_0038815 | circBase | chr16 | - | 28146536 | 28181230 | exonic | NM_015171 | XPO6 | CTGGAGGAGTTGGATGATGAGACTCTGGATGACGATTTGATCCAGTCCCCTGTGACAACC |
| ASCRP3006683 | 0.988239515 | 0.998148217 | 1.0005025 | down | hsa_circRNA_104466 | hsa_circ_0003655 | circBase | chr7 | + | 127344892 | 127361454 | exonic | NM_014390 | SND1 | CTGGAGGGGGAGAACACCCAGAATGGCAACATCACAGAGCTCCTCCTGAAGGAAGGTTTC |
| ASCRP3006684 | 0.90642135 | 0.982324123 | 1.0090113 | down | hsa_circRNA_026428 | hsa_circ_0026428 | circBase | chr12 | - | 52882111 | 52884517 | exonic | NM_005554 | KRT6A | CTGGAGGGTGAGGAGTGCAGGAGCTGTCCCAGATGCAGACCCACATCTCAGACACATCTG |
| ASCRP3006687 | 0.636303758 | 0.929544944 | 1.0657862 | down | hsa_circRNA_038737 | hsa_circ_0038737 | circBase | chr16 | - | 27473614 | 27474913 | exonic | NM_001520 | GTF3C1 | CTGGAGTTGCTCCAGGGTTCACAGAGAGTTTCGGAGCTGCCAACATCTCCCAGGCAGCAC |
| ASCRP3006690 | 0.500373832 | 0.914489113 | 1.0208538 | down | hsa_circRNA_004807 | hsa_circ_0004807 | circBase | chr5 | - | 33576158 | 33596165 | exonic | NM_030955 | ADAMTS12 | CTGGATCGTCGGAAACTGGAGCGAGGTATCCGCCGCCAAACTGCCCATTGCATAAAGAAG |
| ASCRP3006694 | 0.779733167 | 0.966482321 | 1.0097981 | down | hsa_circRNA_062709 | hsa_circ_0062709 | circBase | chr22 | - | 29025539 | 29025818 | exonic | NM_001145418 | TTC28 | CTGGATGATGCAATCAAAGCTCGACTTCTCAATCCCAAGTGGCCAAAGATTCCTCTCTTT |
| ASCRP3006699 | 0.30010106 | 0.909237851 | 1.0332379 | down | hsa_circRNA_101338 | hsa_circ_0031570 | circBase | chr14 | - | 31849672 | 31852977 | exonic | NM_015473 | HEATR5A | CTGGATGGTTGCTGATTTCTGCTCTGATGACATTAGGTCTCCTTGACAGTATCTTGTCAG |
| ASCRP3006701 | 0.863148602 | 0.979863126 | 1.0037273 | down | hsa_circRNA_406316 | | 25070500 | chr3 | - | 101400084 | 101401272 | intronic | ENST00000394077 | RPL24 | CTGGATGTGAGAGATGGGTAATAATAGGGTAACAAAAGTTTTGTGGAGTAATTATGGGGT |
| ASCRP3006702 | 0.01662208 | 0.909237851 | 1.0548244 | down | hsa_circRNA_001668 | hsa_circ_0001668 | circBase | chr7 | + | 1529263 | 1532681 | antisense | NM_001080453 | INTS1 | CTGGATGTTTTCAGGGTGATGGTAGGAGGGGCCAGGCCTTCCAGTAGAGGGTAGAGATGG |
| ASCRP3006711 | 0.268643783 | 0.909237851 | 1.036786 | down | hsa_circRNA_001820 | hsa_circ_0000728 | circBase | chr16 | + | 89979183 | 89979271 | intronic | ENST00000539976 | MC1R | CTGGCCATTTCTAGGAGCGGTGCCCTGAGGGCTGAGCCACGTGTGCCCATCTCAGACGTG |
| ASCRP3006712 | 0.238499652 | 0.909237851 | 1.0336612 | down | hsa_circRNA_406399 | | 25070500 | chr3 | - | 176665094 | 176700237 | intergenic |  |  | CTGGCTAACAGATCTCCTTGAGTGTGTGATGACAGTGACCGGGAATGAATTCTAGACTCA |
| ASCRP3006725 | 0.625743793 | 0.929544944 | 1.0895483 | down | hsa_circRNA_057362 | hsa_circ_0057362 | circBase | chr2 | + | 189851784 | 189858808 | exonic | NM_000090 | COL3A1 | CTGGGATTAATGGTAGTCCTGGTGGTAAAGGCGAAATGAACTATTCTCCCCAGTATGATT |
| ASCRP3006726 | 0.064516383 | 0.909237851 | 1.0909769 | down | hsa_circRNA_002149 | hsa_circ_0001627 | circBase | chr6 | - | 90959407 | 90981660 | intronic | ENST00000343122 | BACH2 | CTGGGCTCAGCGCGTTCGTTCACATAGCTCCCAGTTTTAACATTTCGCCACCTACTGAAG |
| ASCRP3006727 | 0.220703028 | 0.909237851 | 1.0373287 | down | hsa_circRNA_000006 | hsa_circ_0001821 | circBase | chr8 | + | 128902834 | 128903244 | exonic | uc003ysl.3 | PVT1 | CTGGGCTTGAGGCCTGATCTTTTGGCCAGAAGGAGATTAAAAAGATGCCCCTCAAGATGG |
| ASCRP3006730 | 0.755550584 | 0.957859239 | 1.0118676 | down | hsa_circRNA_007467 | hsa_circ_0007467 | circBase | chr15 | - | 83858112 | 83861060 | exonic | ENST00000563404 | HDGFRP3 | CTGGGGGAGAAGGACAGAAGATGAATTCAGATTGGAACTTTTGGCAGAAAGTCAAAACTG |
| ASCRP3006731 | 0.851012621 | 0.978900086 | 1.0078086 | down | hsa_circRNA_104801 | hsa_circ_0087319 | circBase | chr9 | - | 84225134 | 84235472 | exonic | NM_005077 | TLE1 | CTGGGGGGAAACCAGTAATTCCCTCCTGGTCCCAGACAGTCTAAGAGGCACAGATAAACG |
| ASCRP3006742 | 0.900839165 | 0.981257955 | 1.0040153 | down | hsa_circRNA_046481 | hsa_circ_0046481 | circBase | chr17 | + | 80721840 | 80899359 | exonic | NM_005993 | TBCD | CTGGTGCCCCAGAATGGATGATGAACTTGTTGTTGGACATAGTGCAAGATCAGACATCTC |
| ASCRP3006744 | 0.919572002 | 0.98346125 | 1.003311 | down | hsa_circRNA_005634 | hsa_circ_0005634 | circBase | chr20 | + | 35127990 | 35154409 | exonic | NM_183006 | DLGAP4 | CTGGTGGAGACCCCCGAGAAGAGGAAGGTTGACTGCATTCAGCCAGTGCCAAAAGAGGAG |
| ASCRP3006746 | 0.67473425 | 0.93829504 | 1.0179419 | down | hsa_circRNA_102464 | hsa_circ_0008950 | circBase | chr19 | + | 14568842 | 14569187 | exonic | NM_002741 | PKN1 | CTGGTGGCTGAGGTGAAGTCAGCACTGTGCTTAAGCTGGATAACACAGTGGTGGGGCAGA |
| ASCRP3006751 | 0.196420566 | 0.909237851 | 1.0418787 | down | hsa_circRNA_092556 | hsa_circ_0001583 | circBase | chr6 | - | 26056122 | 26056299 | sense overlapping | NM_005319 | HIST1H1C | CTGTAACCAAGAAAGTGGCTAAGAGCCCAAAGAAGGCCAAGGTTAAAAAGGCGGGCGGAA |
| ASCRP3006754 | 0.707282593 | 0.945024804 | 1.0099248 | down | hsa_circRNA_044334 | hsa_circ_0044334 | circBase | chr17 | + | 46022924 | 46026674 | exonic | NM_018129 | PNPO | CTGTAATAAAAGCCTATCAGCCGTGCACTTTAGACTCTAATCCCTTTGCTTCCCTTGTCT |
| ASCRP3006761 | 0.528133132 | 0.91700841 | 1.0384353 | down | hsa_circRNA_048545 | hsa_circ_0048545 | circBase | chr19 | - | 3976053 | 3985461 | exonic | NM_001961 | EEF2 | CTGTACTAGTGCCATTGGAATAATAAATTTGATAAGGTGGTGACTCTTCCGCCGTCGTCG |
| ASCRP3006762 | 0.022729681 | 0.909237851 | 1.0437093 | down | hsa_circRNA_101065 | hsa_circ_0006763 | circBase | chr12 | + | 52139686 | 52145377 | exonic | NM_014191 | SCN8A | CTGTAGGAAATCTGGCTACAACTGAGGTGGAAATTAAGAAGAAAGGCCCTGGATCTCTTT |
| ASCRP3006769 | 0.891281332 | 0.979863126 | 1.0062625 | down | hsa_circRNA_081536 | hsa_circ_0081536 | circBase | chr7 | + | 100457506 | 100459533 | exonic | NM_020246 | SLC12A9 | CTGTATGTGCTGGGCCACGTCACCCTGGGAGACCTCGGACCCTGCTGCAGGAAGACTATG |
| ASCRP3006776 | 0.782299089 | 0.966482321 | 1.0115517 | down | hsa_circRNA_017667 | hsa_circ_0017667 | circBase | chr10 | + | 7830092 | 7844817 | exonic | NM_005174 | ATP5C1 | CTGTCATCACAAAAGAGTTGATTGAAATTATCTCTGGTGCTGCAGCTCTGGGAGGGGCGC |
| ASCRP3006778 | 0.638388354 | 0.929544944 | 1.0236262 | down | hsa_circRNA_004135 | hsa_circ_0004135 | circBase | chr9 | - | 113734352 | 113798481 | intronic | uc011lwn.2 | LPAR1 | CTGTCATGGCTGCCATCTCTACTTCCATCCCTGTAATTTCACAGCCCCAGGGAACAGCTC |
| ASCRP3006781 | 0.998781489 | 0.99972787 | 1.0000883 | down | hsa_circRNA_005500 | hsa_circ_0005500 | circBase | chr20 | + | 32619327 | 32621045 | exonic | ENST00000413297 | RALY | CTGTCCCCTCACGTCTCATGTGTGTACTCCTACCATGCAGGTGAGCCCTATTCCCAGAGG |
| ASCRP3006783 | 0.810486669 | 0.974895666 | 1.0251711 | down | hsa_circRNA_100821 | hsa_circ_0005204 | circBase | chr11 | - | 57258696 | 57259335 | exonic | NM_003627 | SLC43A1 | CTGTCCTCGGAGATGCCAGGGTCTGTCCCCTTACGCAAGAGCCTCTGCTCCCCCACTTTC |
| ASCRP3006789 | 0.346593507 | 0.91119147 | 1.0884402 | down | hsa_circRNA_406046 | | 25070500 | chr2 | - | 219130434 | 219130553 | intronic | ENST00000248450 | AAMP | CTGTCTCCTCTCCCCACCGTCTTTCTCTCCATCAAGCACCCGGATTCCGTGTGAGTGGAC |
| ASCRP3006792 | 0.426866029 | 0.91119147 | 1.0514823 | down | hsa_circRNA_102211 | hsa_circ_0045905 | circBase | chr17 | + | 76410959 | 76415806 | exonic | NR_110602 | PGS1 | CTGTCTCTAGCATCACCTCTCAGCACGATTTTCCCGAGAGTTCACAGGCCTCTGGCTGTA |
| ASCRP3006796 | 0.181111922 | 0.909237851 | 1.0618371 | down | hsa_circRNA_102466 | hsa_circ_0049785 | circBase | chr19 | + | 14705443 | 14706127 | exonic | NM_207390 | CLEC17A | CTGTGAATCTTGAGCCTTCTCCATTGCAGCCATCCCTGGCCGCAAAGGAAACAGAGAAAC |
| ASCRP3006798 | 0.962231205 | 0.99396088 | 1.0022463 | down | hsa_circRNA_103278 | hsa_circ_0001265 | circBase | chr3 | + | 9711115 | 9712854 | exonic | NM_022485 | MTMR14 | CTGTGACCTGATGGTGGAGAACAAGAAGGTGAAGTTTGGCATGAAGGGGTGCAGATGATG |
| ASCRP3006800 | 0.952296133 | 0.992077624 | 1.0024874 | down | hsa_circRNA_400042 | hsa_circ_0092302 | circBase | chr19 | - | 2431110 | 2431310 | intronic | ENST00000325327 | LMNB2 | CTGTGACTGTTTTGGAACCCAGAACAGAATGTGTAGCTCACAGGGGGGGTCCCCTGAGAG |
| ASCRP3006802 | 0.003048116 | 0.870215632 | 1.0490541 | down | hsa_circRNA_404429 | | 25070500 | chr1 | - | 5964676 | 5969273 | exonic | NM_015102 | NPHP4 | CTGTGAGCAGAGATGGCACCTTTGATGCTGGCCCGCCAGCGCCAGTACCTCGAGTTCTCG |
| ASCRP3006808 | 0.127016185 | 0.909237851 | 1.101767 | down | hsa_circRNA_081599 | hsa_circ_0081599 | circBase | chr7 | + | 101183173 | 101183330 | exonic | NM_133457 | COL26A1 | CTGTGCCACGACAGAGAAGGCCCACGGGCCCAGCCGGTCCTCCTGCTAGAAGCAGCAGAA |
| ASCRP3006812 | 0.301741033 | 0.909237851 | 1.0367639 | down | hsa_circRNA_063013 | hsa_circ_0063013 | circBase | chr22 | - | 34000420 | 34046654 | exonic | NM_004737 | LARGE | CTGTGCGTGTGGACTTCTACAATGCAGACGAGCTCAAGATGGAAAGCCCGTGTCTCTGTC |
| ASCRP3006813 | 0.351169109 | 0.91119147 | 1.0551189 | down | hsa_circRNA_103457 | hsa_circ_0067159 | circBase | chr3 | - | 127294245 | 127294652 | exonic | NM_001136053 | TPRA1 | CTGTGCTTCGACATCATCGAGGGGCTCTGGTCTACTCTCTGGTGGTCATCCTTCCCAAGA |
| ASCRP3006824 | 0.984567188 | 0.998148217 | 1.0006037 | down | hsa_circRNA_058097 | hsa_circ_0058097 | circBase | chr2 | - | 216240351 | 216288249 | exonic | NM_002026 | FN1 | CTGTGGTCATCGACGCCTCCACTGTTTTGGTTCAGACTCGAGGAGGAAATTCCAATGGTG |
| ASCRP3006832 | 0.068751783 | 0.909237851 | 1.0573699 | down | hsa_circRNA_000874 | hsa_circ_0000874 | circBase | chr19 | - | 3977580 | 3978020 | intronic | ENST00000600794 | EEF2 | CTGTGTGAGGAGAACATGCGGGGTGTCCGCCCGTCAGGAGCTCAAGCAGCGGGCGCGCTA |
| ASCRP3006835 | 0.724448012 | 0.949785489 | 1.0129065 | down | hsa_circRNA_003704 | hsa_circ_0003704 | circBase | chr16 | - | 23091266 | 23093878 | exonic | NM_020718 | USP31 | CTGTGTGCAATCACCATGGCACCATGCAAGGGGGGCACTACACAGCTTGCCCCCGATGAT |
| ASCRP3006840 | 0.48684686 | 0.91119147 | 1.0208894 | down | hsa_circRNA_400107 | | 25242744 | chr1 | - | 3659367 | 3659754 | exonic | NR_033708 | TP73-AS1 | CTGTGTTTTCTCTGGGACATGTCTTCTGCCCAGGCACTTTGATCCGGTTTTCCAGTTCTT |
| ASCRP3006841 | 0.492745286 | 0.91119147 | 1.061663 | down | hsa_circRNA_104319 | hsa_circ_0079492 | circBase | chr7 | + | 16720925 | 16729531 | exonic | NM_014038 | BZW2 | CTGTTACCTCGTCTTTGAGAAAAGCCAACTTAGACAAGAGGCTGCTTCCCCTGGAGGAAC |
| ASCRP3006845 | 0.497023867 | 0.913224439 | 1.033819 | down | hsa_circRNA_001379 | hsa_circ_0000516 | circBase | chr14 | + | 20811398 | 20811483 | antisense | NR_002312 | RPPH1 | CTGTTAGGGCCGCCTCTGGCCCTATCTGACCTCGCGCGGAGCCCCGTTCTCTGGGAACTC |
| ASCRP3006848 | 0.160263172 | 0.909237851 | 1.0773982 | down | hsa_circRNA_102448 | hsa_circ_0002579 | circBase | chr19 | + | 11223953 | 11224438 | exonic | NM_000527 | LDLR | CTGTTCATGGGCTCCATCGCCTACCTCTTCTTCACCAACCGGCACGAGGTCAGGAAGATG |
| ASCRP3006855 | 0.864347091 | 0.979863126 | 1.0060315 | down | hsa_circRNA_104763 | hsa_circ_0086720 | circBase | chr9 | - | 33953282 | 33996331 | exonic | NM_018449 | UBAP2 | CTGTTCTCCTCAGAGCCTGCTTATGGAAGTGACAGGGAAAAATCAGGATGAATGCATAGT |
| ASCRP3006859 | 0.60730906 | 0.929544944 | 1.0244266 | down | hsa_circRNA_103507 | hsa_circ_0067913 | circBase | chr3 | - | 169863210 | 169890500 | exonic | NM_024947 | PHC3 | CTGTTGTCTCGTCGTCATCGTCATCTTCCTGTCAGTCTGCAGCTACTCAGGTAATTCAAC |
| ASCRP3006860 | 0.258873715 | 0.909237851 | 1.0472234 | down | hsa_circRNA_049537 | hsa_circ_0049537 | circBase | chr19 | - | 12778880 | 12780047 | exonic | NM_016145 | WDR83OS | CTGTTGTTAATAAAGTTTTTCACTCTGGCTGTCGTACAAGCCCCCGCCGAGCGAATGTAA |
| ASCRP3006866 | 0.113481838 | 0.909237851 | 1.0303954 | down | hsa_circRNA_100571 | hsa_circ_0018004 | circBase | chr10 | + | 27024168 | 27024508 | exonic | NM_014317 | PDSS1 | CTGTTTGCCTGTCAGCAGGTCTCTGTTCTAGGATGTCCCGACCCAGTGGTGCATGAGATC |
| ASCRP3006868 | 0.591050327 | 0.927905266 | 1.0315495 | down | hsa_circRNA_021529 | hsa_circ_0021529 | circBase | chr11 | - | 27645540 | 27645692 | exonic | TCONS_00019121 | XLOC_009399 | CTGTTTTCTTCTTGACCTACCAGTCTGCCCTGCTCATACTTGATCTGGATGAGACTGATA |
| ASCRP3006869 | 0.217385504 | 0.909237851 | 1.0655349 | down | hsa_circRNA_103502 | hsa_circ_0001358 | circBase | chr3 | + | 169694733 | 169706147 | exonic | NM_003262 | SEC62 | CTGTTTTGTAGCCAGTATTCTTCTCCTTGCTGTTGCTTCAAAAGCAGTGGACTGTCTTTT |
| ASCRP3006870 | 0.250393508 | 0.909237851 | 1.0639522 | down | hsa_circRNA_100541 | hsa_circ_0017636 | circBase | chr10 | - | 7285519 | 7327916 | exonic | NM_001018039 | SFMBT2 | CTTAAAAATGGAGTCAGCCTCACTCCTCCCAAAGCAATCAAAGAGAAGTACACAGACTGG |
| ASCRP3006871 | 0.068575933 | 0.909237851 | 1.0585836 | down | hsa_circRNA_032244 | hsa_circ_0032244 | circBase | chr14 | + | 65879447 | 66028484 | exonic | NR_038170 | FUT8 | CTTAAAACAACAGAATGAAGACTTGAGGCGAATGGCCGAATCTCTCCGATTCGCGCGCGC |
| ASCRP3006872 | 0.240754388 | 0.909237851 | 1.0441818 | down | hsa_circRNA_101367 | hsa_circ_0001998 | circBase | chr14 | + | 65922338 | 66028484 | exonic | NM_178155 | FUT8 | CTTAAAACAACAGAATGAAGACTTGAGGCGAATGGCCGAATCTCTCCGGTTGCTGCTTTT |
| ASCRP3006874 | 0.826424306 | 0.977459664 | 1.0101268 | down | hsa_circRNA_103470 | hsa_circ_0067301 | circBase | chr3 | - | 129280642 | 129280746 | exonic | NM_015103 | PLXND1 | CTTAACACGCTGGCCCATTACAAGAGTGGTTCGCCTCCAGCACACAGAGCTACATCCTTC |
| ASCRP3006881 | 0.625547241 | 0.929544944 | 1.0242421 | down | hsa_circRNA_103971 | hsa_circ_0001541 | circBase | chr5 | + | 139819703 | 139828890 | exonic | NM_017747 | ANKHD1 | CTTAATGGAGGCCTGCATGTCGCAGTCTAGCAGAAGCTTGTTCAGATGGGGATGTTAATG |
| ASCRP3006893 | 0.785303985 | 0.966801146 | 1.019546 | down | hsa_circRNA_102690 | hsa_circ_0054033 | circBase | chr2 | - | 37105033 | 37105170 | exonic | NM_003162 | STRN | CTTACGGTGGCCAATGAAGCAGACTCACTAACTTATGATTGGAAGCATTGACATTTCCTC |
| ASCRP3006894 | 0.124270023 | 0.909237851 | 1.1127252 | down | hsa_circRNA_406934 | | 25070500 | chr7 | - | 31815277 | 31867987 | exonic | NM_001191057 | PDE1C | CTTACGTTGCCAGGGTGACAGAGAAGCAGAGCTGGGGCTGCCTTTTTCTCCTCTGTGTGA |
| ASCRP3006895 | 0.365696499 | 0.91119147 | 1.0450446 | down | hsa_circRNA_404771 | | 25070500 | chr10 | + | 71142242 | 71146174 | exonic | NM_000188 | HK1 | CTTACTAAGGGATGCGATAAAAAGGAGAGAGGTATTCCCGGCGTTTCCACAAGACTCTAA |
| ASCRP3006896 | 0.85900161 | 0.97947761 | 1.0082234 | down | hsa_circRNA_103405 | hsa_circ_0066380 | circBase | chr3 | + | 58255047 | 58256791 | exonic | NM_020676 | ABHD6 | CTTACTACCCATCGGATGTCTCCAGCCTGTGTCTCGTGTGTCCTGCTGTTCCTTCCAAAG |
| ASCRP3006897 | 0.090519657 | 0.909237851 | 1.1845116 | down | hsa_circRNA_050649 | hsa_circ_0050649 | circBase | chr19 | - | 36245469 | 36247930 | exonic | ENST00000004982 | HSPB6 | CTTACTCTCCCAATAAATGTGCTAGAGCTCTGCACTGCAACGCGGAGGAGCAGGATGGAG |
| ASCRP3006898 | 0.20169585 | 0.909237851 | 1.1452649 | down | hsa_circRNA_050648 | hsa_circ_0050648 | circBase | chr19 | - | 36245469 | 36246795 | exonic | ENST00000004982 | HSPB6 | CTTACTCTCCCAATAAATGTGCTAGAGCTCTGCGTGCCGACGGACCCCGGCCACTTTTCG |
| ASCRP3006899 | 0.2586419 | 0.909237851 | 1.1399357 | down | hsa_circRNA_102505 | hsa_circ_0050395 | circBase | chr19 | - | 33122307 | 33133051 | exonic | NM_032139 | ANKRD27 | CTTACTCTCTCAGATGACTTCGTCTCCCACCGACTGCCTGTTTAAGCTTTAACATACCTC |
| ASCRP3006904 | 0.464077133 | 0.91119147 | 1.0191637 | down | hsa_circRNA_008546 | hsa_circ_0008546 | circBase | chr15 | - | 43242459 | 43258484 | exonic | NM_174916 | UBR1 | CTTACTTGGATGAATATGGAGAAACAGACCCTGGCCTGAACTCCATTGGGTGTGATATTC |
| ASCRP3006909 | 0.459482692 | 0.91119147 | 1.0262163 | down | hsa_circRNA_400305 | | 25242744 | chr1 | + | 116926635 | 116944277 | exonic | NM_000701 | ATP1A1 | CTTAGGATGTATCCCCTCAAGTTGGACGTGATAAGTATGAGCCTGCAGCTGTTTCAGAAC |
| ASCRP3006910 | 0.233037289 | 0.909237851 | 1.0647517 | down | hsa_circRNA_079245 | hsa_circ_0079245 | circBase | chr7 | + | 5327436 | 5327616 | exonic | NM_001040661 | SLC29A4 | CTTAGGGCCAGGGGCGTCCCAGCTTTCACGGATACTACAGAGGCTGCCATGGGCTCCGTG |
| ASCRP3006921 | 0.412112383 | 0.91119147 | 1.0396263 | down | hsa_circRNA_401586 | | 25242744 | chr16 | + | 29916172 | 29917448 | exonic | uc002duu.3 | ASPHD1 | CTTATGTCTTCTCCTAGTACAACCAAACAGGCCTAAAGATCCCTCCTGGCTGTGAGCTGG |
| ASCRP3006924 | 0.062928526 | 0.909237851 | 1.0776632 | down | hsa_circRNA_404284 | | 25242744 | chr9 | + | 131730784 | 131731793 | exonic | NM_015354 | NUP188 | CTTATTTGTCAGACAGAGCGCCAAGTGTCTCGCTGGTTTGTTCAGTGCCTTCGGGAACAG |
| ASCRP3006930 | 0.577463853 | 0.923965283 | 1.0365376 | down | hsa_circRNA_402533 | | 25242744 | chr20 | - | 18022177 | 18022367 | exonic | NM_021220 | OVOL2 | CTTCAACGACACCTTCGACCTGAAGAGGCACGTCCGCACACACACAGTTCACCACAGGCA |
| ASCRP3006931 | 0.039790475 | 0.909237851 | 1.2885361 | down | hsa_circRNA_025500 | hsa_circ_0025500 | circBase | chr12 | - | 12672795 | 12715448 | exonic | NM_030640 | DUSP16 | CTTCAACTCTGTTCACCTGCTTGCAGGTTTTTAAACGATTATTACCACCTCTCTCTCCCC |
| ASCRP3006933 | 0.095463992 | 0.909237851 | 1.1080002 | down | hsa_circRNA_001754 | hsa_circ_0000559 | circBase | chr14 | - | 89747293 | 89750866 | exonic | ENST00000557718 | FOXN3 | CTTCAAGAGAAATGGAGCCCTTCTCCAAGGGAAGGATGACTAAGAGTTTAGTGCTGGAAG |
| ASCRP3006955 | 0.148765827 | 0.909237851 | 1.0408875 | down | hsa_circRNA_406677 | | 25070500 | chr5 | - | 138456723 | 138463542 | exonic | NM_022464 | SIL1 | CTTCAGCCAGGTCCCTGACTATGGCTCCCCAGAGCCTGCCTTCATCTAGGATGGCTCCTC |
| ASCRP3006964 | 0.56562256 | 0.920978531 | 1.0698236 | down | hsa_circRNA_032475 | hsa_circ_0032475 | circBase | chr14 | + | 72165697 | 72176381 | exonic | NM_015556 | SIPA1L1 | CTTCAGTATAAACGATGCTGCTTCCCACACAAGGCTGTCTCCTGGTTCGGACATCTATGT |
| ASCRP3006980 | 0.554178031 | 0.919549314 | 1.0406602 | down | hsa_circRNA_100021 | hsa_circ_0009456 | circBase | chr1 | - | 5987708 | 6022009 | exonic | NM_015102 | NPHP4 | CTTCCAGGAAACCACCCACGTCCCCTTCGAAAAACAGACACATGACCCTCATTGAGAACT |
| ASCRP3006982 | 0.226747753 | 0.909237851 | 1.0431418 | down | hsa_circRNA_001858 | hsa_circ_0001858 | circBase | chr9 | - | 37006469 | 37020798 | exonic | NM_016734 | PAX5 | CTTCCAGTCACAGCATAGGACATGGAGGAGTGAATCAGCTTGGGGGGGTTTTTGTGAATG |
| ASCRP3006997 | 0.646523763 | 0.931414925 | 1.0296321 | down | hsa_circRNA_405724 | | 25070500 | chr19 | - | 7692128 | 7693184 | exonic | NM_020196 | XAB2 | CTTCCTCAAGGAGGAAGAGGACCTCCCCTATGAGGAGGAAATCATGCGGAACCAATTCTC |
| ASCRP3007000 | 0.369819294 | 0.91119147 | 1.0266968 | down | hsa_circRNA_100842 | hsa_circ_0022591 | circBase | chr11 | + | 62652648 | 62653080 | exonic | NM_002394 | SLC3A2 | CTTCCTGGACAGGCTCTTGATTGCGGGGACTAACTCCTCCGACCTTCAGCAGATCCTGAG |
| ASCRP3007004 | 0.807406171 | 0.974037816 | 1.0082948 | down | hsa_circRNA_407306 | | 25070500 | chrX | + | 84301451 | 84303379 | intronic | uc010nmp.3 | APOOL | CTTCCTTGACACTCCCATATCTGGACAGCTCAAATCTAGATGGGAAAACTGACAACCATG |
| ASCRP3007006 | 0.483128698 | 0.91119147 | 1.1115917 | down | hsa_circRNA_101555 | hsa_circ_0001955 | circBase | chr15 | - | 64495280 | 64508912 | exonic | NM_022048 | CSNK1G1 | CTTCGAAATCAGGTGAAGGTCTCCCACAGGTGTATTACTTTGGACCATGTGGGAAATATA |
| ASCRP3007011 | 0.022068935 | 0.909237851 | 1.0483074 | down | hsa_circRNA_404583 | | 25070500 | chr1 | + | 151630710 | 151665959 | exonic | NM_030918 | SNX27 | CTTCGAGAGGGTGTTCTGCGAGCTCAAGTGGAGAAAAGAGGTATATAATGTTTACATGGC |
| ASCRP3007021 | 0.306652445 | 0.909237851 | 1.1446989 | down | hsa_circRNA_404458 | | 25070500 | chr1 | - | 25644553 | 25645709 | intronic | ENST00000473314 | C1orf63 | CTTCGGTCTCCCAAACTGCTGCAATTACAGTGAAGAATAAGAAAAGACCTCCTGGTACTT |
| ASCRP3007026 | 0.462533704 | 0.91119147 | 1.016357 | down | hsa_circRNA_405620 | | 25070500 | chr17 | + | 66303083 | 66397591 | exonic | NM_014960 | ARSG | CTTCTACATTACCGGAAACCTTACAGAAACATGAAGCCCTCAACCATCTGCTACTCAGTT |
| ASCRP3007034 | 0.448527128 | 0.91119147 | 1.0331929 | down | hsa_circRNA_104053 | hsa_circ_0002958 | circBase | chr6 | + | 5368782 | 5369415 | exonic | NM_006567 | FARS2 | CTTCTCCAAGCATGAGAACCTGTGAGAAGTTTCTACAATGGTGGGCTCAGCTCTCAGGAG |
| ASCRP3007036 | 0.809467473 | 0.974638647 | 1.0158044 | down | hsa_circRNA_405527 | | 25070500 | chr17 | - | 3474817 | 3477249 | exonic | NM_018727 | TRPV1 | CTTCTCCCTGCGGTCAAGCAGAGCGGTGGTGACGCTGATTGAAGACGGGAAGAATGACTC |
| ASCRP3007039 | 0.079276319 | 0.909237851 | 1.1123356 | down | hsa_circRNA_104387 | hsa_circ_0080425 | circBase | chr7 | + | 70880874 | 70886091 | exonic | NM_022479 | WBSCR17 | CTTCTCTCCCCATCAGAGGAGCTGAAGGTCCCCCTAGAGGAGTATGTCCACAAACGCTAC |
| ASCRP3007043 | 0.151439335 | 0.909237851 | 1.0658825 | down | hsa_circRNA_100207 | hsa_circ_0012277 | circBase | chr1 | + | 46290104 | 46295253 | exonic | NM_015112 | MAST2 | CTTCTCTTACTGCTGGCCTGGATGTAGTAACTGGAGTTAGTCCCCTGCTCTTCAGGAAAC |
| ASCRP3007047 | 0.005525939 | 0.879037174 | 1.1021107 | down | hsa_circRNA_101257 | hsa_circ_0030062 | circBase | chr13 | - | 41826780 | 41837620 | exonic | uc001uxx.3 | MTRF1 | CTTCTGAATTATACACCAGCAGATTATGGTACTCACAGTTCTTTCCTGATCTTCCCCGCC |
| ASCRP3007059 | 0.221153322 | 0.909237851 | 1.1177637 | down | hsa_circRNA_100476 | hsa_circ_0016863 | circBase | chr1 | + | 230795209 | 230807386 | exonic | NM_007357 | COG2 | CTTCTTCGAGAAGTCACAGGAGGTGCCATCTCCAGGAAGATTTCGATGTCGATCATTTTG |
| ASCRP3007067 | 0.215164512 | 0.909237851 | 1.0415452 | down | hsa_circRNA_001948 | hsa_circ_0001948 | circBase | chrX | - | 149962163 | 149984551 | exonic | NM_031462 | CD99L2 | CTTGAAGACATAGTAGGGGGTGGAGAATACAAACCTGACAAGGGTAAAGAGCCATGGGAC |
| ASCRP3007068 | 0.219466442 | 0.909237851 | 1.1291926 | down | hsa_circRNA_406142 | | 25070500 | chr21 | - | 34974539 | 34985912 | exonic | NM_145858 | CRYZL1 | CTTGAAGATAAGCAGTGCCTTGAAAGATTCAGACCTCCCATAGGAATTTTGCCCCTGGAC |
| ASCRP3007076 | 0.636273492 | 0.929544944 | 1.063163 | down | hsa_circRNA_000996 | hsa_circ_0001901 | circBase | chr9 | - | 138773785 | 138774005 | intronic | ENST00000312405 | CAMSAP1 | CTTGACCAGAAATGCCAGGCTTGGAACAGTGATGGTGCGGGGGAGGCAGGTCTCCTGTCT |
| ASCRP3007077 | 0.274721502 | 0.909237851 | 1.0470844 | down | hsa_circRNA_002761 | hsa_circ_0002761 | circBase | chr3 | - | 185407142 | 185407415 | exonic | NM_006548 | IGF2BP2 | CTTGACCATAAAGAACATCACTAAGCAGACCCAGTCCCGAGCCATGGAGAAGCTAAGCGG |
| ASCRP3007080 | 0.553612073 | 0.919549314 | 1.029509 | down | hsa_circRNA_400040 | hsa_circ_0092305 | circBase | chr17 | - | 79872718 | 79873138 | intronic | ENST00000328666 | SIRT7 | CTTGACTCCTGGTGGTTGGACCTGTTGTGTGTTTTACTTTCTAGGAGCTCCCCCTCTGAA |
| ASCRP3007083 | 0.551682924 | 0.919549314 | 1.0332912 | down | hsa_circRNA_405469 | | 25070500 | chr16 | + | 56792449 | 56839544 | exonic | NM_014669 | NUP93 | CTTGACTTTACTCAAGAAAGCGAGGTCAGTTCTCCTCGGGTCTCGGGGACTTGACATATC |
| ASCRP3007085 | 0.607507541 | 0.929544944 | 1.0190653 | down | hsa_circRNA_001964 | hsa_circ_0001964 | circBase | chr12 | + | 27521194 | 27533337 | exonic | NM_020183 | ARNTL2 | CTTGAGATCTTTAAAAGGTAAAGTGTTGAGAGAGGAGAACCAGTGCATTGCTCCTGTGGT |
| ASCRP3007091 | 0.544892613 | 0.919549314 | 1.0217277 | down | hsa_circRNA_060170 | hsa_circ_0060170 | circBase | chr20 | + | 34770193 | 34778719 | exonic | NM_012156 | EPB41L1 | CTTGATGGAGGTAGCCCCTGGAATTTTGCCTTCACAGTCAAGTTCTACCCGCCTGATCCT |
| ASCRP3007092 | 0.392537395 | 0.91119147 | 1.0749036 | down | hsa_circRNA_101822 | hsa_circ_0006275 | circBase | chr16 | - | 58610366 | 58617086 | exonic | NM_016284 | CNOT1 | CTTGATGTGGCCCAGGACTTGAAGTATTGAAGAATGTCGCAATATAATCGTGCAGTTTGG |
| ASCRP3007098 | 0.958795531 | 0.993354918 | 1.0048359 | down | hsa_circRNA_102207 | hsa_circ_0045881 | circBase | chr17 | + | 76198579 | 76198832 | exonic | NM_001010982 | AFMID | CTTGCCTTTCTTCCTGTTCTTTCACGGAGGATACTGGCAGAGCGGAAGCCACCACAAGGG |
| ASCRP3007102 | 0.211414561 | 0.909237851 | 1.0486494 | down | hsa_circRNA_003900 | hsa_circ_0003900 | circBase | chr11 | - | 61094250 | 61097546 | exonic | NM_001923 | DDB1 | CTTGGAAACAGGAAAATGTCGAAGCTGAAGCTTCCATGGTGATCGCAGGGGGAGAGCAAG |
| ASCRP3007109 | 0.063786853 | 0.909237851 | 1.0877053 | down | hsa_circRNA_053882 | hsa_circ_0053882 | circBase | chr2 | + | 33002948 | 33012216 | exonic | NM_017735 | TTC27 | CTTGGACTTACGTGACAAATACAAAGATGTTCAGCTCGGGGTGTGGTTTTCTCTCGGTTG |
| ASCRP3007114 | 0.326845184 | 0.909237851 | 1.0800325 | down | hsa_circRNA_405025 | | 25070500 | chr12 | + | 69853296 | 69856305 | sense overlapping | ENST00000549261 | RP11-956E11.1 | CTTGGCATCTCTCCATAGTCACCATTTAGAAGGCTCCTGGGTTCTGTTCTATGTCATTTC |
| ASCRP3007116 | 0.488575487 | 0.91119147 | 1.1280859 | down | hsa_circRNA_011491 | hsa_circ_0011491 | circBase | chr1 | - | 34090650 | 34209207 | exonic | NM_052896 | CSMD2 | CTTGGCCCAATGGAATGTCTCAGCGCCCACGTGTGTGGTCTCCTGCTTCTTCAACTTCAC |
| ASCRP3007123 | 0.274217274 | 0.909237851 | 1.0271504 | down | hsa_circRNA_400770 | | 25242744 | chr11 | + | 66252643 | 66255478 | exonic | NM_005700 | DPP3 | CTTGGCTCAGGCGTTCCTGGTCTATGCCGCGGGTGTTTACTCCAACATGGGCAACTACAA |
| ASCRP3007124 | 0.072544448 | 0.909237851 | 1.3576328 | down | hsa_circRNA_084900 | hsa_circ_0084900 | circBase | chr8 | - | 95504879 | 95550574 | exonic | NM_015496 | KIAA1429 | CTTGGGAAGCACAAGCATGAGACCTTTATAACGTCAAGAGAGACATCTCCCCATACATTT |
| ASCRP3007130 | 0.28255662 | 0.909237851 | 1.1158998 | down | hsa_circRNA_092412 | hsa_circ_0000574 | circBase | chr14 | - | 105131084 | 105131167 | sense overlapping | ENST00000564585 | RP11-982M15.8 | CTTGGGTCTCCTTTTCCAGCACAGGTGCCTTCTCCCTCGGCAGGAGGGTGCGATGGTCTC |
| ASCRP3007132 | 0.888370858 | 0.979863126 | 1.0060809 | down | hsa_circRNA_403604 | | 25242744 | chr6 | - | 43527914 | 43541338 | exonic | NM_020750 | XPO5 | CTTGGTCAAGTTTTGTGAGGAGTTTAAAGAAAAGTGTCCTATCTGTGTCCCCTGTGGCTT |
| ASCRP3007133 | 0.48689372 | 0.91119147 | 1.0172414 | down | hsa_circRNA_104794 | hsa_circ_0087275 | circBase | chr9 | + | 80921229 | 80923499 | exonic | NM_021154 | PSAT1 | CTTGTACAACACGCCTCCATGTTTCAGAAATTCCAGATCCAAGCACCTGGAACCTCAACC |
| ASCRP3007138 | 0.923648087 | 0.983668077 | 1.0022581 | down | hsa_circRNA_103036 | hsa_circ_0060035 | circBase | chr20 | - | 33603810 | 33609159 | exonic | NM_015638 | TRPC4AP | CTTGTCCTCCATGGTCACAACCAGAACTGTGACTGTAGCCCGCCTCCATTGTGTTCCCTC |
| ASCRP3007139 | 0.890329896 | 0.979863126 | 1.0037554 | down | hsa_circRNA_001881 | hsa_circ_0001881 | circBase | chr9 | + | 111881957 | 111882086 | antisense | NM_032012 | TMEM245 | CTTGTCGAAGCGCAGCGCCAGCGCCGCGGTCCGCGGGGCCTCCAGGATGAAGTAGACCAG |
| ASCRP3007145 | 0.565056902 | 0.920978531 | 1.0207043 | down | hsa_circRNA_001256 | hsa_circ_0001256 | circBase | chr22 | - | 50689402 | 50689520 | intronic | ENST00000483222 | HDAC10 | CTTGTGTACCATGAGGACATGACGGCCACCCGGCTGCTCTGGGACGAGCAGTGGCAGGCA |
| ASCRP3007151 | 0.43700134 | 0.91119147 | 1.0405715 | down | hsa_circRNA_002134 | hsa_circ_0002134 | circBase | chr3 | + | 61608136 | 61609229 | intronic | ENST00000295874 | PTPRG | CTTGTTCAGGAGTGACAGATTGCAGTACGTATTTTAGGACCTGCCACCCATTTTGATTTG |
| ASCRP3007166 | 0.68074186 | 0.941064238 | 1.0493374 | down | hsa_circRNA_406517 | | 25070500 | chr4 | + | 109550361 | 109550978 | intronic | ENST00000394668 | RPL34 | CTTTACCCCACCCACTGCATATATAAACTTTTAACTCCTTTTTTTCTCCCACATAACCCC |
| ASCRP3007178 | 0.988449168 | 0.998148217 | 1.0005957 | down | hsa_circRNA_402721 | | 25242744 | chr22 | - | 32121523 | 32134897 | exonic | NM_173566 | PRR14L | CTTTCACATGTGGACCTCCCTGAAGATTTTCTAAGGAGCAAAGGGTGAATCATTTGATGT |
| ASCRP3007181 | 0.657211364 | 0.933363463 | 1.0150203 | down | hsa_circRNA_002048 | hsa_circ_0000727 | circBase | chr16 | - | 89484691 | 89497734 | exonic | NM_001256182 | ANKRD11 | CTTTCAGCTATGGAATCGCCCGTGCAGCCTGCCAGGACTCTTCCAGACACGGGATTTGTC |
| ASCRP3007188 | 0.626553527 | 0.929544944 | 1.1331724 | down | hsa_circRNA_104833 | hsa_circ_0002191 | circBase | chr9 | + | 97535283 | 97563284 | exonic | NM_032823 | C9orf3 | CTTTCCAAGTCTGGGGATGGCCAGGCCATGTGTTTATACTGTGGGATCTCCCATAAACAA |
| ASCRP3007193 | 0.952168172 | 0.992077624 | 1.0046991 | down | hsa_circRNA_404984 | | 25070500 | chr12 | + | 27867712 | 27878600 | exonic | ENST00000542199 | MRPS35 | CTTTCCTGTGCTCTCACCAACCTACATATTACCTGTCAACTTGATAGCGGAAAGAACACC |
| ASCRP3007200 | 0.038893525 | 0.909237851 | 1.2148238 | down | hsa_circRNA_001067 | hsa_circ_0000644 | circBase | chr15 | - | 81195758 | 81195954 | intronic | ENST00000558261 | RP11-351M8.1 | CTTTCTCCACTTTCTCCCCGTTTACCAGTCTAAGGGGTTGACACAGTGAAACTCCAACTC |
| ASCRP3007207 | 0.477739625 | 0.91119147 | 1.0228389 | down | hsa_circRNA_100055 | hsa_circ_0009964 | circBase | chr1 | + | 12335881 | 12338095 | exonic | NM_015378 | VPS13D | CTTTGAATCTTTGCATAGAGGTCAAGATAACTCCAGGAGGAAAAGTAGGGATGGGTCAGC |
| ASCRP3007214 | 0.065044639 | 0.909237851 | 1.0668742 | down | hsa_circRNA_407242 | | 25070500 | chr9 | - | 128002616 | 128002954 | intronic | ENST00000324460 | HSPA5 | CTTTGAGATGTCATTGTATCTGTGTTCGACCGGGTTTTTCTCATCCAGTTAGAGAACGGG |
| ASCRP3007219 | 0.00491458 | 0.879037174 | 1.1649935 | down | hsa_circRNA_101031 | hsa_circ_0007478 | circBase | chr12 | - | 27143382 | 27152609 | exonic | NM_016551 | TM7SF3 | CTTTGAGGCAGGAGAGGGTAGTTGTGCTTCCCTAGACTCTCCTTTCCAATTCCTCGGAAA |
| ASCRP3007224 | 0.57465677 | 0.922626849 | 1.0179976 | down | hsa_circRNA_058741 | hsa_circ_0058741 | circBase | chr2 | + | 234054804 | 234091145 | exonic | NM_005541 | INPP5D | CTTTGCCACATTTGAGGCAGGAGTCACTTCCCAGTTTGTCTCCAAGAACGCAGGTTCCTG |
| ASCRP3007241 | 0.920443722 | 0.98346125 | 1.0041835 | down | hsa_circRNA_406714 | | 25070500 | chr5 | - | 171785557 | 171785759 | intronic | ENST00000311601 | SH3PXD2B | CTTTGGTAGCAACCTATCCTCTGCTCACTGGGGAGGACCTGTGCAGTCATCGCCTATACT |
| ASCRP3007242 | 0.76874529 | 0.963602436 | 1.0084533 | down | hsa_circRNA_041698 | hsa_circ_0041698 | circBase | chr17 | - | 5298210 | 5302970 | exonic | NM_002532 | NUP88 | CTTTGTACGAAGCCATTGCCCTGCAGATCCCAAGTGTCCTTCAAGATATCACTGTACTCA |
| ASCRP3007256 | 0.410341827 | 0.91119147 | 1.0399856 | down | hsa_circRNA_403838 | | 25242744 | chr7 | + | 73969503 | 73972052 | exonic | NM_005685 | GTF2IRD1 | CTTTTCAATAAGAAATACGGCCCGAGCTGCTCACTGAGGGAGTCAAAGAGCCCATCATGG |
| ASCRP3007262 | 0.0193981 | 0.909237851 | 1.2195084 | down | hsa_circRNA_043898 | hsa_circ_0043898 | circBase | chr17 | - | 40879652 | 40882936 | exonic | NM_001991 | EZH1 | CTTTTCTCAAAAAGCTGGACACCTGTTCTGCTGTTGTGTCCTGCCATTCTCCTGAAGAAC |
| ASCRP3007268 | 0.771583711 | 0.96364966 | 1.0427765 | down | hsa_circRNA_056530 | hsa_circ_0056530 | circBase | chr2 | + | 135170439 | 135212192 | exonic | NM_002410 | MGAT5 | CTTTTCTTGTGTGATTGTTTGTTGGACTTGGGTTCCCTTACGAGGGCCCAGCTCCCCTGG |
| ASCRP3007278 | 0.524823175 | 0.916971445 | 1.0625593 | down | hsa_circRNA_100956 | hsa_circ_0000357 | circBase | chr11 | - | 116734383 | 116744352 | exonic | NM_025164 | SIK3 | GAAAAAATGGGCAACAACAGCAGCATCAAACAGCTGCAGCAGGAGCAGTCTCTCCTACAG |
| ASCRP3007280 | 0.190500819 | 0.909237851 | 1.1383447 | down | hsa_circRNA_027446 | hsa_circ_0027446 | circBase | chr12 | + | 66221780 | 66232349 | exonic | NM_003483 | HMGA2 | GAAAAACGGCCAAGAGGCAGACCTAGGAAATGGGAACCAACCGGTGAGCCCTCTCCTAAG |
| ASCRP3007284 | 0.858887077 | 0.97947761 | 1.006795 | down | hsa_circRNA_104109 | hsa_circ_0076413 | circBase | chr6 | + | 42819829 | 42821476 | exonic | NM_015349 | GLTSCR1L | GAAAAAGTAGTTGGATCATCTCCTGGCCATCCAGCTGTGCAGGCTCAGAAAAAATGTCTG |
| ASCRP3007288 | 0.214258774 | 0.909237851 | 1.0935076 | down | hsa_circRNA_101508 | hsa_circ_0007705 | circBase | chr15 | + | 44788577 | 44789336 | exonic | NM_016396 | CTDSPL2 | GAAAACAAGAAGCACACCGGAATTCTCCCTAGTTTTAGACTTGCACCAGTAACTCCAGAT |
| ASCRP3007289 | 0.386744317 | 0.91119147 | 1.0391682 | down | hsa_circRNA_102118 | hsa_circ_0044413 | circBase | chr17 | - | 47388673 | 47395345 | exonic | NM_014897 | ZNF652 | GAAAACACACACTGATAAGATGCTCCCAAGTCCCTTGCAGCTCTCTTCAGAAGAAAAATC |
| ASCRP3007293 | 0.175170378 | 0.909237851 | 1.1420465 | down | hsa_circRNA_007105 | hsa_circ_0007105 | circBase | chr18 | - | 57056124 | 57069951 | sense overlapping | TCONS_00026535 | XLOC_012858 | GAAAACTAAGGCTGAGAACATTGAAGTAGCACGATCATGGCAGAGCGTCTCCTGCAGTTC |
| ASCRP3007304 | 0.001205887 | 0.870215632 | 1.100389 | down | hsa_circRNA_001537 | hsa_circ_0001537 | circBase | chr5 | + | 137726701 | 137735701 | exonic | NM_016604 | KDM3B | GAAAAGCCGGCCACGCAGTGAATTCAAGAAATTCTATTCTGGCCTCTTCTGGATTTGGAG |
| ASCRP3007307 | 0.482497334 | 0.91119147 | 1.0913537 | down | hsa_circRNA_001717 | hsa_circ_0001717 | circBase | chr7 | + | 73752777 | 73753334 | exonic | NM_003388 | CLIP2 | GAAAAGGACCTGCGCCTGGGGGACCGCGTGCTGGCTCCCCACTGCACAAACAGTCATCTG |
| ASCRP3007309 | 0.116200559 | 0.909237851 | 1.0746614 | down | hsa_circRNA_102688 | hsa_circ_0006422 | circBase | chr2 | + | 36737125 | 36749456 | exonic | NM_016441 | CRIM1 | GAAAAGGCCAGTGTTGTCCCTACTGCATAGCCGAGGAACTATGTTCAGAACGTAAACAAG |
| ASCRP3007312 | 0.402559893 | 0.91119147 | 1.0432059 | down | hsa_circRNA_100606 | hsa_circ_0018529 | circBase | chr10 | - | 70246900 | 70248386 | exonic | NM_152707 | SLC25A16 | GAAAAGTGCCTGTGTTTCATTTTTTACTTTTGGTACCTTGAAGAGTGTTGGGCTTTCCCA |
| ASCRP3007314 | 0.3231223 | 0.909237851 | 1.0304023 | down | hsa_circRNA_406431 | | 25070500 | chr4 | + | 1343311 | 1349033 | exonic | NM_020894 | UVSSA | GAAAATACAAGGAGCTGGACATCGAGCCTGAGGGAGGGGAAAGGCGCAGGGTCTTCAGAG |
| ASCRP3007329 | 0.85224072 | 0.978900086 | 1.009705 | down | hsa_circRNA_045788 | hsa_circ_0045788 | circBase | chr17 | - | 74283272 | 74286158 | exonic | NM_032134 | QRICH2 | GAAAATTCTGTCTCTGAAGCCTCCCTTTACCTGCAGGACCAGTCAAAAGGACCATACCTC |
| ASCRP3007336 | 0.841046195 | 0.978181218 | 1.0122558 | down | hsa_circRNA_103066 | hsa_circ_0006332 | circBase | chr20 | + | 42331129 | 42333998 | exonic | NM_002466 | MYBL2 | GAAACATGCTGCGACCCTGATGCTTGGTGTGACCTGAGTAAATTTGACCTCCCTGAGGAA |
| ASCRP3007338 | 0.047064561 | 0.909237851 | 1.1269634 | down | hsa_circRNA_038054 | hsa_circ_0038054 | circBase | chr16 | + | 15758592 | 15761296 | exonic | NM_017668 | NDE1 | GAAACCATCAAGACACCATGCCACAAGGAGAGTGATCTCTTCCCCTGTTTTCACAATGGA |
| ASCRP3007354 | 0.60713148 | 0.929544944 | 1.0338043 | down | hsa_circRNA_100438 | hsa_circ_0006827 | circBase | chr1 | - | 207989273 | 207990759 | exonic | uc001hgr.2 | mir-29b-2 | GAAACTGGATGCAGCAAGGAAATAGGATTTAACCGCTCTCTGCCTCCCAGAGGACTCAGG |
| ASCRP3007358 | 0.183871973 | 0.909237851 | 1.0491895 | down | hsa_circRNA_008936 | hsa_circ_0008936 | circBase | chr1 | - | 10527246 | 10529395 | exonic | NM_004401 | DFFA | GAAAGAAGATCTGTCCAGCATCATCCTCCTATCAGAGGAGGACCTCCAGCCTGTGACATT |
| ASCRP3007361 | 0.355697017 | 0.91119147 | 1.1143463 | down | hsa_circRNA_007315 | hsa_circ_0007315 | circBase | chr3 | + | 110863745 | 110866331 | exonic | NM_001243288 | PVRL3 | GAAAGACCTATTTCAGATGTTCCATTTAAGCAGACCTCTTCCATAGCTGTAGCTGGAGCG |
| ASCRP3007367 | 0.998841021 | 0.99972787 | 1.0001754 | down | hsa_circRNA_076057 | hsa_circ_0076057 | circBase | chr6 | + | 34789429 | 34803241 | exonic | NM_017754 | UHRF1BP1 | GAAAGAGCCTGGCCCCTGAACCTGTGCAGGTTCACTAAGAATCTTTCCCCAGACAAAATC |
| ASCRP3007372 | 0.009953027 | 0.909237851 | 1.0744516 | down | hsa_circRNA_100209 | hsa_circ_0012300 | circBase | chr1 | - | 46521466 | 46546422 | exonic | NM_003629 | PIK3R3 | GAAAGATCCGAGATCAACACCTTGTCTCTTCCACCAAAGCCACCTAAGCCAATGACTTCA |
| ASCRP3007377 | 0.611633409 | 0.929544944 | 1.0241557 | down | hsa_circRNA_005272 | hsa_circ_0005272 | circBase | chr4 | + | 154191486 | 154197282 | exonic | NM_015271 | TRIM2 | GAAAGCCTCTCTCTTGCCCAAACCACGATGGGAATCAGCAGCGTGCAGGGTCAAAGACAG |
| ASCRP3007379 | 0.399469305 | 0.91119147 | 1.0370174 | down | hsa_circRNA_404862 | | 25070500 | chr11 | + | 20129328 | 20136114 | intronic | ENST00000311043 | NAV2 | GAAAGCTGCAAAACTGGGGCTACGAGAGAGATCTGGGCCACTTCCAGACTGTGAGAAGAC |
| ASCRP3007382 | 0.7915676 | 0.967847019 | 1.013406 | down | hsa_circRNA_101591 | hsa_circ_0036282 | circBase | chr15 | + | 74865202 | 74865545 | exonic | NM_006465 | ARID3B | GAAAGGAAAGAGTTCCTGGATGACCTCTTCGTCTTTATGCAGAAGAGGGAATGGTGGTTT |
| ASCRP3007386 | 0.613063606 | 0.929544944 | 1.0284012 | down | hsa_circRNA_403042 | | 25242744 | chr3 | + | 184654033 | 184682345 | exonic | NM_015303 | VPS8 | GAAAGGAGATATTCATGGTGCCTTCCTAATAATGTTAGAGGAACTCGTGTCCCTGAAGCC |
| ASCRP3007390 | 0.750569847 | 0.957214076 | 1.00749 | down | hsa_circRNA_092495 | hsa_circ_0001090 | circBase | chr2 | - | 201719338 | 201719809 | exonic | NM_004071 | CLK1 | GAAAGGATTCTTGGACCTCTACCAAAACATATGATACAGAAAACCAGCCCTAGGGTGGTC |
| ASCRP3007391 | 0.200029891 | 0.909237851 | 1.0417879 | down | hsa_circRNA_101768 | hsa_circ_0038649 | circBase | chr16 | + | 24043456 | 24135302 | exonic | NM_002738 | PRKCB | GAAAGGCAGCTTTGGCAAGGACCCCCGCAGCAAACACAAGTTTAAGATCCACACGTACTC |
| ASCRP3007393 | 0.137508637 | 0.909237851 | 1.0508406 | down | hsa_circRNA_104355 | hsa_circ_0079985 | circBase | chr7 | - | 44012228 | 44012922 | exonic | NR_003655 | POLR2J4 | GAAAGGTCTGCCTTACAAGGGACACCATAGCTTCTCACCCTAAGGGACAGCCACAGTGTG |
| ASCRP3007395 | 0.061842708 | 0.909237851 | 1.0559293 | down | hsa_circRNA_068472 | hsa_circ_0068472 | circBase | chr3 | - | 186509513 | 186510736 | exonic | NM_002916 | RFC4 | GAAAGTGATTACAGACATTGCCGGGTGGGAAGCCGTGTCCGCCTTTTAAGATTGTGATTC |
| ASCRP3007401 | 0.948017237 | 0.992077624 | 1.002561 | down | hsa_circRNA_404592 | | 25070500 | chr1 | - | 155721557 | 155730432 | exonic | NM_001282856 | GON4L | GAAATAAGACCCCTGCTGAGGTGCGAGAAGCCCTACAACATATCCCTGGCAAGTATGAAG |
| ASCRP3007411 | 0.667361186 | 0.937594692 | 1.0434265 | down | hsa_circRNA_102924 | hsa_circ_0008365 | circBase | chr2 | - | 224856519 | 224866639 | exonic | NM_006216 | SERPINE2 | GAAATCCTATCAAGTGCCAATGCTGGCCCAGCTCTCCGTGTTCCGGTGTGGTCGTCCTTG |
| ASCRP3007419 | 0.903098207 | 0.981257955 | 1.0053559 | down | hsa_circRNA_006604 | hsa_circ_0006604 | circBase | chr2 | + | 232153372 | 232156156 | exonic | ENST00000428662 | ARMC9 | GAAATGATCCGCCAGATAGAATTCATCATCAAGCAGCTAAATTCCGGTCCCCTGAAGGAG |
| ASCRP3007428 | 0.356587424 | 0.91119147 | 1.052542 | down | hsa_circRNA_023828 | hsa_circ_0023828 | circBase | chr11 | - | 78433712 | 78926954 | exonic | NM_001098816 | TENM4 | GAAATGTCACCAACATCCTAGAGCTGAGAAAAGATCCCGCACCATCCTCCAGGATCCAAT |
| ASCRP3007431 | 0.657127927 | 0.933363463 | 1.0447659 | down | hsa_circRNA_000129 | hsa_circ_0000129 | circBase | chr1 | - | 151145974 | 151149507 | exonic | ENST00000491094 | VPS72 | GAAATGTGTGACATTGCAGACTTGATCCTGCTCCCTCGGTGTCTGCATTGACTCCTCATG |
| ASCRP3007435 | 0.902568137 | 0.981257955 | 1.0077825 | down | hsa_circRNA_102881 | hsa_circ_0057582 | circBase | chr2 | - | 197135916 | 197172809 | exonic | NM_020760 | HECW2 | GAAATTCTACAGGAGCGTCAACCTGATCTTACCAGGAATCACTCACTCAGACTGGGAGGC |
| ASCRP3007440 | 0.139213755 | 0.909237851 | 1.1489233 | down | hsa_circRNA_104248 | hsa_circ_0002073 | circBase | chr6 | + | 158870027 | 158873300 | exonic | NM_020245 | TULP4 | GAACAACTACGATGACTTGTCTCCCACGGTCATCCGCTCAGGGCTGAAAGGTGCTGTTTG |
| ASCRP3007443 | 0.630525561 | 0.929544944 | 1.017413 | down | hsa_circRNA_101460 | hsa_circ_0034067 | circBase | chr15 | + | 22925975 | 22926065 | exonic | NM_014608 | CYFIP1 | GAACAAGCCACCGTCCACTCTAGCATGCCAAATTTCAACACTAACTTTGAAGACAGAAAT |
| ASCRP3007454 | 0.201413399 | 0.909237851 | 1.0397832 | down | hsa_circRNA_102408 | hsa_circ_0048430 | circBase | chr19 | + | 2790418 | 2811732 | exonic | NM_003249 | THOP1 | GAACAGCAAGCCTGTGCAGGAGACATGGCGGACGCAGCATCTCCGTGCTCTGTGGTAAAC |
| ASCRP3007458 | 0.508059972 | 0.915689485 | 1.0236769 | down | hsa_circRNA_102133 | hsa_circ_0006508 | circBase | chr17 | + | 57808781 | 57816308 | exonic | NM_030938 | VMP1 | GAACAGGGCTGCACACCTTTCTGCTTTATCTGCTCCTCAAGAGTTACTGATCTATGAAAT |
| ASCRP3007462 | 0.628359464 | 0.929544944 | 1.0318096 | down | hsa_circRNA_101066 | hsa_circ_0026337 | circBase | chr12 | + | 52180325 | 52188425 | exonic | NM_014191 | SCN8A | GAACATCTTCGACTTCGTGGTAGTCATCCTCTCCATTGTGGGTGGTGGTGAATGCCTTGG |
| ASCRP3007466 | 0.909783191 | 0.983376499 | 1.0060944 | down | hsa_circRNA_401666 | | 25242744 | chr16 | + | 83940591 | 83945972 | exonic | NM_012213 | MLYCD | GAACATTCCTCATAAAGCGAGTCGTCAAGGAGTTGCAGGAAATGAATGGGGTGCTGAAAG |
| ASCRP3007476 | 0.087433872 | 0.909237851 | 1.1134647 | down | hsa_circRNA_080346 | hsa_circ_0080346 | circBase | chr7 | + | 65817491 | 65821858 | exonic | NM_003596 | TPST1 | GAACCAGGAGCCTCTTCCATACATGAGGTCTATAAGGGAGAATTCCAACTACCTGACTTT |
| ASCRP3007479 | 0.14983466 | 0.909237851 | 1.1603268 | down | hsa_circRNA_103047 | hsa_circ_0060144 | circBase | chr20 | + | 34389412 | 34459751 | exonic | NM_016436 | PHF20 | GAACCGACTTCTCCCCTTGTGGAATTACAAGAGATTTCGACTGTGGAAGGAGAATAAAGG |
| ASCRP3007484 | 0.2427005 | 0.909237851 | 1.0338101 | down | hsa_circRNA_101011 | hsa_circ_0007481 | circBase | chr12 | - | 11126253 | 11199787 | exonic | NR_037918 | PRH1-PRR4 | GAACCTGCTGTATCTACTCAAGAGTGGAAGTTTTCACAGGTTCTCCAGCATTTGGCTCAA |
| ASCRP3007485 | 0.827150548 | 0.977459664 | 1.0242413 | down | hsa_circRNA_105001 | hsa_circ_0005477 | circBase | chrX | - | 54481880 | 54483000 | exonic | NM_004463 | FGD1 | GAACCTTCCTGGTGTCAGGAAAGCAGCGCTCCCTCGAGCTCCAGGCCAGAGTCTCTGGAG |
| ASCRP3007501 | 0.973817729 | 0.996425773 | 1.0012677 | down | hsa_circRNA_404013 | | 25242744 | chr8 | - | 37704371 | 37704693 | exonic | NM_018310 | BRF2 | GAACTGGTGAAGACCTATTGCAGCAGGTCTCCGGCGAGTGAGAGACCTTTGTCGAGTTCT |
| ASCRP3007510 | 0.114087244 | 0.909237851 | 1.1437166 | down | hsa_circRNA_103633 | hsa_circ_0069681 | circBase | chr4 | - | 48552608 | 48553595 | exonic | NM_015030 | FRYL | GAAGAAAAAAGAACTACTTCCAGTAGCAATACAATGGTAGCTCCCACAGATGGCAATCCT |
| ASCRP3007515 | 0.080113438 | 0.909237851 | 1.0616119 | down | hsa_circRNA_101879 | hsa_circ_0040507 | circBase | chr16 | - | 74926381 | 74937998 | exonic | NM_030581 | WDR59 | GAAGAAAGGATCTTGTCCAGATCTCTCTCAGCCTTGTCTGCTTATCACACTGGCTTGATC |
| ASCRP3007516 | 0.731243591 | 0.952599775 | 1.0324768 | down | hsa_circRNA_104939 | hsa_circ_0089131 | circBase | chr9 | + | 133738149 | 133738422 | exonic | NM_005157 | ABL1 | GAAGAAATACAGCCTGACGGTGGCCGTGAAGACCTTGAAGCTCTACGTCTCCTCCGAGAG |
| ASCRP3007520 | 0.255217367 | 0.909237851 | 1.0869552 | down | hsa_circRNA_004611 | hsa_circ_0004611 | circBase | chr16 | - | 8997124 | 8998422 | exonic | NM_003470 | USP7 | GAAGAACTCCTCGCTTGCTGAGTTTGTTCAGAGCCTCTCTCAGACCATGGTGAAGTTTTA |
| ASCRP3007528 | 0.61164556 | 0.929544944 | 1.0319579 | down | hsa_circRNA_006998 | hsa_circ_0006998 | circBase | chrY | - | 2111063 | 2111271 | exonic | NM_145177 | DHRSX | GAAGAAGCTTCTCGGCTGGTTGCTTTTCAAGTGCCTGCTACTCACCCCACGCAGCCTACG |
| ASCRP3007543 | 0.07175758 | 0.909237851 | 1.0707691 | down | hsa_circRNA_100478 | hsa_circ_0016873 | circBase | chr1 | - | 230979099 | 230979642 | exonic | NM_032800 | C1orf198 | GAAGACACCCTGTTCTCGGAACCCAAGTTTGCACAGAGTCAGATGGAGTTCAGTATCTCC |
| ASCRP3007545 | 0.282932545 | 0.909237851 | 1.0488052 | down | hsa_circRNA_103637 | hsa_circ_0007928 | circBase | chr4 | + | 52729602 | 52752804 | exonic | NM_015115 | DCUN1D4 | GAAGACATTGGTGTTGAACCAGAAAACATTTTCAGCTGAACTCTCATCTCTCAACACTGG |
| ASCRP3007554 | 0.833978763 | 0.978181218 | 1.0081894 | down | hsa_circRNA_102393 | hsa_circ_0048025 | circBase | chr18 | + | 77193578 | 77227582 | exonic | NM_006162 | NFATC1 | GAAGAGAAAGCGAAGCCAGTACCAGCGTTTCACCTACCTTCCCGCCAACGCCCGACCCTG |
| ASCRP3007556 | 0.766765116 | 0.962339655 | 1.0170322 | down | hsa_circRNA_039219 | hsa_circ_0039219 | circBase | chr16 | + | 46934593 | 46950619 | exonic | NM_133443 | GPT2 | GAAGAGAAGCTCTTTCTCCTGGCTGATGAGGTGATGGCACTATGCACCTACCCAAACCTG |
| ASCRP3007562 | 0.383343541 | 0.91119147 | 1.09603 | down | hsa_circRNA_402177 | | 25242744 | chr2 | - | 25650403 | 25656864 | exonic | NM_021907 | DTNB | GAAGAGCTGATGAAGTTGCTGAAGACTCGTCCTCCCACTGACTTGAGCTTTAACTTTGAT |
| ASCRP3007563 | 0.114829191 | 0.909237851 | 1.0401215 | down | hsa_circRNA_001350 | hsa_circ_0000253 | circBase | chr10 | - | 97999787 | 97999925 | intronic | NR_047681 | BLNK | GAAGAGCTTAGGTGGTGTGGGAAGATCTGTCTGAGAAGAAACAAGAAAGCAAACAGGTAA |
| ASCRP3007569 | 0.02750119 | 0.909237851 | 1.0961688 | down | hsa_circRNA_405509 | | 25070500 | chr16 | + | 89289564 | 89291210 | exonic | NM_001201407 | ZNF778 | GAAGAGGAGTTGAGGGCAGGGCGGAGAGCAGTTCTCCAAGGACGCGGTGACCTTTGACGA |
| ASCRP3007572 | 0.026140847 | 0.909237851 | 1.0626063 | down | hsa_circRNA_000954 | hsa_circ_0000954 | circBase | chr19 | - | 54377094 | 54377343 | antisense | NM_138373 | MYADM | GAAGAGGGCCGCATAGCAGGCGAAGATGATGCAGGCAACGAAGGTCTCCAGCACCTTCAG |
| ASCRP3007573 | 0.503052261 | 0.914489113 | 1.0550429 | down | hsa_circRNA_404830 | | 25070500 | chr11 | + | 830718 | 830831 | intronic | ENST00000450448 | EFCAB4A | GAAGAGGGGGCCACCCGGCCCCTGCCCTGTCCCCACGGTCACCCGTGCACTCTCCCCCTG |
| ASCRP3007575 | 0.11967811 | 0.909237851 | 1.0625227 | down | hsa_circRNA_101353 | hsa_circ_0031897 | circBase | chr14 | + | 51710573 | 51713938 | exonic | NM_030755 | TMX1 | GAAGAGTATTGAGCCCGTTTCATCATGGTTTGGTCCAGGTTCTGTTCTTTATGCCCCGTG |
| ASCRP3007576 | 0.374888945 | 0.91119147 | 1.0297077 | down | hsa_circRNA_103980 | hsa_circ_0006528 | circBase | chr5 | - | 145197456 | 145205763 | exonic | NM_138492 | PRELID2 | GAAGAGTCTGTCTTCCGGGAAAGTATGGAAAACCCAAATTGGTTTCCACGCTTTGAGAAG |
| ASCRP3007577 | 0.80024348 | 0.972258433 | 1.0071857 | down | hsa_circRNA_060011 | hsa_circ_0060011 | circBase | chr20 | + | 33501898 | 33509669 | exonic | NM_018677 | ACSS2 | GAAGAGTTGGAAGGTGAAGCTGAAGGTTATCTGATGCCTTCTACAGGGGGGAAAAGCTTG |
| ASCRP3007579 | 0.541935364 | 0.919549314 | 1.0618227 | down | hsa_circRNA_104086 | hsa_circ_0003738 | circBase | chr6 | + | 25420341 | 25435832 | exonic | NM_017640 | LRRC16A | GAAGATATTTCCTGGCCTCTCTCCAGTGTCCTTACATCATGCCGAGCCTTCCTTGTAACA |
| ASCRP3007580 | 0.564991966 | 0.920978531 | 1.0217888 | down | hsa_circRNA_055264 | hsa_circ_0055264 | circBase | chr2 | + | 74140611 | 74143892 | exonic | NM_001615 | ACTG2 | GAAGATCAAGGCATCGTCCTGGATTCAGGTGATGGCGTCACCCACAATGTCCCCATCTAT |
| ASCRP3007583 | 0.852522439 | 0.978900086 | 1.0065308 | down | hsa_circRNA_101177 | hsa_circ_0006390 | circBase | chr12 | + | 121000747 | 121001746 | exonic | NM_014868 | RNF10 | GAAGATCTCAGCAACTGTGGTGGAGATTGCTGGCTACTCCATGTCTGAGACTCGGGAAGA |
| ASCRP3007587 | 0.708175721 | 0.945024804 | 1.0954837 | down | hsa_circRNA_100236 | hsa_circ_0012634 | circBase | chr1 | - | 54506428 | 54509198 | exonic | NM_004872 | TMEM59 | GAAGATGGAGAAAGTGATGGCTTTTTAAGATGCCTCTCTCTCTTATGTCCCTGATGCCAA |
| ASCRP3007591 | 0.146020933 | 0.909237851 | 1.073016 | down | hsa_circRNA_015962 | hsa_circ_0015962 | circBase | chr1 | + | 201966446 | 201969143 | exonic | NM_020216 | RNPEP | GAAGATTGAACCAGGTATGACTTGCTCTTCATGCCACCGTCCTTTCCATTTGGAGGAATG |
| ASCRP3007596 | 0.023519935 | 0.909237851 | 1.1763154 | down | hsa_circRNA_001175 | hsa_circ_0001658 | circBase | chr6 | + | 157357968 | 157406039 | exonic | uc003qqq.1 | ARID1B | GAAGCAACCAGTCTCGATCTGGCCCAATCTCTCCTGCAAGTATCCCAGGTTGAAGTCTTG |
| ASCRP3007608 | 0.188320424 | 0.909237851 | 1.1977887 | down | hsa_circRNA_404768 | | 25070500 | chr10 | + | 70411602 | 70432802 | exonic | NM_030625 | TET1 | GAAGCCCAAGCCCCAGAAGATTTAGAATTGATCCAAGCTCTCCCTTACATATCGAGTTAT |
| ASCRP3007609 | 0.887096978 | 0.979863126 | 1.0048868 | down | hsa_circRNA_000875 | hsa_circ_0000875 | circBase | chr19 | - | 4053968 | 4055245 | exonic | NM_015898 | ZBTB7A | GAAGCCCTACGAGTGCAACATCTGCAAGGTCCGCTTCACCAGGTCTCGGCGCGGAAGATG |
| ASCRP3007615 | 0.526996498 | 0.916971445 | 1.0255336 | down | hsa_circRNA_100992 | hsa_circ_0024866 | circBase | chr11 | - | 129827640 | 129830977 | exonic | NM_020228 | PRDM10 | GAAGCTGCACAGGCATGCTGCAGACAGACCTTTCCGTCCTTCATGAACACACGTGTGGTA |
| ASCRP3007628 | 0.707960783 | 0.945024804 | 1.0064566 | down | hsa_circRNA_092378 | hsa_circ_0000201 | circBase | chr1 | - | 247095061 | 247095200 | sense overlapping | ENST00000478568 | AHCTF1 | GAAGGCACTCTCGGGGGCGTGTGGCTCTGAGGTGGCCGGGGAGGCGAGCAGGCAGCGTTG |
| ASCRP3007631 | 0.455633514 | 0.91119147 | 1.0397374 | down | hsa_circRNA_003792 | hsa_circ_0003792 | circBase | chr12 | + | 2595269 | 2622150 | exonic | NM_000719 | CACNA1C | GAAGGCATGGATGAGGAGAAGCCCCGAAACAGTCTCCAGGTGGTCCTGAATTCCATCATC |
| ASCRP3007634 | 0.351110645 | 0.91119147 | 1.0281908 | down | hsa_circRNA_000775 | hsa_circ_0001928 | circBase | chrX | + | 73071909 | 73072064 | sense overlapping | TCONS_00017433 | XLOC_008015 | GAAGGGAATCAGCAGGTATCCGAGGCCCCGATGGGCAAGGAAAAATAAAAAAAAAAAAAG |
| ASCRP3007641 | 0.63657628 | 0.929544944 | 1.0581976 | down | hsa_circRNA_401725 | | 25242744 | chr17 | - | 17124850 | 17127457 | exonic | NM_144997 | FLCN | GAAGGTGCTCCGACCGAGGATACCTTGGTCCAGATGGAGAAGCTCGCTGGTCTGCCCTGG |
| ASCRP3007650 | 0.216663181 | 0.909237851 | 1.0604037 | down | hsa_circRNA_404848 | | 25070500 | chr11 | + | 12183625 | 12183966 | exonic | NM_014632 | MICAL2 | GAAGTCGTGCACGAACACCAAGGTGTGACGTTTCTCCAGATACTTCATGCTGTTCACCTG |
| ASCRP3007656 | 0.636304788 | 0.929544944 | 1.0672382 | down | hsa_circRNA_403715 | | 25242744 | chr6 | + | 138528178 | 138531178 | exonic | NM_020340 | ARFGEF3 | GAAGTGATGAAGGGAGAAATGCCTGCTGCCTCTCCAGTTGGCTTTGGAATCCAAGAATGT |
| ASCRP3007658 | 0.078072758 | 0.909237851 | 1.0991891 | down | hsa_circRNA_076039 | hsa_circ_0076039 | circBase | chr6 | - | 34024320 | 34101636 | exonic | NM_000841 | GRM4 | GAAGTGCACCAGTTGGTAGGAGGAGAGGATTGGAGCTGTTTTCTCCTTGATGCCAAGATA |
| ASCRP3007661 | 0.606324563 | 0.929544944 | 1.104516 | down | hsa_circRNA_005073 | hsa_circ_0005073 | circBase | chr15 | - | 73052747 | 73072663 | sense overlapping | NM_031284 | ADPGK | GAAGTGGATGAGTTCCACCTCATTTTAGAGTATCAAGCAGGGGCTCTTCCCTGCTATTCA |
| ASCRP3007663 | 0.628370918 | 0.929544944 | 1.0329053 | down | hsa_circRNA_001359 | hsa_circ_0000157 | circBase | chr1 | - | 169663839 | 169664181 | intronic | ENST00000236147 | SELL | GAAGTGGGGCAGGGAGAGTATGGGAGCCAGAGGAACATCATTATCTAATAAGTCACCTAA |
| ASCRP3007667 | 0.384103618 | 0.91119147 | 1.0357401 | down | hsa_circRNA_074491 | hsa_circ_0074491 | circBase | chr5 | + | 149215825 | 149221940 | exonic | NM_133263 | PPARGC1B | GAAGTGTTTGGTGAGATTGAGGAGTGCGAGGTGCTGACAAGAAATAGGAGGACTCACCCC |
| ASCRP3007677 | 0.497886311 | 0.913224439 | 1.0332111 | down | hsa_circRNA_011206 | hsa_circ_0011206 | circBase | chr1 | - | 31205314 | 31230683 | exonic | NM_006762 | LAPTM5 | GAAGTTTCAATAAAGCAGCAACAAGCTTCTGGAGGGCAGCCAGCAGCTTCCCCTTCTCTG |
| ASCRP3007679 | 0.081181259 | 0.909237851 | 1.075613 | down | hsa_circRNA_076734 | hsa_circ_0076734 | circBase | chr6 | - | 52128811 | 52146973 | exonic | NM_002388 | MCM3 | GAATAAACTTAGCCATTTCGTACACATGGTGTTCTCTAGTTCGTCCCAAAGTCGTCCGCA |
| ASCRP3007680 | 0.300867871 | 0.909237851 | 1.1082525 | down | hsa_circRNA_080289 | hsa_circ_0080289 | circBase | chr7 | - | 56169265 | 56174187 | exonic | NM_016139 | CHCHD2 | GAATAAAGTCATTTTCTTCCAACCATCTTCCGGTCTCCTCAGAAGTCGCTTAGCTCTTCG |
| ASCRP3007684 | 0.29495528 | 0.909237851 | 1.0247114 | down | hsa_circRNA_400428 | | 25242744 | chr1 | - | 229675202 | 229685181 | exonic | NM_012089 | ABCB10 | GAATAAGCATTGGAGCTGCGGTTGGATTTCTCACGATGTCCAGTGTTATCTCCATGTCTG |
| ASCRP3007689 | 0.544020081 | 0.919549314 | 1.0404348 | down | hsa_circRNA_010906 | hsa_circ_0010906 | circBase | chr1 | - | 24495906 | 24507344 | exonic | NM_170743 | IFNLR1 | GAATACCTGGATTACCTTTTTGAAGGGAGGCCCCGTCTGGCCCCTCCCCAGAATGTGACG |
| ASCRP3007692 | 0.346785684 | 0.91119147 | 1.0724249 | down | hsa_circRNA_100370 | hsa_circ_0015002 | circBase | chr1 | + | 161293403 | 161298287 | exonic | NM_003001 | SDHC | GAATATAGGTTCAAACCGTCCTCTGTCTCCCCACATTACTATCTACAGACACGTTGGTCG |
| ASCRP3007696 | 0.571669876 | 0.922333971 | 1.0258443 | down | hsa_circRNA_100519 | hsa_circ_0017412 | circBase | chr10 | - | 909682 | 910210 | exonic | NM_015155 | LARP4B | GAATATGACTCTCTGCCTGAAAATAGCGAGACAGGTACCAGCAACTAAGGTTTCAGAGCT |
| ASCRP3007707 | 0.59434676 | 0.929544944 | 1.0147237 | down | hsa_circRNA_060491 | hsa_circ_0060491 | circBase | chr20 | - | 43139929 | 43142681 | exonic | NM_006811 | SERINC3 | GAATCATGGTTGGCTCTTTCTACATCCCTGGGGGCTATTTCAGCTCAGGTTCCATGCCTC |
| ASCRP3007709 | 0.072357255 | 0.909237851 | 1.1061968 | down | hsa_circRNA_104506 | hsa_circ_0082628 | circBase | chr7 | - | 138761031 | 138764989 | exonic | NM_020119 | ZC3HAV1 | GAATCCACCTCTCCTTCTTCACATCGTAGAAACATGGCATATAGGGCTAGAAGCAAGAGT |
| ASCRP3007720 | 0.840598578 | 0.978181218 | 1.0184468 | down | hsa_circRNA_041586 | hsa_circ_0041586 | circBase | chr17 | - | 4585796 | 4586247 | exonic | NM_014389 | PELP1 | GAATGAAGGCTTGTATGACCTATTTCCCTCGGGCTTGTGGTTCTCTCAAAACCCAGGACC |
| ASCRP3007741 | 0.685801823 | 0.942626653 | 1.0232773 | down | hsa_circRNA_102091 | hsa_circ_0044158 | circBase | chr17 | - | 43197691 | 43198739 | exonic | NM_133373 | PLCD3 | GAATGGTCAACGTGGACATGAACGACATGTACGCCTACCTCCTCTTCAAGGCCTGACGGA |
| ASCRP3007758 | 0.554659549 | 0.919867482 | 1.0209669 | down | hsa_circRNA_070294 | hsa_circ_0070294 | circBase | chr4 | - | 85617884 | 85724620 | exonic | NM_014991 | WDFY3 | GAATTCATCAGAGTCCATCGTGAGGCATGCATATCATTCTGTTTCAACTCCCCCTGTTTA |
| ASCRP3007762 | 0.238558735 | 0.909237851 | 1.0692938 | down | hsa_circRNA_043247 | hsa_circ_0043247 | circBase | chr17 | - | 35578644 | 35601650 | exonic | NM_198834 | ACACA | GAATTCCAGTTCATGCTGCCCACATCTCATCCAAACAGGCTGAACTTCACACAGGTAGTC |
| ASCRP3007766 | 0.293268316 | 0.909237851 | 1.0661165 | down | hsa_circRNA_101571 | hsa_circ_0036005 | circBase | chr15 | + | 67004005 | 67008836 | exonic | NM_005585 | SMAD6 | GAATTCTCAGAATCTCCGCCACCTCCCTACTCTCGGCTGTCTCCTCGCGACGAGTACAAG |
| ASCRP3007767 | 0.615435481 | 0.929544944 | 1.0796071 | down | hsa_circRNA_104728 | hsa_circ_0086242 | circBase | chr9 | - | 3330258 | 3395596 | exonic | NM_002919 | RFX3 | GAATTCTGGTCACTCAGTGACACACACAACTCGGGCCTCCCCAGCGACAAGACCATCATG |
| ASCRP3007779 | 0.101168211 | 0.909237851 | 1.0775939 | down | hsa_circRNA_003447 | hsa_circ_0003447 | circBase | chr7 | - | 140285388 | 140305606 | exonic | uc003vvx.3 | DENND2A | GAATTTTCCTGCTTCTCCCACCTCTTCCATCCCTGACACACTCACCAAGGTGACCTGGAA |
| ASCRP3007784 | 0.45081174 | 0.91119147 | 1.0896289 | down | hsa_circRNA_004988 | hsa_circ_0004988 | circBase | chr11 | - | 59406520 | 59410508 | exonic | NM_152716 | PATL1 | GACAAAACTGCAGTGCTGCCATGCTTACTGAGTCCCTTCTCTCTCCTTCTCTATCATCTT |
| ASCRP3007786 | 0.747239135 | 0.95565436 | 1.050489 | down | hsa_circRNA_102605 | hsa_circ_0052455 | circBase | chr2 | - | 1890310 | 1896059 | exonic | NM_015025 | MYT1L | GACAAAAGCATTCGAAGTATGCTGGCCACCAGCTCCCAAGAACTCAACGAAGCGGTACTG |
| ASCRP3007788 | 0.275911446 | 0.909237851 | 1.0806593 | down | hsa_circRNA_102784 | hsa_circ_0055734 | circBase | chr2 | + | 97818240 | 97820478 | exonic | NM_001164315 | ANKRD36 | GACAAAGGATAAGTTTGCTTTGGAATCTGAGTGCTTCCTCCTGTTGAAGAGGCTGTTGAC |
| ASCRP3007805 | 0.209262005 | 0.909237851 | 1.0692133 | down | hsa_circRNA_100084 | hsa_circ_0007991 | circBase | chr1 | - | 21329205 | 21415706 | exonic | NM_001198801 | EIF4G3 | GACAATGAATTCACAACCTCAAACCCGTTCTCCGAATTCCTAGAGGACCTGTGCAACAAC |
| ASCRP3007814 | 0.058383155 | 0.909237851 | 1.0895209 | down | hsa_circRNA_402755 | | 25242744 | chr22 | + | 44322814 | 44333152 | exonic | NM_025225 | PNPLA3 | GACACCCTCTCGCCCAGGCTCGCTACAGAGCAGACTCTGCAGGTCCTCTCAGATCTTGTG |
| ASCRP3007822 | 0.084716681 | 0.909237851 | 1.0825572 | down | hsa_circRNA_008721 | hsa_circ_0008721 | circBase | chr18 | - | 67602967 | 67614669 | intronic | ENST00000581982 | CD226 | GACACTGATTCTCCTGTGACCTCAGAACATTCTAACCAGCCTTTCAAACAGTTTCCAGAG |
| ASCRP3007831 | 0.095592016 | 0.909237851 | 1.169589 | down | hsa_circRNA_102594 | hsa_circ_0052012 | circBase | chr19 | + | 50902107 | 50902741 | exonic | NM_002691 | POLD1 | GACAGAGCCCCTCATCTTCCAACAGTTGGAGATTGACCATTATGTGGGATGGATGGCAAG |
| ASCRP3007832 | 0.052480453 | 0.909237851 | 1.1056587 | down | hsa_circRNA_021493 | hsa_circ_0021493 | circBase | chr11 | + | 20083852 | 20089944 | exonic | NM_145117 | NAV2 | GACAGCAAATTTCATGGATCCTCACTCTCCTTGGTTTCCAGCACATCGTCAGTTTATTCT |
| ASCRP3007836 | 0.320659653 | 0.909237851 | 1.0686609 | down | hsa_circRNA_101478 | hsa_circ_0008946 | circBase | chr15 | + | 40477365 | 40477843 | exonic | NM_001211 | BUB1B | GACAGCCAGTTATCTCCAAGCCAGAACAGAGGACTCCAAAATCCATTTCCTCAACAGATG |
| ASCRP3007839 | 0.792017559 | 0.967847019 | 1.0118084 | down | hsa_circRNA_102403 | hsa_circ_0048234 | circBase | chr19 | + | 1417498 | 1432689 | exonic | NM_018959 | DAZAP1 | GACAGCTCAGCCAGACTTCCCCTATGGTCAGTATGGAAGCTCTTCGTGGGCGGTCTTGAC |
| ASCRP3007851 | 0.101638599 | 0.909237851 | 1.0915023 | down | hsa_circRNA_001416 | hsa_circ_0001338 | circBase | chr3 | - | 128824688 | 128825122 | intronic | NM_001204888 | RAB43 | GACATAGGAACCAGGAGCCAAGTGGTAGCCCGAGGTCCCTCTCTAATATGAAGAAGCAAG |
| ASCRP3007853 | 0.503237929 | 0.914489113 | 1.0624541 | down | hsa_circRNA_002015 | hsa_circ_0002015 | circBase | chr17 | - | 53478829 | 53481229 | exonic | NM_012329 | MMD | GACATCAATGGTTAAATCTTCGTGAACTTGGACCCCTGGCATCTCATATGCGTTGGTTTA |
| ASCRP3007874 | 0.659742821 | 0.93421067 | 1.0802506 | down | hsa_circRNA_025249 | hsa_circ_0025249 | circBase | chr12 | - | 6859878 | 6860842 | exonic | NM_005439 | MLF2 | GACATGTTTGGGATGATGAATGACATGATTGGAAACATGGCTGGAGCTGTCTCCCCCTTT |
| ASCRP3007875 | 0.556239874 | 0.920978531 | 1.0371145 | down | hsa_circRNA_056734 | hsa_circ_0056734 | circBase | chr2 | + | 153399252 | 153417549 | exonic | NM_052905 | FMNL2 | GACATTCTGCACTGCGGAACGATTCCAGGTGAAGAATCCTCCCCATACATACATTCAAAA |
| ASCRP3007878 | 0.629666871 | 0.929544944 | 1.0324975 | down | hsa_circRNA_102599 | hsa_circ_0052166 | circBase | chr19 | + | 55420603 | 55421425 | exonic | NM_004829 | NCR1 | GACATTGAGAACACCAGCCTTGCACCTGAAGACCCCACCTTTCCTGAAATGTATGACACA |
| ASCRP3007881 | 0.439843122 | 0.91119147 | 1.0346448 | down | hsa_circRNA_103217 | hsa_circ_0063266 | circBase | chr22 | + | 38270376 | 38272018 | exonic | NM_016091 | EIF3L | GACCAAGAGCATGTTCCAGAGGACCACGTACAAGTATGAGATGGTGACCCTGAGAGTGTG |
| ASCRP3007882 | 0.086659997 | 0.909237851 | 1.0444973 | down | hsa_circRNA_062191 | hsa_circ_0062191 | circBase | chr22 | + | 18609120 | 18609801 | exonic | NM_018943 | TUBA8 | GACCAAGAGGACCATCCAGTTTGTAGACTGGTGTCCCACAGGCTTCAAGACAGATGCTTG |
| ASCRP3007885 | 0.8442815 | 0.978279024 | 1.0114831 | down | hsa_circRNA_065638 | hsa_circ_0065638 | circBase | chr3 | - | 49394608 | 49395791 | exonic | NM_000581 | GPX1 | GACCAAGGCGAGTTTCCCCACTAATAAAGTGCCGGGTGTCAGCAGAACAGTTAAAAGGAG |
| ASCRP3007886 | 0.302122344 | 0.909237851 | 1.0445266 | down | hsa_circRNA_100425 | hsa_circ_0015928 | circBase | chr1 | + | 201823722 | 201828122 | exonic | NM_018085 | IPO9 | GACCAATGTCATCCTTGCAGACCTCAACCTCTCAGGGTGCAGCCAAAGTCCTGATCTTTC |
| ASCRP3007893 | 0.152559391 | 0.909237851 | 1.1453335 | down | hsa_circRNA_405439 | | 25070500 | chr16 | + | 12155384 | 12162989 | exonic | NM_032167 | SNX29 | GACCAGAGGACCACGTTCTCCCAGATCCTGGACTTCGGTACAGGCCACTGGAAGGGAACA |
| ASCRP3007894 | 0.538862521 | 0.919549314 | 1.0640175 | down | hsa_circRNA_092374 | hsa_circ_0000178 | circBase | chr1 | - | 208017318 | 208017404 | intronic | ENST00000415882 | C1orf132 | GACCAGAGTCATGACAGATGCAGGAAAGGTGCTCCCAGGGACGGGGTTGAACAATAGGAG |
| ASCRP3007895 | 0.836799179 | 0.978181218 | 1.0085084 | down | hsa_circRNA_005128 | hsa_circ_0005128 | circBase | chr3 | + | 111664114 | 111681159 | exonic | NM_145753 | PHLDB2 | GACCAGCATCTCTGCTTGCTCACCAGACAACATCTCTAGGAAAAGGAGAATCTTTGTAAT |
| ASCRP3007901 | 0.553560722 | 0.919549314 | 1.0234036 | down | hsa_circRNA_100149 | hsa_circ_0002306 | circBase | chr1 | + | 33058531 | 33060824 | exonic | NM_001040441 | ZBTB8A | GACCATCTAAGTAGCCATTTTCGAACAAATGGAGATCTCCTCTCATCAGTCTCACCTCCT |
| ASCRP3007907 | 0.053234412 | 0.909237851 | 1.1550246 | down | hsa_circRNA_102554 | hsa_circ_0002945 | circBase | chr19 | + | 41736871 | 41737203 | exonic | NM_001699 | AXL | GACCCACTGCACCCTGCAGGGCTGAACAAGACATCCTCTTTCTCCTGCGAAGCCCATAAC |
| ASCRP3007912 | 0.986009915 | 0.998148217 | 1.0013645 | down | hsa_circRNA_401117 | | 25242744 | chr12 | + | 133721019 | 133728490 | exonic | NM_015394 | ZNF10 | GACCCATCCTGCTTTGTCTCCTCAGCACTCTGCTGTCACTCAAGGAAGTATCATCAAGAA |
| ASCRP3007915 | 0.247471707 | 0.909237851 | 1.0684669 | down | hsa_circRNA_000932 | hsa_circ_0000932 | circBase | chr19 | + | 39943995 | 39944161 | exonic | NM_003169 | SUPT5H | GACCCCCCAAGAAACCCCGCCATGGAGGCTTCATTCTGGACGAGGCTGGTAGACGAAGAG |
| ASCRP3007916 | 0.153114028 | 0.909237851 | 1.0895607 | down | hsa_circRNA_014572 | hsa_circ_0014572 | circBase | chr1 | - | 155327106 | 155330200 | exonic | NM_018489 | ASH1L | GACCCCCCAATTACAGATGAAGCCAATGTCCAATCGTGAAAGGTCTGTTAATGGAGTATA |
| ASCRP3007924 | 0.532514165 | 0.919549314 | 1.0453907 | down | hsa_circRNA_079276 | hsa_circ_0079276 | circBase | chr7 | - | 5568791 | 5570232 | exonic | NM_001101 | ACTB | GACCGAGGCCCCCCTGAACCCCAAGGCCAACCGCGAGAAGATGACCCAGACCGCCGAGAC |
| ASCRP3007929 | 0.353494946 | 0.91119147 | 1.059029 | down | hsa_circRNA_009052 | hsa_circ_0009052 | circBase | chr17 | + | 60741908 | 60742310 | exonic | NM_006039 | MRC2 | GACCTATGTGCTCTGCCCTACCACGAACCCAACGTCTTCCTCATCTTCAGCCATGGACTG |
| ASCRP3007930 | 0.241704661 | 0.909237851 | 1.0504364 | down | hsa_circRNA_100664 | hsa_circ_0005741 | circBase | chr10 | - | 103384501 | 103436193 | exonic | NM_022039 | FBXW4 | GACCTCAACAGGATGACCAGTGTCCCAGTGAAGGAACGAGTGAAGGTGTCTCAGAACTGG |
| ASCRP3007933 | 0.401947862 | 0.91119147 | 1.0697574 | down | hsa_circRNA_103489 | hsa_circ_0067717 | circBase | chr3 | + | 149563797 | 149613347 | exonic | NM_007282 | RNF13 | GACCTCATTAGCATGGGATCCAACGACAGTGATTTTACAACGAGATGCTGCTCTCCATAG |
| ASCRP3007934 | 0.39854412 | 0.91119147 | 1.0247044 | down | hsa_circRNA_003681 | hsa_circ_0003681 | circBase | chr16 | - | 71736500 | 71748704 | exonic | NM_015020 | PHLPP2 | GACCTCGGCTGTATGATTCGATTTTATGGTGAGAAATATGAAACGCAATGGGAGCAGAAA |
| ASCRP3007936 | 0.415890932 | 0.91119147 | 1.0198547 | down | hsa_circRNA_001732 | hsa_circ_0001732 | circBase | chr7 | + | 101813725 | 101838883 | exonic | NM_001913 | CUX1 | GACCTGAGCGAGGGCAGAGGTGGCTCAGAGAGAGGCGGAGACCTTAAGGGAACAGCTCTC |
| ASCRP3007940 | 0.738779592 | 0.953746099 | 1.0140026 | down | hsa_circRNA_026634 | hsa_circ_0026634 | circBase | chr12 | + | 53837174 | 53858636 | exonic | uc010soh.1 | PCBP2 | GACCTGGAGGGACCACCTCTAGAGGGCAGCCAGGGCCAAATCCATATCCCCCCAATATTG |
| ASCRP3007943 | 0.275361848 | 0.909237851 | 1.0407077 | down | hsa_circRNA_402513 | | 25242744 | chr2 | + | 242651392 | 242662686 | exonic | NM_032329 | ING5 | GACCTTACCACGAAACCCAAAGGAAAATGAAGGCCGGGGTCAGAAAGAAAAAAGAGGGTC |
| ASCRP3007944 | 0.245912544 | 0.909237851 | 1.0444328 | down | hsa_circRNA_102614 | hsa_circ_0006089 | circBase | chr2 | + | 9490936 | 9499018 | exonic | NM_003887 | ASAP2 | GACCTTAGATGTACTGGGAACATCTGAGCTGCTGGCTAACCGGCCTCCTGCAAAGCTCAA |
| ASCRP3007946 | 0.786154429 | 0.966895211 | 1.0077724 | down | hsa_circRNA_406898 | | 25070500 | chr7 | - | 1976322 | 2041756 | exonic | NM_003550 | MAD1L1 | GACCTTGAGGCTGCCGCCGCGAGTCTGCCATCGTCCAAGGAGGTGGCAGGCTCAGCTGTC |
| ASCRP3007951 | 0.733694822 | 0.952949359 | 1.0380279 | down | hsa_circRNA_092458 | hsa_circ_0000872 | circBase | chr19 | - | 3976434 | 3976629 | intronic | ENST00000600794 | EEF2 | GACGACACCTGGAGACTGTCCCGACACAGCGACGCTCCCCTGAGAGGTGGTGGCGGAGAC |
| ASCRP3007957 | 0.005932087 | 0.879037174 | 1.0787756 | down | hsa_circRNA_104032 | hsa_circ_0075303 | circBase | chr5 | + | 179146668 | 179146782 | exonic | NM_001746 | CANX | GACGCAGAGAAACCTGAGGATTGGGATGAAGATGCCCCTGCTAAGATTCCAGATGAAGAG |
| ASCRP3007960 | 0.057194081 | 0.909237851 | 1.1244871 | down | hsa_circRNA_042265 | hsa_circ_0042265 | circBase | chr17 | - | 17787947 | 17797124 | exonic | NM_001082968 | TOM1L2 | GACGCTCTGTCTCCCATACACACACCACAGCGGGTGCTGGAGACATGTGTGAAGAACTGT |
| ASCRP3007962 | 0.016411204 | 0.909237851 | 1.0909289 | down | hsa_circRNA_100667 | hsa_circ_0000255 | circBase | chr10 | - | 103432671 | 103436193 | exonic | NM_022039 | FBXW4 | GACGGCCAAGGATGACCAGTGTCCCAGTGAAGGAACGAGTGAAGGTGTCTCAGAACTGGA |
| ASCRP3007965 | 0.022485547 | 0.909237851 | 1.1301846 | down | hsa_circRNA_006349 | hsa_circ_0006349 | circBase | chr1 | + | 32557275 | 32566163 | exonic | NM_018056 | TMEM39B | GACGTCTCCCACTTCCGCTTCCATGTTTGGGATGTACATTCCGTTCCTGCAGCTGAATTG |
| ASCRP3007966 | 0.639752775 | 0.929544944 | 1.0124478 | down | hsa_circRNA_092437 | hsa_circ_0000741 | circBase | chr17 | + | 7402357 | 7402810 | exonic | NM_000937 | POLR2A | GACGTCTTCCTGGAGCGGGTGGAACGGCACATGTGTGATGGGGACATTGTTATCTTCAAC |
| ASCRP3007967 | 0.021199572 | 0.909237851 | 1.0679172 | down | hsa_circRNA_401183 | | 25242744 | chr13 | + | 98996015 | 99030172 | exonic | NM_005766 | FARP1 | GACGTGTAATGACACCAGCGCAGCTCTCTTGATTTCACACATTGTGCAATCAAAGAGCTC |
| ASCRP3007970 | 0.032797828 | 0.909237851 | 1.1002316 | down | hsa_circRNA_058934 | hsa_circ_0058934 | circBase | chr2 | + | 241402763 | 241407495 | exonic | NM_002081 | GPC1 | GACGTGTGTTCTTTTGAGTCCTTGTATGAATAAAAGGCTGGAAACCTACAGTCCCCCTGG |
| ASCRP3007990 | 0.802973428 | 0.974037816 | 1.0380793 | down | hsa_circRNA_004738 | hsa_circ_0004738 | circBase | chr5 | + | 170667931 | 170669824 | exonic | NM_022897 | RANBP17 | GACTCACTACTCTTGGTAATCAGATCCTGTCCCTTGGGAGCCTCTCAAAAGATCAGATTT |
| ASCRP3007996 | 0.488206957 | 0.91119147 | 1.0160467 | down | hsa_circRNA_104931 | hsa_circ_0006088 | circBase | chr9 | + | 131362358 | 131367756 | exonic | NM_003127 | SPTAN1 | GACTCCCACGACCTGCAGCGCTTCCTTAGCGATTTCCGGATGAAACTGATTCCAAGACAG |
| ASCRP3007999 | 0.263484911 | 0.909237851 | 1.1332223 | down | hsa_circRNA_004885 | hsa_circ_0004885 | circBase | chr1 | - | 6659106 | 6659512 | exonic | NM_014851 | KLHL21 | GACTCTAAACGGACTCATGTACTTTGTCAGGTGGGTCCGATGGCTCCCGGCTCTATGACT |
| ASCRP3008003 | 0.176944632 | 0.909237851 | 1.0691505 | down | hsa_circRNA_103023 | hsa_circ_0059859 | circBase | chr20 | + | 32198899 | 32199141 | exonic | NM_005093 | CBFA2T2 | GACTCTTGTTCTTGCACTGGTGTAAGCAATGGCATCAACCATTCTCCTCCTACCCTGAAT |
| ASCRP3008012 | 0.440813463 | 0.91119147 | 1.0683505 | down | hsa_circRNA_101061 | hsa_circ_0004313 | circBase | chr12 | + | 51449617 | 51450285 | exonic | NM_015416 | LETMD1 | GACTGCTCAGGAAGTAAAATCGGTACCTGTTTCCCAGGCAACTACTGATCAGGCATTTCT |
| ASCRP3008017 | 0.152376994 | 0.909237851 | 1.0678118 | down | hsa_circRNA_049382 | hsa_circ_0049382 | circBase | chr19 | + | 11217240 | 11218190 | exonic | NM_000527 | LDLR | GACTGGTCAGATGAACCCATCAAAGAGTGCGCTGTGGCCACCTGTCGCCCTGACGAATTC |
| ASCRP3008018 | 0.669387894 | 0.938080316 | 1.0186664 | down | hsa_circRNA_007730 | hsa_circ_0007730 | circBase | chr12 | + | 51076920 | 51090958 | exonic | NM_173602 | DIP2B | GACTGTAGCAATCCGCAGGTAGCCCTGGTTTACCCCAACAATGATCCAGTCATGTTTATG |
| ASCRP3008020 | 0.70070594 | 0.944102043 | 1.0236048 | down | hsa_circRNA_400095 | hsa_circ_0092321 | circBase | chr9 | + | 116764513 | 116764813 | intronic | ENST00000288466 | ZNF618 | GACTGTGAGCTCTTCCCCGAAGGGAGGCCTCCTGATGCGGGACAGCTCAGGTGTGGGAGG |
| ASCRP3008023 | 0.303130298 | 0.909237851 | 1.0228812 | down | hsa_circRNA_101968 | hsa_circ_0041811 | circBase | chr17 | - | 7217398 | 7217478 | exonic | NM_004489 | GPS2 | GACTGTTCACACAGGAACTCATCTCCTCAGCATGCAGGTGACCTGACCACCCTAACATCA |
| ASCRP3008026 | 0.216285299 | 0.909237851 | 1.059926 | down | hsa_circRNA_104209 | hsa_circ_0078155 | circBase | chr6 | + | 147527106 | 147655358 | exonic | NM_139244 | STXBP5 | GACTGTTGTTCCAGAGGATCGCTGCAAATCTCCAACCTCTGACTGTTCGCCATGGATTTC |
| ASCRP3008028 | 0.433158468 | 0.91119147 | 1.033308 | down | hsa_circRNA_405755 | | 25070500 | chr19 | - | 19756461 | 19756599 | sense overlapping | NM_020410 | ATP13A1 | GACTTCAACAGCCAGTTTGGCCTCGTGGACATCCCTGGCCCGCCCTTCATGGAGAGCCTG |
| ASCRP3008034 | 0.640395116 | 0.929544944 | 1.0627549 | down | hsa_circRNA_000686 | hsa_circ_0000686 | circBase | chr16 | + | 29677910 | 29678100 | intronic | ENST00000449759 | QPRT | GACTTCCCCTGTGGGGCCTGGGGGGAGAGGGGTTTGCCCTGGTCTCGGGGACTGGTCTGG |
| ASCRP3008036 | 0.718396764 | 0.948649554 | 1.0153321 | down | hsa_circRNA_005361 | hsa_circ_0005361 | circBase | chr1 | - | 46099160 | 46108171 | exonic | NM_021639 | GPBP1L1 | GACTTCTCAGAGAATAGAGACTGTGACAAGCTGGAAGATCCTTCCTTGAATCCAGAAGCT |
| ASCRP3008040 | 0.306733207 | 0.909237851 | 1.0640796 | down | hsa_circRNA_028294 | hsa_circ_0028294 | circBase | chr12 | + | 112165765 | 112165947 | exonic | NM_025247 | ACAD10 | GACTTGAAGACTATGGGAAGCAAGTGTCATTGGCACCCCCTTCTATGTGATGGAGTACTG |
| ASCRP3008054 | 0.959653832 | 0.993354918 | 1.0017269 | down | hsa_circRNA_404552 | | 25070500 | chr1 | + | 94964157 | 94965170 | exonic | NM_002858 | ABCD3 | GAGAAAAGCAAAGAATGGCGGTTCGATCTGGGGCTAATGTTCTAATTTGTGGTCCAAATG |
| ASCRP3008084 | 0.118400709 | 0.909237851 | 1.0646145 | down | hsa_circRNA_059914 | hsa_circ_0059914 | circBase | chr20 | - | 32878137 | 32881962 | exonic | NM_000687 | AHCY | GAGAAGGTGAACATCAAGCCGCAGGTGCAGTGGTCCAGCTGCAACATCTTCTCCACCCAG |
| ASCRP3008086 | 0.966070427 | 0.995283777 | 1.002211 | down | hsa_circRNA_103237 | hsa_circ_0063526 | circBase | chr22 | - | 41650311 | 41677086 | exonic | NM_002883 | RANGAP1 | GAGAAGTCAGCCACGCCCTCACGGAAGATTCTGGACCCTAACACTGGGTCTGCAGATCTC |
| ASCRP3008089 | 0.130741557 | 0.909237851 | 1.0566799 | down | hsa_circRNA_010844 | hsa_circ_0010844 | circBase | chr1 | + | 23408712 | 23410184 | exonic | NM_015013 | KDM1A | GAGAATAAAACTTCATATAAAATTGGCCCCAAAGAAACTGTGGTGTCTCGTTGGCGTGCT |
| ASCRP3008096 | 0.320462136 | 0.909237851 | 1.0456367 | down | hsa_circRNA_000440 | hsa_circ_0000440 | circBase | chr12 | - | 113487898 | 113488149 | intergenic |  |  | GAGAATGTTTCCTGCGTTTCCCAAGGGGTGACATCTGAGCCATGACTTGAAGGAGAGTAG |
| ASCRP3008100 | 0.367758469 | 0.91119147 | 1.0305816 | down | hsa_circRNA_006026 | hsa_circ_0006026 | circBase | chr22 | + | 31976257 | 32007826 | exonic | NM_001007467 | SFI1 | GAGACAACAGTATTTTTGCTTTAGAGCCCTAAAAGACAATGTGACCCACGCTCATCTCCA |
| ASCRP3008102 | 0.311483371 | 0.909237851 | 1.0742404 | down | hsa_circRNA_102265 | hsa_circ_0046557 | circBase | chr17 | + | 80851422 | 80869665 | exonic | NM_005993 | TBCD | GAGACAAGCAGGGCACTTTCCCTCATGGTATTGATATTTTGACCACAGCTGACTATTTTG |
| ASCRP3008106 | 0.774023073 | 0.964265101 | 1.0176622 | down | hsa_circRNA_052507 | hsa_circ_0052507 | circBase | chr2 | - | 9017159 | 9048846 | exonic | NM_138799 | MBOAT2 | GAGACACAGTATGAAAGAACAGAGCCATCTCCAAATTTACTGCTTTGTGTTTGCTCTGGG |
| ASCRP3008121 | 0.247009558 | 0.909237851 | 1.0452827 | down | hsa_circRNA_103910 | hsa_circ_0073271 | circBase | chr5 | - | 88044886 | 88047860 | exonic | NM_002397 | MEF2C | GAGACCTCACGTCTGGTGCAGGCACCAGTGCAGGCTGTTCCACCTCCCAACTTCGAGATG |
| ASCRP3008123 | 0.29769322 | 0.909237851 | 1.0540411 | down | hsa_circRNA_405375 | | 25070500 | chr15 | + | 68937498 | 68937926 | sense overlapping | NM_006091 | CORO2B | GAGACCTGAGATCTCTTTCCAGAGCTTGCCTAAAGATGTCCTGGCGTCCGCAATACCGTA |
| ASCRP3008126 | 0.358833385 | 0.91119147 | 1.0465716 | down | hsa_circRNA_001386 | hsa_circ_0001386 | circBase | chr4 | - | 1219147 | 1235307 | exonic | NM_001012614 | CTBP1 | GAGACCTTGGGCATCATCGGACTTGTAAGACTTTGACCCCGACGCAGGCTTCAGTGATTG |
| ASCRP3008130 | 0.542174805 | 0.919549314 | 1.0211893 | down | hsa_circRNA_100559 | hsa_circ_0000219 | circBase | chr10 | - | 15858833 | 15889942 | exonic | NM_024948 | FAM188A | GAGACTCACCTCACCGTATTTTTTGCCAAGGGTTTGTGTTTAGTGAATCAGAGGGATCTG |
| ASCRP3008132 | 0.526802832 | 0.916971445 | 1.038154 | down | hsa_circRNA_100334 | hsa_circ_0014132 | circBase | chr1 | + | 151214598 | 151215043 | exonic | NM_003557 | PIP5K1A | GAGACTTTGCAAATGCTAACTACAAGTGAAGCCTTCTCCTTCCAAAAAGTTTCGGTCTGG |
| ASCRP3008133 | 0.532858525 | 0.919549314 | 1.0620425 | down | hsa_circRNA_400862 | | 25242744 | chr11 | - | 122954387 | 122968660 | exonic | NM_024769 | CLMP | GAGAGAAAGAGGGAGAGGATGAACGTCTGCCTCCCAAATCTAGGATTGTTTCCTACTATG |
| ASCRP3008137 | 0.601107506 | 0.929544944 | 1.027779 | down | hsa_circRNA_021845 | hsa_circ_0021845 | circBase | chr11 | - | 46455023 | 46534363 | exonic | NM_017749 | AMBRA1 | GAGAGACCTCCATGAGGGAGGCTGCTGGAATCTTCCCTCATTTCATTATCCCGTTATGAT |
| ASCRP3008138 | 0.635033811 | 0.929544944 | 1.0120421 | down | hsa_circRNA_000578 | hsa_circ_0000487 | circBase | chr13 | - | 50655754 | 50656296 | intronic | ENST00000425586 | DLEU2 | GAGAGACGGGGCTGAGGGGGGAGGGTTGGAGTTTGCGCATGCGTAAAAATGTCGGGAAAG |
| ASCRP3008143 | 0.355300086 | 0.91119147 | 1.0455973 | down | hsa_circRNA_080285 | hsa_circ_0080285 | circBase | chr7 | - | 56150970 | 56151408 | exonic | NM_006213 | PHKG1 | GAGAGAGGCTGCGAGGATGAAGAGAGGGGAGCTCTTTGACTACCTCACTGAGAAGGTCAC |
| ASCRP3008144 | 0.481181627 | 0.91119147 | 1.028482 | down | hsa_circRNA_001532 | hsa_circ_0001532 | circBase | chr5 | - | 134862377 | 134862465 | intergenic |  |  | GAGAGATACAGTAGAGAACCAGCCGTCACCTCAGGAAGGGAGAAGGCTATAACTCTGCAG |
| ASCRP3008145 | 0.360320517 | 0.91119147 | 1.0728028 | down | hsa_circRNA_080719 | hsa_circ_0080719 | circBase | chr7 | - | 75190632 | 75197560 | exonic | NM_005338 | HIP1 | GAGAGATCAGTGGATTGAAGGCACAGCTAGAAAACATGAAGACTGAGGCCTCCCAGCTGA |
| ASCRP3008151 | 0.920188693 | 0.98346125 | 1.0038296 | down | hsa_circRNA_102203 | hsa_circ_0045848 | circBase | chr17 | + | 75085234 | 75085353 | exonic | NR_027058 | SNHG20 | GAGAGCATTCGAAGTGACCTCAGCAGGGCATCCAGGTTTCAACAGAAAATGAGAAATATC |
| ASCRP3008156 | 0.851891339 | 0.978900086 | 1.0260958 | down | hsa_circRNA_401622 | | 25242744 | chr16 | - | 67270459 | 67271285 | exonic | NM_013241 | FHOD1 | GAGAGGAGCATCTACAAACGCTGGCGGCGCTCCCGGACCAGGACTCCTTCTACGATGTGA |
| ASCRP3008165 | 0.808009813 | 0.974037816 | 1.0224113 | down | hsa_circRNA_000481 | hsa_circ_0001828 | circBase | chr8 | + | 142139086 | 142139265 | intronic | ENST00000262585 | DENND3 | GAGAGTCTCTGGGGCCTCTCTCTTCAGTCGCTCCCATCTTGGGCAGGTGCCCTGGAGCAG |
| ASCRP3008171 | 0.251383451 | 0.909237851 | 1.1481825 | down | hsa_circRNA_406748 | | 25070500 | chr6 | - | 29643162 | 29643836 | exonic | NM_001109809 | ZFP57 | GAGAGTTTGTCCATCTCCCAAACACAGAAGGCCTTTCAGAAGCCAGTCACCTTTGAGGAT |
| ASCRP3008173 | 0.577697393 | 0.923965283 | 1.0244018 | down | hsa_circRNA_001382 | hsa_circ_0000602 | circBase | chr15 | - | 52725371 | 52726564 | sense overlapping | NM_000259 | MYO5A | GAGATAAAGTCCTCCTGCTTCACCTCGAGGAAGGAAAGGATGTCTGAGAAATCACTAAGG |
| ASCRP3008177 | 0.542201268 | 0.919549314 | 1.028846 | down | hsa_circRNA_003814 | hsa_circ_0003814 | circBase | chr6 | - | 90461149 | 90472249 | exonic | NM_014611 | MDN1 | GAGATATAGTCCGAGTTGTCTCTGCAGGTTATAAACCGGTGGACCATAAGCTTATTTGGC |
| ASCRP3008184 | 0.586652753 | 0.927905266 | 1.0192231 | down | hsa_circRNA_400379 | | 25242744 | chr1 | - | 202104277 | 202104928 | exonic | NM_138795 | ARL8A | GAGATCTGCTGCTACTCCATCTCTTGCAAAGAAAAGGACAACATTGGTACATGGTGGATG |
| ASCRP3008185 | 0.120121793 | 0.909237851 | 1.0509922 | down | hsa_circRNA_029545 | hsa_circ_0029545 | circBase | chr12 | + | 133291443 | 133294706 | exonic | NM_138575 | PGAM5 | GAGATCTTCATCTGTCACGCCAACGTCATCCGCTACATCGTGTGCAGGCGAGAACCACTG |
| ASCRP3008186 | 0.172363458 | 0.909237851 | 1.0912353 | down | hsa_circRNA_103209 | hsa_circ_0009004 | circBase | chr22 | - | 36712561 | 36714370 | exonic | NM_002473 | MYH9 | GAGATCTTTGATCTGCCCAAAAGGTGTCCCATCTCTTGGGTATCAATGTGACCGATTTCA |
| ASCRP3008192 | 0.15866414 | 0.909237851 | 1.0996708 | down | hsa_circRNA_405076 | | 25070500 | chr12 | - | 118511503 | 118511797 | exonic | NM_019086 | VSIG10 | GAGATGGAAATCTGGCTGAGTGTGAAAGGGGGTCCCTCCCTTCTCTCTGAGCCCATGAAG |
| ASCRP3008195 | 0.976467055 | 0.996952313 | 1.0008446 | down | hsa_circRNA_104367 | hsa_circ_0080170 | circBase | chr7 | - | 47451324 | 47467974 | exonic | NM_022748 | TNS3 | GAGATGTCATGGGCGGGAAAGGACGCATAGGAGTGGTCATATCATCCTACATGCATTTCA |
| ASCRP3008203 | 0.756390226 | 0.957967508 | 1.006143 | down | hsa_circRNA_101456 | hsa_circ_0006770 | circBase | chr15 | - | 20666444 | 20688770 | exonic | uc001ytg.3 | HERC2P3 | GAGATTGAATGTGCCACTTGCATTCACAAGAGATGGGCTCTGTGGTCTGTGGAATGAAAT |
| ASCRP3008205 | 0.739785331 | 0.953746099 | 1.0315617 | down | hsa_circRNA_103747 | hsa_circ_0001449 | circBase | chr4 | + | 148860975 | 148876525 | exonic | NM_024605 | ARHGAP10 | GAGATTTCATTGTTCCAGCCAGTATAAATGACCAAGGATTGTACAGAGTTGTGGGGGTGA |
| ASCRP3008206 | 0.003127064 | 0.870215632 | 1.0565075 | down | hsa_circRNA_104686 | hsa_circ_0085540 | circBase | chr8 | + | 128902834 | 128951922 | exonic | uc003ysl.3 | PVT1 | GAGATTTGGAGAGGCCTGATCTTTTGGCCAGAAGGAGATTAAAAAGATGCCCCTCAAGAT |
| ASCRP3008207 | 0.222432979 | 0.909237851 | 1.1335378 | down | hsa_circRNA_405391 | | 25070500 | chr15 | - | 75766029 | 75782642 | exonic | NM_002833 | PTPN9 | GAGCAAAAAGTCTTGGTGATTGTCATGACCACCCGGTCTCCAGGAAACCTAGAGAAAAAC |
| ASCRP3008208 | 0.195702104 | 0.909237851 | 1.0614568 | down | hsa_circRNA_104067 | hsa_circ_0002245 | circBase | chr6 | + | 17507399 | 17514185 | exonic | NM_006366 | CAP2 | GAGCAAAACAAATGACGTGGCCGCACTTCTGAAACCCATATCGGAAAAGATTCAGGAAAT |
| ASCRP3008213 | 0.723790778 | 0.949785489 | 1.0222422 | down | hsa_circRNA_000561 | hsa_circ_0000561 | circBase | chr14 | + | 93118615 | 93118705 | sense overlapping | NM_024832 | RIN3 | GAGCAAGGCCAGGACACAGAGGGGGACCAGCTCAGCCTGCCTCCCCAAGGGACCTCAGAC |
| ASCRP3008218 | 0.666035023 | 0.937594692 | 1.0191632 | down | hsa_circRNA_017469 | hsa_circ_0017469 | circBase | chr10 | + | 1170157 | 1170964 | exonic | NM_014023 | WDR37 | GAGCAGCCGACAGCACTGTGACTTCTGCCGTGTTCACCGTGGGAGACAACGTGGTTTCAG |
| ASCRP3008219 | 0.153802756 | 0.909237851 | 1.1073596 | down | hsa_circRNA_008593 | hsa_circ_0008593 | circBase | chr1 | + | 198190128 | 198222310 | sense overlapping | NM_133494 | NEK7 | GAGCAGCCTGTCTCTTGGATGGAGTACCAGTAGCTTTAAAAAAAGTGCAGGAAAAGGAAA |
| ASCRP3008222 | 0.382916739 | 0.91119147 | 1.0242298 | down | hsa_circRNA_000639 | hsa_circ_0000844 | circBase | chr18 | + | 42269709 | 42269921 | intronic | ENST00000282030 | SETBP1 | GAGCAGGTGGGTATGGAGCAGCAGGGAAGATCATCCATTTGGGAATGTAGAGGGAGAAAG |
| ASCRP3008227 | 0.734861461 | 0.952949359 | 1.0116621 | down | hsa_circRNA_103403 | hsa_circ_0066367 | circBase | chr3 | + | 58131647 | 58135954 | exonic | NM_001457 | FLNB | GAGCATTTGTGACCTGAACCTGAAAATCCCAGAGAGCCCACTCCAGTTCTACGTGAACTA |
| ASCRP3008241 | 0.778228504 | 0.965597559 | 1.0221126 | down | hsa_circRNA_402089 | | 25242744 | chr19 | + | 35169641 | 35174143 | exonic | NM_018443 | ZNF302 | GAGCCCTGGATGATGGAGAAAAAACTGTCAAAAGGACACTGCCCATCTCTAAGATAAGAA |
| ASCRP3008242 | 0.822826434 | 0.976440085 | 1.0075638 | down | hsa_circRNA_104970 | hsa_circ_0002172 | circBase | chrX | - | 299338 | 308428 | exonic | NM_013239 | PPP2R3B | GAGCCCTGGGAGGACGGGCCTGCGGCTGCCCCCTCTACTGGAAGGGGCCGCTCTTCTATG |
| ASCRP3008246 | 0.274892361 | 0.909237851 | 1.0424364 | down | hsa_circRNA_103056 | hsa_circ_0004053 | circBase | chr20 | - | 35433137 | 35434360 | exonic | NM_080627 | SOGA1 | GAGCGGGCAGAGCTCAAGGGCCATACCTCCCAGGTGAACAGCATGAAGGAGCTGTACTTG |
| ASCRP3008251 | 0.238097504 | 0.909237851 | 1.0371589 | down | hsa_circRNA_004930 | hsa_circ_0004930 | circBase | chr7 | - | 148543561 | 148547617 | sense overlapping | NM_004456 | EZH2 | GAGCTCATTGCGCGGGACTAGGGAGGTCTACCTATTTAGTCTTACACTTTCAAAGAACTA |
| ASCRP3008254 | 0.840575302 | 0.978181218 | 1.0127341 | down | hsa_circRNA_008404 | hsa_circ_0008404 | circBase | chr14 | + | 77930940 | 77932010 | exonic | NM_012111 | AHSA1 | GAGCTCTATAGAGTGTTTACCACCCAAGAGAGTTCACCCAGGGCATGATCTTACCTACAA |
| ASCRP3008261 | 0.854264245 | 0.978900086 | 1.0202084 | down | hsa_circRNA_101976 | hsa_circ_0041992 | circBase | chr17 | - | 8109808 | 8111158 | exonic | NM_004217 | AURKB | GAGCTGAAGATTGCTGACTTCGGCTGGTCTGTGCATGCGCCCTCCCTGAGGCTCCATCTG |
| ASCRP3008263 | 0.968009006 | 0.995499551 | 1.0014084 | down | hsa_circRNA_102193 | hsa_circ_0003684 | circBase | chr17 | - | 73238416 | 73239651 | exonic | NM_014001 | GGA3 | GAGCTGATGAAGTACCTGGGGGACAGGGTGTCTGAGAAAGTGAAGACCAAGGTTATTGAG |
| ASCRP3008272 | 0.761564313 | 0.961197676 | 1.010977 | down | hsa_circRNA_100011 | hsa_circ_0000007 | circBase | chr1 | - | 1735857 | 1737977 | exonic | NM_002074 | GNB1 | GAGCTGGCAGGACACACAGGCTTCTCGTCAGTGCCTCGCAGGATGGTAAACTTATCATCT |
| ASCRP3008277 | 0.802653531 | 0.974011963 | 1.0277466 | down | hsa_circRNA_001161 | hsa_circ_0001161 | circBase | chr20 | + | 44640275 | 44640841 | sense overlapping | NM_004994 | MMP9 | GAGCTGTGCGTCTTCCCCTTCACTTTCCTGGGCCAATCCTACTCCGCCTGCACCACGGAC |
| ASCRP3008279 | 0.318034093 | 0.909237851 | 1.0685845 | down | hsa_circRNA_026036 | hsa_circ_0026036 | circBase | chr12 | - | 49054148 | 49063055 | exonic | NM_017822 | KANSL2 | GAGCTTCAGCCCACTGAGAGTTTACCTCTGGAGTTCAGTGATGCAGTAGTCTCCTGACTG |
| ASCRP3008282 | 0.615636558 | 0.929544944 | 1.021887 | down | hsa_circRNA_006464 | hsa_circ_0006464 | circBase | chr2 | + | 242606059 | 242608100 | exonic | NM_013325 | ATG4B | GAGCTTGACCCGTCCATCGCTGTGGAAGGTTGTGCAGGACCAGCGTTCCCTGTGCAGGCG |
| ASCRP3008292 | 0.895162228 | 0.980833002 | 1.0031459 | down | hsa_circRNA_001223 | hsa_circ_0001223 | circBase | chr22 | - | 35948707 | 35948901 | antisense | NM_014310 | RASD2 | GAGGAACGCGGTCAGGAGGAAGGCGTCCCGGGTGGAGGCCGTGGATCTGAACTGGGTGAG |
| ASCRP3008306 | 0.56757779 | 0.920978531 | 1.0256794 | down | hsa_circRNA_001108 | hsa_circ_0001132 | circBase | chr20 | - | 8352261 | 8352391 | antisense | NM_015192 | PLCB1 | GAGGACATAAAGCAAGCCCCTCTGTCCAAGAGCCCACATATTTGTTCATTTACTTTTTAA |
| ASCRP3008308 | 0.96256176 | 0.99396088 | 1.0013154 | down | hsa_circRNA_101615 | hsa_circ_0036444 | circBase | chr15 | - | 78398080 | 78398276 | exonic | NM_006383 | CIB2 | GAGGACATGATTGCCAAGGCCCCTGACTTCCTCAGACTTCAACACTGACAACTTCATCTG |
| ASCRP3008311 | 0.819013111 | 0.976440085 | 1.0079253 | down | hsa_circRNA_100031 | hsa_circ_0009577 | circBase | chr1 | - | 8525984 | 8674745 | exonic | NM_012102 | RERE | GAGGACGAAGTGGTCCACAACTCCCAGGCCTGTTGCAGATCTCCAACTCCTGCTTTGTGT |
| ASCRP3008320 | 0.499741727 | 0.91447302 | 1.0308564 | down | hsa_circRNA_404678 | | 25070500 | chr1 | + | 230381808 | 230386306 | exonic | NM_004481 | GALNT2 | GAGGAGAGAACCTAGGACAGGACTCGGGTTGTGTCACCCATCATCGATGTCATTAATATG |
| ASCRP3008324 | 0.049958188 | 0.909237851 | 1.0769832 | down | hsa_circRNA_402632 | | 25242744 | chr20 | - | 62275108 | 62275662 | exonic | NM_015894 | STMN3 | GAGGAGCGGAGGAAGCCTACAAGGAGAAGATGAAGGAGCTGTCGGTGCTGTCGCTCATCT |
| ASCRP3008325 | 0.884226568 | 0.979863126 | 1.0048568 | down | hsa_circRNA_000634 | hsa_circ_0000634 | circBase | chr15 | - | 74988220 | 74988302 | intronic | ENST00000569561 | EDC3 | GAGGAGCTCCAGGCCCGTCGGCGCGGAGAGAATTCTTCTGTGGGTCCTCTGGTGTTGAGT |
| ASCRP3008326 | 0.272421436 | 0.909237851 | 1.0740731 | down | hsa_circRNA_406169 | | 25070500 | chr22 | - | 18324587 | 18379747 | exonic | NM_015241 | MICAL3 | GAGGAGGACGTCGCCAGCAGGACTACGCCGACACAGAGCTCCTGCTTTCCCGAGAAAACG |
| ASCRP3008334 | 0.017981906 | 0.909237851 | 1.0531117 | down | hsa_circRNA_100556 | hsa_circ_0003136 | circBase | chr10 | - | 13375816 | 13387028 | exonic | NM_012247 | SEPHS1 | GAGGAGTGGCTACCACTGTCTGCCAACCCAATGAATTTATCATGTGGATCCATAAAGAAC |
| ASCRP3008338 | 0.718080912 | 0.948649554 | 1.0148426 | down | hsa_circRNA_407093 | | 25070500 | chr8 | - | 90657364 | 90660841 | intronic | ENST00000519655 | RP11-37B2.1 | GAGGATAGGAAAAGTCAGCTCTCCTTATACAGGTAGAGGAGAACGTCGCAGAGAAAAAGG |
| ASCRP3008343 | 0.777438297 | 0.96521144 | 1.0272137 | down | hsa_circRNA_101004 | hsa_circ_0000375 | circBase | chr12 | - | 6657590 | 6657991 | exonic | NM_080730 | IFFO1 | GAGGATTTCTCAGGCTATGCCATGGCAGCTGCAGAGGCCCAGGGAGAGGTCCCATCCATG |
| ASCRP3008345 | 0.345080153 | 0.91119147 | 1.0696779 | down | hsa_circRNA_100876 | hsa_circ_0023404 | circBase | chr11 | + | 71668272 | 71671937 | exonic | NM_018320 | RNF121 | GAGGCACCCACGCTCCTACAATGTTGATATGTCAGATCTCTCTCCAGAAGAGCAATGGAG |
| ASCRP3008349 | 0.706274847 | 0.945024804 | 1.0195981 | down | hsa_circRNA_079973 | hsa_circ_0079973 | circBase | chr7 | - | 43906156 | 43909145 | exonic | NM_032014 | MRPS24 | GAGGCATAGTCTCTCATCTGCTATTGAATAAAGACCTTCTATCTTGTCCCCACCCCTCAG |
| ASCRP3008353 | 0.174089927 | 0.909237851 | 1.1269345 | down | hsa_circRNA_000380 | hsa_circ_0000380 | circBase | chr12 | - | 15095126 | 15095229 | sense overlapping | NM_001175 | ARHGDIB | GAGGCCAGAAAATGGGCAAATTATCACTAACAGATCTTCCACGTCTCCATCTCAGTACAC |
| ASCRP3008355 | 0.588675627 | 0.927905266 | 1.0274469 | down | hsa_circRNA_040730 | hsa_circ_0040730 | circBase | chr16 | + | 85667519 | 85695371 | exonic | NM_014615 | GSE1 | GAGGCCCAGGAGAAAGGCATGAGCCATGAGCCCAAGTCCCCTTCGCTAGGGATGCTTTCC |
| ASCRP3008361 | 0.720351393 | 0.949318546 | 1.0082557 | down | hsa_circRNA_104982 | hsa_circ_0006931 | circBase | chrX | + | 16846216 | 16847893 | exonic | NM_018360 | TXLNG | GAGGGAAGAGCTGGAGGAGAGCAGGAGTGTTCAGAAGCAAATGAAGATCCTGCAGAAGAA |
| ASCRP3008362 | 0.955066747 | 0.992245164 | 1.0016138 | down | hsa_circRNA_103778 | hsa_circ_0005694 | circBase | chr5 | + | 353844 | 376831 | exonic | NM_020731 | AHRR | GAGGGAAGGCTGCTGTTGGAGGAGGCCCGCCGTGGGGGCAGAGAAGTCCAACCCCTCCAA |
| ASCRP3008364 | 0.783236138 | 0.966660917 | 1.0067406 | down | hsa_circRNA_404541 | | 25070500 | chr1 | - | 85121514 | 85136991 | exonic | NM_014021 | SSX2IP | GAGGGAGAGACGAAGCTTTACAGAAGCCGCTATTCGCCTGGGATTGGAGGAGATGCCTAC |
| ASCRP3008365 | 0.632815799 | 0.929544944 | 1.0118564 | down | hsa_circRNA_002008 | hsa_circ_0000406 | circBase | chr12 | - | 56552657 | 56552839 | antisense | NM_021019 | MYL6 | GAGGGAGGAAGAAAAGGAGAAAAGAGGCAGAAGTAGCTGATTAAAGGACAAAGGAAAGCA |
| ASCRP3008366 | 0.599484684 | 0.929544944 | 1.0246101 | down | hsa_circRNA_092405 | hsa_circ_0000525 | circBase | chr14 | + | 23778198 | 23778449 | intronic | ENST00000553781 | BCL2L2-PABPN1 | GAGGGATCACGCCTATAGGTGTGGCTGGGGGCCAGGAGAGCAGGAACAGAACAGAGAAAT |
| ASCRP3008369 | 0.65251031 | 0.933189368 | 1.0112516 | down | hsa_circRNA_007593 | hsa_circ_0007593 | circBase | chr19 | - | 19118155 | 19119237 | exonic | ENST00000600239 | SUGP2 | GAGGGGAGAGAGTTGACATGAAGACAATGGAGACTGCAGAGAAACTGGCTAGATTTGTTG |
| ASCRP3008374 | 0.5727658 | 0.922333971 | 1.0176212 | down | hsa_circRNA_000666 | hsa_circ_0000666 | circBase | chr16 | + | 1797029 | 1798721 | exonic | NM_015133 | MAPK8IP3 | GAGGTCATCGGGGATGTGGACGAAGGGGCCGACCTCCTAGTGTCCACAGGATGAAATGTC |
| ASCRP3008380 | 0.763271419 | 0.962146956 | 1.0051169 | down | hsa_circRNA_053944 | hsa_circ_0053944 | circBase | chr2 | - | 33808728 | 33810511 | exonic | uc002roz.1 | FAM98A | GAGGTGTGCTTTGTGTGAAAGGTGAGCAATAAAGTATCTGTTAAGTTCGTGTTGATGGGC |
| ASCRP3008381 | 0.848687859 | 0.978900086 | 1.0109354 | down | hsa_circRNA_402100 | | 25242744 | chr19 | + | 40704281 | 40704462 | exonic | NM_002446 | MAP3K10 | GAGGTTATCCGTCTCTCCCTCTTCTCCAAAAGCAGTGATGTCTGGAGTCCTGATCCTGGA |
| ASCRP3008383 | 0.236018843 | 0.909237851 | 1.0272815 | down | hsa_circRNA_404492 | | 25070500 | chr1 | - | 42330248 | 42335301 | exonic | ENST00000479350 | HIVEP3 | GAGGTTCTCCTCATGACTCAGAGCCCCTCTGAGAAGAGAAACAGGATTATAGAACACATG |
| ASCRP3008392 | 0.311332319 | 0.909237851 | 1.0660985 | down | hsa_circRNA_406839 | | 25070500 | chr6 | + | 126131327 | 126132928 | intronic | ENST00000229634 | NCOA7 | GAGTACTAACTACCCCAACTCATGGTAGCATGGCTTCCACTCCCAATACGTCATTGAGGA |
| ASCRP3008394 | 0.712140998 | 0.946024376 | 1.0146939 | down | hsa_circRNA_100937 | hsa_circ_0004214 | circBase | chr11 | + | 94532555 | 94533477 | exonic | NM_130847 | AMOTL1 | GAGTATGGGGTAACGAGTTGAAGATCCTCTTTGTAACTTCCACTCCCCAAACTTCCTGAG |
| ASCRP3008409 | 0.658125028 | 0.933884861 | 1.0124106 | down | hsa_circRNA_074407 | hsa_circ_0074407 | circBase | chr5 | - | 145522974 | 145547828 | exonic | NM_020117 | LARS | GAGTCTCCGCTGGGCATTAGGCATGTGCTGATAAGTTGAAAAGAGAAATAGAGCTGTATG |
| ASCRP3008423 | 0.165652356 | 0.909237851 | 1.0466119 | down | hsa_circRNA_404903 | | 25070500 | chr11 | - | 70644547 | 70666787 | exonic | NM_012309 | SHANK2 | GAGTGCGTGGAGGAGGTCCAGTGTAAGCCCAGGGACAGCCAGGCAGTGCCCTTCCGAGAG |
| ASCRP3008426 | 0.02311701 | 0.909237851 | 1.1802881 | down | hsa_circRNA_404938 | | 25070500 | chr11 | - | 116627861 | 116631668 | exonic | NM_032725 | BUD13 | GAGTGGACAGGTGACTGCCAGAAAGCAACTGATTCAGACCTTTCTTCTCCACGGCATAAA |
| ASCRP3008434 | 0.695321944 | 0.943480789 | 1.014374 | down | hsa_circRNA_407273 | | 25070500 | chrX | - | 16871028 | 16871799 | intronic | ENST00000380084 | RBBP7 | GAGTGTGTCATCCCTGGGTGGAAAATTCAGTGGGATCTTGTTAAATGGGTGACCTGAATG |
| ASCRP3008439 | 0.753149295 | 0.957534606 | 1.018491 | down | hsa_circRNA_083391 | hsa_circ_0083391 | circBase | chr8 | + | 15508205 | 15519805 | exonic | NM_006765 | TUSC3 | GAGTTCATCTATAACAAGACTGGTTGGGCCATGGTGTCTCTGGCAAGCTAATGAAGAATA |
| ASCRP3008442 | 0.85113879 | 0.978900086 | 1.0095211 | down | hsa_circRNA_101593 | hsa_circ_0004401 | circBase | chr15 | - | 74948073 | 74948409 | exonic | NM_025083 | EDC3 | GAGTTCTGCACGGGGTCATCTAGTAGCAGGCACCCAAATCAGGCAACTCCCAAGAAAAGT |
| ASCRP3008447 | 0.559949707 | 0.920978531 | 1.0273897 | down | hsa_circRNA_001369 | hsa_circ_0000401 | circBase | chr12 | - | 52308994 | 52309096 | antisense | NM_000020 | ACVRL1 | GAGTTGCGGGAGGTCATGTCTGAGGCGATGAAGCCTGTGTGCAGAAAGTCGTAGAGGGAG |
| ASCRP3008450 | 0.267553777 | 0.909237851 | 1.0531275 | down | hsa_circRNA_404527 | | 25070500 | chr1 | - | 67861087 | 67868100 | antisense | NM_001559 | IL12RB2 | GAGTTTGCGAGGGTGGGAAGAAGACCCTCTGATCTCTTGAGAGGTGCTTTACTCATGAGG |
| ASCRP3008454 | 0.09289244 | 0.909237851 | 1.0886372 | down | hsa_circRNA_103808 | hsa_circ_0004599 | circBase | chr5 | - | 31448653 | 31451747 | exonic | NM_013235 | DROSHA | GATAAATATCATGTCACGCCTTGGCCAAGATGACCCAACTCCCTCGAGCATGCAATGATG |
| ASCRP3008458 | 0.08861814 | 0.909237851 | 1.0703332 | down | hsa_circRNA_102018 | hsa_circ_0042819 | circBase | chr17 | - | 28003837 | 28022541 | exonic | NM_033389 | SSH2 | GATAACAGAGTTCACATATTCAAACCTGTATCTGTGCAGGCAATGTGGCGATCTCCAACA |
| ASCRP3008465 | 0.319285098 | 0.909237851 | 1.0354124 | down | hsa_circRNA_060930 | hsa_circ_0060930 | circBase | chr20 | - | 52773707 | 52790516 | exonic | NM_000782 | CYP24A1 | GATAATACGCCTCAGGACAGGAGGAAACGCAGCGCCAGCAGCATCTCATCTACCCTCCTT |
| ASCRP3008467 | 0.976023291 | 0.996952313 | 1.002434 | down | hsa_circRNA_101441 | hsa_circ_0033408 | circBase | chr14 | + | 102842986 | 102931626 | exonic | NM_014844 | TECPR2 | GATAATGATTGAGCCACCTGTCCAGCCCCCAGGTTTCCCTAGATGACAAATAAACATTCC |
| ASCRP3008469 | 0.259743071 | 0.909237851 | 1.0578459 | down | hsa_circRNA_407012 | | 25070500 | chr7 | - | 130737183 | 130785946 | intronic | uc011kpk.2 | FLJ43663 | GATAATTGATAACACCCTGTAACAGCTGAGCCTGTCTTCATCCACAACAGCTTGTTCCTA |
| ASCRP3008472 | 0.136001037 | 0.909237851 | 1.0510623 | down | hsa_circRNA_103163 | hsa_circ_0062321 | circBase | chr22 | - | 20800729 | 20825802 | exonic | NM_032775 | KLHL22 | GATACAGGAGGGACGTGCACCAGAGGAATGTTTGCTGGGGGATTGAAGGAGATGGAACAG |
| ASCRP3008475 | 0.461312679 | 0.91119147 | 1.0284115 | down | hsa_circRNA_100397 | hsa_circ_0015358 | circBase | chr1 | - | 175996707 | 175996824 | exonic | NM_022457 | RFWD2 | GATACCATTTGGCTTTCGGCTGTGCAGTGAAGCTGTGGTCTACCAATCTAGACAACTCAG |
| ASCRP3008478 | 0.534597491 | 0.919549314 | 1.024467 | down | hsa_circRNA_092180 | hsa_circ_0092180 | circBase | chrY | + | 150833 | 159885 | exonic | NM_018390 | PLCXD1 | GATACCTGGAGACCATGAAGAGCTGCGGCCGCCCAGGTTGCCCAGGGCTCACCTCTGATG |
| ASCRP3008479 | 0.239826201 | 0.909237851 | 1.0293767 | down | hsa_circRNA_009169 | hsa_circ_0009169 | circBase | chr3 | - | 186988982 | 187003844 | intronic | ENST00000392472 | MASP1 | GATACTGAGTTTCTCATCCCTGCAGCTGATAGTGAAAAGGTGGCTGCTTCTCTATTATGC |
| ASCRP3008483 | 0.084065856 | 0.909237851 | 1.040217 | down | hsa_circRNA_000645 | hsa_circ_0000862 | circBase | chr18 | - | 77693968 | 77695767 | intronic | ENST00000357575 | PQLC1 | GATAGCAAGGATGAAGAAGTCAAGGTTGCCCCCAGGCGGTCCTTCCTGGCCATCTATCTG |
| ASCRP3008485 | 0.379615437 | 0.91119147 | 1.0258304 | down | hsa_circRNA_100707 | hsa_circ_0020313 | circBase | chr10 | + | 126185529 | 126186697 | exonic | NM_022126 | LHPP | GATAGGAGTGGAAGCCCACCAGGCGTTACTACAAGGAGACCTCTGGCCTGATGCTGGACG |
| ASCRP3008491 | 0.622774818 | 0.929544944 | 1.0693455 | down | hsa_circRNA_404837 | | 25070500 | chr11 | - | 3777940 | 3778768 | intronic | ENST00000324932 | NUP98 | GATATATATGATGTCTCCCCAAAAAACAAAAACAAGCCGAGTGTGGTGTGAACCACCACG |
| ASCRP3008492 | 0.240724364 | 0.909237851 | 1.0386605 | down | hsa_circRNA_032547 | hsa_circ_0032547 | circBase | chr14 | - | 74756185 | 74757202 | exonic | NM_005050 | ABCD4 | GATATATCCCCTGAAGGAGGTCTACCCCGACTCAGACCCCCAGGGTGGCCAGCGGCAGAG |
| ASCRP3008493 | 0.483577267 | 0.91119147 | 1.0572998 | down | hsa_circRNA_101877 | hsa_circ_0004519 | circBase | chr16 | - | 74670243 | 74671868 | exonic | NM_018124 | RFWD3 | GATATCACAGCCTTCTCCTCAGGCCTCTTTTCTTCCAGTTCCCTACTGAAGGAACAGATG |
| ASCRP3008495 | 0.30867526 | 0.909237851 | 1.0651796 | down | hsa_circRNA_100635 | hsa_circ_0008856 | circBase | chr10 | + | 82266983 | 82269227 | exonic | NM_030927 | TSPAN14 | GATATCGATCTGCAAAACCTCATCGACTCCCTTCAGAAAGCTGGTGTGCTGTCCGACCTC |
| ASCRP3008504 | 0.381748367 | 0.91119147 | 1.0313281 | down | hsa_circRNA_074410 | hsa_circ_0074410 | circBase | chr5 | - | 145533296 | 145533373 | exonic | NM_020117 | LARS | GATATTGCTGCCCTCAGAGACTTGAAGAAAAAGCAAGCACTGGTGTGGTTACAAGTGTTC |
| ASCRP3008506 | 0.083846638 | 0.909237851 | 1.0959286 | down | hsa_circRNA_100188 | hsa_circ_0011950 | circBase | chr1 | - | 42041214 | 42050989 | exonic | NM_024503 | HIVEP3 | GATCAAAATCTTCGAAGGAGGAAGAAGAGCGTGATTTCTCCTCCAAGGCCGTTGATCTCC |
| ASCRP3008512 | 0.504129621 | 0.914489113 | 1.0153118 | down | hsa_circRNA_404966 | | 25070500 | chr12 | - | 12397199 | 12397589 | intronic | ENST00000535731 | LRP6 | GATCAACCCAGAGCTATTGCCTTAGATCCTTCAACGGCCCCTTTGTTGCTTTATGCAAAC |
| ASCRP3008528 | 0.81138327 | 0.974944807 | 1.0067746 | down | hsa_circRNA_002792 | hsa_circ_0002792 | circBase | chr21 | - | 47734629 | 47735461 | exonic | NM_058180 | C21orf58 | GATCATCCTGCCTACGGAAATGAGGACCGGCCGGACGATGCCCTGCAGACTGCTCTGAAG |
| ASCRP3008534 | 0.31292742 | 0.909237851 | 1.0445954 | down | hsa_circRNA_012761 | hsa_circ_0012761 | circBase | chr1 | + | 61872233 | 61892228 | exonic | NM_001134673 | NFIA | GATCCAAGCTTTGTAAATATCCCTCAACAGACACAGCCCAATGGGAGCAGCCAAGGCAAG |
| ASCRP3008550 | 0.843890156 | 0.978270321 | 1.0072809 | down | hsa_circRNA_100695 | hsa_circ_0005516 | circBase | chr10 | - | 121275020 | 121286936 | exonic | NM_002925 | RGS10 | GATCCTGGAAGAACCGCACCCTCTGATGTTCCAGAAACTCCAGGACCAGACATCCACGAC |
| ASCRP3008551 | 0.890977534 | 0.979863126 | 1.0051881 | down | hsa_circRNA_101673 | hsa_circ_0005483 | circBase | chr16 | - | 929569 | 961079 | exonic | NR_033645 | LMF1 | GATCCTGTTCCAGGATGGGAGTCCCAGCTTCTGGAGACGGGGTTCCTGGGGATCTTCCTG |
| ASCRP3008554 | 0.621708939 | 0.929544944 | 1.0196392 | down | hsa_circRNA_400155 | | 25242744 | chr1 | - | 21586763 | 21599404 | exonic | NM_001397 | ECE1 | GATCGAGGAGCTCAGGGCCAAACCTCTAATGGAGTTGATTGAGAGGGATCCCCCTCTGTG |
| ASCRP3008556 | 0.620692845 | 0.929544944 | 1.0136523 | down | hsa_circRNA_102162 | hsa_circ_0045202 | circBase | chr17 | + | 61655830 | 61657304 | exonic | NM_005828 | DCAF7 | GATCGCCCATGACAAAGAGGTTCAGCTTGTTGGTTTAGATGAGGAGAGTTCAGAGTTTAT |
| ASCRP3008564 | 0.171459036 | 0.909237851 | 1.0606401 | down | hsa_circRNA_102220 | hsa_circ_0009101 | circBase | chr17 | - | 79244717 | 79258695 | exonic | NM_138570 | SLC38A10 | GATCTACAAGAAAATCCACAAGAACGCACTTTCCTCCCAGCATTCCACGCCTACGGGAAG |
| ASCRP3008566 | 0.239502831 | 0.909237851 | 1.1303435 | down | hsa_circRNA_104740 | hsa_circ_0086444 | circBase | chr9 | - | 19070216 | 19076702 | exonic | NM_017645 | HAUS6 | GATCTAGCCAACAGTCTGTAGATCTTTTACCACCAATGTCTCCCCTTTCGTTTGATCCTG |
| ASCRP3008578 | 0.914186205 | 0.98346125 | 1.0034659 | down | hsa_circRNA_000878 | hsa_circ_0000878 | circBase | chr19 | - | 4589738 | 4589839 | intergenic |  |  | GATCTGAGCTCGACACCCTTGATGGCCTCTCTGCACACTGTCAGTGTCGGAGGTTTTGTA |
| ASCRP3008584 | 0.89377521 | 0.98039597 | 1.0044599 | down | hsa_circRNA_100365 | hsa_circ_0014798 | circBase | chr1 | + | 156848913 | 156849949 | exonic | NM_002529 | NTRK1 | GATCTTCACCTACGGCAAGCAGCCCTGGTACCAGCTCTCCAACACGGAGATCCCATGGAC |
| ASCRP3008589 | 0.103041298 | 0.909237851 | 1.0471149 | down | hsa_circRNA_100802 | hsa_circ_0009018 | circBase | chr11 | + | 44129232 | 44135851 | exonic | NM_000401 | EXT2 | GATCTTCCAGAGAAAGGACCAGGGAGTGTGAGGAAGAGGCTGTCTGTGTCATTATGTGTG |
| ASCRP3008591 | 0.166900948 | 0.909237851 | 1.0999731 | down | hsa_circRNA_103664 | hsa_circ_0003534 | circBase | chr4 | - | 76902524 | 76903190 | exonic | NM_018115 | SDAD1 | GATCTTCTCTCCTGCAATCATACCGTATTGGATCCAGATCTGCGAATGTTTCTACAGCAG |
| ASCRP3008593 | 0.899368205 | 0.981257955 | 1.0214283 | down | hsa_circRNA_102805 | hsa_circ_0056146 | circBase | chr2 | + | 113321942 | 113322076 | exonic | NM_019014 | POLR1B | GATCTTGCTCCAGGCATCGCAGATTCTCTTCGTCATTTTAAGGGGTCACTCCCATTGATG |
| ASCRP3008600 | 0.621163051 | 0.929544944 | 1.0199231 | down | hsa_circRNA_102461 | hsa_circ_0003935 | circBase | chr19 | - | 13443682 | 13445307 | exonic | NM_000068 | CACNA1A | GATGAAACTGACGGGGAGCAGAGGCATCCCTTTGATGGGAGTTTGCCAAAGAAAGGGAAC |
| ASCRP3008610 | 0.576671609 | 0.923965283 | 1.0658238 | down | hsa_circRNA_100043 | hsa_circ_0009624 | circBase | chr1 | - | 8926337 | 8927309 | exonic | NM_001428 | ENO1 | GATGAAGGCGGGTTTGCTCCCAACATCCTGGAGAATAAAGAAGCTAAGTTTGGTGCGAAC |
| ASCRP3008626 | 0.856165171 | 0.978983332 | 1.0066192 | down | hsa_circRNA_003040 | hsa_circ_0003040 | circBase | chr13 | + | 24823614 | 24826000 | exonic | NM_153023 | SPATA13 | GATGAGAGCATCCAACGGTCGTCCCTGATGGCCCCTGGAGGCGAAGCTCATCACAGGATG |
| ASCRP3008641 | 0.122173234 | 0.909237851 | 1.111368 | down | hsa_circRNA_016199 | hsa_circ_0016199 | circBase | chr1 | - | 205138290 | 205156934 | exonic | NM_015375 | DSTYK | GATGATGCTACTAACATGGAGTTTAAAGGCCAACTGAGCTGCATTTCCTTCCCACCTAAG |
| ASCRP3008651 | 0.166520152 | 0.909237851 | 1.1332271 | down | hsa_circRNA_101144 | hsa_circ_0028196 | circBase | chr12 | - | 110826316 | 110834257 | exonic | NM_016238 | ANAPC7 | GATGCCATTCTAGTGAGTTATTCTCCCCACCTCAGAAGTACCAGCTTTTGGTGTATCATG |
| ASCRP3008655 | 0.083950065 | 0.909237851 | 1.1045564 | down | hsa_circRNA_030296 | hsa_circ_0030296 | circBase | chr13 | - | 51957464 | 51963580 | exonic | NM_012141 | INTS6 | GATGCCTTACAATTATCCAGTCCTTCTTCCCCTCTTAGGCCGTTCATATTCTGTGTGTTC |
| ASCRP3008660 | 0.641224552 | 0.929544944 | 1.0233282 | down | hsa_circRNA_002532 | hsa_circ_0002532 | circBase | chrX | + | 64295353 | 64295792 | sense overlapping | TCONS_00017187 | XLOC_008000 | GATGCTATGCTAAGCTGGGCAGAGAGACCATGGCGACATCCCCCAGACATCTAGACTATG |
| ASCRP3008664 | 0.150234033 | 0.909237851 | 1.0999766 | down | hsa_circRNA_406620 | | 25070500 | chr5 | - | 72850146 | 72857113 | exonic | NM_023039 | ANKRA2 | GATGCTCTTAGCTGAATGCAATATCCATACATCTCCTTCTCCGGGAATTCAAGTAAGGCA |
| ASCRP3008667 | 0.752961304 | 0.957534606 | 1.010248 | down | hsa_circRNA_102978 | hsa_circ_0004525 | circBase | chr20 | + | 398169 | 398574 | exonic | NM_006462 | RBCK1 | GATGCTGGAAGGCTGTGGGTGAGCGTGGAGGATGCTCAGATGCACACCGTCACCATCTGG |
| ASCRP3008675 | 0.515307738 | 0.916971445 | 1.063927 | down | hsa_circRNA_407051 | | 25070500 | chr8 | - | 28654060 | 28671129 | exonic | NM_018250 | INTS9 | GATGGAATGGTGGGAGAGTTCTGCAGCAACCTAGGCTGTTACCTTCTCCTCTCAAGGATG |
| ASCRP3008676 | 0.072591767 | 0.909237851 | 1.0985656 | down | hsa_circRNA_100875 | hsa_circ_0023397 | circBase | chr11 | + | 71201890 | 71202949 | exonic | NM_018161 | NADSYN1 | GATGGACAGGTGTCCCAGACCGACGAGTCTCCTGGGCTACCTGACCAAGTACGACTGCTC |
| ASCRP3008681 | 0.613233297 | 0.929544944 | 1.0158956 | down | hsa_circRNA_069334 | hsa_circ_0069334 | circBase | chr4 | - | 24875302 | 24878319 | exonic | NM_173463 | CCDC149 | GATGGAGATCCATCACTTCCTCCTGAAAAAAGGAAACAGTACCTGGTGTGTAAGAGGAAG |
| ASCRP3008687 | 0.009856543 | 0.909237851 | 1.1473638 | down | hsa_circRNA_101780 | hsa_circ_0038773 | circBase | chr16 | - | 28112778 | 28113266 | exonic | NM_015171 | XPO6 | GATGGATCGGGCTTTCGGACAGTCCTTTCTCCAGCCCGACATCCACCTTTTTAAACAAAA |
| ASCRP3008690 | 0.367054273 | 0.91119147 | 1.1250453 | down | hsa_circRNA_105037 | hsa_circ_0091710 | circBase | chrX | - | 151130894 | 151131115 | exonic | NM_004961 | GABRE | GATGGCAAGGTGTTGTACACAATTAGGAATACACCATTGACATCATCTTCTCCCAGACCT |
| ASCRP3008692 | 0.424667374 | 0.91119147 | 1.0171202 | down | hsa_circRNA_101404 | hsa_circ_0032664 | circBase | chr14 | - | 75563802 | 75590926 | exonic | NM_033116 | NEK9 | GATGGCATACCATTCTCATCGTTGGATGACTCACTGGTTGTGTGGAAGGAAGTCGATTTG |
| ASCRP3008693 | 0.146400949 | 0.909237851 | 1.0431751 | down | hsa_circRNA_103963 | hsa_circ_0001538 | circBase | chr5 | + | 138699447 | 138700432 | exonic | NM_016480 | PAIP2 | GATGGCTCTTCTCTGGAAGATCTTGTGGTTAAAAACGACAACCAACATCAGCCATGAAAG |
| ASCRP3008706 | 0.11488348 | 0.909237851 | 1.0631506 | down | hsa_circRNA_045308 | hsa_circ_0045308 | circBase | chr17 | + | 62515438 | 62522318 | exonic | NM_138363 | CEP95 | GATGTATCAGAAAAACTCTCTCAGCGGCTTTCTGAACTAGATTGGGTGCTCCTTGTCTTC |
| ASCRP3008708 | 0.235626505 | 0.909237851 | 1.0591559 | down | hsa_circRNA_092441 | hsa_circ_0000764 | circBase | chr17 | - | 40176084 | 40176486 | antisense | NM_017595 | NKIRAS2 | GATGTCAACCAAGAAAAACTACACTCCCAGGAGGCCAACTTCTTCCAGCCAAGAAAAGGA |
| ASCRP3008716 | 0.800052275 | 0.972258433 | 1.0107035 | down | hsa_circRNA_102954 | hsa_circ_0003923 | circBase | chr2 | + | 238933982 | 238940895 | exonic | NM_080678 | UBE2F | GATGTCGTTTGGGGATTAAACTCTTTGTTTACTCCTCCCAAAGTGAAATGCCTGACCAAG |
| ASCRP3008723 | 0.548241343 | 0.919549314 | 1.028896 | down | hsa_circRNA_400319 | | 25242744 | chr1 | - | 151111900 | 151112566 | exonic | NM_030913 | SEMA6C | GATGTGGAGAACTGTGCTGTACGGGGAAAGCTGACGGTACTTCCCCATTATCCTGGTTTC |
| ASCRP3008724 | 0.251923668 | 0.909237851 | 1.1541334 | down | hsa_circRNA_103759 | hsa_circ_0071224 | circBase | chr4 | - | 151771875 | 151789457 | exonic | NM_006726 | LRBA | GATGTGGAGAAGAGATACTAAAAGTAATTGCGACCCTACTTCGAAATTCTCCCCAGTGCC |
| ASCRP3008726 | 0.786941775 | 0.967162564 | 1.0139908 | down | hsa_circRNA_401760 | | 25242744 | chr17 | + | 27794162 | 27809306 | exonic | NM_020791 | TAOK1 | GATGTGTGGTCTCTTGGAATAACATGTATTGAACTAGGCACGAGATGTGCGTACCAATGA |
| ASCRP3008727 | 0.33674979 | 0.91119147 | 1.0349164 | down | hsa_circRNA_402318 | | 25242744 | chr2 | - | 97373450 | 97400263 | exonic | NM_030805 | LMAN2L | GATGTGTTCTTGCCCTCAGTGGACAATATGAAGCTGCCTGAGACCATGTTTCCTGAGAGA |
| ASCRP3008730 | 0.189632662 | 0.909237851 | 1.0775513 | down | hsa_circRNA_061376 | hsa_circ_0061376 | circBase | chr21 | - | 30428647 | 30446010 | exonic | uc002ynb.3 | CCT8 | GATGTTATAATAAACATATTGTTACTGTCAAGAGGCGGCCCCACGCTGCTTTCCCAGAAG |
| ASCRP3008741 | 0.148638416 | 0.909237851 | 1.0485757 | down | hsa_circRNA_103400 | hsa_circ_0066336 | circBase | chr3 | + | 58104598 | 58112489 | exonic | NM_001457 | FLNB | GATTACGATGTTAATATCACATATGGAGGAGCCCACATCCCCGGGCAACATGCAGGTTCT |
| ASCRP3008742 | 0.540200994 | 0.919549314 | 1.0570327 | down | hsa_circRNA_103401 | hsa_circ_0003006 | circBase | chr3 | + | 58111307 | 58112489 | exonic | NM_001457 | FLNB | GATTACGATGTTAATATCACATATGGAGGAGCCCACATCCCCGGTCTCCATGTAGTGGAG |
| ASCRP3008745 | 0.939953527 | 0.989295564 | 1.0025282 | down | hsa_circRNA_000230 | hsa_circ_0000765 | circBase | chr17 | - | 40266710 | 40266909 | intronic | ENST00000225916 | KAT2A | GATTAGGTGGCGTTGCAGCTGGGTGGTGTGCACACATGCGCATCTGTGGGATTTTTCATG |
| ASCRP3008748 | 0.25732518 | 0.909237851 | 1.0871748 | down | hsa_circRNA_401461 | | 25242744 | chr15 | + | 73570471 | 73575452 | exonic | NM_002499 | NEO1 | GATTATCGCTGTCTTTTGTACCCGTCGTACCACCTCTCACCAGAAAAACCTCAGGGTCTG |
| ASCRP3008749 | 0.157616956 | 0.909237851 | 1.0406423 | down | hsa_circRNA_100697 | hsa_circ_0020246 | circBase | chr10 | - | 123600596 | 123683844 | exonic | NM_007041 | ATE1 | GATTATTCGTTTTTGTCTTTGGGCGTCTACTCTGCACTACGGCATGTGGGCACATTCCAT |
| ASCRP3008754 | 0.479079189 | 0.91119147 | 1.0261249 | down | hsa_circRNA_103644 | hsa_circ_0007476 | circBase | chr4 | + | 54280781 | 54294350 | exonic | NM_030917 | FIP1L1 | GATTCCACCACCGGAAACAGCACTTCTTCTCAGTCTCAGACAAGTACTGCCTCCAGAAAA |
| ASCRP3008761 | 0.776480887 | 0.965028024 | 1.0138408 | down | hsa_circRNA_001015 | hsa_circ_0001015 | circBase | chr2 | + | 61632920 | 61633008 | antisense | NM_014709 | USP34 | GATTCCTCCTCAGTCAGATTACAAATTCTTTAAGAGTTTTTCCTTCTCATCTGTACTCCA |
| ASCRP3008762 | 0.253711857 | 0.909237851 | 1.0489599 | down | hsa_circRNA_003806 | hsa_circ_0003806 | circBase | chr3 | - | 87017739 | 87018273 | exonic | NM_016206 | VGLL3 | GATTCGATACAGACAGCTCAGCTCTCTCAAGCCAGCGGAATAGTTTCCCAACTTCCTTTT |
| ASCRP3008773 | 0.900076236 | 0.981257955 | 1.0067283 | down | hsa_circRNA_103801 | hsa_circ_0071989 | circBase | chr5 | - | 16683988 | 16764505 | exonic | NM_012334 | MYO10 | GATTGATTCTGTGTGTGCCTCTGACAGCCCTGATAGGCAGTAGACAGCAGGGACTCCCTG |
| ASCRP3008776 | 0.671471565 | 0.938080316 | 1.0602015 | down | hsa_circRNA_101465 | hsa_circ_0034189 | circBase | chr15 | - | 28090104 | 28096621 | exonic | NM_000275 | OCA2 | GATTGCAGAACAGCATGGATATGGGTTCTCCTTCATGGAATTTTTCAGATTCCCGTGCTC |
| ASCRP3008784 | 0.682449872 | 0.941949564 | 1.0205839 | down | hsa_circRNA_101384 | hsa_circ_0032391 | circBase | chr14 | + | 71428942 | 71479919 | exonic | NM_014982 | PCNX | GATTGGCATTAACTTTGACAGACTCACACTTTTGGCCCTGTTTGATAGTGATCCTGGTGG |
| ASCRP3008794 | 0.262220719 | 0.909237851 | 1.0665557 | down | hsa_circRNA_028184 | hsa_circ_0028184 | circBase | chr12 | - | 110819556 | 110819753 | exonic | NM_016238 | ANAPC7 | GATTGTTATGAAGCTGTCACAGCTTCTATAGCAAACGCTACTCCCGGGCCCTCTATTTAG |
| ASCRP3008813 | 0.377541342 | 0.91119147 | 1.0531731 | down | hsa_circRNA_104252 | hsa_circ_0001661 | circBase | chr6 | + | 159001971 | 159010814 | exonic | NM_020823 | TMEM181 | GATTTGAACACCTGAAGCTCCCCATCAAGGGAATGAACTTCACAGACCTAAAGTGATCCA |
| ASCRP3008816 | 0.029310123 | 0.909237851 | 1.1106834 | down | hsa_circRNA_405333 | | 25070500 | chr15 | + | 45059428 | 45059793 | sense overlapping | NM_080745 | TRIM69 | GATTTGCCTTCTTTCAGTCTGACACTGACTAACAACCTCGACAAGGCCTGTCTCCACTAA |
| ASCRP3008819 | 0.400630111 | 0.91119147 | 1.0276575 | down | hsa_circRNA_018572 | hsa_circ_0018572 | circBase | chr10 | + | 70723046 | 70731808 | exonic | NM_004728 | DDX21 | GATTTGGTTATACAAAGCTCTCCACCAAAGGCCGAGGAGTGACCTTCCTATTTCCTATAC |
| ASCRP3008822 | 0.483587932 | 0.91119147 | 1.1432416 | down | hsa_circRNA_090069 | hsa_circ_0090069 | circBase | chrX | + | 22108546 | 22117269 | exonic | NM_000444 | PHEX | GATTTGTTTAGGATATTAGGGTCTGAGAGAAAGAACTGGACCAAGCAACACTCTCCCTGG |
| ASCRP3008830 | 0.760267067 | 0.960870011 | 1.0113558 | down | hsa_circRNA_102436 | hsa_circ_0049164 | circBase | chr19 | + | 9939267 | 9939550 | exonic | NM_024292 | UBL5 | GATTTTTAAGGACCACGTGTCTCTGGGGGACTCACGGATGATACCATCGGGGACCTTAAG |
| ASCRP3008844 | 0.131017209 | 0.909237851 | 1.0694845 | down | hsa_circRNA_405249 | | 25070500 | chr14 | + | 67720920 | 67722005 | intronic | ENST00000261681 | MPP5 | GCAAAGCAATAGCCATATTTTAAGTTTCTTAAGGATACGGACTCTGCCTCAGCAGCCTTT |
| ASCRP3008846 | 0.5711386 | 0.922333971 | 1.0164273 | down | hsa_circRNA_100626 | hsa_circ_0018848 | circBase | chr10 | - | 75472961 | 75481262 | exonic | NR_026592 | BMS1P4 | GCAAAGTATTAGTTGCCAAGCTGTTCTGCCTTTCTGGAATGGTGCATGGAGAATATCAAA |
| ASCRP3008850 | 0.83795277 | 0.978181218 | 1.004925 | down | hsa_circRNA_105028 | hsa_circ_0008350 | circBase | chrX | - | 118774654 | 118787003 | exonic | NM_015129 | SEPT6 | GCAACAAGATGATGAGGGCGCGGCAGTATCCTTGGGGCACTGTGCAGGCTACAAGCCTAT |
| ASCRP3008854 | 0.975162097 | 0.996425773 | 1.0012752 | down | hsa_circRNA_084135 | hsa_circ_0084135 | circBase | chr8 | - | 42716887 | 42743014 | exonic | NM_030954 | RNF170 | GCAACCCAGATCTACCTGGAATGGCCAAATATCAAGGTGAAGTTCAAAGTTTGAAACTGG |
| ASCRP3008873 | 0.273455253 | 0.909237851 | 1.0573264 | down | hsa_circRNA_101487 | hsa_circ_0008926 | circBase | chr15 | + | 41648236 | 41668026 | exonic | NM_016359 | NUSAP1 | GCAAGTTTGTCTCGTCCCCTCAACTATGAACCACACAAAGGTAACAGAGATTCAAAGGTA |
| ASCRP3008884 | 0.93633505 | 0.987920247 | 1.005728 | down | hsa_circRNA_000635 | hsa_circ_0000635 | circBase | chr15 | - | 75684855 | 75687163 | sense overlapping | NM_015477 | SIN3A | GCACAATGGTGTTGGGGGCAGCTTTAACAAAGTATGGCGAGAACAAAATGAGAAATACTA |
| ASCRP3008897 | 0.950085664 | 0.992077624 | 1.008121 | down | hsa_circRNA_000850 | hsa_circ_0000850 | circBase | chr18 | + | 48422178 | 48452268 | exonic | NM_002396 | ME2 | GCACGGCTGAAGAAGCATATACACTTACAGAGGTGAAAGAAAAGATGTTGTCCCGGTTAA |
| ASCRP3008898 | 0.649748243 | 0.932467957 | 1.0290843 | down | hsa_circRNA_104872 | hsa_circ_0088045 | circBase | chr9 | - | 114860749 | 114864565 | exonic | NM_022486 | SUSD1 | GCACTCAGTGCAAATAACAATAGCAACTCCCCCAGCAGAAGTTGATCTCTTAGAAGATGA |
[truncated: 176,190 more chars]
